# A Field Guide to Pandemic, Epidemic and Sporadic Clones of Methicillin-Resistant *Staphylococcus aureus*

**DOI:** 10.1371/journal.pone.0017936

**Published:** 2011-04-06

**Authors:** Stefan Monecke, Geoffrey Coombs, Anna C. Shore, David C. Coleman, Patrick Akpaka, Michael Borg, Henry Chow, Margaret Ip, Lutz Jatzwauk, Daniel Jonas, Kristina Kadlec, Angela Kearns, Frederic Laurent, Frances G. O'Brien, Julie Pearson, Antje Ruppelt, Stefan Schwarz, Elizabeth Scicluna, Peter Slickers, Hui-Leen Tan, Stefan Weber, Ralf Ehricht

**Affiliations:** 1 Institute for Medical Microbiology and Hygiene, Technical University of Dresden, Dresden, Germany; 2 Department of Microbiology and Infectious Diseases, PathWest Laboratory Medicine - WA, Royal Perth Hospital, Perth, Western Australia, Australia; 3 Microbiology Research Unit, Dublin Dental University Hospital, University of Dublin, Trinity College, Dublin, Ireland; 4 Department of Para-Clinical Sciences, The University of the West Indies, St. Augustine, Trinidad and Tobago; 5 Infection Control Unit, Mater Dei Hospital, Msida, Malta; 6 Department of Microbiology, Faculty of Medicine, The Chinese University of Hong Kong, Shatin, Hong Kong, China; 7 Infection Control Unit, Dresden University Hospital, Dresden, Germany; 8 Department of Environmental Health Sciences, Freiburg University Medical Centre, Freiburg, Germany; 9 Institute of Farm Animal Genetics, Friedrich-Loeffler-Institut (FLI), Neustadt-Mariensee, Germany; 10 Staphylococcus Reference Unit, Centre for Infections, Health Protection Agency, London, United Kingdom; 11 Université Lyon, Centre National de Référence des Staphylocoques, Lyon, France; 12 School of Biomedical Sciences, Curtin University, Perth, Western Australia, Australia; 13 Alere Technologies GmbH, Jena, Germany; 14 Sheikh Khalifa Medical City, Abu Dhabi, United Arab Emirates; Columbia University, United States of America

## Abstract

In recent years, methicillin-resistant *Staphylococcus aureus* (MRSA) have become a truly global challenge. In addition to the long-known healthcare-associated clones, novel strains have also emerged outside of the hospital settings, in the community as well as in livestock. The emergence and spread of virulent clones expressing Panton-Valentine leukocidin (PVL) is an additional cause for concern. In order to provide an overview of pandemic, epidemic and sporadic strains, more than 3,000 clinical and veterinary isolates of MRSA mainly from Germany, the United Kingdom, Ireland, France, Malta, Abu Dhabi, Hong Kong, Australia, Trinidad & Tobago as well as some reference strains from the United States have been genotyped by DNA microarray analysis. This technique allowed the assignment of the MRSA isolates to 34 distinct lineages which can be clearly defined based on non-mobile genes. The results were in accordance with data from multilocus sequence typing. More than 100 different strains were distinguished based on affiliation to these lineages, SCC*mec* type and the presence or absence of PVL. These strains are described here mainly with regard to clinically relevant antimicrobial resistance- and virulence-associated markers, but also in relation to epidemiology and geographic distribution. The findings of the study show a high level of biodiversity among MRSA, especially among strains harbouring SCC*mec* IV and V elements. The data also indicate a high rate of genetic recombination in MRSA involving SCC elements, bacteriophages or other mobile genetic elements and large-scale chromosomal replacements.

## Introduction

*Staphylococcus aureus* is a ubiquitous bacterium colonising 20–30% of the human population [1]. Beyond asymptomatic carriage, *S. aureus* causes a wide range of infections, such as skin and soft tissue infections (SSTI), bone, joint and implant infections, pneumonia, septicaemia and various toxicoses such as toxic shock syndrome. It also occurs in many different species of animals, where it may cause comparable disease such as bovine mastitis.

Shortly after the introduction of penicillin in the 1940s, the first penicillinase-producing *S. aureus* strains were detected, leading to the development of the penicillinase-resistant semi-synthetic penicillins such as methicillin, oxacillin and the first/second generation cephalosporins. Within a year after the introduction of these drugs, methicillin-resistant *S. aureus* (MRSA) were reported in the United Kingdom (UK) [2]. Resistance is due to a modified penicillin binding protein (PBP2' or PBP2a) encoded by the *mecA* gene. Apart from ceftobiprole [3], the presence of PBP2a confers resistance towards all β-lactam antibiotics. As methicillin and oxacillin can be used as indicators of resistance, PBP2a- or *mecA-*positive *S. aureus* are referred to as either methicillin-resistant *S. aureus* (MRSA) or oxacillin-resistant *S. aureus* (ORSA). The *mecA* gene is located on complex mobile genetic elements, known as SCC*mec* (“staphylococcal cassette chromosome” or “staphylococcal chromosomal cassette” harbouring *mecA*). In addition to *mecA*, SCC*mec* elements comprise recombinase genes, regulatory elements and, variably, additional genes encoding resistance to other antimicrobials, such as aminoglycosides or macrolides, and to heavy metal ions [4]; [ 5]. SCC*mec* types I, II and III (or 1B, 2A, 3A, [6]) are typically restricted to MRSA strains associated with healthcare infections and are not found widely among the healthy population. These strains, which initially were known as “Epidemic MRSA” (EMRSA), are now frequently referred to as “hospital-acquired” or “healthcare-associated” MRSA (HA-MRSA). The presence of their SCC*mec* elements correlates with a relatively slower growth rate and it has been assumed that they may confer a selective disadvantage in the absence of antibiotics [7]; [ 8]. Subsequently, strains carrying these elements may be less fit to survive in a competitive environment with faster growing wild type strains once antibiotic therapy is discontinued.

The epidemiology of MRSA has changed since the 1990s with the emergence of new SCC*mec* elements such as types IV and V (2B, 5C2). Strains carrying these elements evolved predominantly outside of healthcare settings or proved capable of spreading outside of hospitals, infecting not only patients but also colonising healthy contact persons. Many different strains of so-called “community-acquired” or “community-associated” MRSA (CA-MRSA) have spread worldwide. Some CA-MRSA strains harbour genes encoding the bi-component toxin Panton-Valentine leukocidin (PVL, [9]). Although this toxin was identified in *S. aureus* as early as 1932 [10], its presence in MRSA is a very recent phenomenon [11]. These strains are frequently associated with chronic/recurrent SSTI as well as with life-threatening necrotising pneumonia [12], often in previously healthy young people. PVL-positive CA-MRSA have become a serious public health concern because of their virulence, their ability to cause outbreaks in households and close contact social groups, and their rapid spread in many countries.

Since 2003, some notable MRSA strains carrying SCC*mec* IV or V have spread among livestock revealing the truly zoonotic potential of *S. aureus*/MRSA. These strains have been dubbed “livestock-associated“ MRSA (LA-MRSA, [13]).

Additional novel SCC*mec* elements have been described [6]; [14]–[17] which indicate an ongoing evolution of antibiotic resistance in *S. aureus*. As β-lactam antibiotics are the first-line compounds for treatment of staphylococcal infections, this development may significantly limit therapeutic options. Alternative drugs that may be used to treat MRSA infections are generally expensive (*e.g.,* quinupristin-dalfopristin, tigecycline, daptomycin and linezolid), or are problematic with regard to tissue penetration and efficiency (*e.g.,* vancomycin) or toxicity (*e.g.,* rifampicin). Of further concern is that resistance of MRSA to these compounds has already been observed, *e.g., vanA*-mediated vancomycin resistance [18] and *cfr*-mediated linezolid resistance [19]–[21].

The limited choice of therapeutic options available has made it necessary to attempt to limit the spread of MRSA by using a range of infection prevention and control measures such as hand hygiene, the use of protective clothing and equipment (*e.g.,* examination gloves or face masks), and accommodation of patients in isolation rooms or wards. The cost of these measures as well as the significant expense of second-line antimicrobials places a serious economic burden on scarce healthcare resources. Recent studies from Europe indicated that the average excess costs per MRSA-positive patient range from €5,700 to €10,000 [22]–[24]. Given the large number of MRSA carriers in Europe and in the USA, the financial burden to healthcare in these regions may account for billions of Euro or dollars (http://www.infectioncontroltoday.com/hotnews/55h168584264313.html).

MRSA have become a real international problem. Some strains predominate in geographically restricted settings while others have achieved pandemic spread. Since a bewildering biodiversity of novel strains and SCC*mec* elements has been described in recent years, the objective of this study is to summarise the genotypic characteristics of pandemic, epidemic and sporadic MRSA strains from different parts of the world based on the authors' genotyping experiments and on the published literature.

## Results

**Figure 1:**
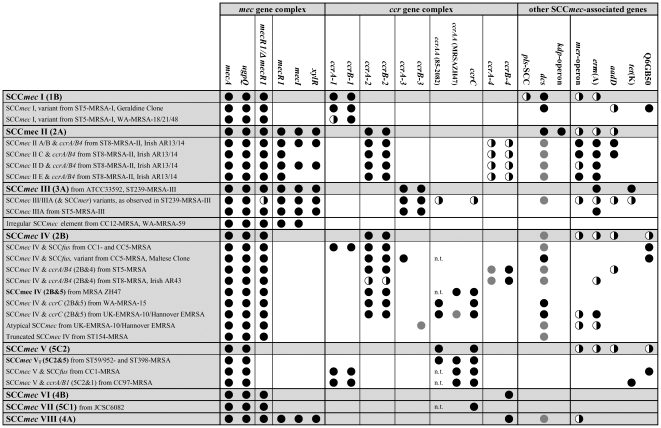
Overview of SCC*mec* elements, their variants and DNA microarray hybridisation patterns. Black circles, positive; grey circles, present, but yielding weak or ambiguous signals; divided circles, variable genes.

**Figure 2:**
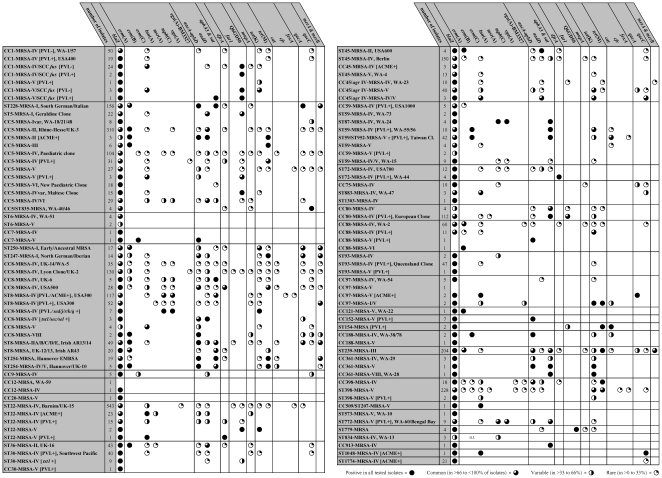
Resistance genes in MRSA strains.

**Figure 3:**
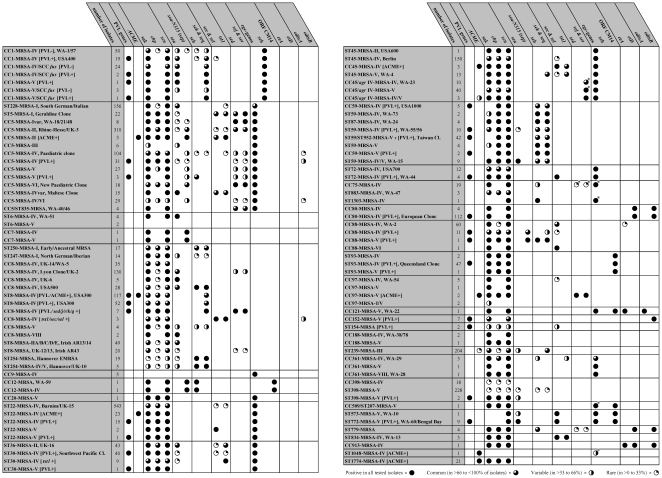
Virulence-associated genes in MRSA strains (*, see text for further explanation).

Information on target genes, probes and primers is provided in Supplemental file S1. The sequence types (ST, as defined by multilocus sequence typing, or MLST, [25]), *spa*, SCC*mec*, capsule and *agr* types associated with each clonal complex (CC) as well as the names and accession numbers of associated whole genome sequenced strains are shown in Supplemental file S2. An overview of SCC*mec* types and their array hybridisation patterns is provided in Figure 1. The antimicrobial resistance and virulence-associated genes of each strain are shown in Figures 2 and 3, respectively, while complete hybridisation profiles for the individual strains are provided in Supplemental file S3.

### Clonal complex 1

CC1 includes several strains of CA-MRSA including MW2/USA400, the first known PVL-positive MRSA [11]. The CC1 strains ST573 and ST772 differ significantly in MLST alleles to all other CC1 isolates and yield deviant hybridisation patterns. Therefore they will be described separately (see below). The remaining CC1-MRSA cluster into seven strains that can be differentiated based on the presence of the PVL genes, SCC*mec* elements IV or V and the SCC*fus* element. The latter comprises *ccrA-1* and *ccrB-1* as well as the fusidic acid resistance marker Q6GD50 (GenBank BX571857.1:SAS0043) first described in the genome sequence of the CC1-MSSA strain Sanger MSSA 476 [26]. All isolates of these CC1-MRSA strains harbour the enterotoxin gene *seh* and many isolates also carry the enterotoxin genes *sek* and *seq*. Other enterotoxin genes (*sea-N315, seb, sec* and *sel*) are only found sporadically. Most isolates harbour the immune evasion cluster (IEC) genes associated with β-haemolysin-converting phages in various combinations.

One CC1-MRSA strain carries SCC*mec* IV and PVL, but lacks SCC*fus*. The genome sequence of MW2 [27] is a representative of this strain, which is also known as USA400 or Canadian MRSA-7 [28]. The first known PVL-positive CA-MRSA belonged to this strain. In the late 1990s, it caused fatal infections in previously healthy children from Minnesota and North Dakota [11]. The authors sporadically found isolates in Australia, Germany and the UK.

A second strain also carries SCC*mec* IV, lacks SCC*fus*, but differs from USA400 in being PVL-negative. It has been found by the authors primarily in Australia where it is designated as West Australian (WA) MRSA-57 although it also includes some of the isolates originally described as WA MRSA-1. It has been isolated sporadically in Saxony (Germany, [29]), Ireland [30] and Egypt (isolate courtesy of M. Kamal El Din, Cairo).

A third strain is PVL-negative, but harbours both, SCC*mec* IV and SCC*fus*. It includes WA MRSA-45 and also some WA MRSA-1 isolates. It has been isolated in Australia as well as, sporadically, in Abu Dhabi (United Arab Emirates [31]), the UK and Ireland [30].

Sporadic isolates of PVL-positive CC1-MRSA-IV with SCC*fus*, PVL-positive CC1-MRSA-V and PVL-negative CC1-MRSA-V with SCC*fus* have been isolated from a small number of cases in Australia. A PVL-positive CC1-MRSA-V with SCC*fus* has been found in Abu Dhabi [31].

### Clonal complex 5

CC5 is another common and widespread clonal complex, which comprises a large number of different MRSA strains, both HA- and CA-MRSA, some of which have attained pandemic spread.

Several whole genome sequences are available (Supplemental file S2) which comprise the features of the CC5 core genome including characteristic allelic variants of the *ssl/set* genes and of genes encoding microbial surface components recognising adhesive matrix molecules of the host (MSCRAMM genes). CC5 isolates carry the enterotoxin gene cluster *egc* (*seg, sei, sem, sen, seo* and *seu/y*), although partial deletions have been observed. β-haemolysin-converting phages carrying *sak, scn* and *chp* as well as *sea* or *sea-N315* in various combinations are commonly detected. Many variable virulence- or resistance-associated genes and several different SCC*mec* elements have been found in CC5.

ST228-MRSA-I is colloquially known as the South German Epidemic Strain, Italian Clone, [32]; [33] or Spanish PFGE types E6/9/15/17/18 [34]. The strain occurs in Germany (where it appears to be a strictly HA-MRSA strain, although its incidence is decreasing), Hungary (where it replaced the “Hungarian Clone” ST239-MRSA-III, [35]), Italy [32]; [33]; [36], Slovenia [36] and Switzerland [37]. It was also found sporadically in Austria [38], Israel [39], Malta [40] and Australia. MLST variants of this strain have been described from Croatia (ST111, [41]) and Paraguay (ST5, ST221, [42]). The isolates harbour SCC*mec* I. A variant lacking *ccrA/B-1* was identified in Saxony in 2000. Variable markers [43] include the mercury resistance operon, the β-lactamase operon as well as several other resistance genes (Figure 2), the toxic shock syndrome toxin *tst1*, IEC genes and the MSCRAMM genes *bbp*, *clfA* and *sdrD*. In contrast to other CC5 strains, ST228-MRSA-I lacks *fnbB*, a gene encoding a fibronectin-binding protein. Deletions of the leukocidin genes *lukD+lukE* and of neighbouring genes from the *egc* locus are common [43].

A ST5-MRSA-I, known as the Geraldine Clone, is a second SCC*mec* I strain from CC5. This is a common CA-MRSA clone in France [44]. Apart from a single veterinary isolate from Germany (courtesy of B. Walther, FU Berlin), isolates have not been detected outside of France. Unlike ST228-MRSA-I, Geraldine Clone isolates harbour Q6GD50, enterotoxin genes *sed, sej* and *ser* and *fnbB*. Most isolates carry *tst1* and the enterotoxin genes *sec* and *sel*. The SCC*mec* element lacks *pls-*SCC.

The Australian strains WA MRSA-18, -21 (both ST5) and -48 (ST835) represent a third group of CC5-MRSA-I. These strains, however, harbour an atypical or truncated SCC*mec* I element. The genes *mecA, ΔmecR1, ugpQ* and *ccrB-1* are detectable; *ccrA-1* occasionally yields a signal and *pls-*SCC is usually absent. Resistance markers detected among these CC5-MRSA-I isolates include the β-lactamase operon and, in some isolates, *qacC* encoding resistance to quaternary ammonium compounds. In addition to *egc,* the enterotoxin genes *sed, sej* and *ser* are associated with these isolates as well as, variably, *sea* or *sea-N315.*

ST5-MRSA-II is a pandemic CC5 strain. A MLST single locus variant (SLV) of this strain, ST225-MRSA-II, has been reported [45]. Different STs as well as a number of additional genetic polymorphisms within this strain might suggest a polyphyletic origin by multiple and independent transfers of SCC*mec* II elements to various CC5-MSSA precursor strains [46]. Synonyms and vernacular names are UK-EMRSA-3, New York-Japan Clone, Rhine-Hesse Epidemic Strain, Irish AR07.3, Irish AR07.4, Irish AR11, USA100 and Canadian MRSA-2 [28]. Published CC5 genome sequences of isolates belonging to this strain include Mu3, Mu50, N315, JH1 and JH9. Isolates of ST5/ST225-MRSA-II have been identified in Austria [38], Croatia [41], Hong-Kong (China, [47]), Hungary [35], Japan [48], Portugal [49], Taiwan [50], the UK and the USA [48]. The authors have identified this strain in Ireland [15] and in different regions of Germany, the UK, Hong Kong and Australia. In Dresden/Saxony, ST5-MRSA-II is the second most prevalent MRSA strain (accounting for approximately 30% of isolates), and the most frequently isolated strain in this region's intensive care units [51]. Transmission of this strain has been reported in Australia where the index case was shown to be a healthcare worker who previously underwent surgery in a New York hospital [52]. ST5-MRSA-II isolates usually exhibit *spa* types t002, t003 or, in Ireland, t045. Antimicrobial resistance markers in ST5/ST225-MRSA-II isolates may vary (see Figure 2), although *erm*(A) and the aminoglycoside resistance gene *aadD* are commonly present. Variable virulence-associated genes include *tst1, sea, sea-N315, sec, sed, sej, sel* and *ser* [43]. Variants of this strain harbouring the arginine catabolic mobile element (ACME) have been isolated in Hong Kong and California/USA (NARSA 642).

CC5-MRSA-III has been isolated from humans in the South African province of KwaZulu-Natal [53], from Korean chicken meat samples [54], and from turkeys or turkey meat in Germany (courtesy of S. Cortez de Jäckel, Delbrück, Germany, and of the Federal Institute for Risk Assessment, Berlin, Germany). The latter isolates harbour SCC*mec* IIIA, lacking *tet*(K) and the mercury resistance operon. CC5-MRSA-III may initially appear to be methicillin-susceptible but resistance is observed following growth on selective agar containing methicillin [54].

CC5-MRSA-IV strains have achieved pandemic spread and significant clinical relevance. Colloquially strains have been described as the Paediatric Clone (although the first strain described under that name HDE288 has been subsequently shown to be CC5-MRSA-VI, [55]), WA MRSA-03 (ST5), -25 (ST575), -39 (ST526), -50 (ST73) and -74 (ST5), USA800 or Marseille Cystic Fibrosis Clone (ST5; [56]). As for ST5/ST225-MRSA-II, a large proportion of CC5-MRSA-IV isolates carry *sea-N315* and/or *sed+sej+ser.* Other virulence genes, including *tst1* and the epidermal cell differentiation inhibitor gene *edinA,* can be found in a minority of isolates. A novel enterotoxin gene has recently been located on the same plasmid as *edinA* (GenBank AP003089.1). Variable resistance markers in CC5-MRSA-IV are shown in Figure 2.

PVL-positive CC5-MRSA-IV has been identified by the authors in patients from the UK, France, Australia, Switzerland and Senegal and previously in Ireland [57]. Based on toxin gene profiles, these isolates cluster into three major variants harbouring *sea-N315+sed+sej+ser* or *sea*+*edinA*, or *edinA.* Variable resistance markers are shown in Figure 2.

ST5-MRSA-V has been described in Australia, where it is colloquially known as WA MRSA-11, -14, -34 and -35, Ireland [30] and Abu Dhabi. PVL-positive CC5-MRSA-V has been identified sporadically in Germany and Abu Dhabi.

CC5-MRSA-VI was first observed in Portugal in 1992 [58]. It was first described as Paediatric Clone carrying SCC*mec* IV but was later reclassified as harbouring a SCC*mec* VI element [55]. In France it was dubbed the New Paediatric Clone [44]. This strain has been identified in Portugal, Colombia, Argentina and the USA [58]. The authors identified New Paediatric Clone isolates (*spa* type t105 or t777) in France, and very sporadically in Australia and Germany, as well as PVL-positive CC5-MRSA-VI (*spa* t311) in Switzerland. The latter strain has also been reported from the Azores and Portugal [59].

A further novel SCC*mec* element (SCC*mec* type VII, [6] or SCC*mec* JCSC6082, GenBank: AB373032.1) was recently described in Sweden [16]. This strain (courtesy of C. Berglund, Stockholm, Sweden) yields signals with probes for *mecA, ΔmecR1, ugpQ* and *ccrC*, and it is positive for *blaZ* and *tet*(M).

In addition to the above mentioned strains, several CC5-MRSA carry multiple or composite SCC*mec* elements. One of these strains (ST149 which is a MLST single locus variant, SLV, of ST5, *spa-*type t002) was recently described in Malta [40]. This strain carries a SCC*mec* IV element as well as Q6GD50 and novel *ccrA* and *ccrB* genes (GenBank GU066221). The latter are identical to *ccrASHP+ccrBSHP* in a Chinese isolate of *S. haemolyticus* (GenBank EU934095.1, [60]), where these genes were accompanied by the ACME locus [61]. This locus, however, is absent in the Maltese strain. It lacks PVL, but it carries *sea*, *egc* as well as, commonly, *tst1, sec* and *sel*. Some isolates harbour the resistance genes *erm*(C) and *tet*(K). This strain appears to be common in Malta [40], but has not been reported elsewhere. Another, PVL- and *tst1*-positive ST149-MRSA-IV has been isolated from a Libyan patient in Switzerland [62].

A composite variant of a SCC*mec* IV has been described in the CC5 strain MRSA-ZH47 [63]. It resembles a SCC*mec* IV element, but also carries a set of SCC*mec* V-like recombinase genes (*ccrC* and “*ccrAA*”, a putative recombinase gene, see Materials and Methods).

Another CC5 strain with multiple/composite SCC*mec* elements originates from Spain. Isolates from a German patient hospitalised in the Canary Islands [29] and from a patient in Barcelona (Courtesy of S. Molinos, Barcelona) yielded hybridisation signals for *mecA, ΔmecR1, ugpQ, ccrA-2, ccrB-2, ccrA-4* and *ccrB-4*. Because of the carriage of *erm*(C), *msr*(A), *mph*(C), *aphA3* and *sat* as well as affiliation to *spa* type t067, these isolates probably represent an epidemic strain, or a close relative of it, recently described in Spain [64]. Similar isolates harbouring only *erm*(C) have been found by the authors in France, Australia and Germany.

A CC5 strain with a composite SCC*mec* element comprising SCC*mec* IV, *ccrA-1, ccrB-1* and Q6GD50 has recently been described in the Netherlands [65].

Australian ST835 strains WA MRSA-40/46 harbour SCC*mec* V_T_ elements and *ccrA/B-2* genes. These strains vary in the presence of β-lactamase genes, the mupirocin and trimethoprim resistance genes *mupA* and *dfrA, qacA* as well as of *sed+sej+ser.* The authors have identified other CC5-MRSA which harbour composite or multiple SCC*mec* elements including [*mecA, ΔmecR1, ugpQ, ccrA-1, ccrB-1, pls-*SCC, *dcs, ccrC*], [*mecA, ΔmecR1, ugpQ, dcs, ccrA-2, ccrB-2, ccrC*] and [*mecA, ugpQ,* “*ccrAA*”, *ccrC, ccrA-4, ccrB-4*]. However, these MRSA strains were only represented by one or two isolates in each case.

### Sequence type 6

According to MLST data, ST6 is a double locus variant (DLV) of ST5 (*arcC*-12 and *yqiL*-3 rather than *arcC*-1 and *yqiL*-10, as in ST5). However, ST6 isolates differ in *agr* group, capsule and *spa* types. They harbour different alleles of *hsdS*-, *set/ssl*- and several MSCRAMM genes (*bbp, fnbB, sdrC, sdrD, clfA, clfB, sasG*). In addition, isolates of this ST carry *cna* but lack *egc.* These observations may be explained by a large scale chromosomal replacement with one parental strain belonging to CC5. The origin of the inserted region has not yet been determined. The size and location of the insert can be estimated by analysing the known positions of the probe sequences within the published CC5 genome sequences. Thus, the insert is localised around *oriC,* and ranges from *hsdS3* downstream of *oriC* to the *ssl/set*-locus upstream of *oriC*. This equals *ca*. 1.500.000 bp (*i.e.,* about half of the genome). It can be assumed that the SCC*mec* element is integrated into that insert. ST6 strains tested for the present study were PVL negative. They included ST6-MRSA-IV from Australia (where it is known as WA MRSA-51) and Abu Dhabi [31] as well as ST6-MRSA-V from Hong Kong.

### Clonal complex 7

CC7-MRSA are rare. One ST, ST1048, is discussed separately due to its divergent hybridisation pattern.

The authors identified single isolates of CC7-MRSA-IV and CC7-MRSA-V in Saxony and in Australia, respectively. The enterotoxin A allele *sea-N315,* also known as *sep*, is present in both isolates (and it is also common in CC7-MSSA). Both isolates are PVL-negative.

### Clonal complex 8

Similar to CC5, CC8 is a pandemic MRSA lineage. Numerous MRSA strains have originated from CC8, including both CA- and HA-MRSA, and several whole genome sequenced MRSA strains represent this lineage (see Supplemental file S2). Although related to CC8, ST72 and ST239/241 are discussed separately as they exhibit distinct hybridisation patterns. The core genome genes of the remaining CC8 strains such as protease, *ssl/set* and MSCRAMM genes of clinical isolates are in accordance with the sequenced genomes although occasional deletions of genes such as *bbp, clfA* or *sdrD* can be observed. Carriage of exotoxins and of β-haemolysin-converting phages is highly variable. Several different CC8 strains have been described, and can be distinguished based on SCC*mec* types and exotoxin profiles.

The first known MRSA was a CC8 strain, ST250-MRSA-I, which is also known as Early or Ancestral MRSA, Irish AR02 or Irish Phenotype I and II. The genome sequence (GenBank CP000046) of the strain COL, which was isolated in England in the 1960s, is representative of this strain [66]. The ST250-MRSA-I clone seems to be disappearing. However, it is still isolated in Australia, although rarely (one isolate out of more than 4000 tested, [67]). The description in this study is based on COL and on isolates recovered from hospitalised patients in Ireland in the 1970s to 1980s [15]. All isolates closely resemble COL, and they also show a characteristic deletion of several *ssl/set* genes. The carriage of *pls-*SCC [15], several resistance markers (see Figure 2), enterotoxin genes *seb+sek+seq* and of β-haemolysin-converting phages (*sak, chp* and *scn*) is variable.

A very similar and, likewise, ancient strain is ST247-MRSA-I. A reference isolate, NARSA 209, was recovered in the UK in 1971. Vernacular names include North German Epidemic Strain, UK-EMRSA-5, -8 and -17, Rome Clone, Spanish PFGE type E1, [34], Irish AR22, Irish New02 and, after an outbreak in Barcelona in 1989, Iberian Clone [68]. ST247-MRSA-I seems to be receding, as observed, for example, in Portugal [49] and Spain [69]. It has not been identified in Dresden, Saxony, since 1997 ([70] and authors' observations) or in Ireland since 1999 [15]. Thus, the description in this study is based partially on isolates recovered from the 1990s. More recently, ST247-MRSA-I has been found in Australia, Croatia [41], the Czech Republic [71], Italy, where it is also becoming increasingly rare [33], and in the Netherlands (isolates courtesy of P. Beisser, Maastricht). Although this strain yields a hybridisation profile similar to that of ST250-MRSA-I, it harbours a complete set of *ssl/set* genes, and with regard to these genes it resembles the NCTC8325 or USA300 genome sequences more than COL. Enterotoxin genes *seb+sek+seq* are rare, but about half of the isolates harbour *sea*. As in other CC8 isolates, *bbp* is deleted in a number of isolates. In addition to SCC*mec* I, all tested isolates carry *tet*(M). Additional resistance markers have been detected in some isolates (Figure 2).

Several different CC8-MRSA-IV strains have been described previously based on different typing methods. In the following paragraphs, we define these strains with reference to their carriage of exotoxin genes. However, as these genes are located on mobile elements such as phages or plasmids, it may be that these “strains” are in fact polyphyletic clusters. One of these strains includes CC8-MRSA-IV isolates which lack PVL or any enterotoxin genes (besides the ubiquitous enterotoxin homologue, GenBank CP000046.1:SACOL1657). They belong to ST8 or ST576 (*tpi-*19 instead of *tpi*-4) and include UK-EMRSA-14 and WA MRSA-5, -6 and -31. NARSA 645 is a reference strain that shows an Iberian Clone-like PFGE pattern (http://www.narsa.net). The authors found only a single isolate from Germany. However, in Australia this strain appears to be rather common. Isolates differ in the carriage of β-haemolysin-converting phages and of a variety of resistance genes.

Another PVL-negative CC8-MRSA-IV has been named the Lyon Clone or UK-EMRSA-2. It is frequently isolated in France [44], and can occasionally be identified in Germany, Ireland [30], the UK, the Netherlands (isolates courtesy of P. Beisser, Maastricht), Norway (courtesy of H. V. Aamont, Aakershus) and Australia. Most isolates of this strain carry *sea, sak* and *scn,* as well as, commonly, *sed+sej+ser.* Some isolates lack the *sea* gene. These isolates harbour *chp, sak* and *scn,* or they have a non-disrupted *hlb* gene and lack all IEC genes. Variable resistance markers are the mercury resistance and β-lactamase operons, *erm*(A), *erm*(C), *vga*(A)*-BM3327, aacA-aphD, aadD, dfrA, far1, tet*(K), *tet*(M), *qacA* and *qacC*. Another related strain has been described as UK-EMRSA-6. It is also *sea-*positive, and differs from Lyon Clone/UK-EMRSA-2 in the presence of *aphA3+sat*.

Another strain of CC8-MRSA-IV carries the enterotoxin genes *seb+sek+seq*, but lacks PVL. It is known as USA500 with NARSA 385 being a reference strain. Isolates have been identified in the USA (NARSA 119, -120, -121, -678, -686 and -708) and Australia (as a SLV, ST612, WA MRSA-20, [72]) as well as, for the present study, in the UK, in Ireland and, sporadically, in Germany. Some of the German isolates were recovered from patients with travel histories (Ethiopia, Zimbabwe and Mozambique) suggesting a wide distribution of this strain in Sub-Saharan Africa. This is also indicated by frequent observations of ST612 in South Africa [73]. USA500 has also been found in horses from the USA, Canada [74], Ireland [30] and Germany [75] as well as from humans with contact to horses [30]. Equine isolates differ from human isolates of USA500 by the absence of lysogenic β-haemolysin converting phages and thus they also lack the *sea, sak, chp* and *scn* genes. The carriage of resistance markers is highly variable (Figure 2).

A notable PVL-positive ST8-MRSA-IV is the widely known strain USA300 (also known as WA MRSA-12, Canadian MRSA-10 [28] or Spanish PFGE type A, [34]). Within a few years it has spread extensively across the USA, effectively marginalising other *S. aureus* strains, MRSA as well as MSSA [76]. It is mainly community-associated, but hospital-associated cases also occur [77]; [78]. In Europe, USA300 has not been isolated frequently. For instance, eight out of 25 PVL-positive CA-MRSA from Ireland were identified as USA300 [57]. Since these isolates originated from a sample of 1,389 MRSA isolates, it can be concluded that USA300 in particular, and PVL-positive MRSA in general, are much less of a problem in Ireland than in the USA [57]. In Dresden/Saxony, the authors identified the first case of a USA300 infection in 2005 [29], but only sporadic isolates have subsequently been observed (including three among 304 genotyped MRSA isolates from patients of the University Hospital Dresden, 2007 to 2009, unpublished observation by the authors). In the UK, USA300 is much less common than in the USA [79]. USA300 has also been infrequently found in Switzerland [80] and Spain [81]. In Abu Dhabi, three out of 54 isolates were identified as USA300 [31]. In Australia, although the overall prevalence is low, case numbers are steadily increasing [82]. Some infections with this strain have also been noted in Japan [61] and Hong Kong (in the present study). Two whole genome sequences of USA300 have been published (USA300-FPR3757, GenBank CP000255, [61] and USA300-TCH1516, GenBank CP000730, [83]). Both sequences harbour SCC*mec* IV and adjacent ACME genes. It has been hypothesised that this locus is related to enhanced survival of the organism on intact skin and, thus, to increased transmissibility by skin contact. However, a considerable proportion of Australian USA300 isolates lack the ACME locus [82]. The absence of ACME has been described in isolates from Colombia, too [84]. Other genes or gene clusters are also subject to a high degree of genetic variability in USA300 and genotyping of 76 West Australian isolates identified 16 different variants of USA300 [82]. However, the most common variant in Germany, Abu Dhabi and Australia is indistinguishable from the sequenced strain USA300-TCH1516. Variability within USA300 can involve β-haemolysin converting phages (*sak, chp, scn*) and enterotoxin genes (*sek, seq*). The authors also observed isolates that yielded USA300-like hybridisation patterns (including ACME), but lacked the SCC*mec* element [82] or even the PVL genes (isolate courtesy of P. Beisser, Maastricht), respectively. Resistance genes in USA300 are highly variable (see Figure 2 and [82]). The recent detection of plasmid-encoded *cfr* in an Irish USA300 isolate [21] is a reason for concern as this gene confers resistance to five classes of antimicrobial drugs including the oxazolidinones (linezolid).

Other CC8-MRSA-IV strains have been found sporadically in Australia. One carries PVL and enterotoxin genes *sed+sej+ser* and *sek+seq*, but lacks ACME (WA MRSA-62). Another harbours *tst1+sec+sel* and, variably, *edinA*.

CC8-MRSA-V has been found occasionally in Saxony and Australia. The Australian CC8-MRSA-V isolates (WA MRSA-53) carry *sea* and *seb+sek+seq*. These genes, however, are absent in the Saxon isolate.

CC8-MRSA-VIII harbouring a class A *mec* complex and *ccrA/B-4* genes has been recently described in Canada [17] where this strain is known as Canadian MRSA-9. It was reported to carry enterotoxin genes *sea* and *seb*. The authors obtained two isolates (one from Ireland [85], and one from Australia, WA MRSA-16) which were similar with regard to SCC*mec* associated genes. However, they lacked the enterotoxin genes.

Several CC8-MRSA carry irregular, multiple or composite SCC*mec* elements. One is ST8-MRSA-II also known as Irish AR05, AR13, AR14, Irish-01 or Irish New03. This strain was predominant in Ireland [86] in the 1990s. However, it now appears to be marginalised by other strains. Thus, the following description is based partially on isolates from the 1990s. It is *spa* type t190. This strain harbours SCC*mec* II, from which the *kdp* operon is absent [15]. Based on this observation as well as on additional variation affecting the *mec* complex and the presence of pUB110, carrying *aadD*, and Tn*554*, carrying *erm*(A), SCC*mec* II subtypes A to E have been described [15]. Thus, the microarray allows rapid SCC*mec* II subtyping with [*aadD+* and *mecI/xylR*+] being SCC*mec* IIA or IIB (depending on presence and localisation of Tn*554*), [*aadD*+ and *mecI/xylR-*] being SCC*mec* IIC, [*aadD*- and *mecI/xylR*+] being SCC*mec* IID and finally, [*aadD*- and *mecI/xylR*-] being SCC*mec* IIE. Additionally, this strain harbours *ccrA/B-4* genes, which are more homologous to those of the SCC-CI element from *S. epidermidis* than to other *ccrA/B-4* sequences from MRSA [87]. The microarray revealed that the mercury resistance operon is usually present, and a possible link to additional *ccrA/B-4* genes, as seen in a SCC-CI element from *S. epidermidis* (ATCC12228), is currently under investigation. Essentially all ST8-MRSA-II, AR13/14 isolates harbour *erm*(A) and *aacA-aphD*. Other resistance markers including *blaZ+blaI+blaR* and *mupA* are variable. The majority of isolates carry a lysogenic β-haemolysin converting phage (*scn, sak,* and *sea*). They do not harbour enterotoxin genes apart from *sea.* Protease genes *splA, splB* and *splE* as well as *sdrC* are usually absent.

Another strain with an irregular or composite SCC*mec* element is ST8-MRSA-IV+*ccrA/B-4*, also known as Irish AR43, Irish-02, UK-EMRSA-12 and -13. This strain predominated in Northern Ireland in 1999 [86] (when most of the isolates described herein were sampled), but it has also been found in Norway (isolates courtesy of H. V. Aamont, Aakershus) as well as by the authors in the UK and Australia. The *spa* type is usually t190 [87]. This strain harbours SCC*mec* IV, although some isolates have been found to lack *ccrA/B-2,* possibly due to *in vitro* passage or exposure to freezing and thawing during storage. Variation in the region downstream of the *mec* complex or, respectively, in the J1 and J3 regions, allowed distinguishing subtypes IVE and IVF [15]. Isolates of Irish AR43 harbour an additional set of *ccrA/B-4* genes similar to Irish AR13/14.

Irregular SCC*mec* elements can also be observed in ST254-MRSA which is known as UK-EMRSA-10 or the Hannover Epidemic strain (*spa* t009 or t036). Although in the 1990s ST254-MRSA was frequently isolated in German hospitals, it has receded since 2000. However, this strain is the predominant MRSA isolated from horses in Germany [75]. There are two variants of this strain which differ in relation to the carriage of SCC*mec* associated genes (see Figure 1). The Hannover Epidemic Strain is a multi-resistant strain, and the antimicrobial resistance genes *tet*(M) and *aacA-aphD* are always present. In addition, *aphA3, sat* as well as the mercury resistance operon can be detected in most isolates. The MSCRAMM gene *bbp* is usually absent. Enterotoxin genes *seb+sek+seq* are always detectable. Genes associated with lysogenic β-haemolysin-converting phages (s*ea, sak* and *scn*) are usually present in isolates from humans, but have not been detected in equine isolates.

### Clonal complex 9

Most CC9-MRSA strains have been recovered from veterinary sources. Particular strains from humans, ST733/834, have been assigned to CC9 by MLST, but will be discussed separately because of their distinct hybridisation pattern. CC9-MRSA with SCC*mec* types III and V have mainly been recovered from pigs or farm workers in mainland China, Hong Kong and Malaysia as well as in Italy [88]-[92]. The authors recently identified isolates of CC9-MRSA-IV in turkeys (courtesy of S. Cortez de Jäckel, Delbrück) and in retail chicken meat from Germany. Isolates are PVL-negative and do not carry other enterotoxin genes besides *egc.* Resistance markers are the β-lactamase operon, *qacC*, *erm*(B) and *aadD*.

### Clonal complex 12

An isolate of CC12-MRSA-IV genotyped for this study was found in Ireland [15]. This strain was also observed in a small number of cases in Norway [93]. Another CC12-MRSA strain, WA MRSA-59 from Australia, has an irregular or truncated SCC*mec* element (*mecA, ugpQ, mecI, mecR1*, but no *xylR* and no detectable recombinase genes). Isolates carried the enterotoxin homologue ORF CM14, *sea-N315* and *seb*. PVL has not been detected in this lineage.

### Clonal complex 15

While CC15-MSSA are abundant among healthy carriers [94], MRSA from this lineage are extremely rare. To our knowledge, CC15-MRSA has only been detected in a collection of Italian MRSA strains isolated in 1980. This included three SCC*mec* I isolates and an isolate described to carry an SCC*mec* I variant in which *ccrA/B-1* was replaced by *ccrA/B-2* [33]. Within the present study, the authors did not identify a single CC15-MRSA isolate among approximately 3,000 genotyped MRSA. Thus, the description of the general features of this CC relies on data from MSSA (Supplemental file S3, [94]; [95]). Exotoxin genes (*lukF/S-PV*, *sea, etA*) are detectable only in a very small minority of isolates. The *chp* and *scn* genes are present, but *sak* was absent from essentially all isolates.

### Clonal complex 20

A single isolate of a CC20-MRSA-V was identified in 2009 in Australia. It is negative for PVL genes. It also lacks other toxin genes, although *seb, sec*, *sel* and *lukM+lukF-P83* genes have been identified by the authors in CC20-MSSA of human and veterinary origin.

### Clonal complex 22

CC22 is a common and widespread clonal group and different MRSA strains have emerged from this genetic background. The sequencing of a complete genome of a CC22 strain could provide valuable insight in *S. aureus* biodiversity since alleles from CC22 appear to differ from previously published sequences. For instance, a CC22-specific allele of *ssl7/set1* has already been identified (GenBank AF188836). Many probes on the DNA array yield irregular or weak signals with CC22 isolates, which could be attributed to the presence of divergent sequences. Beside *ssl/set*- and MSCRAMM genes, this also affects the γ-haemolysin locus. Probes for *lukS/F-hlg* and *hlgA* (derived from CC1, 5 and 8 sequences) yield weak or no signals, while a probe based on a *lukS-hlg* sequence from CC45 is strongly reactive (GenBank EF672356) [96]). Leukocidin *lukD+lukE* and proteases *splA, splB* and *splE* have not been detected in CC22 isolates and it is not yet known whether they may be present as variant alleles or are absent in this lineage.

CC22-MRSA with SCC*mec* types I, II or III have, to our knowledge, not been reported.

ST22-MRSA-IV is a pandemic CC22-MRSA strain. This strain is known as UK-EMRSA-15, Irish AR06, Barnim Epidemic Strain or Spanish PFGE type E13, [34], or Canadian MRSA-8 [28]. It has been reported in many countries [33]; [57]; [97]-[104]. Where ST22-MRSA-IV occurs, it tends to be abundant. In Dresden, ST22-MRSA-IV accounted for nearly 50% (141 out of 304 genotyped isolates from 2007 to 2009); in Portugal for 54% [49], in Malta for 66% [40], and in Ireland [85]; [105] as well as on the Azores [59] for more than 80% of MRSA isolates. In England, ST22-MRSA-IV is increasingly common (apparently at the expense of ST36-MRSA-II, UK-MRSA-16, [106]), being currently responsible for 85% of MRSA bacteraemia cases. ST22-MRSA-IV occurs in hospitals as well as in outpatients, and it has been recovered from animals such as horses [75], cats [107] and dogs [102]; [107]. Common resistance markers are β-lactamase and *erm*(C). A high percentage of Maltese ST22-MRSA-IV isolates harbour Q6GD50 which has not been found in isolates from other geographic regions [40]. Variable virulence markers in ST22-MRSA-IV are *sec* and *sel* as well as the IEC genes encoded by lysogenic β-haemolysin-converting phages (*sak, chp, scn*). The presence of *tst1,* or sometimes of *tst1* and *sea*, is found in some isolates from Abu Dhabi [31], Egypt (isolates courtesy of M. Kamal El Din, Cairo) and England (where patients' names suggest a Middle Eastern origin). A case of an infection with a *tst1-*positive CC22-MRSA-IV has recently been reported from the USA, where it was one of the first reports of this strain [108].

Another, distinct ST22-MRSA-IV strain is found in Dublin, Ireland [109]. Its most distinguishing feature is the presence of the ACME-locus. It carries β-lactamase and *erm*(C) as well as, in the majority of cases, *lnu*(A), *aacA-aphD, aadD* and *mupA.*

A large scale nosocomial outbreak of PVL-positive ST22-MRSA-IV has been described in Bavaria, Germany [110]. Other isolates have also been found in Australia, England [111], Ireland [57] Abu Dhabi [31] and Hong Kong. Additionally, PVL-positive ST22-MRSA-IV has been isolated from patients in Germany who had family ties to Turkey [112]. Their presence in epidemiologically unrelated settings suggested a polyphyletic origin, given that PVL-positive CC22-MSSA are common and widespread [113]. Indeed, the demonstration of at least three different PVL-encoding phages, ϕPVL, ϕ108PVL and an unidentified icosahedral phage, in CC22-MRSA from England and Wales [40] indicates that this “strain” has evolved on multiple occasions. Furthermore, ST22-MRSA-IV harbouring PVL and *tst1* genes have been recently described in India [114]. Variable resistance markers in PVL-positive ST22-MRSA-IV include β-lactamase, *erm*(C), *aacA-aphD, aadD, dfrA* and Q6GD50.

Sporadic cases of a PVL-negative CC22-MRSA-V have been identified by the authors in Saxony, as well as a PVL-positive CC22-MRSA-V in Western Australia.

### Clonal complex 30

CC30 is another important clonal complex from which HA- and CA-MRSA originate. It is represented by the genome sequence of Sanger MRSA 252. Core genomic markers, *ssl/set*- and MSCRAMM genes are present as allelic variants which differ distinctly from other CCs. Some of these variants resemble alleles found in other CCs such as CC22 and CC45 [96] which may be more closely related to CC30 than to other CCs such as CC1, CC5 or CC8 [115]. Shared features of CC30 strains include the presence of *egc* (although partial deletions may occur) and *cna*. MSCRAMM genes *bbp, fnbB* and *sdrD* are usually detectable, but may be deleted from individual isolates. Lysogenic β-haemolysin-converting phages are usually commonly present. Because of the variable presence of *sea* and *chp,* it can be assumed that different phages have integrated into CC30 genomes.

CC30-MRSA-I was reported in Italy in 1980 [33].

A more widespread HA-MRSA strain from this lineage is ST36/39-MRSA-II, also known as UK-EMRSA-16 [116], USA200, Irish AR7.0/AR07.2, Spanish PFGE type E12, [34], or Canadian MRSA-4 [28]. Although frequently isolated in the UK and Ireland in the 1990s [15], recently it has become increasingly rare [107]. Isolates have also been found in Malta [40]; [117] and South Africa [73]. In Germany, ST36-MRSA-II is very rare. This strain harbours SCC*mec* II including *aadD* and *erm*(A) in integrated p*UB110* and Tn*554*, respectively, as well as the β-lactamase operon and occasional other resistance markers (Figure 2). Most clinical isolates carry *tst1,* although this gene is absent in the genome sequence of Sanger MRSA252. This strain also lacks *sdrD*. The enterotoxin gene *sea* is also common, although another reference strain, ATCC43300, is *sea*-negative. ATCC43300 is unique among the tested ST36/39-MRSA-II in being positive for *sec+sel* and *fnbB*.

Another important CC30-MRSA strain is the PVL-positive ST30-MRSA-IV, Southwest Pacific Clone, USA1100 or West Samoan Phage Pattern (WSPP) Clone. This CA-MRSA strain was first observed among Samoan immigrants in New Zealand, but is widespread with isolates investigated in the present study coming from Germany, Switzerland, the UK, Australia [29]; [80], Hong Kong, Taiwan [50], Abu Dhabi, and the USA (NARSA 484). Other reports of this strain include Ireland [57], where it is the predominant PVL-positive MRSA strain, Scandinavia, Latvia [118] and Kuwait [119]. The WSPP Clone apparently evolved from the pandemic phage type 80/81 strain. This MSSA emerged in the 1950s and caused outbreaks of severe infections worldwide; however it virtually disappeared after the introduction of penicillinase-resistant β-lactams [120].

A PVL-negative, but *tst1*-positive strain of ST30-MRSA-IV is sporadically isolated in Ireland [15] and Australia. An infection with a PVL-positive CC30-MRSA-V has been described in Egypt [121]. A similar isolate has been identified from a patient with a SSTI living in the German/Polish border region (courtesy of R. Hillert, Görlitz).

### Clonal complex 45

CC45 is another major lineage from which several MRSA strains have emerged. They cluster into two distinct groups as the majority of isolates belong to *agr* group I, while some strains yield a unique pattern. They fail to react with all three *agrD*-I probes, but they are positive with *agrB* and *agrC* probes corresponding to *agr* groups I and IV (for the sake of simplicity they are here referred to as CC45/*agr* IV). PVL has not been detected in the present study among CC45-MRSA isolates. However, two cases of PVL-positive ST45-MRSA-IV from Belgium [45] and Germany [122] have been reported. Leukocidin genes *lukD+lukE* were not identified. In contrast to CC22, the γ-haemolysin genes *hlgA* and *lukF-hlg* can be detected in CC45 isolates, and *lukS-hlg* was shown to be present in a specific allelic variant (GenBank EF672356, [96]). As observed in CC22, probes for protease genes do not yield signals. Enterotoxins *sec* and *sel* are occasionally found. Irregular signals for *ssl/set* might indicate the presence of yet unknown alleles. The gene *cna* is present, but *sasG* can only be detected in the CC45/*agr* IV isolates.

ST45-MRSA-II, (known as USA600, USA600-MRSA-II or Canadian MRSA-1 [28]) appears to be largely restricted to North America, where glycopeptide-resistant variants and an unusually high mortality rate of bloodstream infections associated with this strain have been reported [123]. Outside the USA, ST45-MRSA-II is rarely isolated, but this strain has, like ST45-MRSA-I and -III, been sporadically detected in Hong Kong [47]. In addition, the authors have identified a single isolate of ST45-MRSA-II in Australia. This isolate as well as reference strains NARSA 22, NARSA 648 and NARSA 715 were genotyped. They carry SCC*mec* II, including *aadD* and *erm*(A), as well as the β-lactamase operon. Variable resistance markers (positive in NARSA 22) include *aacA-aphD, dfrA* and *qacC.*

Another important CC45 strain is ST45-MRSA-IV, which is known as the Berlin Epidemic Strain, WA MRSA-75 or USA600-MRSA-IV. In Saxony, it is a relatively common MRSA strain (*ca.* 9% of MRSA isolates from Dresden, 2007-2009), and in Belgium it is the predominant MRSA strain [45]. This strain also occurs in the UK, the Netherlands [45], Switzerland [124], Croatia [41] and Australia. Unlike the Australian isolates, most of the Saxon isolates harbour *aphA3* and *sat.* As in other CC45 strains, *egc* is present but *seg* is not detectable in many isolates. Enterotoxin genes *sec* and *sel* as well as genes *sak, chp* and *scn*, indicative of lysogenic β-haemolysin-converting phages, are commonly detected.

A similar CC45-MRSA-IV strain isolated in Australia carries *sec, sel, tst1* and ACME.

ST45-MRSA-V has been found sporadically in Germany and in Australia (WA MRSA-4) as well as in Portugal [49]. Most isolates tested for this study harbour *tst1, sek* and *seq.*

A distinct group of CC45 isolates display *agr* IV alleles. Furthermore, these strains differ from other CC45 strains in that they carry other alleles of MSCRAMM genes *fnbA, fnbB, sdrD, vwb* and of *lmrP*. They are positive for *sasG*. Isolates carry *sej* and usually yield weak signals for *ser* probes, which may indicate the presence of an allelic variant of this gene. The gene *sed*, which is normally located on the same plasmids as *sej* and *ser* (*e.g*., pIB485, GenBank M94872.1) cannot be detected. The *spa* types associated with CC45/*agr* IV isolates are t727, t1081 (although this type can also be observed in ST1048 and ST1774, see below and [125]) or t1575. CC45/*agr* IV MRSA are widespread and increasingly common in Australia. There are two different strains carrying SCC*mec* type IV (WA MRSA-23) or V elements (WA MRSA-84), respectively. CC45/*agr* IV-MRSA-IV and -V are commonly isolated in Hong Kong (author's observations, [47]; [125]) and few isolates of a CC45/*agr* IV-MRSA with a combined SCC*mec* IV/V element have been identified as part of this study in Hong Kong. One of these isolates is ACME-positive. Sporadic isolates of CC45/*agr* IV-MRSA-IV have also been observed in Ireland [15].

### Clonal complex 59

Several strains of CC59-MRSA can be distinguished based on carriage of SCC*mec* elements and PVL as well as on MLST and *spa* typing. West Australian strains of CC59 have recently been described in detail [126].

One PVL-positive strain, ST59-MRSA-IV, also known as USA1000, is mainly restricted to the USA (reference strains NARSA 483, NARSA 676). The authors identified a single isolate of USA1000 from Australia. The *spa* types associated with this strain are t216 or t316. USA1000 carries the β-lactamase operon and, variably, *erm*(A). Beside PVL, it harbours *chp, scn* and, variably, the enterotoxin genes *seb, sek* and *seq*. A sporadic Australian strain (WA MRSA-73, *spa* t528) is PVL-negative, but otherwise indistinguishable from USA1000 in terms of overall hybridisation patterns.

WA MRSA-24 is an infrequently isolated, mainly Australian CC59 strain with a SCC*mec* IV element. This strain belongs to ST87 (a SLV of ST59) and *spa* type t216. The β-lactamase operon, *msr*(A), *mph*(C), *aphA3*+*sat, seb, sek* and *seq* are present. This strain is also positive for *sak*, *chp* and *scn* but lacks *sea*. PVL is absent.

A group of sporadic Australian, Hong Kong and UK [127] isolates, dubbed WA MRSA-55/56, are ST59-MRSA-IV/*spa* type t437. These isolates carry PVL (although one PVL-negative isolate was also identified), *seb, sek* and *seq.* The gene *sea* is only detected occasionally.

The most widespread CC59-MRSA strain is PVL-positive ST59/952-MRSA-V_T_ which is known as the Taiwan Clone. Isolates of this strain belong to *spa* types t437, t1950 or t2365. ST59/952-MRSA-V_T_ is a common and clinically important MRSA strain in Taiwan [14]; [128]-[131]. The authors found this strain in Hong Kong, Australia, where it is the most frequently isolated CC59-MRSA strain, Saxony (a single case of recurrent furunculosis, [29]) and the UK [127]. This strain has a characteristic variant of a SCC*mec* V element, in which two *ccrC* genes are present (SCC*mec* V_T_ or 5C2&5, GenBank AB12129, [132]). Enterotoxin genes *seb+sek*+*seq* are usually detectable. All isolates carry *erm*(B), *aphA3+sat* and *blaZ+blaI+blaR*. In addition, *tet*(K) and *cat* can often be found. This strain is indistinguishable from WA MRSA-55/56 in all markers covered by the microarray with the sole exception of the SCC*mec* element. Therefore, it may be assumed that both strains emerged from the same ancestral MSSA acquiring different SCC*mec* elements.

Additionally, there are also ST59-MRSA isolates harbouring a SCC*mec* V rather than a SCC*mec* V_T_ element. PVL-negative ST59-MRSA-V (WA MRSA-9) has occasionally been observed in Australia [126] as well as PVL-positive CC59/ST359-MRSA-V in the UK [80].

WA MRSA-15 is another ST59 strain with a composite or novel SCC*mec* element. This is the second most common CC59-MRSA strain in Australia [126]. Its *spa* type is t976. Microarray hybridisation and PCR results suggest either the presence of a composite IV and V SCC*mec* element, or the presence of *ccrC* in addition to SCC*mec* IV. This strain is PVL-negative, but it harbours *sea* and usually *seb+sek*+*seq.* The β-lactamase operon is present while *msr*(A)*+mph*(C), *aphA3+sat* and *tet*(K) can be detected sporadically.

### Sequence type 72

According to the MLST database, ST72 belongs to CC8. However, isolates yield a distinctive hybridisation profile. Differences to CC8 include the presence of *egc* and of CC5-like alleles of several MSCRAMM genes (*bbp, sdrC, sdrD*). MLST suggests a recombination of CC5 and CC8. Specifically, *yqiL*, *pta* and *tpi* may be derived from CC8 (*yqiL*-3, *pta*-4, *tpi-*4), while *gmk*, *aroE* and *arcC* suggest CC5 parentage (*gmk*-8, *aroE*-4, *arcC*-1). The *glpF* allele (*glpF*-1) may have originated from either clonal complex. Mapping the positions of probe binding sites over the known CC5 and CC8 genomes shows that CC5- and CC8-derived genes alternate through the genome suggesting multiple recombination events. Alleles of some *ssl/set* genes and *vwB, sasG* and *clfB* which are not associated with CC5 or CC8 suggest the involvement of additional, yet unidentified donor strains.

Two MRSA strains have been identified. One is a PVL-negative ST72-MRSA-IV which is known as USA700. It does not harbour exotoxins beside *egc*, and its carriage of resistance genes is variable (Figure 2). This strain includes NARSA 386 and 689. Isolates have been identified in Saxony and in Abu Dhabi. PVL-positive ST72-MRSA-IV has been isolated by the authors in Australia (WA MRSA-44) and from SSTIs of German tourists returning from Costa Rica.

### Clonal complex 75, sequence types 883 and 1303

CC75 (ST75, ST1304) as well as ST883 and ST1303 are discussed as a group because they share several distinctive features. ST75-MRSA-IV (WA MRSA-8 and -79) isolates have been recovered from people residing in remote communities of Northern and Western Australia, and appear to be largely restricted to this part of the world. Although it is PVL-negative, this strain is a common cause of community acquired SSTIs [133]. There has been a single report of ST75-MRSA-V [133]. One isolate of ST1304-MRSA-IV (WA MRSA-72) from Australia was characterised. This is a SLV of ST75-MRSA-IV that also differs in carriage of possibly plasmid-borne enterotoxin genes (see below). ST883-MRSA-IV (WA MRSA-47) and ST1303-MRSA-IV occur sporadically in the Northern part of Western Australia.

**Figure 4:**
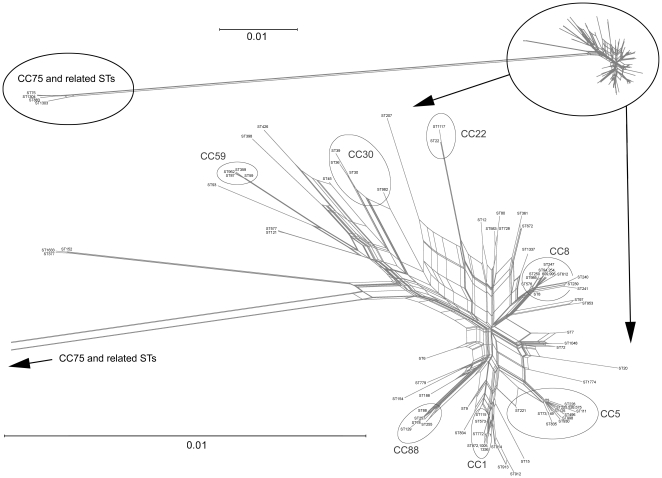
Network graph visualising relationships between concatenated MLST sequences of all STs mentioned in this study.

Recently it has been shown that MLST genes of these strains differ grossly from that of any other known *S. aureus* (see [134] and Figure 4). Although not phenotypically different to other *S. aureus*, it has been suggested that ST75 strains should be regarded as a new subspecies of *S. aureus* [134]. These strains do not yield hybridisation signals with specific probes for capsule types 1, 5 or 8 and for *agr* groups I to IV. Unique *agrB, agrD* and *agrC* sequences have been demonstrated in ST75 (GenBank FJ154839, [135]; [136]) and ST883 (GenBank HQ260328, [136]). Genes encoding leukocidins and exfoliative toxins as well as protease genes *splA, splB* and *splE* cannot be detected by microarray hybridisation. Probes for MSCRAMM-, *ssl/set*- and *hsdS*-genes generally yield patterns which differ from all other *S. aureus* strains. With regard to these genes, ST883 and ST1303 resemble each other but differ from CC75. The gene *cna* is absent and *sasG* can be detected in CC75 only. The β-haemolysin gene *hlb* can only be detected when using random primer directed amplification, suggesting the presence of an allelic variant. CC75 and ST1303 carry *egc* (although *sen* is either absent, or present in an unknown allele). Seven out of eighteen ST75-MRSA-IV isolates as well as the single ST1303-MRSA-IV isolate tested carry *seb*. ST1304-MRSA-IV is positive for *sed+sej+ser.*

### Clonal complex 80

The vast majority of CC80 isolates belong to a PVL-positive strain harbouring SCC*mec* IV. Although SCC*mec* type I has previously been described in CC80 [41], the authors did not identify this strain in this study. Likewise, PVL-negative variants of CC80-MRSA-IV are infrequently isolated and have only been found in France and Croatia [41].

PVL-positive CC80-MRSA-IV has been dubbed the European CA-MRSA Clone. The strain is widespread and has been isolated in Austria [137], Denmark (where this strain was detected as early as 1993 [138]), France [44], Germany [80]; [139], Greece (isolates courtesy of V. Gogou, Larissa, [48]; [140]), Ireland [57], Malta [40], the Netherlands [141], Norway [93], Portugal [142], Sweden [143], Switzerland [80] and the UK [80]; [144]; [145]. In Greece, a considerable percentage of MRSA infections can be attributed to this strain [140]. The authors have identified isolates in Abu Dhabi, and other studies also indicate a wide distribution in the Middle East including Kuwait [119], Lebanon [146], Israel [39], Egypt [147] Algeria [148] and Tunisia [149]. Travellers returning from the Dead Sea or from Saudi Arabia to Germany [112], or from Tunisia or Libya to Switzerland [62] as well as patients with family ties to Turkey [112] have been found to carry this strain. In Australia, ST80-MRSA-IV as well as two SLVs, ST583 (WA MRSA-17) and ST728 (WA MRSA-30) are rarely isolated. CC80-MRSA-IV carries PVL, *etD* and *edinC*, but lack enterotoxin genes (beside an ubiquitous homologue, GenBank CP000046.1:SACOL1657). Nearly all CC80-MRSA-IV isolates carry *aphA3* and *sat*; and they harbour the plasmid encoded genes *blaZ, tet*(K) and *far1*. Additional resistance genes *lnu*(A) or *erm*(C) are rarely detected. Mupirocin resistance in this strain has been reported previously [150].

### Clonal complex 88

Several different CA-MRSA belong to this CC which appears, based on *spa* sequences and hybridisation profile, closely related to CC1 and CC80. It includes ST78-MRSA-IV (as well as its SLVs ST129, ST255, ST257), which is known in Australia as WA MRSA-2. This strain is PVL-negative, but usually harbours enterotoxin genes *sec* and *sel*. In addition, the vast majority of isolates carry *blaZ* and *erm*(A). It is frequently isolated in Australia, and a single isolate has been identified, as part of this study, in a patient from Saxony.

CC88-MRSA-IV carrying the exfoliative toxin gene *etA* were identified by the authors in the Netherlands, Portugal, Angola and Senegal. There is also a report from Japan [151]. PVL-positive CC88-MRSA-IV has been identified by the authors in the UK [80], Abu Dhabi and Australia. Other reports of this strain have come from Spain [152] and Nigeria [153].

PVL-positive CC88-MRSA-V and PVL-negative CC88-MRSA-VI have been identified sporadically by the authors in Western Australia. PVL-positive CC88-MRSA-V were recently described from Italy [33]; [ 154].

### Sequence type 93

ST93 is a unique ST of *S. aureus*, which is essentially restricted to Australia. While PVL-negative ST93-MRSA-IV and PVL-positive ST93-MRSA-V_T_ are extremely rare, a PVL-positive ST93-MRSA-IV is common and currently spreading across Australia [155]. This CA-MRSA strain is also known as the Queensland Clone [156]. A few cases have been identified in the UK suggesting small scale importation due to travel activities [157]. Recently, a whole genome sequence, JKD6159 (GenBank CP002114), has been released. In terms of hybridisation profiles and especially with regard to *ssl/set* genes, ST93 differs markedly from other *S. aureus* lineages [80]. Enterotoxin genes and *tst1* are absent but the enterotoxin homologue ORF CM14 is present.

### Clonal complex 97

CC97-MSSA can often be isolated from cattle [158] and occasionally from humans [95], but MRSA from this lineage are rare. CC97-MRSA-IV has sporadically been isolated from human patients in Australia (WA MRSA-54), Abu Dhabi [31] and, as part of this study, in Saxony. A ST97-MRSA-V has been isolated in Egypt (isolate courtesy of M. Kamal El Din, Cairo). A further CC97-MRSA strain has recently been described from the UK [159]; [160], in which a SCC*mec* V element, ACME and *ccrA/B-4* genes are detectable. A CC97-MRSA strain was identified by the authors in swine from Germany. The strain carries a novel or composite/hybrid SCC*mec* element yielding signals with probes for *mecA, ugpQ, ccrA-1, ccrB-1,* “*ccrAA*” and *ccrC*. All CC97-MRSA isolates tested are PVL-negative.

### Clonal complex 121

Although CC121-MSSA are a common cause of SSTI worldwide [113]; [142]; [153]; [161]; [162], MRSA from this lineage appear to be very rare. The authors found a single MRSA isolate, CC121/ST577-MRSA-V (WA MRSA-22), in Australia. While PVL is common in CC121-MSSA [113], this isolate is PVL-negative. It harbours *etA* and *edinA*. Further CC121-MRSA-IV have been described from sporadic cases in the UK [145], Portugal [163], Poland (http://www.isssi2008.com/abstract/13.asp), China (see MLST database, quoted in [163]) and the USA [164]. A PVL-positive CC121-MRSA-V strain was identified recently in two unrelated paediatric patients from Cambodia [165].

### Clonal complex 152

CC152-MRSA-V has been found sporadically in Germany [80], Sweden [166], Switzerland [62] and Australia (WA MRSA-89). Some patients infected with this strain had ties to Balkan countries (Macedonia, Kosovo [62]; [80]) which might indicate a wider distribution in that region. All isolates of this strain carry PVL genes and *edinB*. Probes for *lukD+lukE* as well as *hlgA+lukS-hlg+lukF-hlg* yield signals only after random amplification, which suggests the presence of hitherto unknown alleles. Enterotoxin genes and most *ssl/set* genes are not detectable.

ST377-MRSA-V has been observed in France, the Netherlands, Switzerland and Australia [167]. It is closely related to ST152 and cannot be distinguished using standard MLST primers. Specific *gmk* primers able to distinguish ST152 from ST377 have been described by Garnier [167]. A ST152-MRSA with a non-typeable SCC*mec* element has been isolated in Denmark from a patient with a travel history to Kosovo [138].

### Sequence type 154

Two ST154-MRSA isolates have been characterised in this study, both cultured from central Asian immigrants to Western Europe [57]; [166]. The origin of these two isolates as well as a report from Mongolia [168] suggests a wide distribution of this strain in Central Asia. One isolate harbours a typical SCC*mec* IV element, while the other (courtesy of C. Berglund, Stockholm) lacks *ccrA-2* and *ccrB-2*. Both isolates are PVL-positive.

### Clonal complex 188

According to MLST data, CC188 is related to CC1 although it differs in two alleles (*arcC-*3 and *gmk*-8 instead of *arcC-*1 and *gmk*-1 [169]). However, CC188 hybridisation profiles are clearly distinct. Differences include the alleles of the *agr* locus, *clfA, clfB, ebh, ebpS, fnbA, fnbB, hsdS, sdrC, sdrD* and *vwb*, the presence of *cna* as well as the absence of *sasG, seh, splA* and *Q2FXC0*. Since these genes are distributed widely across the genome, the differences to CC1 strains cannot readily be explained by chromosomal replacement as previously observed for ST34 and ST239 [170]. Therefore, either the positions of the genes in the genome may be different than in the sequenced genomes, or CC188 originates from multiple recombination events.

ST188-MRSA-IV has been found sporadically in Australia (WA MRSA-38 or -78, [72]). Apart from SCC*mec* IV, β-lactamase and *aacA-aphD*, this strain may carry *erm*(B), *tet*(K) and *cat.* PVL, enterotoxin genes and *tst1* are not present. Other ST188-MRSA have been observed from Asian countries: ST188-MRSA-III/*spa* t189 in Korea [171], PVL-negative CC188-MRSA-V in Hong Kong, and PVL-positive ST188-MRSA-V in Malaysia [172].

### Sequence type 239

ST239 isolates belong to CC8. However, ST239 (including ST240 and ST241, which differ only in mutations in *pta* or *yqiL* genes, respectively) is discussed separately from CC8. The reason is the integration of a CC30 DNA fragment of approximately 635,000 base pairs (or *ca.* 20% of the genome, [170]; [173]) into a CC8 parent strain, with the integration site being localised around *oriC*. This has led to divergent MLST profiles (*arcC*-2, rather than *arcC*-3, [170]; [173]), *spa* types and hybridisation profiles. Differences to other CC8 strains include the affiliation to capsule type 5, the alleles of *aur, clfB* and *isaB* and the presence of *cna,* while other markers (such as *agr* group I alleles, *ssl/set* genes *etc.*) are in accordance to CC8. Two genome sequences of ST239-MRSA-III have recently been released (strain TW20, [173] and JKD6008, [174]).

ST239-MRSA-III is probably the oldest pandemic MRSA strain. It has been reported in many European countries including Croatia [41] the Czech Republic [71]; [175], Greece (isolates courtesy of V. Gogou, Larissa), Italy [33], Malta [40], Portugal [49]; [176], Spain [69] and the UK. In Hungary, it was common but it has been largely replaced by ST228-MRSA-I [35]. In Ireland, ST239-MRSA-III became the predominant strain in the 1980s [15], after it was introduced into the country following the repatriation from Iraq of a trauma patient [177]. In Saxony, this strain is rarely isolated and has been identified by the authors in patients with travel histories to Greece [29] or Turkey. This strain is frequently isolated in Turkey [178], Iran [179], Saudi Arabia [180], Hong Kong [47], mainland China [181]; [182], Taiwan [14]; [181] and Singapore [183]. In Trinidad & Tobago [184] it is virtually the only existent MRSA. In Australia, it is a common cause of hospital-acquired infection in the East coast states, and large outbreaks in the 1980s were attributable to this strain [155]. Furthermore, ST239-MRSA-III has also been reported in Argentina [185], Brazil [186]; [187], Chile [185], Egypt (isolates courtesy of M. Kamal El Din, Cairo), India [104], Korea [171], Malaysia [172], Mongolia [168], New Zealand [174], Pakistan [188], Paraguay [42], Russia [189], South Africa [73], Thailand [176] and Uruguay [185]. The reference strain ATCC33592, recovered in a hospital in New York [190], belongs also to ST239-MRSA-III.

The evolution of this strain has recently been reviewed based on genome sequences of 63 isolates from different parts of the world analysing genome-wide single-nucleotide polymorphisms, insertions or deletions [191]. In short, ST239-MRSA-III can be divided into three clades; a European, presumably ancestral one, an Asian and a South-American. However, there is some evidence for secondary, travel-associated cross-transmission [191].

ST239-MRSA-III is colloquially known as the Czech, Vienna, Hungarian, Portuguese or Brazilian Clone, UK-EMRSA-1, -4, -7, -9 or -11, AUS-EMRSA-2 or -3, Irish Phenotype III, Irish AR01, -09, -15 or -23, Canadian MRSA-3 (this refers to the ST241 SLV, [28]) or as Canadian MRSA-6 [28]. However, all these designations should be regarded as synonyms as their distinction is not always clear-cut. For instance, the Hungarian Clone was described as harbouring SCC*mec* III, while the Brazilian Clone carries SCC*mec* IIIA [192]. The difference is only the presence (III) or absence (IIIA) of an integrated plasmid p*T181*, which encompasses, among other genes, *tet*(K). Since the SCC*mec*-associated mercury resistance operon and other resistance and enterotoxin genes vary independently of p*T181*/*tet*(K) among these clones and since *tet*(K) might also be part of free plasmids, *tet*(K) could be regarded just as one mobile element among many others. Beyond the issue of *tet*(K), there are also other variations of the SCC*mec* III element. About two thirds of tested isolates harbour the mercury resistance operon. It is often, but not always, accompanied by “*ccrAA*”*+ccrC.* This indicates a linkage of SCC*mec* III to SCC*mer* [4]. Some isolates do not yield signals with a probe which normally reacts in both, *mecR1* and *ΔmecR1*; this could probably be attributed to another partial deletion of the *mecR1* gene. In one case, the authors found the absence of *mecI, mecR1* and *xylR.* Variable resistance genes in ST239-MRSA-III include *erm*(A), *erm*(C), *aacA-aphD, aadD, aphA3, sat, dfrA, mupA, tet*(K), different alleles of *cat*, *qacA* and *qacC.* PVL has not been detected in ST239-MRSA-III. Enterotoxin genes *sea, sek* and *seq* as well as phage-associated genes *sak, scn* and *chp* are variable. The ACME locus is present in a minority of isolates ([172], and in three out of some 200 tested by the authors).

### Clonal complex 361

The authors observed three different CC361-MRSA strains. All are PVL-negative. CC361-MRSA-IV is detected sporadically in Western Australia (ST672-MRSA-IV or WA MRSA-29, [72]) and one isolate originated from Ireland. CC361-MRSA-V was isolated in Abu Dhabi where it appears to be rare [31]. A CC361-MRSA-VIII was found in Australia where it is referred to as WA MRSA-28 [72]. Isolates harbour the enterotoxin gene locus *egc,* which, however, might be partially deleted. One out of three tested WA MRSA-29 isolates carries *tst1* and *seb*, which is a highly unusual combination of virulence genes [193].

### Clonal complex 398

CC398-MRSA has recently received a lot of attention as strains from this lineage are of animal origin but are able to cause disease in humans. This has led to intense investigations including the sequencing of a complete genome of a ST398-MRSA-V strain, S0385, from the Netherlands [194].

ST398-MRSA-III, *spa* type t567, has been observed in Belgium [13]. ST398-MRSA-IV isolates appear to be a rare livestock-associated MRSA (LA-MRSA) strain. Two infections of humans were observed in Hong-Kong [47] and isolates from poultry have been reported from Belgium [13]. In Germany, this strain has been detected in cattle [195] and in turkeys (courtesy of S. Cortez de Jäckel, Delbrück), and by the authors in turkey meat samples. Isolates are negative for exotoxin genes and for *sak, scn* and *chp.*

PVL-negative ST398-MRSA-V is frequently found in association with livestock, although this strain is also increasingly isolated from human patients without animal contact. It was first discovered in a family outbreak in the Netherlands in 2006. Family members were farmers, and the strain was subsequently found in pigs from the same farm. Further investigations showed its presence in a high proportion of Dutch pigs as well as in farm personnel, veterinarians and students [196]-[200]. Recently, this strain has been observed not only in pigs, but in humans, cattle [158]; [195], horses [75], dogs [201], poultry [13], chickens and turkeys (courtesy of S. Cortez de Jäckel, Delbrück and of the Federal Institute for Risk Assessment, Berlin). It was also found in retail meat of different domestic animals [202]; [203]. In addition to the Netherlands, ST398-MRSA-V has been identified in Germany, Belgium, Italy, Austria, Spain [13]; [90]; [202]; [204]-[208] Canada (http://promedmail.oracle.com/pls/otn/f?p=2400:1001:8447806422203976::NO::F2400_P1001_BACK_PAGE,F2400_P1001_PUB_MAIL_ID:1010,81749), the USA [209] and Australia (author's unpublished observation). This strain shows a remarkable diversity with regard to resistance genes. Isolates carry SCC*mec* V, or rather SCC*mec* V_T_, as indicated by the genome sequence of strain S0385 [194]. Rarely, recombinase genes “*ccrAA*” and *ccrC* may be absent. Essentially all isolates harbour the β-lactamase operon and *tet*(M). Additionally, *tet*(K) is frequently detected. The multidrug resistance gene *cfr* has recently been observed in ST398-MRSA-V [19]. In addition to the resistance markers detected by array hybridisation (Figure 2), *tet*(L), *dfrK* and *dfrG* can be found by PCR in some isolates [210]–[212]. Recently, the novel trimethoprim resistance gene *dfrK* [210], the macrolide-lincosamide-streptogramin B resistance gene *erm*(T) [212], a ABC transporter gene *vga*(C) for streptgramin A-lincosamide-pleuromutilin resistance ([213]) and the novel apramycin resistance gene *apmA* [214] have been described on plasmids from this strain. Many isolates of ST398-MRSA-V harbour multiple resistance genes which provide the same resistance phenotypes (*tet*(K)*+tet*(M), *tet*(L)*+tet*(M) or *tet*(K)*+tet*(L)*+tet*(M); *erm*(A)*+erm*(C) or *erm*(A)*+erm*(B)). The vast majority of ST398-MRSA-V isolates are negative for enterotoxin genes. Among 54 swine isolates from Germany, *seb* was detected only once, and *sek+seq* were detected in three isolates [215]. The genes *sak, scn* and *chp* were absent from isolates of the European LA-MRSA strain. However, these genes where detected by the authors in two human isolates from Hong Kong; one of which harbours *sea*.

PVL-positive ST398-MRSA-V has been described in China [216], and in children of Asian origin living in Sweden [217]. Two isolates (courtesy of C. Welinder-Olsson, Gothenburg, Sweden) have been genotyped. In contrast to the livestock-associated ST398-MRSA-V isolates, they carry *sak, scn* and *chp.* One isolate harbours also *sea*.

### Sequence type 426

ST426-MRSA-IV has recently been described from the Arkhangelsk region in Russia [162]. The authors did not isolate ST426-MRSA within the present study, but genotyping data of ST426-MSSA isolates [94]; [95]; [113] allow the general features of that sequence type to be described. ST426-MSSA isolates harbour *tst1, sea* and ORF CM14 as well as, variably *sec+sel, see* and/or *sek+seq*. The gene *cna* can be detected in most isolates. Notably, *nuc* (encoding thermostable nuclease, DNAse) is not detectable by hybridisation. Since ST426 isolates are phenotypically DNAse-positive, the presence of a variant *nuc* allele can be assumed.

### Clonal complex 509

CC509 appears to be a very rare CC [94]; [218], but MRSA belonging to it have previously been observed in Queensland, Australia [218]. One isolate of CC509/ST207-MRSA-V from New South Wales, Australia was genotyped. It is PVL-negative. The *egc* locus appears to be present in a variant or truncated form with only genes *sem* and *seo* being detectable.

### Sequence types 573 and 772

Sequence types 573 and 772 belong, according to the MLST database, to CC1. However, with respect to the *pta* allele these strains differ from other CC1 (*pta*-12 or *pta*-22, respectively). Their hybridisation profile is also distinct, which may be attributed to one or multiple recombination events that have introduced genes from other CCs into CC1. This includes *agr* (group II rather than III), genes encoding the capsule type (5 rather than 8), the *egc* enterotoxin gene cluster, and the enterotoxin homologue ORF CM14. The enterotoxin H gene *seh*, which is otherwise typical for CC1 strains, cannot be detected in ST573/772. The genes *cna* and *sasG* are present. Protease genes *splA, splB* and *splE* are absent.

A PVL-negative ST573-MRSA-V strain has been described from Australia (WA MRSA-10, [72]).

PVL-positive ST772-MRSA-V, which is known as WA MRSA-60 or the Bengal Bay Clone [111], is relatively multi-resistant compared to other CA-MRSA. In addition to a SCC*mec* V (specifically, a V_T_ [127]) element, they carry variably *blaZ*, *erm*(C), *msr*(A), *mph*(C), *aacA-aphD, aphA3, sat* and *tet*(K). Besides the PVL genes, all isolates tested harbour *sea*, and most carry *sec* and *sel.* This strain was found by the authors in Australia, Germany, the UK, Hong Kong and Abu Dhabi. German and British patients usually had a travel history or family background suggesting an infection in India or Bangladesh ([111], H.J. Linde, Regensburg, Germany, pers. communication, and own observations) where it appears to be increasingly common [104].

### Sequence type 779

ST799-MRSA has been found sporadically by the authors in the UK, Ireland, France and Australia. Isolates harbour a C2 *mec* gene complex (*mecA, ugpQ*) but carriage of *ccr* genes varies (“*ccrAA*”*+ccrC* or “*ccrAA*”*+ccrA/B4*, or no detectable recombinases). Q6GD50 is detectable, possibly indicating a combination of SCC*mec* and SCC*fus* elements. ST779-MRSA are PVL-negative, but carry *etD* and *edinB.*

### Sequence type 834

According to the MLST database, ST834 belongs to CC9. However, microarray hybridisation profiles differ from other CC9 strains in several key features such as *agr* allele (*agr* group I rather than II), capsule type (8 rather than 5), *spa* type, presence of *sasG* and alleles of some MSCRAMM genes (*bbp, map, vwb*). ST834-MRSA-IV occurs rarely in Australia, where it has been described as WA MRSA-13. The tested isolates harbour *tst1, sec* and *sel*, but lack PVL genes. PVL-negative ST834-MRSA-IV also has been observed in Cambodia [165].

### Clonal complex 913

A single isolate of a ST913-MRSA-IV was identified by the authors from a Lebanese refugee in Germany who suffered from haemorrhagic bronchitis. It is positive for two genes encoding exfoliative toxins (*etA* and *etD*) as well as for *edinB*. It lacks enterotoxins and PVL. A recent paper [39] on MRSA isolates from the Negev region of Israel suggests a wider distribution of this strain in the Middle East as well as the presence of closely related ST912 and ST914.

### Sequence type 1048

This sequence type has only been reported in Hong Kong where it was sporadically isolated in nursing homes [125]. The authors found a single isolate from Hong Kong. ST1048 is related to CC7, but differs in some markers including the presence of *sasG* and *cna*, and the alleles of *arcC* and *spa.* It has *spa* type t1081, which can also be observed in CC45 and ST1774. The tested isolate carries SCC*mec* IV, *ccrC,* and ACME. It is negative for PVL and enterotoxin genes, but a previous study [125] indicated the presence of *egc* genes *seg* and *sei* in some isolates of this ST.

### Sequence type 1774

This strain was isolated from several patients in Hong Kong and has not been described previously. It shares *spa* type t1081 with other clones (CC45 and ST1048, see [125]). Isolates carry SCC*mec* IV, *ccrC,* and ACME. PVL and enterotoxin genes cannot be detected. Variable resistance markers include the β-lactamase operon and *qacC*.

## Discussion

### Strain definition and nomenclature

Genes of the “core genome” and the “core variable genome” [219] yield essentially the same phylogenetic information as the seven housekeeping genes used for MLST. Consequently, a MLST clonal complex can be identified by microarray hybridisation based on a characteristic fingerprint pattern [96], provided that a hybridisation pattern for this CC has been defined previously. For CC assignment, the presence or absence of *sasG, cna, fosB*, *lukD+lukE*, *egc*, *seh*, ORF CM14 as well as the identification of the actual allelic variant of genes of the *agr, ssl/set*, *hysA*, *hsdS,* and capsule loci and of genes encoding MSCRAMMs, proteases and a leukocidin homologue (“*lukY*”) are used [96]. Whilst clonal complex affiliation can be easily determined, the assignment to “strains” is not that straightforward. The concept of the “strain” can be beneficial for infection control purposes and subsequently, a wide variety of different epidemic or pandemic MRSA strains have been described. Unfortunately, different criteria and different methods have been applied for defining and naming MRSA strains. This has resulted in a confusing situation with several strains having multiple designations. For instance, ST5-MRSA-II is known as the Rhine-Hesse Epidemic Strain in Germany, UK-EMRSA-3 in the UK, USA100 in the USA, CMRSA-2 in Canada, AR07.3, AR07.4 or AR11 in Ireland, and New York-Japan Clone in Australia and elsewhere. For this reason, Enright *et al*. [220] proposed to designate strains by “sequence type-MRSA-SCC*mec* type” such as “ST5-MRSA-II”. We largely followed this principle. However, this nomenclature has shortcomings if clearly different strains have the same ST and SCC*mec* type (such as the various ST8-MRSA-IV). Additional information, such as PVL status or carriage of superantigens, is of relevance in defining the “pathotype” of the organism, which is important for clinical management purposes. Thus, PVL-positive and PVL-negative strains of the same ST and SCC*mec* affiliation are treated separately here, as their clinical significance may be different.

Another challenge, beyond the definition of individual strains, is that the concept of a “strain” in itself cannot properly be defined. Traditionally a strain has been defined as “an isolate or group of isolates that can be distinguished from other isolates of the same genus and species by phenotypic characteristics or genotypic characteristics or both” [221]; [222], which is essentially the same as a clone which is defined as a group of “isolates that are indistinguishable from each other by a variety of genetic tests” [221]; [222]. Thus, the definition of a clone or strain depends on the discriminatory power of the test and/or on the number of different tests applied. Isolates which appear to be indistinguishable by, *e.g.*, PFGE might yield differences detectable by microarray hybridisation or genome sequencing. In the case of *S. aureus*, all previously defined strains can be subdivided into a considerable number of variants as they harbour variable genes in different combinations [29]; [43]; [82]. Of course, it is not practical to regard all these variants as “strains” and to invent and to use different names for, *e.g*. the variants of ST22-MRSA-IV which just differ in carriage of *erm*(C) or *sec+sel.* However, UK-EMRSA-2 and UK-EMRSA-6 (both CC8-MRSA-IV) are regarded as different “strains” although they differ only in the presence of *aphA3* and *sat*, neither of which are particularly important from a clinical perspective. Similarly, Vienna and Hungarian epidemic strains differ basically in the presence of *tet*(K). On the other hand, isolates which are known as the Hannover Epidemic Strain could easily be divided into two “strains” based on their different SCC*mec* elements. In fact, a “strain” may be of polyphyletic origin. MSSA from different branches of one clonal group may have acquired the same SCC*mec* element independently on several occasions. This has previously been proposed in the case of ST5-MRSA-II [46]. These few examples emphasise the fact that the concept of “strains”, although convenient and practical, is in fact a rather arbitrary approach of forcing taxonomy on permanently changing and evolving biological subjects. When scrutinised by methods such as genome sequencing or microarray hybridisation, “strains” are not static blocks comprised of identical isolates, but rather consist of groups of isolates with similar sequences. These sequences might differ in single point mutations (as sometimes obvious in MLST, *e.g*., Taiwan Clone isolates may have different *gmk* alleles), in the composition and sequence of single loci (such as the variable part of the *spa* gene or the *dru* region within the SCC*mec* element) or in the presence or absence of complete genes or multi-gene mobile elements. Thus, the concept of “quasispecies” [223]-[227] could be applied in which the genome “cannot be described as a defined structure, but rather as a weighted average of a large number of individual sequences” [228]. The difference between *S. aureus* and the RNA-viruses (to which this concept has been applied first [228]), is basically the time frame in which variations evolve. Conveniently, the extent and time frame of variability in *S. aureus* provide ample opportunities for typing, *i.e.,* for outbreak investigations and infection control purposes.

### Biodiversity of MRSA and SCC*mec* elements

**Figure 5:**
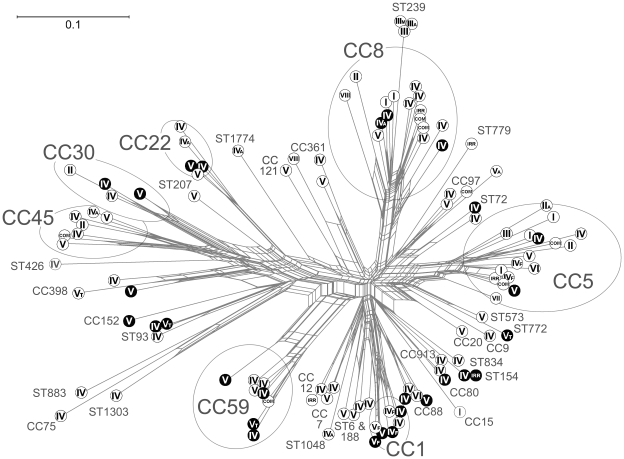
Network graph based on hybridisation profiles, visualising similarities and relationships between clonal complexes and the spread of SCC*mec* elements. Roman numerals indicate SCC*mec* types; PVL-negative strains are shown with black letters on white background, PVL-positive in white letters on black. A, ACME; F, SCC*fus*; M, SCC*mer*; IRR, irregular SCC*mec* elements; COM, composite or multiple SCC*mec* elements.

For more than three decades, MRSA was mainly an issue of hospital hygiene and infection prevention and control. Interestingly, the “older” SCC*mec* elements found in HA-MRSA strains have largely been observed in and restricted to a few genotypes, mainly to CC5 and CC8. CC5 and CC8 harbour the widest diversity of SCC*mec* elements, and some of the most recently described types have only been found in these CCs (Figure 5). This is not related to their overall abundance, *i.e*., to the statistical probability of a gene transfer event. In contrast, MRSA from the equally abundant CC15 [94] has only been described once, some 30 years ago [33]. Thus, SCC*mec* elements from other species may be more readily integrated into CC5 and CC8 than into other *S. aureus* lineages. These two CCs might serve as some kind of “entry gate” of SCC*mec* elements into the *S. aureus* gene pool from which, possibly after some adaptations, these elements can be transferred into other *S. aureus* lineages. The reason for this is unclear, but peculiarities of, *e.g*., lineage-specific restriction-modification systems which can control horizontal gene transfer into *S. aureus* and between different *S. aureus* could be scrutinised with in this regard.

The epidemiology of MRSA has changed over the last two decades with the rise of CA- and LA-MRSA harbouring a previously unknown variety of SCC*mec* elements. It is tempting to speculate that the rise of new strains was not only paralleled, but triggered by the emergence of these elements, especially of SCC*mec* IV. Compared to other SCC*mec* elements, SCC*mec* IV can be found in a wide diversity of different *S. aureus* clonal lineages (Figure 5). Interestingly, it is also the most common SCC*mec* element in *S. epidermidis.* It has been detected in approximately 40% of methicillin-resistant *S. epidermidis* from humans, which belonged to wide variety of genetic backgrounds [229]; and it is also common among coagulase-negative staphylococci from animals [230]. The evolutionary success of this element might suggest either a better ability to be transmitted between different strains, and even species, and/or lower fitness costs [7]; [8]. Recently, a variety of “atypical” SCC*mec* elements have been observed, some of which are described in this study. Whether they are variants of previously known types or entirely new elements still needs to be determined. However, these observations indicate that the spread and evolution of SCC*mec* elements is still ongoing. This prompts the question of where novel elements originated from. Apparently, these elements evolve independently of the *S. aureus* genome they actually reside in. Different SCC*mec* elements may be observed in otherwise virtually identical strains, and strains from completely different clonal complexes may acquire the same SCC*mec* elements. Similar to other mobile genetic elements they could be considered as “selfish genes” [231], *i.e.*, as parasitic pieces of genetic information which undergo their own evolution and compete against each other, as previously discussed by Novick (“Mobile genetic elements are arguably selfish in that their evolution is driven by selective forces that operate on the elements themselves, independently of the host organisms within which they must of necessity reside” [193]). However, contrary to other “selfish” mobile genes (such as endogenous retroviruses in eukaryotic genomes) they confer an advantage to their “host organism” by introducing antibiotic resistance properties and even additional virulence factors such as a recently discovered phenol-soluble modulin [232]. The relative independence of such elements from host genomes also allows them to reside in other species, and, indeed, a wide range of SCC*mec* elements can be found in other staphylococci [229]. These bacteria provide an ample pool of additional hosts for the SCC*mec* elements, and it can be expected that novel elements may be transferred from other staphylococci to *S. aureus*. For that reason, the evolution of antibiotic resistance in bacteria infecting/colonising livestock is highly relevant. Many animal species harbour *S. aureus* as well as their own host-specific staphylococci (such as *S. hyicus* in swine or *S. pseudintermedius* in dogs). Global livestock populations now exceed the populations of most wild animals which might serve as hosts for *S. aureus*. For example, there are 1,300 million cattle, 900 million pigs, 500 million cats and 400 million dogs in the world [http://www.cattle-today.com/, http://en.wikipedia.org]. Because of their close proximity to humans, domestic animals might serve as reservoir for new strains as well as for novel SCC*mec* elements. While some animal-specific *S. aureus* such as ST151 have so far failed to evolve into MRSA or to infect humans, others have truly zoonotic potential with the best documented example being ST398-MRSA-V.

### The role of Panton-Valentine leukocidin

Although PVL has been known for a long time [10]; [233], its occurrence in MRSA strains is a recent phenomenon. Since PVL is phage-encoded, it can be found in *S. aureus* belonging to many different clonal complexes. Occurrence in diverse MSSA has been discussed previously [113]. PVL-positive MRSA are found in clonal complexes or sequence types CC1, CC5, CC8, CC22, CC30, CC59, ST72, CC80, CC88, ST93, ST154, ST398 and ST772 (Figure 5). Additionally, PVL-positive ST45-MRSA have recently been reported [45]; [122]. As with SCC*mec* elements, PVL phages undergo a host-independent evolution. As a set of “selfish genes” they evolved to confer a selective advantage to their staphylococcal hosts in helping them to cope with the immune defences of the vertebrates in which these staphylococci parasitize. Thus, these phages compensate their staphylococcal host for the fitness costs they may cause. This strategy appears to be evolutionary successful as it evolved on multiple occasions. Staphylokinase-encoding phages in *S. aureus* can be regarded as another example. Staphylokinase improves the fitness of *S. aureus* in the human host by facilitating their entry into deeper host tissues and by inhibiting host defensins [234]. SCC*mec* elements also “pay” for being transmitted and multiplied by helping their staphylococcal hosts to cope with antibiotic compounds, which in a certain sense also belong to the defences of the human host against staphylococcal infection.

Some of the recently emerged PVL-positive MRSA strains are known to occur and predominate in certain regions such as USA300 in the USA, ST80-MRSA-IV in Europe and the Middle East, ST772-MRSA-V in India, ST59-MRSA-V_T_ in Taiwan and ST93-MRSA-IV in Australia. Cases outside these regions are increasingly being reported. In part, this can be attributed to human travel activities. Other PVL-positive strains have been found in multiple distant settings (*e.g.*, ST22-MRSA-IV in Bavaria, Germany, in Australian patients of Indian origin, in Abu Dhabi and in Great Britain) which might suggest a polyphyletic origin (see also [40]). Crudely, three different epidemiological situations can be distinguished. First, in European countries (such as Germany, [29]; ; the UK, [111]; Malta, [40] or Ireland, [57]; [235]), the prevalence of PVL-MRSA is low and has remained low for several years. In these countries, a variety of different strains can be observed and individual cases can often be traced to travel histories or to the foreign origin of patients. Thus, detection of such strains in travellers might indicate an epidemic situation elsewhere, and should prompt a thorough documentation of the patient's travel history. It may be speculated that an overwhelming presence of successful PVL-negative clones (ST22-MRSA-IV and ST398-MRSA-V) may hinder the dissemination of PVL-positive clones. However, there are no data indicating whether these strains would prevail in direct competition with a successful PVL-MRSA-clone, and generally it is not known which properties might render a strain “successful”. Secondly, in Australia or Abu Dhabi [31] PVL-MRSA are common and a number of different strains co-exist. Since Australia as well as the Gulf Emirates witnessed a recent and massive immigration of people from all over the world, it can be assumed that these people have introduced epidemic strains from their respective home countries. For instance, USA300 may have come with North American expatriates, or ST772-MRSA-V from India [111]. Thirdly, another situation emerged in the USA, where a single strain of PVL-MRSA (USA300) spread extensively and where this one strain effectively marginalised all other strains, whether PVL-positive or not [76]. Similarly, in Taiwan, most MRSA infections are caused by ST59-MRSA-V_T_ [14]; [236]. A comparable picture may currently evolve in Australia due to a massive increase in ST93-MRSA-IV infections.

The role of PVL in MRSA and especially in USA300 has been extensively and controversially discussed [61]; [237]-[239], with particular emphasis on the situation in the USA. However, other parts of the world harbour other PVL-MRSA. If different strains from diverse clonal groups cause the same distinct syndromes (such as chronic/recurrent SSTI in immunocompetent young adults) the causative factor should be present in all of them. Thus, the observation that, *e.g.,* PVL-positive CC5, CC8 and CC30 isolates can cause the same symptoms, but behave differently than essentially isogenic but PVL-negative CC5, CC8 and CC30 strains rendered it improbable that a factor other than PVL was the key marker of virulence in PVL-positive MRSA. As PVL-MRSA belong to different *agr-*groups, a connection of the evolutionary success of PVL-MRSA to *agr* group affiliation, *i.e*., to peculiarities of gene regulation is also improbable. The diversity and abundance of PVL-MRSA clones in Abu Dhabi and Australia could also indicate that no USA300-specific factor besides PVL (such as ACME) was necessary for the expansion of a PVL-MRSA clone. Thus, our observations suggest that PVL plays a key role in the evolutionary success of MRSA in a clearly defined ecological niche of chronic/recurrent SSTI in otherwise healthy young adults and, rarely, of necrotising pneumonia. In settings where PVL-MRSA is abundant, they also have been observed to cause, *e.g*., bloodstream infections [240]. However, this can be attributed to their overall abundance and to the relative rarity of other strains, since PVL is thought not to contribute to the pathogenesis of bloodstream infections [241].

### Outlook

The evolving issue of community-acquired and livestock-associated MRSA poses a major public health threat. The biological diversity of MRSA is increasing and this enables MRSA to move out of the small and relatively controllable ecological niche of hospitals and intensive care units into the general population of developed and developing nations and into livestock animals. Consequently, it can no longer be considered an exclusive hospital-associated problem, and it cannot be fought by hospital infection prevention and control measures alone. Some of the MRSA strains may replace MSSA in a similar way as penicillinase-positive strains replaced penicillinase-negative strains in the 1950s and 1960s. A consequence of such a development may be that β-lactam antibiotics could only be used when proven to be effective, *i.e.* not as an initial therapy but only after susceptibility testing has been performed. This would result in an increased use of antimicrobials which are more expensive and/or less effective and in dire consequences for individual patients as well as for entire national healthcare systems.

Due to the increasing biodiversity of MRSA and the resulting exploitation of novel ecological niches outside of hospitals it cannot be realistically expected that MRSA might be eradicated easily. In order to check its current proliferation, factors which confer advantage to MRSA need to be turned into a disadvantage. Thus, carriage of *mecA* and/or PVL needs to be “penalised” by consequent treatment, eradication and infection prevention and control measures. Practically, a “search-and-destroy” policy as in Scandinavian countries, the Netherlands and Western Australia is warranted. This requires routine screening assays for *mecA* and/or PVL as well as advanced typing techniques and we anticipate that the methods and data from our study might contribute to this.

## Materials and Methods

### Strains and isolates

A database of microarray experiments performed by the authors on more than 3,000 MRSA isolates has been used for this study. Isolates have been collected as part of routine diagnostic work from the following sources: the Dresden University Hospital (Saxony, Germany) and hospitals of Hoyerswerda (Saxony, Germany) and Saarbrücken (Saarland, Germany), a variety of German intensive care units (as part of the S.A.R.I. study, [242]), the “Friedrich-Loeffler-Institut” (Federal Research Institute for Animal Health, Germany), the national MRSA reference centres in London (UK), Lyon (France) and Dublin (Ireland), hospitals in Ireland, Msida (Malta), Abu Dhabi (United Arab Emirates), Hong Kong (China), Trinidad & Tobago and from the Gram-positive Bacteria Typing and Research Unit, Perth (Australia). The majority of isolates were sampled between 2000 and August 2010. A small number of older isolates were also included (such as for ST247- and ST250-MRSA-I); this is mentioned in the text along with the strain descriptions. Additionally, a collection of reference strains from the Network on Antimicrobial Resistance in *S. aureus* (NARSA, Herndon, Virginia, USA) was characterised, mainly to reflect the diversity of MRSA strains in the USA. A few strains originated from other sources that are acknowledged in the respective sections and in the Acknowledgments.

Additional information on emerging MRSA strains and their geographic distributions as reported in recent publications has also been included.

### Sequence-based typing

Multilocus sequence typing (MLST), which is based on sequencing of internal fragments of *arcC, aroE, glpF, gmk, pta, tpi* and *yqiL* housekeeping genes, was performed on selected isolates. The protocol used was as described by Enright *et al*. [25]. The sequences obtained were compared with those at the MLST website (http://saureus.mlst.net/) to assign a sequence type (ST). Related sequence types were clustered to clonal complexes (CC) using BURST analyses as provided on the MLST website.

The *spa* typing procedures were performed according to previously published protocols [243] using the nomenclature as described on the Ridom website (http://spa.ridom.de/) and either the RIDOM or SPATYPEMAPPER (freeware, download at http://www.clondiag.com/fileadmin/Media/Downloads/SPATypeMapper_0_6.zip) software packages.

### DNA microarray-based typing

The Alere StaphyType DNA microarray was employed using protocols and procedures previously described in detail [29]; [ 96]. The DNA microarray covers 334 target sequences, (approximately 170 distinct genes and their allelic variants) including species markers, SCC*mec*, capsule and *agr* group typing markers, resistance genes, exotoxins, and MSCRAMM genes. Primer and probe sequences have been published previously [29]; [96].

Target genes and information on primers and probes are provided in Supplemental file S1.

MRSA were grown on Columbia blood agar and incubated overnight at 37°C. Culture material was enzymatically lysed prior to DNA preparation using commercially available spin columns (Qiagen, Hilden, Germany). Purified DNA samples were used as templates in a linear primer elongation using one primer per target. All targets were amplified simultaneously, and within this step, biotin-16-dUTP was incorporated into the resulting amplicons. An alternate protocol was used for a few isolates in which amplification and labelling were directed by random primers [139]; [244]. As this protocol does not rely on conserved primer binding sites, it proved to be useful for characterisation of unusual strains which are not fully represented by the published genome sequences (*e.g.,* ST75 strains).

Amplicons obtained using either protocol were hybridised to the microarray followed by washing and blocking steps, and the addition of horseradish-peroxidase-streptavidin conjugate. After further incubation and washing steps, hybridisations were visualised by using a precipitating dye. An image of the microarray was taken and analysed using a designated reader and software (ALERE Technologies GmbH, Jena, Germany). Normalised intensities of the spots were calculated based their average intensities and on the local background [96]. Results were regarded as negative if the normalised intensity for a given probe was below 25% of the median value of species markers (*coa, eno, fnbA, gapA, katA, nuc, rrn, sarA sbi, spa, vraS*) and a biotin staining control. If the normalised intensity of a given probe was higher than 50% of this breakpoint, it was interpreted positive. If it was between 25% and 50%, the result was regarded as ambiguous. For some markers, for which allelic variants were to be discriminated (*bbp, clfA, clfB* and *fnbB* as well as some *set/ssl* genes, *isaB, mprF* and *isdA*), a different approach was used because these alleles differed only in single nucleotides. Here, only the probe with the strongest signal value was regarded as positive, provided that it exceeded the 50% breakpoint. All others were regarded as ambiguous or, if below the 25% breakpoint, as negative. This allowed an easy and clear distinction of clonal complex-specific variants of these genes. Genes which are not present in all tested isolates are labelled as rare, variable or common in Figures 2 and 3 as well as in Supplement S2 (see legends).

The affiliation of isolates to clonal complexes (CCs) or sequence types (STs) as defined by MLST [25] was determined by an automated comparison of hybridisation profiles to a collection of reference strains previously characterised by MLST [29]; [96]. Analysis of hybridisation patterns cannot discriminate sequence types which differ only in single point mutations affecting MLST genes (*e.g.,* ST5 and ST225, or ST59 and ST952). However, there are also sequence types which originate from chromosomal replacements as previously described [170]; [173]. As these events result in different hybridisation patterns, such STs can be easily identified. MRSA strains which belong to these sequence types will be described separately from the parental CCs.

### SCC*mec* typing

The SCC*mec* elements were typed using previously described PCR primers and conditions and by array hybridisation as described below.

For PCR-based typing, structural architecture and *mec*-complex were determined using the primers described by Zhang *et al.,* 2005 [132]. SCC*mec* type IV was further sub-typed using published primers [245]. The cassette chromosome recombinase (*ccr*) was typed as described previously [246]. An ISau4-like transposase (GenBank accession number DQ680163) inserted into the open reading frame V011 of SCC*mec* V_T_ was detected by the production of a *ca*. 1,600-bp PCR and confirmed by sequencing [126].

All Irish MRSA isolates underwent SCC*mec* typing for (i) the *ccr* and *mec* complex genes and (ii) the J regions and *mecI* [15]; [85]; [246]; [247]. In addition, isolates harbouring SCC*mec* IV underwent SCC*mec* IV sub-typing. Sub-typing of variant SCC*mec* II and IV elements from Irish isolates was performed as described previously [15]; [ 245].

The array includes probes for *mecA*, an accompanying gene, *ugpQ*, *mecI* and *xylR*. Two different probes for *mecR1* allow the discrimination of un-truncated *mecR1* and truncated *mecR1 (ΔmecR1)*. Four different alleles of *ccrA* and *ccrB* (*ccrA-1, ccrB-1, ccrA-2, ccrB-2, ccrA-3, ccrB-3, ccrA-4* and *ccrB-4*) can be distinguished. The gene *ccrC* and a “hypothetical protein” accompanying *ccrC* form an additional pair of recombinase genes. Because the latter is an analogue to *ccrA*, it is here tentatively named “*ccrAA*”. Alleles from strain 85-2082 (GenBank AB037671) and strain MRSAZH47 (GenBank AM292304) can be distinguished by different probes. While all SCC*mec* V strains react with the former probe, only a part of them yield signals with probes for the latter variant. This includes strains ST398-MRSA-V and Taiwan Clone ST59-MRSA-V_T_ which are known to harbour a distinct variant, SCC*mec* V_T_ (5C2&5).

J-region genes *dcs*, *pls-*SCC and the *kdp*-operon can also be detected. Other genes with relevance for SCC*mec* are a mercury resistance operon, the tobramycin resistance gene *aadD,* the macrolide, lincosamide and streptogramin B resistance gene *erm*(A), the tetracycline resistance gene *tet*(K) and the fusidic acid resistance marker Q6GD50. However, as these genes are plasmid- or transposon-encoded, they are not necessarily restricted to SCC*mec* elements. An overview on hybridisation patterns associated with the different SCC*mec* types is provided in Figure 1.

### Tree reconstruction

In order to visualise similarities between hybridisation profiles, a network tree using SplitsTree software [248] was constructed. Array hybridisation profiles of the tested strains (Supplemental file S3) were converted into a series of ‘sequences’ (Supplemental file S5). Each position in this ‘sequence’, *i.e*., each probe, could have a value of ‘positive’ (‘C’), ‘negative’ (‘G’), ‘ambiguous’ (‘A’) or ‘variable’ (‘T’) with the latter including all these markers which are in Supplemental file S3 designated as ‘rare’, ‘variable’ or as ‘common’. These ‘sequences’ were used with SplitsTree version 4.11.3 on default settings (characters transformation: uncorrected P/ignore ambiguous states, distance transformation: Neighbour-Net, and variance: ordinary least squares). For CC15 and ST426, data from MSSA [96] were used as no MRSA isolates were found. The scale bar represents the number of differences between signal strings (with 0.1 meaning 10% difference).

Due to the high rate of recombination affecting many genes covered by the array, this tree does not reflect necessarily true phylogenetic relations. However, the observation that strains of the same clonal complex cluster together indicates that phylogenetic relationships indeed result in similar hybridisation patterns and, on a practical level, that the hybridisation profile can be used to predict CC affiliation [96]. Similarly, also CCs which are related according to sequence analyses (*e.g*., CC22, CC30, CC45 and ST207, see [115]) also cluster together.

For comparison, a SplitsTree analysis of the concatenated MLST sequences (Supplemental file S4) of all STs mentioned herein is provided as Figure 4.

## Supporting Information

File S1 Target genes, probes and primers.
(PDF)

File S2 Overview of sequence types (STs), *spa* types, some characteristic genomic markers and fully sequenced genomes of the clonal complexes (CCs) described in this study (**bold typeset** indicates STs and *spa* types identified in the present study; *italic typeset* indicates STs found by the authors in MSSA isolates or *spa* types described in literature or public databases such as RIDOM; *, see text for further explanation).
(PDF)

File S3 Complete hybridisation results for MRSA strains examined in this study.
(PDF)

File S4 "nexus"-file used for the network graph based on concatenated MLST sequences (Figure 4).
(NEX)

File S5 "nexus"-file used for the network graph based on hybridisation profiles (Figure 5).
(NEX)
