# Supplementary material for: A Field Guide to Pandemic, Epidemic and Sporadic Clones of Methicillin-Resistant Staphylococcus aureus
Source: PLoS One. 2011 Apr 6;6(4):e17936. doi: 10.1371/journal.pone.0017936 (PMC3071808; doi:10.1371/journal.pone.0017936)
Supplement: File S1 — Target genes, probes and primers. (PDF) [file pone.0017936.s001.pdf]

Gene	Synonyma	Gene product /function	Alleles	Probe name	Probe definition	Primer name	Primer definition
aacA-aphD		bifunctional enzyme Aac/Aph, gentamicin resistance		aacA-aphD_10,4	AB096217.1 [28286:28313]	aacA-aphD_PM4	AB096217.1[28367:28386]
aadD		aminoglycoside adenyltransferase,tobramycin resistance		aadD_1,2	BA000017.4 [41203:41230:r]	aadD_PM4	BA000017.4[41144:41164:r]
agrB		accessory gene regulator B	*agrB-I*	agrB-I_11	CP000046.1 [2083620:2083646]	agrB-I_51	CP000046.1[2083674:2083696:r]
			agrB-II	agrB-II_11	BA000017.4 [2156206:2156234]	agrB-II_51	BA000017.4[2156235:2156253:r]
			agrB-III	agrB-III_11	BX571856.1 [2087653:2087682]	agrB-III_51	BX571856.1[2184604:2184626:r]
			agrB-IV	agrB-IV_11	AF288215.1 [1200:1226]	agrB-IV_51	AF288215.1[1255:1275:r]
agrC		accessory gene regulator C	*agrC-I*	agrC-I_12	CP000046.1 [2084385:2084411]	agrC-I_51	CP000046.1[2084470:2084490:r]
			agrC-II	agrC-II_11	BA000017.4 [2156768:2156793]	agrC-II_51	BA000017.4[2156859:2156881:r]
			agrC-III	agrC-III_11	BX571856.1 [2185117:2185143]	agrC-III_51	BX571856.1[2185152:2185173:r]
			agrC-IV	agrC-IV_11	AF288215.1 [1553:1580]	agrC-Ia_51	AF288215.1[2049:2069:r]
						agrC-IV_51	AF288215.1[1609:1631:r]
						agrC-IV_52	AJ617711.1[867:889:r]
agrD		accessory gene regulator D	*agrD-I*	agrD-I_11	CP000046.1 [2083761:2083788]	agrD-I_51	CP000046.1[2083792:2083813:r]
				agrD-I_12	CP000046.1 [2083765:2083788]	agrD-II+I_51	CP000046.1[2083820:2083842:r]
				agrD-I_13	CP000046.1 [2083761:2083783]		
			agrD-II	agrD-II_11	BA000017.4 [2156525:2156554]	agrD-II_51	BA000017.4[2156556:2156577:r]
			agrD-III	agrD-III_11	BX571856.1 [2184647:2184676]	agrD-III_51	BX571856.1[2184683:2184699:r]
aphA3		3'5'-aminoglycoside phosphotransferase, neo-/kanamycin resistance		aphA-3_18,3	AY602209.1 [105:130]	aphA-3_PM4	AY602209.1[206:223]
arcA		ACME-locus: arginine deiminase	*arcA-SCC*	hp_arcA_611	AE015929.1[102505:102530:r]	lb_arcA_651_rv	AE015929.1[102460:102479]
arcB		ACME-locus: ornithincarbamoyltransferase	*arcB-SCC*	hp_arcB_611	AE015929.1[99281:99307:r]	lb_arcB_651_rv	AE015929.1[99256:99274]
arcC		ACME-locus: carbamatkinase	*arcC-SCC*	hp_arcC_611	AE015929.1[98603:98631:r]	lb_arcC_651_rv	AE015929.1[98571:98590]
arcD		ACME-locus: arginine/ornithine-antiporter	*arcD-SCC*	hp_arcD_611	AE015929.1[101412:101440:r]	lb_arcD_651_rv	AE015929.1[101381:101398]
aur		aureolysin	*aur (cons)*	hp_aur_613	CP000046.1 [2721001:2721030:r]	lb_aur_651_rv	CP000046.1[2721434:2721453]
			aur (Other than MRSA252)	hp_aur_611	CP000046.1 [2721468:2721496:r]	lb_aur_653_rv	CP000046.1[2720967:2720987]
			aur (MRSA252)	hp_aur_612	BX571856.1 [2812419:2812446:r]	lb_aur_652_rv	BX571856.1[2812384:2812405]
bap		surface protein involved in biofilm formation		hp_bap_611	AY220730.1 [7832:7860]	lb_bap_651_rv	AY220730.1[7869:7891:r]
bbp		bone sialoprotein-binding protein	*bbp (cons)*	hp_bbp_614	CP000046.1 [640403:640431]	lb_bbp_654_rv	CP000046.1[640459:640476:r]
			bbp (ST45)	hp_bbp_611	AM076252.1 [3:31]	lb_bbp_656_rv	CP000046.1[642598:642620:r]
			bbp (RF122)	hp_bbp_612	AJ938182.1 [578264:578291]	lb_bbp_651_rv	AM076252.1[37:59:r]
			bbp (MRSA252)	hp_bbp_613	BX571856.1 [621217:621242]	lb_bbp_653_rv	BX571856.1[621266:621285:r]
			bbp (COL+MW2)	hp_bbp_616	CP000046.1 [642547:642573]	lb_bbp_655_rv	BX571856.1[621919:621939:r]
			bbp (Mu50)	hp_bbp_617	BA000017.4 [638429:638457]	lb_bbp_657_rv	BA000017.4[638483:638502:r]
						lb_bbp_652_rv	AJ938182.1[578305:578327:r]
blaI		beta lactamase repressor (inhibitor)		hp_blaI_611	BX571856.1 [1911923:1911949:r]	lb_blaI_651_rv	BX571856.1[1911882:1911903]
blaR		beta-lactamase regulatory protein		hp_blaR_611	BX571856.1 [1912975:1913002:r]	lb_blaR_652_rv	BX571856.1[1912922:1912942]
				hp_blaR_612	BX571856.1 [1912288:1912317:r]	lb_blaR_653_rv	BX571856.1[1912251:1912274]
blaZ		beta-lactamase		blaZ_11	AB074882.1 [417:441]	blaZ_PM4	BX571856.1[1914099:1914121]
				blaZ_4,2	BX571856.1 [1913997:1914021]	lb_blaZ_651_rv	BX571856.1[1914034:1914054:r]
				hp_blaZ_611	BX571856.1 [1914503:1914531]	lb_blaZ_652_rv	BX571856.1[1914553:1914570:r]
						lb_blaZ_653_rv	DQ016047.1[1101:1120:r]
capH		capsular polysaccharide synthesis enzyme CapH of capsule types 1, 5, and 8	*capH1*	hp_capH1_611	U10927.2 [19165:19192]	lb_capH1_651_rv	U10927.2[19210:19230:r]
			capH5	hp_capH5_611	CP000046.1 [161120:161144]	lb_capH5_651_rv	CP000046.1[161160:161180:r]
			capH8	hp_capH8_611	BX571856.1 [176513:176541]	lb_capH8_651_rv	BX571856.1[176544:176565:r]

Gene	Synonyma	Gene product /function	Alleles	Probe name	Probe definition	Primer name	Primer definition
capI		capsular polysaccharide biosynthesis protein CapI	*capI8*	hp_capI8_612	BX571856.1 [178269:178298]	lb_capI8_651_rv	BX571856.1[178195:178214:r]
						lb_capI8_652_rv	BX571856.1[178312:178332:r]
capJ		O-antigen polymerase CapJ of capsule types 1, 5, and 8	*capJ1*	hp_capJ1_611	U10927.2 [21322:21350]	lb_capJ1_651_rv	U10927.2[21367:21385:r]
			capJ5	hp_capJ5_611	CP000046.1 [163178:163206]	lb_capJ5_651_rv	CP000046.1[163216:163233:r]
				hp_capJ5_612	CP000046.1 [163344:163373:r]	lb_capJ5_652_rv	CP000046.1[163316:163337]
			capJ8	hp_capJ8_611	BX571856.1 [178770:178800]	lb_capJ8_651_rv	BX571856.1[178809:178826:r]
capK		capsular polysaccharide biosynthesis protein CapK of capsule types 1, 5, and 8	*capK1*	hp_capK1_611	U10927.2 [22439:22466]	lb_capK1_651_rv	U10927.2[22490:22508:r]
			capK5	hp_capK5_611	CP000046.1 [164536:164564]	lb_capK5_651_rv	CP000046.1[164576:164595:r]
			capK8	hp_capK8_611	BX571856.1 [178990:179017]	lb_capK8_651_rv	BX571856.1[179021:179045:r]
				hp_capK8_612	BX571856.1 [179930:179956]	lb_capK8_652_rv	BX571856.1[179958:179980:r]
cat		chloramphenicol acetyltransferase	*cat-pC221*	hp_cat_613	M64281.1 [358:389]	lb_cat_654_rv	AB080798.1[2860:2878:r]
			cat-pC223	hp_cat_611	AF507977.1 [17615:17642]	lb_cat_653_rv	AF507977.1[17658:17682:r]
			cat-pMC524	hp_cat_612	AB080798.1 [2826:2854]	lb_cat_655_rv	AJ312056.2[587:605:r]
			cat-pSBK203R	hp_cat_615	M58515.1 [353:384]	lb_cat_652_rv	M58515.1[407:431:r]
ccrA		cassette chromosome recombinase A	*ccrA-1*	hp_ccrA-1_611	CP000046.1 [48646:48672:r]	lb_ccrA-1_651_rv	CP000046.1[48623:48642]
			ccrA-2	hp_ccrA-2_611	BA000017.4 [66314:66342:r]	lb_ccrA-1_652_rv	CP000046.1[48372:48390]
			ccrA-3	hp_ccrA-3_611	AB014436.1 [254:279]	lb_ccrA-2_651_rv	BA000017.4[66290:66306]
			ccrA-4	hp_ccrA-4_612	AF411935.1 [8756:8782]	lb_ccrA-2_652_rv	BA000017.4[65738:65756]
						lb_ccrA-2_653_rv	AB063173.1[6486:6506:r]
						lb_ccrA-3_651_rv	AB014436.1[283:300:r]
						lb_ccrA-3_652_rv	AB014436.1[826:844:r]
						lb_ccrA-4_651_rv	AF411935.1[8582:8602:r]
						lb_ccrA-4_652_rv	AF411935.1[8803:8823:r]
"ccrAA"		hypothetical protein accompanying ccrC	*ccrAA-85-2082*	hp_ccrAA_611	AP008934.1 [56540:56569]	lb_ccrAA_652_rv	AB037671.1[61397:61418]
			ccrAA-MRSAZH47	hp_ccrAA_612	AP006716.1 [63246:63271]	lb_ccrAA_651_rv	AM292304.1[6629:6648:r]
				hp_ccrAA_613	AP006716.1 [63636:63661]	lb_ccrAA_653_rv	AM292304.1[7020:7038:r]
ccrB		cassette chromosome recombinase B	*ccrB-1*	hp_ccrB-1_612	CP000046.1 [47628:47652:r]	lb_ccrB-1_651_rv	CP000046.1[47771:47788]
				hp_ccrB-1_613	CP000046.1 [47794:47823:r]	lb_ccrB-1_652_rv	CP000046.1[47594:47614]
			ccrB-2	hp_ccrB-2_611	BA000017.4 [63943:63970:r]	lb_ccrB-2_651_rv	BA000017.4[63916:63937]
			ccrB-3	hp_ccrB-3_611	AB014436.1 [2110:2135]	lb_ccrB-2_652_rv	DQ483074.1[378:401:r]
			ccrB-4	hp_ccrB-4_611	AY918294.1 [319:343]	lb_ccrB-2_653_rv	BA000017.4[63678:63697]
						lb_ccrB-3_651_rv	AB014436.1[2160:2179:r]
						lb_ccrB-3_652_rv	AB014436.1[2265:2286:r]
						lb_ccrB-4_651_rv	AE015929.1[59580:59600]
						lb_ccrB-4_652_rv	AE015929.1[59457:59474]
ccrC		cassette chromosome recombinase		hp_ccrC_611	AP008934.1 [57303:57333]	lb_ccrC_651_rv	AB037671.1[60643:60662]
cfr		23S rRNA methyltransferase		hp_cfr_611	AJ249217.1 [1048:1074]	lb_cfr_651_rv	AJ249217.1[1075:1093:r]
chp		chemotaxis-inhibiting protein (CHIPS)		hp_chp_611	BX571856.1 [2126835:2126861]	lb_chp_651_rv	BX571856.1[2126883:2126903:r]
				hp_chp_612	BX571856.1 [2127086:2127114]	lb_chp_652_rv	BX571856.1[2127127:2127150:r]
clfA		clumping factor A	*clfA (cons)*	hp_clfA_611	CP000046.1 [881138:881166]	lb_clfA_651_rv	CP000046.1[881192:881211:r]
			clfA (COL+RF122)	hp_clfA_612	CP000046.1 [882182:882210]	lb_clfA_652_rv	CP000046.1[882220:882241:r]
			clfA (MRSA252)	hp_clfA_613	BX571856.1 [889789:889814]		
			clfA (Mu50+MW2)	hp_clfA_614	BA000017.4 [888713:888737]		
clfB		clumping factor B	*clfB (cons)*	hp_clfB_611	CP000046.1 [2713245:2713275:r]	lb_clfB_651_rv	CP000046.1[2713215:2713233]
			clfB (COL+Mu50)	hp_clfB_612	CP000046.1 [2712297:2712328:r]	lb_clfB_652_rv	CP000046.1[2712249:2712271]
			clfB (MW2)	hp_clfB_613	AM075901.1 [1069:1098]	lb_clfB_654_rv	AM075915.1[1125:1146:r]
			clfB (RF122)	hp_clfB_614	AJ938182.1 [2647736:2647765:r]	lb_clfB_653_rv	BX571856.1[2803198:2803221]

Gene	Synonyma	Gene product /function	Alleles	Probe name	Probe definition	Primer name	Primer definition
cna		collagen-binding adhesin		hp_cna_611	BX571856.1 [2879879:2879905:r]	lb_cna_651_rv	BX571856.1[2879853:2879871]
coa		coagulase		coa_consens_11	CP000046.1 [246925:246954]	coa_consens_PM4	CP000046.1[246967:246988]
dcs-Q9XB68		hypothetical protein from SCCmec elements		hp_Q9XB68_611	CP000046.1 [34948:34976]	lb_Q9XB68_651_rv	CP000046.1[35004:35027:r]
dfrA		dihydrofolate reductase type 1		dfrA_12	AE017171.1 [2588:2614:r]	dfrA_PM4	AE017171.1[2494:2513:r]
				2,1-dfrA	AB049452.1 [2076:2103]		
ebh		cell wall associated fibronectin-binding protein		hp_ebh-3prime_611	CP000046.1 [1483834:1483860:r]	lb_ebh-3prime_651_rv	CP000046.1[1483793:1483813]
ebpS		cell surface elastin binding protein	*ebpS (COL)*	hp_ebpS_613	CP000046.1 [1561278:1561303:r]	lb_ebpS_651_rv	CP000046.1[1561493:1561511]
				hp_ebpS_612	CP000046.1 [1561514:1561541:r]	lb_ebpS_652_rv	CP000046.1[1561245:1561268]
				hp_ebpS_614	CP000046.1 [1560815:1560839:r]	lb_ebpS_653_rv	CP000046.1[1560784:1560801]
			ebpS-01-1111 (from CC45)	hp_ebpS_611	AM075954.1 [148:172]		
edinA		epidermal cell differentiation inhibitor precursor		edinA_11	M63917.1 [460:489]	edinA_51	M63917.1[502:520:r]
edinB		epidermal cell differentiation inhibitor B		edinB_11	AB057421.1 [7445:7471]	edinB_51	AB057421.1[7482:7501:r]
edinC		epidermal cell differentiation inhibitor C		edinC_11	AP003088.1 [1810:1839:r]	edinC_51	AP003088.1[1755:1776]
eno		enolase		hp_eno_611	CP000046.1 [870472:870501]	lb_eno_651_rv	CP000046.1[870526:870544:r]
***erm* (A)**	*ermA*	rRNA adenine N-6-methyltransferase, erythromycin/clindamycin resistance		ermA_9,4	BA000017.4 [1762850:1762875]	ermA_PM4	BA000017.4[1762907:1762928]
***erm* (B)**	*ermB*	erythromycin/clindamycin resistance		hp_ermB_611	EF450709.1 [2405:2428]	lb_ermB_651	EF450709.1 [2443:2464]
				hp_ermB_612	EF450709.1 [2528:2557]	lb_ermB_652	EF450709.1 [2564:2584]
***erm* (C)**	*ermC*	erythromycin/clindamycin resistance		ermC_8,1	M17990.1 [1775:1799]	ermC_PM4	AF466402.1[130:150]
				ermC_8,2	M17990.1 [1840:1864]		
etA		exfoliative toxin serotype A		etA_8,2	AP001553.1 [42317:42344]	etA_PM4	AP001553.1[42387:42406]
etB		exfoliative toxin serotype B		etB_9,3	AP003088.1 [5389:5416]	etB_PM4	AP003088.1[5438:5460]
etD		exfoliative toxin D		etD_11	AB057421.1 [5648:5677]	etD_51	AB057421.1[5694:5715:r]
far1	*fusB*	fusidic acid resistence		far1_10	AY047358.1 [1787:1814]	far1_11_PM4	AY047358.1[1818:1838:r]
fexA		chloramphenicol/florfenicol exporter		hp_fexA_611	AJ549214.1 [332:357]	lb_fexA_651_rv	AJ549214.1[364:382:r]
fib		fibrinogen binding protein (19 kDa)	*fib*	hp_fib_611	CP000046.1 [1177103:1177127]	lb_fib_651_rv	CP000046.1[1177131:1177148:r]
			fib (MRSA252)	hp_fib_612	BX571856.1 [1178081:1178105]		
fnbA		fibronectin-binding protein A	*fnbA (cons)*	hp_fnbA_615	CP000046.1 [2570812:2570840:r]	lb_fnbA_652_rv	CP000046.1[2571555:2571576]
			fnbA (COL)	hp_fnbA_612	CP000046.1 [2571598:2571624:r]	lb_fnbA_655_rv	CP000046.1[2570764:2570784]
			fnbA (MRSA252)	hp_fnbA_613	BX571856.1 [2662314:2662342:r]	lb_fnbA_653_rv	BX571856.1[2662290:2662308]
			fnbA (Mu50+MW2)	hp_fnbA_611	BA000017.4 [2644424:2644451:r]	lb_fnbA_651_rv	BA000017.4[2644388:2644409]
			fnbA (RF122)	hp_fnbA_614	AJ938182.1 [2510055:2510084:r]	lb_fnbA_654_rv	AJ938182.1[2510030:2510048]
						lb_fnbA_656_rv	AM076033.1[1356:1379:r]
fnbB		fibronectin-binding protein B	*fnbB (COL)*	hp_fnbB_614	CP000046.1 [2567853:2567879:r]	lb_fnbB_657_rv	CP000046.1[2567809:2567829]
			fnbB (COL+Mu50+MW2)	hp_fnbB_616	CP000046.1 [2567182:2567212:r]	lb_fnbB_658_rv	CP000046.1[2567156:2567173]
			fnbB (Mu50)	hp_fnbB_611	BA000017.4 [2640460:2640489:r]	lb_fnbB_653_rv	BA000017.4[2640421:2640441]
			fnbB (MW2)	hp_fnbB_613	BA000033.2 [2578791:2578820:r]	lb_fnbB_654_rv	BA000017.4[2640538:2640560]
			fnbB (ST15)	hp_fnbB_612	AM076087.1 [758:783]	lb_fnbB_656_rv	AM076068.1[893:912:r]
			fnbB (ST45-2)	hp_fnbB_615	AM076078.1 [866:893]	lb_fnbB_651_rv	AM076087.1[905:925:r]
						lb_fnbB_652_rv	AM076079.1[914:933:r]
						lb_fnbB_655_rv	AM076078.1[900:920:r]
fosB		metallothiol transferase	*fosB*	hp_fosB_611	CP000046.1 [2389191:2389221]	lb_fosB_651_rv	CP000046.1[2389252:2389271:r]
			fosB-plasmid	hp_fosB_612	AP006717.1 [448:478]	lb_fosB_652_rv	AP006717.1[508:527:r]
gapA		glyceraldehyde 3-phosphate dehydrogenase, locus 1		gapA_11	CP000046.1 [865778:865806]	gapA_51	CP000046.1[865816:865836:r]

Gene	Synonyma	Gene product /function	Alleles	Probe name	Probe definition	Primer name	Primer definition
hl		putative membrane protein		hl_11	CP000046.1 [927983:928011]	hl_51	CP000046.1[928034:928052:r]
hla		haemolysin alpha		hla_11	CP000046.1 [1180134:1180163:r]	hla_51	CP000046.1[1180098:1180120]
hlb		haemolysin beta		hp_hlb_611	CP000046.1 [2063898:2063922]	hlb_51	CP000046.1[2063925:2063944:r]
				hp_hlb_612	BA000017.4 [2126171:2126196]		
				hp_hlb_613	S72497.1 [366:390]		
			hlb, un-disrupted	hlb_11	CP000046.1 [2063880:2063906]		
				hlb_12	S72497.1 [347:374]		
hld		haemolysin delta		hld_11	CP000046.1 [2082840:2082864:r]	hld_51	CP000046.1[2082797:2082819]
hlgA		haemolysin gamma, component A		hlgA_11	CP000046.1 [2479145:2479171]	hlgA_51	CP000046.1[2479172:2479193:r]
hlIII		putative membrane protein	*hlIII-consensus*	hp_hlIII_611	CP000046.1 [2239419:2239444:r]	lb_hlIII_651_rv	CP000046.1[2239376:2239397]
			hlIII- other than RF122	hl-III_11	CP000046.1 [2239828:2239856:r]	hl-III_51	CP000046.1[2239783:2239805]
hsdS1		type I site-specific deoxyribonuclease subunit, 1st locus	*hsdS1 (RF122)*	hp_hsdS-RF122-1_611	AJ938182.1 [317663:317689]	lb_hsdS-RF122-1_651_rv	AJ938182.1[317702:317723:r]
hsdS2		type I site-specific deoxyribonuclease subunit, 2nd locus	*hsdS2 (ST5+ST8)*	hp_hsdS-COL-1_611	CP000046.1 [478949:478977]	lb_hsdS-COL-1_651_rv	CP000046.1[478988:479006:r]
			hsdS2 (MW2+476)	hp_hsdS-MW2-1_611	BX571857.1 [441641:441667]	lb_hsdS-MW2-1_651_rv	BA000033.2[443035:443058:r]
			hsdS2 (RF122)	hp_hsdS-RF122-2_611	AJ938182.1 [422326:422351]	lb_hsdS-RF122-2_651_rv	AJ938182.1[422376:422398:r]
			hsdS2 (MRSA252)	hp_hsdS-MRSA252-1_611	BX571856.1 [463045:463073]	lb_hsdS-MRSA252-1_651_rv	BX571856.1[463099:463120:r]
hsdS3		type I site-specific deoxyribonuclease subunit, 3rd locus	*hsdS3 (Other Than RF122+MRSA252)*	hp_hsdS-CC25_611	CP000046.1 [1913615:1913643:r]	lb_hsdS-COL-2_651_rv	CP000046.1[1913581:1913598]
			hsdS3 (ST8+ST1+RF122)	hp_hsdS-COL-2_611	DQ309452.1 [57:85]		
			hsdS3 (Mu50+N315)	hp_hsdS-Mu50-2_611	BA000017.4 [1935888:1935914:r]	lb_hsdS-Mu50-2_651_rv	BA000017.4[1935844:1935865]
			hsdS3 (CC51+252)	hp_hsdS-CC51_611	BX571856.1 [1983689:1983715:r]	lb_hsdS-CC51_651_rv	BX571856.1[1983667:1983686]
			hsdS3 (MRSA252)	hp_hsdS-MRSA252-2_611	BX571856.1 [1983034:1983063:r]	lb_hsdS-MRSA252-2_651_rv	BX571856.1[1983003:1983023]
hsdSx		type I site-specific deoxyribonuclease subunit, unknown locus	*hsdSx (CC25)*	hp_hsdS-CC25_612	CP000046.1 [1914582:1914609:r]	lb_hsdS-CC25_651_rv	CP000046.1[1914550:1914567]
			hsdSx (CC15)	hp_hsdS-CC15_611	DQ309450.1 [976:1000]	lb_hsdS-CC15_651_rv	DQ309450.1[1009:1031:r]
			hsdSx (etd)	hp_hsdS-etd_611	AB057421.1 [2572:2598:r]	lb_hsdS-etd_651_rv	AB057421.1[2543:2565]
hysA1/2		hyaluronate lyase, first / second locus	*hysA1 (MRSA252)*	hp_hysA_613	BX571856.1 [1975471:1975495]	lb_hysA_652_rv	BX571856.1[1975386:1975408:r]
			hysA1 (MRSA252+RF122) and/or hysA2 (cons)	hp_hysA_614	CP000046.1 [2275950:2275980]	lb_hysA_651_rv	CP000046.1[2275984:2276004:r]
			hysA1 (MRSA252+RF122) / hysA2 (COL+USA300)	hp_hysA_615	BX571856.1 [1975353:1975381]		
hysA2		hyaluronate lyase, second locus	*hysA2 (Other Than MRSA252)*	hp_hysA_611	CP000046.1 [2274647:2274673]	lb_hysA_653_rv	CP000046.1[2274574:2274597:r]
			hysA2 (COL+USA300+NCTC8325)	hp_hysA_617	CP000046.1 [2274542:2274572]	lb_hysA_654_rv	CP000046.1[2274687:2274705:r]
			hysA2 (Other Than COL+USA300+NCTC8325)	hp_hysA_616	BX571856.1 [2376035:2376064]		
			hysA2 (OtherThan COL+USA300+NCTC8325)	hp_hysA_618	BA000017.4 [2343407:2343437]		
			hysA2 (MRSA252)	hp_hysA_612	BX571856.1 [2376142:2376170]		
icaA		intercellular adhesion protein A		hp_icaA_611	CP000046.1 [2764366:2764391]	lb_icaA_651_rv	CP000046.1[2764412:2764432:r]
icaC		intercellular adhesion protein C		hp_icaC_611	CP000046.1 [2766355:2766384]	lb_icaC_651_rv	CP000046.1[2766392:2766410:r]
icaD		biofilm PIA synthesis protein D		hp_icaD_611	CP000046.1 [2764671:2764700]	lb_icaD_651_rv	CP000046.1[2764728:2764750:r]
isaB		immunodominant antigen B	*isaB*	hp_isaB_611	CP000046.1 [2722864:2722888:r]	lb_isaB_651_rv	CP000046.1[2722835:2722853]
			isaB-MRSA252	hp_isaB_612	BX571856.1 [2813801:2813828:r]	lb_isaB_652_rv	BX571856.1[2813775:2813793]
isdA		transferrin-binding protein	*isdA (cons)*	hp_isdA_611	CP000046.1 [1148523:1148547:r]	lb_isdA_651_rv	CP000046.1[1148500:1148518]
			isdA (MRSA252)	hp_isdA_612	BX571856.1 [1149423:1149447:r]	lb_isdA_653_rv	BX571856.1[1149379:1149401]
			isdA (Other Than MRSA252)	hp_isdA_614	CP000046.1 [1148394:1148422:r]	lb_isdA_652_rv	CP000046.1[1148360:1148382]
						lb_isdA_654_rv	AY175448.1[298:320:r]
katA		katalase A		katA_11	CP000046.1 [1374794:1374818]	katA_PM4	CP000046.1[1374825:1374845]

Gene	Synonyma	Gene product /function	Alleles	Probe name	Probe definition	Primer name	Primer definition
kdpA		potassium-translocating ATPase A, chain 2		hp_kdpA-SCC_611	BA000017.4 [77596:77622]	lb_kdpA-SCC_651_rv	BA000017.4[77655:77675:r]
						lb_kdpA-SCC_652_rv	BA000017.4[77890:77910:r]
kdpB		potassium-transporting ATPase B, chain 1		hp_kdpB-SCC_611	BA000017.4 [79736:79763]	lb_kdpB-SCC_651_rv	BA000017.4[79776:79795:r]
kdpC		potassium-translocating ATPase C, chain 2		hp_kdpC-SCC_612	BA000017.4 [81035:81061:r]	lb_kdpC-SCC_651_rv	BA000017.4[80962:80980:r]
						lb_kdpC-SCC_652_rv	BA000017.4[81011:81031]
kdpD		sensor kinase protein		hp_kdpD-SCC_611	BA000017.4 [76370:76397:r]	lb_kdpD-SCC_651_rv	BA000017.4[76331:76349]
kdpE		KDP operon transcriptional regulatory protein		hp_kdpE-SCC_611	BA000017.4 [73744:73769:r]	lb_kdpE-SCC_651_rv	BA000017.4[73717:73735]
lmrP		hypothetical protein, similar to integral membrane protein LmrP	*lmrP (OtherThanRF122)*	hp_lmrP_611	CP000046.1 [181497:181522]	lb_lmrP_651_rv	CP000046.1[181530:181547:r]
			lmrP (OtherThanRF122)	hp_lmrP_613	CP000046.1 [182184:182210]	lb_lmrP_653_rv	CP000046.1[182214:182234:r]
			lmrP (RF122)	hp_lmrP_612	AJ938182.1 [140620:140646]	lb_lmrP_652_rv	AJ938182.1[140655:140672:r]
			lmrP (RF122)	hp_lmrP_614	AJ938182.1 [141308:141333]	lb_lmrP_654_rv	AJ938182.1[141338:141358:r]
lnu **(A)**	*linA, lin(A)*	lincosaminid-nucleotidyltransferase		linA_19,2	J03947.1 [866:890]	linA_51	J03947.1[1049:1069:r]
				linA_19,3	J03947.1 [938:962]	linA_PM4	J03947.1[1036:1053]
lukD		leukocidin D component		lukD_11	CP000046.1 [1934731:1934760:r]	lukD_51	CP000046.1[1934686:1934706]
lukE		leukocidin E component		lukE_11	CP000046.1 [1935944:1935968:r]	lukE_51	CP000046.1[1935901:1935920]
lukF-hlg		haemolysin gamma / leukocidin, component B		lukF_10	CP000046.1 [2481634:2481659]	lukF_11b_PM4	CP000046.1[2481663:2481680:r]
lukS-hlg		haemolysin gamma / leukocidin, component C	*lukS*	lukS_10	CP000046.1 [2480644:2480668]	lukS_11_PM4	CP000046.1[2480672:2480692:r]
			lukS-ST45	hp_lukS-ST45_611	EF672356.1[663:686]		EF672356.1[690:710:r]
							EF672356.1[690:711:r]
lukF-PV		Panton Valentine leukocidin F component		lukF_PV_10	AB006796.1 [2256:2284]	lukF-PV_11_PM4	AB006796.1[2295:2316:r]
lukS-PV		Panton Valentine leukocidin S component		lukS_PV_20	AB006796.1 [1628:1656]	lukS-PV_21_PM4	AB006796.1[1679:1699:r]
lukF-PV83		F component from hypothetical leukocidin from ruminants		lukF-PV-P83_11	AB044554.1 [42010:42037]	lukF-PV-P83_51	AB044554.1[42053:42070:r]
lukM		S component from hypothetical leukocidin from ruminants		lukM_11	AB044554.1 [40866:40893]	lukM_51	AB044554.1[40914:40932:r]
"lukX"	*SAV2004*	leukocidin/haemolysin toxin family protein		lukX_11	CP000046.1 [2065056:2065080:r]	lukX_51	CP000046.1[2065011:2065033]
"lukY"	*SAV2005*	leukocidin/haemolysin toxin family protein	*lukY*	lukY-var1_11	CP000046.1 [2066795:2066824:r]	lukY-var2_51	CP000046.1[2066757:2066777]
			lukY-MRSA252	lukY-var2_11	BX571856.1 [2171414:2171443:r]		
map		Major histocompatibility complex class II analog protein (=Extracellular adherence protein, eap)	*map (COL)*	hp_map_611	CP000046.1 [2063009:2063037:r]	lb_map_652_rv	CP000046.1[2062965:2062984]
			map (MRSA252)	hp_map_613	BX571856.1 [2123661:2123688:r]	lb_map_651_rv	BX571856.1[2123618:2123636]
			map (Mu50+MW2)	hp_map_612	BA000017.4 [2082228:2082252:r]	lb_map_653_rv	BA000017.4[2082185:2082206]
mecA		penicillin binding protein 2, betalactam resistance defining MRSA		mecA_1,4	CP000046.1 [39915:39942:r]	mecA_PM4	CP000046.1[39857:39876:r]
				mecA_11	CP000046.1 [40041:40068:r]	mecA_51	CP000046.1[40007:40025]
mecI		meticillin-resistance regulatory protein		hp_mecI_611	BA000017.4 [49133:49162]	lb_mecI_651_rv	BA000017.4[49169:49190:r]
mecR1	*mecR*	signal transducer protein MecR1	*mecR1 / delta mecR1*	hp_mecR_611	CP000046.1 [41853:41882]	lb_mecR_651_rv	CP000046.1[41885:41906:r]
			mecR1 , un-truncated only, as in SCC*mec* II, III	hp_mecR_612	BA000017.4 [48685:48711]	lb_mecR_652_rv	BA000017.4[48726:48745:r]
mef **(A)**	*mefA, mefE*	macrolide efflux protein A		hp_mefA_611	AB011259.1 [536:563]	lb_mefA_651_rv	AB011259.1[570:588:r]
				hp_mefA_612	AB011259.1 [1045:1072]	lb_mefA_652_rv	AB011259.1[1078:1099:r]
merA		mercury reductase		hp_merA_611	AB179623.1 [2351:2379:r]	lb_merA_651_rv	AB037671.1[39315:39334]
merB		mercuric resistance operon regulatory protein		hp_merB_611	AB179623.1 [1018:1043:r]	lb_merB_651_rv	AB037671.1[38000:38018]
mph(C)	*mpbBM, mph BM, mph(BM)*	probable lysylphosphatidylglycerol synthetase		hp_mpbBM_611	AB013298.1 [2664:2693]	lb_mpbBM_651_rv	AB013298.1[2700:2720:r]
				hp_mpbBM_612	AB013298.1 [2896:2924]	lb_mpbBM_652_rv	AB013298.1[2929:2947:r]
mprF		defensin resistance protein	*mprF (COL+MW2)*	hp_mprF_611	CP000046.1 [1407488:1407518]	lb_mprF_651_rv	CP000046.1[1407519:1407542:r]
			mprF (Mu50+252)	hp_mprF_612	BA000017.4 [1442171:1442201]		
msr(A)	*msrA*	energy-dependent efflux of erythromycin		msrA_15,3	AB013298.1 [1525:1552]	msrA_PM4	AB013298.1[1614:1635]
mupA	*mupR, ileS*	mupirocin resistence protein		mupR_13,2	X75439.1 [1504:1531]	mupR_PM4	X75439.1[1623:1642]
nuc1		thermostable extracellular nuclease		hp_nuc1_611	CP000046.1 [888207:888233]	lb_nuc1_651_rv	CP000046.1[888249:888266:r]

Gene	Synonyma	Gene product /function	Alleles	Probe name	Probe definition	Primer name	Primer definition
ORF CM14		enterotoxin-like protein ORF CM14		hp_entCM14_611	AJ938182.1 [37154:37182]	entCM14_51	U10927.2[32900:32918:r]
				hp_entCM14_612	AJ938182.1 [37532:37557]	lb_entCM14_651_rv	AJ938182.1[37591:37610:r]
pls-SCC		plasmin-sensitive surface protein		hp_plsSCC_611	CP000046.1 [57330:57354]	lb_plsSCC_651_rv	CP000046.1[57378:57398:r]
Q2FXC0		hypothetical protein, located next to serine protease operon		hp_Q2FXC0_611	CP000046.1 [1922667:1922692]	lb_Q2FXC0_651_rv	CP000046.1[1922702:1922721:r]
Q2YUB3		Unspecific efflux/transporter		hp_Q2YUB3_611	AJ938182.1 [2026944:2026969:r]	lb_Q2YUB3_651_rv	AJ938182.1[2026920:2026937]
Q6GD50		hypothetical protein associated with fusidic acid resistance		hp_Q6GD50_611	AF411935.1 [423:452:r]	lb_Q6GD50_651_rv	AF411935.1[372:390]
Q7A4X2		hypothetical protein		hp_Q7A4X2_611	BA000017.4 [1952955:1952984]	lb_Q7A4X2_651_rv	BA000017.4[1953003:1953025:r]
qacA		quaternary ammonium compound resistance protein A		hp_qacA_611	AF053771.1 [976:1004]	qacA_PM4	AF053771.1[2361:2383]
						lb_qacA_651_rv	AB255366.1[19153:19173:r]
						lb_qacA_652_rv	AB255366.1[20475:20498:r]
qacC		quaternary ammonium compound resistance protein C	*qacC (cons)*	hp_qacC_611	AB125342.1 [2382:2411]	lb_qacC_651_rv	AB125342.1[2431:2450:r]
			qacC (equine)	hp_qacC_614	AJ512814.1 [1518:1545]	lb_qacC_653_rv	AJ512814.1[1567:1590:r]
			qacC (SA5)	hp_qacC_613	U81980.1 [2017:2043]	lb_qacC_654_rv	U81980.1[2065:2086:r]
			qacC (Ssap)	hp_qacC_612	Y16945.1 [1951:1981]	lb_qacC_652_rv	AE016833.1[8848:8869:r]
			qacC (ST94)	hp_qacC_615	Y16944.1 [1622:1649]	lb_qacC_655_rv	Y16944.1[1692:1714:r]
rrn STAU		Ribosomal sequence from S. aureus (genusspecific positive control)		s_aur_rrn_1PM4	CP000046.1 [1979941:1979966:r]	saur_rrn_1_6_PM4	CP000046.1[1979903:1979921:r]
						saur_rrn_1_7_PM4	CP000046.1[1979866:1979882:r]
saeS		histidine protein kinase, sae locus		hp_saeS_611	CP000046.1 [788443:788471:r]	lb_saeS_651_rv	CP000046.1[788396:788417]
						lb_saeS_652_rv	CP000046.1[787918:787939]
sak		staphylokinase		hp_sak_611	BA000017.4 [2086572:2086601:r]	lb_sak_651_rv	BA000017.4[2086553:2086571]
				sak_11	BA000017.4 [2086418:2086443:r]	sak_51	BA000017.4[2086376:2086395]
sarA		staphylococcal accessory regulator A		hp_sarA_611	CP000046.1 [700076:700102:r]	lb_sarA_651_rv	CP000046.1[700049:700066]
sasG		Staphylococcus aureus surface protein G	*sasG (COL+Mu50)*	hp_sasG_613	CP000046.1 [2562368:2562396:r]	lb_sasG_651_rv	CP000046.1[2562772:2562794]
			sasG (MW2)	hp_sasG_612	BX571857.1 [2552889:2552918:r]	lb_sasG_652_rv	CP000046.1[2562337:2562358]
			sasG (OtherThan MRSA252+RF122)	hp_sasG_611	CP000046.1 [2562815:2562842:r]	lb_sasG_653_rv	BA000033.2[2573526:2573543]
sat		streptothricine-acetyltransferase		sat_17,2	U51474.1 [393:421]	sat_PM4	U51474.1[488:505]
				sat_17,3	U51474.1 [429:456]		
sbi		IgG-binding protein		sbi-var1_11	CP000046.1 [2476904:2476929]	sbi-var1_51	CP000046.1[2476963:2476982:r]
				sbi-var1_12	CP000046.1 [2477142:2477171]	sbi-var1_52	CP000046.1[2477188:2477210:r]
scn		Staphylococcal complement inhibitor (SCIN)		hp_scn_611	BA000017.4 [2084397:2084425:r]	lb_scn_651_rv	BA000017.4[2084367:2084387]
sdrC		Ser-Asp rich fibrinogen-/bone sialoprotein-binding protein C	*sdrC (cons)*	hp_sdrC_613	CP000046.1 [633257:633283]	lb_sdrC_651_rv	CP000046.1[632900:632919:r]
			sdrC (B1)	hp_sdrC_612	AM076155.1 [1009:1036]	lb_sdrC_652_rv	AM076155.1[1039:1061:r]
			sdrC (COL)	hp_sdrC_615	CP000046.1 [633866:633892]	lb_sdrC_653_rv	CP000046.1[633304:633322:r]
			sdrC (Mu50)	hp_sdrC_614	BA000017.4 [630748:630774]	lb_sdrC_655_rv	CP000046.1[633915:633933:r]
			sdrC (MW2+MRSA252+RF122)	hp_sdrC_616	BX571856.1 [617689:617717]	lb_sdrC_654_rv	BX571856.1[617128:617145:r]
			sdrC (Other Than MRSA252+RF122)	hp_sdrC_611	CP000046.1 [632858:632886]		
sdrD		Ser-Asp rich fibrinogen-/bone sialoprotein-binding protein D	*sdrD (cons)*	hp_sdrD_614	CP000046.1 [637654:637681]	lb_sdrD_652_rv	CP000046.1[637025:637046:r]
			sdrD (COL+MW2)	hp_sdrD_612	CP000046.1 [636995:637020]	lb_sdrD_654_rv	CP000046.1[637691:637712:r]
			sdrD (Mu50)	hp_sdrD_613	BA000017.4 [633896:633924]	lb_sdrD_655_rv	AM076208.1[854:875:r]
			sdrD (other)	hp_sdrD_611	AM076221.1 [157:186]	lb_sdrD_653_rv	BA000017.4[633928:633948:r]
						lb_sdrD_651_rv	AM076221.1[195:212:r]
sea	*entA, entP, sep*	enterotoxin A	*sea*	entA_3,2	BA000017.4 [2088572:2088598:r]	entA-var1_51	BA000017.4[2088473:2088492]
				entA_3,3	BA000017.4 [2088482:2088456]		
				entA-var1_11	BA000017.4 [2088512:2088536:r]		
			sea-320E (entA-320E)	entA-var2_11	AY196686.1 [508:532]		
			sea-N315 (entP, sep)	entA-var3_11	BA000018.3 [2011518:2011545:r]	entA-var3_51	BA000018.3[2011492:2011510]

Gene	Synonyma	Gene product /function	Alleles	Probe name	Probe definition	Primer name	Primer definition
seb	*entB*	enterotoxin B		entB_11	CP000046.1 [916903:916927]	entB_51	CP000046.1[916957:916976:r]
				entB_4,1	CP000046.1 [916583:916609]	entB-41_PM4	CP000046.1[916642:916663]
sec	*entC*	enterotoxin C		entC_5,2	BA000017.4 [2134733:2134761:r]	entC_PM4	BA000017.4[2134593:2134610:r]
				entC_5,3	BA000017.4 [2134652:2134680:r]		
sed	*entD*	enterotoxin D		entD_11	M94872.1 [675:702]	entD_51	M94872.1[705:723:r]
see	*entE*	enterotoxin E		entE_11	AY518387.1 [305:328]	entE_51	AY518387.1[348:365:r]
seg	*entG, selg*	enterotoxin G		entG_11	BA000017.4 [1954500:1954526:r]	entG_51	BA000017.4[1954468:1954486]
seh	*entH*	enterotoxin H		entH_11	AB060536.1 [139:164]	entH_51	AB060536.1[178:196:r]
sei	*entI, seli*	enterotoxin I		entI_11	BA000017.4 [1957319:1957343:r]	entI_51	BA000017.4[1957273:1957293]
sej	*entJ*	enterotoxin J		entJ_11	AB075606.1 [1849:1876:r]	entJ_51	AB075606.1[1804:1823]
sek	*entK*	enterotoxin K		hp_entK_611	CP000046.1 [905353:905384:r]	entK_PM4	CP000046.1[904957:904979:r]
				hp_entK_612	CP000046.1 [905035:905065:r]	lb_entK_651_rv	CP000046.1[905311:905334]
						lb_entK_652_rv	CP000046.1[904995:905017]
						lb_entK_653_rv	BA000033.2[2087412:2087433:r]
sel	*entL*	enterotoxin L		entL_11	BA000017.4 [2134144:2134171]	entL_51	BA000017.4[2134182:2134202:r]
sem	*entM, selm*	enterotoxin M		entM_11	BA000017.4 [1958262:1958291:r]	entM_51	BA000017.4[1958242:1958260]
						entM_52	BX571856.1[1999451:1999468]
sen	*entN, seln*	enterotoxin N	*sen- other than RF122*	entN_11	BA000017.4 [1955521:1955545:r]	entN_51	BA000017.4[1955492:1955513]
			sen-consensus	hp_entN_611	BA000017.4 [1955741:1955768:r]	lb_entN_651_rv	BA000017.4[1955709:1955731]
seo	*entO, selo*	enterotoxin O		entO_11	BA000017.4 [1958936:1958962:r]	entO_51	BA000017.4[1958904:1958925]
seq	*entQ*	enterotoxin Q		hp_entQ_611	CP000046.1 [906043:906072:r]	entQ_PM4	CP000046.1[905715:905734:r]
				hp_entQ_612	CP000046.1 [905818:905848:r]	lb_entQ_651_rv	CP000046.1[905997:906018]
						lb_entQ_652_rv	CP000046.1[905862:905881]
ser	*entR*	enterotoxin R		entR_11	AB075606.1 [750:775]	entR_51	AB075606.1[783:802:r]
"setB1"		staphylococcal exotoxin-like protein, second locus	*setB1*	setB-SA1178_11	CP000046.1 [1182940:1182967:r]	setB-SA1178_51	CP000046.1[1182916:1182934]
			setB1-MRSA252	setB-SAR1139_11	BX571856.1 [1185070:1185094:r]	setB-SAR1139_51	BX571856.1[1185043:1185061]
"setB2"		staphylococcal exotoxin-like protein, second locus	*setB2*	setB-SA1179_11	CP000046.1 [1183750:1183777:r]	setB-SA1179_51	CP000046.1[1183717:1183736]
			setB2-MRSA252	setB-SAR1140_11	BX571856.1 [1185785:1185812:r]	setB-SAR1140_51	BX571856.1[1185730:1185749]
"setB3"	*MW0345*	staphylococcal exotoxin-like protein, second locus	*setB3*	setB-SA1180_11	CP000046.1 [1184568:1184596:r]	setB-SA1180	CP000046.1[1184540:1184558]
"setC"		staphylococcal exotoxin-like protein		setC-MW0345_11	CP000046.1 [446394:446420]	setC-MW0345_51	CP000046.1[446441:446462:r]
seu / sey		Enterotoxin U and/or Y		hp_entU_611	BA000017.4 [1956679:1956708:r]	lb_entU_651_rv	BA000017.4[1956633:1956653]
spa		Protein A		proteinA_12	CP000046.1 [107407:107435:r]	proteinA_51	CP000046.1[107900:107917]
						proteinA_52	CP000046.1[107378:107395]
splA		Serinprotease A		splA_11	CP000046.1 [1921021:1921046:r]	splA_51	CP000046.1[1920987:1921005]
splB		Serinprotease B		splB_11	CP000046.1 [1920101:1920126:r]	splB_51	CP000046.1[1920073:1920090]
splE		Serinprotease E		hp_splE_611	CP000046.1 [1917590:1917613:r]	lb_splE_651_rv	CP000046.1[1917552:1917572]

Gene	Synonyma	Gene product /function	Alleles	Probe name	Probe definition	Primer name	Primer definition
ssl01	*set6, set16*	staphylococcal superantigen-like protein 1	*set6-COL* (SACOL468): probe 1_11+probe 4_11	set6-var1_11	CP000046.1 [470653:470680]	set6-var1_51	CP000046.1[470687:470707:r]
			set6-Mu50 (SAV0422): probe 1_11+probe 1_12/ 4_11	set6-var1_12	BA000017.4 [467359:467382]	set6-var1_52	CP000046.1[470893:470911:r]
			set6-MW2 (MW0382): probe 2_11+probe 2_12	set6-var2_11	BX571856.1 [452856:452881]	set6-var2_51	BX571856.1[452890:452909:r]
			SAR0422 (MRSA 252): probe 2_11+probe 1_12	set6-var2_12	BA000033.2 [429731:429757]		
				set6-var4_11	CP000046.1 [470856:470879]		
			ssl01-RF122	hp_ssl01_611	AJ938182.1 [412017:412045]	lb_ssl01_651_rv	AJ938182.1[412058:412076:r]
ssl02	*set7, set17*	staphylococcal superantigen-like protein 2	*ssl02*	set7-var1_11	CP000046.1 [471582:471608]	set7-var1_51	CP000046.1[471646:471665:r]
			ssl02-MRSA252	set7-var2_11	BX571856.1 [453788:453814]		
ssl03	*set8, set18*	staphylococcal superantigen-like protein 3	*ssl03*	set8_11	CP000046.1 [472456:472481]	set8_51	CP000046.1[472502:472520:r]
				hp_ssl03_611	AJ938182.1 [413800:413827]		
			ssl03-MRSA252	set-SAR0424_11	BX571856.1 [454824:454850]		
ssl04	*set9, set19*	staphylococcal superantigen-like protein 4	*ssl04*	set9-var1_11	CP000046.1 [474254:474283]	set9-var1_51	CP000046.1[474328:474349:r]
				set9-var1_12	CP000046.1 [474511:474537]	set9-var1_52	CP000046.1[474556:474575:r]
			ssl04-MRSA252	set-SAR0425_11	BX571856.1 [456040:456064]	set-SAR0425_51	BX571856.1[454869:454886:r]
				set-SAR0425_12	BX571856.1 [455386:455412]	set-SAR0425_52	BX571856.1[456703:456721:r]
ssl05	*set3, set20*	staphylococcal superantigen-like protein 5	*ssl05*	set3-var1_11	BA000017.4 [470660:470688]	set3-var1_51	BA000017.4[470703:470720:r]
				hp_ssl05_612	BA000017.4 [470300:470327]	lb_ssl05_652_rv	BA000017.4[470338:470359:r]
			ssl05-MRSA252	set3-var2_11	BX571856.1 [457227:457253]	set3-var2_51	BX571856.1[457263:457284:r]
			ssl05-RF122	hp_ssl05_611	AJ938182.1 [415143:415170]	lb_ssl05_651_rv	CP000253.1[393062:393084:r]
ssl06	*set21*	staphylococcal superantigen-like protein 6		set21_11	BA000033.2 [435210:435237]	set21_51	BA000033.2[435244:435264:r]
				hp_ssl06_611	BX571857.1 [433755:433784]	lb_ssl06_651_rv	BA000033.2[435132:435153:r]
						lb_ssl06_652_rv	CP000253.1[394288:394308:r]
ssl07	*set1, set22*	staphylococcal superantigen-like protein 7	*ssl07*	set1-var4_11	BA000017.4 [471560:471589]	set1-var1_51	BA000017.4[471600:471617:r]
			ssl07-MRSA252	set1-var1_11	BX571856.1 [458486:458515]		
			ssl07-FRI326	set1-var2_11	AF188836.1 [165:194]		
ssl08	*set12, set23*	staphylococcal superantigen-like protein 8	*ssl08*	set12_11	BA000017.4 [472594:472621]	set12_51	BA000017.4[472624:472644:r]
				hp_ssl08_611	AJ938182.1 [417435:417464]	lb_ssl08_651_rv	AJ938182.1[417465:417488:r]
ssl09	*set5, set24*	staphylococcal superantigen-like protein 9	*ssl09*	set5-var1_11	CP000046.1 [475126:475152]	set5-var1_51	CP000046.1[475171:475191:r]
				hp_ssl09_611	CP000046.1 [475125:475151]		
			ssl09-MRSA252	set5-var2_11	BX571856.1 [459446:459472]	set5-var2_51	BX571856.1[459498:459519:r]
ssl10	*set4, set25*	staphylococcal superantigen-like protein 10	*ssl10*	set4-var1_11	CP000046.1 [476429:476457]	set4-var1_51	CP000046.1[476474:476491:r]
			ssl10-MRSA252	hp_ssl10_611	AJ938182.1 [419736:419765]		
			ssl10-RF122	set4-var2_11	BX571856.1 [460746:460772]	set4-var2_51	BX571856.1[460783:460800:r]
ssl11	*set2, set26*	staphylococcal superantigene-like protein 11	*ssl11 (COL)*	set2-var4_11	CP000046.1 [480340:480368]	set2-var4_51	CP000046.1[480377:480395:r]
			ssl11 (Mu50+N315)	set2-var3_11	BA000017.4 [478916:478945]	set2-var3_51	BA000017.4[478947:478967:r]
			ssl11 (MW2+MSSA476)	set2-var1_11	BA000033.2 [443788:443816]	set2-var1_51	BA000033.2[443857:443879:r]
			ssl11 (MRSA252)	set2-var2_11	BX571856.1 [464936:464964]	set2-var2_51	BX571856.1[464979:464999:r]
sspA		glutamylendopeptidase		hp_sspA_611	CP000046.1 [1063762:1063789:r]	lb_sspA_651_rv	CP000046.1[1063730:1063748]
						lb_sspA_653_rv	CP000046.1[1063034:1063053]
						lb_sspA_652_rv	BA000017.4[1097992:1098012]
sspB		staphopain B, Protease		hp_sspB_611	CP000046.1 [1062809:1062834:r]	lb_sspB_651_rv	CP000046.1[1062777:1062797]
				hp_sspB_612	CP000046.1 [1062120:1062149:r]	lb_sspB_652_rv	CP000046.1[1062098:1062116]
sspP		staphopain A (staphylopain A), Protease		hp_sspP_611	CP000046.1 [2034882:2034908]	lb_sspP_651_rv	CP000046.1[2034918:2034936:r]
			sspP (other than ST93)	hp_sspP_612	CP000046.1 [2035388:2035416]	lb_sspP_652_rv	CP000046.1[2035421:2035442:r]

Gene	Synonyma	Gene product /function	Alleles	Probe name	Probe definition	Primer name	Primer definition
"tetEfflux"		Transport-/Effluxprotein		hp_tetEfflux_611	CP000046.1 [2238561:2238587:r]	lb_tetEfflux_651_rv	CP000046.1[2238525:2238546]
						lb_tetEfflux_652_rv	BX571856.1[2342013:2342032]
tet(K)	*tetK*	tetrazyklin-resistance		tetK_12,3	M16217.1 [1424:1452]	tetK_PM4	M16217.1[1582:1604:r]
				tetK_12,4	M16217.1 [1507:1535]		
tet(M)	*tetM*	tetrazyklin-resistance		tetM_11,3	BA000017.4 [440374:440400:r]	tetM_51	BA000017.4[440340:440358]
tst1		toxic shock syndrome toxin 1	*tst1 (other than RF122)*	tst1_16,2	BA000017.4 [2137855:2137879]	tst1_PM4	BA000017.4[2137910:2137930]
			tst1 (consensus)	hp_tst_611	BA000017.4 [2138016:2138044]	lb_tst_651_rv	BA000017.4[2138051:2138070:r]
ugpQ		glycerophosphoryl diester phosphodiesterase, associated with mecA		hp_ugpQ_611	CP000046.1 [38665:38690]	lb_ugpQ_651_rv	CP000046.1[38701:38719:r]
vanA		vancomycin resistance gene		vanA_18,2	AE017171.1 [35199:35226]	vanA_PM4	AE017171.1[35302:35321]
vanB		vancomycin resistance gene from enterococci and Clostridium		vanB_11	AE016954.1 [78616:78640:r]	vanB_PM4	AE016954.1[78518:78535:r]
				vanB_19,3	AE016954.1 [78613:78638:r]		
vanZ		teicoplanin resistance gene from enterococci		vanZ_20,3	AE017171.1 [37564:37589]	vanZ_PM4	AE017171.1[37613:37632]
***vat* (A)**		virginiamycin A acetyltransferase		vatA_15,3	AF117258.1 [2296:2323:r]	vatA_PM4	AF117258.1[2229:2247:r]
***vat* (B)**		acetyltransferase inactivating streptogramin A		vatB_16,3	U19459.1 [543:570]	vatB_PM4	U19459.1[659:680]
***vga* (A)**	*vga*	ATP binding protein, streptogramin-A-resistance	*vga* (A)	vga_17,3	AF117259.1 [3729:3756]	vga_PM4	AF117259.1[3779:3799]
			vga (A)*-BM 3327*	vgaA_18,3	AF186237.2 [6470:6497]	vgaA_PM4	AF117259.1[3925:3944]
***vgb* (A)**		virginiamycin B hydrolase		vgb_19,2	M36022.1 [1040:1067]	vgb_PM4	M36022.1[1126:1148]
vraS		sensor protein		hp_vraS_612	CP000046.1 [2005634:2005659:r]	lb_vraS_651_rv	CP000046.1[2005818:2005835]
						lb_vraS_652_rv	CP000046.1[2005605:2005624]
vwb		van Willebrand factor binding protein	*vwb (cons)*	hp_vwb_615	CP000046.1 [884859:884884]	lb_vwb_651_rv	CP000046.1[883877:883899:r]
			vwb (COL+MW2)	hp_vwb_612	CP000046.1 [883836:883864]	lb_vwb_656_rv	CP000046.1[884895:884914:r]
			vwb (MRSA252)	hp_vwb_613	BX571856.1 [891899:891927]	lb_vwb_653_rv	BX571856.1[891952:891972:r]
			vwb (Mu50)	hp_vwb_614	BA000017.4 [890919:890947]	lb_vwb_654_rv	BA000017.4[890960:890980:r]
						lb_vwb_655_rv	BA000017.4[891393:891412:r]
			vwb (RF122)	hp_vwb_611	AJ938182.1 [821019:821046]	lb_vwb_652_rv	AJ938182.1[821064:821083:r]
xylR		homolog of xylose repressor, associated with SCCmec-elements		hp_xylR_611	BA000017.4 [50113:50141]	lb_xylR_651_rv	BA000017.4[50152:50171:r]
