# Supplementary material for: A Field Guide to Pandemic, Epidemic and Sporadic Clones of Methicillin-Resistant Staphylococcus aureus
Source: PLoS One. 2011 Apr 6;6(4):e17936. doi: 10.1371/journal.pone.0017936 (PMC3071808; doi:10.1371/journal.pone.0017936)
Supplement: File S2 — Overview of sequence types (STs), spa types, some characteristic genomic markers and fully sequenced genomes of the clonal complexes (CCs) described in this study (bold typeset indicates STs and spa types identified in the present study; italic typeset indicates STs found by the authors in MSSA isolates or spa types described in literature or public databases such as RIDOM; *, see text for further explanation). (PDF) [file pone.0017936.s002.pdf]

Clonal complex	Sequence types	*Spa* types	Characteristic genomic markers	Full genome sequences
**CC1**	**ST1, ST872, ST1005, ST1115, ST1336.**<br>(ST573/772 are discussed separately)	**t127**, **t128**, *t174, t175, t176*, **t386**, *t558*	*agr* III,<br>capsule type 8,<br>*seh, cna, sasG*	MW2/USA400 (BA00003),<br>MSSA 476 (BX571857)
**CC5**	**ST5, ST73, ST111, ST125, ST149, ST221, ST225, ST228, ST496, ST526, ST575, ST835**, **ST930, ST998**	**t001**, **t002**, **t003**, *t010*, **t023**, **t041**, **t045**, *t053*, **t062**, **t067**, **t088**, **t105**, **t110**, **t143**, **t149**, **t303**, **t311**, **t442**, **t447**, **t458**, **t548**, **t570**, **t627**, **t688**, **t777**, **t811**, **t837**, **t892**, **t893**, **t1290**, **t4065**	*agr* II,<br>capsule type 5,<br>*egc, sasG*	Mu50 (BA000017),<br>Mu3 (AP009324),<br>N315 (BA000018),<br>JH1 (CP000736),<br>JH9 (CP000703)<br>04-02981 (MSSA, CP001844)
**ST6**		**t701**	*agr* I,<br>capsule type 8,<br>*cna, sasG*	-
**CC7**	*ST7*<br>(ST1048 is discussed separately)	*t091*	*agr* I,<br>capsule type 8	-
**CC8**	**ST8, ST94, ST247, ST250, ST254, ST576, ST609, ST612, ST985, ST995, ST1337**<br>(ST72, ST239-241 are discussed separately)	**t008, t009, t018, t024, t036, t052, t064, t068, t121, t190, t194, t211, t334,** *t400*, **t451, t4691,** *t1029*, **t1677, t2047,** *t2169*, **t2196, t2206, t2238,t2514, t3503, t4268, t4306**	*agr* I,<br>capsule type 5,<br>*sasG*	COL (CP000046)<br>USA300-FPR3757 (CP000255)<br>USA300-TCH1516 (CP000730)<br>NCTC8325 (MSSA, CP000253)<br>Newman (MSSA, AP009351)
**CC9**	*ST9*<br>(ST584/733/834 are discussed separately)	*t100, t411, t899, t4358*	*agr* II,<br>capsule type 5,<br>*egc*	-
**CC12**	**ST12**	*t156*, **t160**	*agr* II,<br>capsule type 8<br>ORF CM14, *cna*	-
CC15	*ST15, ST582*	*t084, t094, t360*	*agr* II,<br>capsule type 8,<br>*sasG*	-
**CC20**	*ST20*	*t148, t195, t1023*	agr I,<br>capsule type 5,<br>*egc, sasG,*	-
**CC22**	**ST22,** *ST1117*	**t005, t016, t020, t022, t032, t223, t309, t432, t451, t515, t531, t717, t981, t891, t1214, t1370, t1802, t1865, t1977, t2480, t2951, t3185, t3501, t3505, t5711**	*agr* I,<br>capsule type 5,<br>*egc, cna, sasG.*	EMRSA15 (finished genome at: http://www.sanger.ac.uk/resources/downloads/bacteria/staphylococcus-aureus.html)
**CC30**	**ST30, ST36, ST39, ST982**	**t007, t012,** *t017*, **t018, t019, t021, t253, t300,** *t318*, **t419, t924, t5074**	*agr* III,<br>capsule type 8,<br>*egc, cna*	MRSA252 (BX571856)
**CC45**	*ST45*	**t004, t015, t040,** *t116,* **t123, t727, t750, t1081, t1575,** *t1608*, **t2135,** *t2714*	*agr* I or *agr* IV*<br>capsule type 8,<br>*egc**, *cna*, (*sasG**)	-
**CC59**	**ST59, ST87, ST359, ST952**	**t216, t316, t437, t441, t528, t976,** *t1151*, **t1950, t2365**	*agr* I,<br>capsule type 8<br>*sasG*	-
**ST72**		**t791**	*agr* I,<br>capsule type 5<br>*egc, sasG*	-
**CC75**	**ST75, ST1304, ST883, ST1303**	novel (**259-31-17-17-17-23-17-17-23-17-22**) for ST75-MRSA-IV<br>novel (**259-23-23-17-17-17-23-23-23-17-16**) for ST883-MRSA-IV	atypical *agr* allele,<br>no capsule genes detected,<br>*egc* (in CC75, ST1303),<br>*sasG* (in CC75)	-
**CC80**	**ST80, ST583, ST728**	**t044, t131, t416**	*agr* III,<br>capsule type 8,<br>*sasG*	-
**CC88**	**ST78, ST88, ST129, ST255, ST257**	**t186, t690,** *t693, t729, t730*, **t786, t1598, t3205**	*agr* III,<br>capsule type 8,<br>*sasG*	-
**ST93**		**t202**	*agr* III,<br>capsule type 8<br>ORF CM14	JKD6159 (CP002114)

Clonal complex	Sequence types	*Spa* types	Characteristic genomic markers	Full genome sequences
**CC97**	**ST97, ST953**	*t044, t131,* **t1234**	*agr* I,<br>capsule type 5<br>*sasG*	-
**CC121**	*ST121*, **ST577**	*t159, t284, t314, t435, t518, t850*, **t3025**	*agr* IV<br>capsule type 8<br>ORF CM14, *egc, cna*	-
**CC152**	**ST152,** *ST377, ST1633*	**t355**	*agr* I,<br>capsule type 5<br>*cna*	-
**ST154**		**t667**	*agr* III,<br>capsule type 8,<br>*cna, sasG*	-
**CC188**	**ST188**	**t189**	*agr* I,<br>capsule type 8,<br>*cna*	-
**ST239**	(including *ST240, ST241*)	**t030, t037, t363, t1155**	*agr* I,<br>capsule type 8,<br>*cna, sasG*	TW20 (FN433596)<br>JKD6008 (CP002120)
**CC361**	**ST361, ST672**	**t315, t1309**	*agr* I,<br>capsule type 8,<br>*egc, sasG*	-
**CC398**	**ST398**	**t011, t034,** *t567*, **t571, t1197, t1250, t1451, t1456, t2510**	agr I,<br>capsule type 5,<br>*cna*	S0385 (AM990992)
ST426		*t271, t536*	*agr* I,<br>capsule type 8,<br>ORF CM14, *cna**	-
**CC509**	**ST207**	**t375**	*agr* III,<br>capsule type 8.<br>*egc**, *cna, sasG*	-
**ST573 and ST772**		**t657, t1839, t3387, t5073**	agr II,<br>capsule type 5,<br>ORF CM14, *egc, cna, sasG*	-
**ST779**		**t878**	*agr* III,<br>capsule type 5,<br>*sasG*	-
**ST834**		**t3029,** novel (**15-17-20-17-12-17-16**)	*agr* I,<br>capsule type 8<br>*sasG*	-
**CC913**	*ST912*, **ST913**, *ST914*	**t991**	*agr* II,<br>capsule type 8<br>*sasG*	-
**ST1048**		**t1081**	*agr* I,<br>capsule type 8,<br>*egc**, *cna, sasG*	-
**ST1774**		**t1081**	*agr* I,<br>capsule type 8,<br>*cna*	-
