# Supplementary material for: A Field Guide to Pandemic, Epidemic and Sporadic Clones of Methicillin-Resistant Staphylococcus aureus
Source: PLoS One. 2011 Apr 6;6(4):e17936. doi: 10.1371/journal.pone.0017936 (PMC3071808; doi:10.1371/journal.pone.0017936)
Supplement: File S3 — Complete hybridisation results for MRSA strains examined in this study. (PDF) [file pone.0017936.s003.pdf]

	number of isolates tested	SPECIES MARKER							REGULATORY GENES							
		rrn STAU	gapA	katA	CoA	nuc1	spa	sbi	sarA	saeS	vraS	agrI (total)	agrII (total)	agrIII (total)	agrIV (total)	hld
CC1-MRSA-IV [PVL-], WA-1/57	50	POS	POS	POS	POS	POS	POS	POS	POS	POS	POS	NEG	NEG	POS	NEG	POS
CC1-MRSA-IV [PVL+], USA400	19	POS	POS	POS	POS	POS	POS	POS	POS	POS	POS	NEG	NEG	POS	NEG	POS
CC1-MRSA-IV/SCCfus [PVL-]	24	POS	POS	POS	POS	POS	POS	POS	POS	POS	POS	NEG	NEG	POS	NEG	POS
CC1-MRSA-IV/SCCfus [PVL+]	2	POS	POS	POS	AMB	POS	POS	POS	POS	POS	POS	NEG	NEG	AMB	NEG	POS
CC1-MRSA-V [PVL+]	1	POS	POS	POS	POS	POS	POS	POS	POS	POS	POS	NEG	NEG	POS	NEG	POS
CC1-MRSA-V/SCCfus [PVL-]	3	POS	POS	POS	POS	POS	POS	POS	POS	POS	POS	NEG	NEG	POS	NEG	POS
CC1-MRSA-V/SCCfus [PVL+]	1	POS	POS	POS	POS	POS	POS	POS	POS	POS	POS	NEG	NEG	POS	NEG	POS
ST228-MRSA-I, South German/Italian MRSA	156	POS	POS	POS	POS	POS	POS	POS	POS	POS	POS	NEG	POS	NEG	NEG	POS
ST5-MRSA-I, Geraldine Clone	22	POS	POS	POS	POS	POS	POS	POS	POS	POS	POS	NEG	POS	NEG	NEG	POS
CC5-MRSA-Ivar., WA-18/21/48	8	POS	POS	POS	POS	POS	POS	POS	POS	POS	POS	NEG	POS	NEG	NEG	POS
CC5-MRSA-II, Rhine-Hesse /UK-3	310	POS	POS	POS	POS	POS	POS	POS	POS	POS	POS	NEG	POS	NEG	NEG	POS
CC5-MRSA-II [ACME+]	3	POS	POS	POS	POS	POS	POS	POS	POS	POS	POS	NEG	POS	NEG	NEG	POS
CC5-MRSA-III	6	POS	POS	POS	POS	POS	POS	POS	POS	POS	POS	NEG	POS	NEG	NEG	POS
CC5-MRSA-IV, Paediatric clone	104	POS	POS	POS	POS	POS	POS	POS	POS	POS	POS	NEG	POS	NEG	NEG	POS
CC5-MRSA-IV [PVL+]	31	POS	POS	POS	POS	POS	POS	POS	POS	POS	POS	NEG	POS	NEG	NEG	POS
CC5-MRSA-V	27	POS	POS	POS	POS	POS	POS	POS	POS	POS	POS	NEG	POS	NEG	NEG	POS
CC5-MRSA-V [PVL+]	3	POS	POS	POS	POS	POS	POS	POS	POS	POS	POS	NEG	POS	NEG	NEG	POS
CC5-MRSA-VI, New Paediatric Clone	18	POS	POS	POS	POS	POS	POS	POS	POS	POS	POS	NEG	POS	NEG	NEG	POS
ST5-MRSA-VII (SCC-JCSC6082)	1	POS	POS	POS	AMB	POS	POS	POS	POS	POS	POS	NEG	POS	NEG	NEG	POS
CC5-MRSA-IVvar, Maltese Clone	15	POS	POS	POS	POS	POS	POS	POS	POS	POS	POS	NEG	POS	NEG	NEG	POS
CC5-MRSA-SCC(MRSAZH47)	1	POS	POS	POS	POS	POS	POS	POS	POS	POS	POS	NEG	POS	NEG	NEG	POS
CC5-MRSA-IV/VI	29	POS	POS	POS	POS	POS	POS	POS	POS	POS	POS	NEG	POS	NEG	NEG	POS
CC5-MRSA-IV/SCCfus	1	POS	POS	POS	POS	POS	POS	POS	POS	POS	POS	NEG	POS	NEG	NEG	POS
CC5/ST835-MRSA, WA-40/46	4	POS	POS	POS	POS	POS	POS	POS	POS	POS	POS	NEG	POS	NEG	NEG	POS
ST6-MRSA-IV, WA-51	4	POS	POS	POS	POS	POS	POS	POS	POS	POS	POS	POS	NEG	NEG	NEG	POS
ST6-MRSA-V	2	POS	POS	POS	POS	POS	POS	POS	POS	POS	POS	POS	NEG	NEG	AMB	POS
CC7-MRSA-IV	1	POS	POS	POS	POS	POS	POS	POS	POS	POS	POS	POS	NEG	NEG	NEG	POS
CC7-MRSA-V	1	POS	POS	POS	POS	POS	POS	POS	POS	POS	POS	POS	NEG	NEG	NEG	POS
ST250-MRSA-I, Early/Ancestral MRSA	17	POS	POS	POS	POS	POS	POS	POS	POS	POS	POS	POS	NEG	NEG	NEG	POS
ST247-MRSA-I, North German/Iberian MRSA	14	POS	POS	POS	POS	POS	POS	POS	POS	POS	POS	POS	NEG	NEG	NEG	POS
CC8-MRSA-IV, UK-14/WA-5	35	POS	POS	POS	POS	POS	POS	POS	POS	POS	POS	POS	NEG	NEG	NEG	POS
CC8-MRSA-IV, Lyon Clone/UK-2	130	POS	POS	POS	POS	POS	POS	POS	POS	POS	POS	POS	NEG	NEG	NEG	POS
CC8-MRSA-IV, UK-6	5	POS	POS	POS	POS	POS	POS	POS	POS	POS	POS	POS	NEG	NEG	NEG	POS
CC8-MRSA-IV, USA500	28	POS	POS	POS	POS	POS	POS	POS	POS	POS	POS	POS	NEG	NEG	NEG	POS
ST8-MRSA-IV [PVL/ACME+], USA300	117	POS	POS	POS	POS	POS	POS	POS	POS	POS	POS	POS	NEG	NEG	NEG	POS
ST8-MRSA-IV [PVL+], USA300	52	POS	POS	POS	POS	POS	POS	POS	POS	POS	POS	POS	NEG	NEG	NEG	POS
CC8-MRSA-IV [PVL/sed/j/r/k/q+]	7	POS	POS	POS	POS	POS	POS	POS	POS	POS	POS	POS	NEG	NEG	AMB	POS
CC8-MRSA-IV [tst1/sec/sel+]	3	POS	POS	POS	POS	POS	POS	POS	POS	POS	POS	POS	NEG	NEG	AMB	POS
CC8-MRSA-V	4	POS	POS	POS	POS	POS	POS	POS	POS	POS	POS	POS	NEG	NEG	NEG	POS
CC8-MRSA-VIII	2	POS	POS	POS	POS	POS	POS	POS	POS	POS	POS	POS	NEG	NEG	NEG	POS
ST8-MRSA-IIA/B/C/D/E, Irish AR13/14	49	POS	POS	POS	POS	POS	POS	POS	POS	POS	POS	POS	NEG	NEG	NEG	POS
ST8-MRSA, UK-12/13, Irish AR43	20	POS	POS	POS	POS	POS	POS	POS	POS	POS	POS	POS	NEG	NEG	NEG	POS
ST254-MRSA, Hannover EMRSA	19	POS	POS	POS	POS	POS	POS	POS	POS	POS	POS	POS	NEG	NEG	NEG	POS
ST254-MRSA-IV/V, Hannover/UK-10	5	POS	POS	POS	POS	POS	POS	POS	POS	POS	POS	POS	NEG	NEG	NEG	POS
CC9-MRSA-IV	5	POS	POS	POS	POS	POS	POS	POS	POS	POS	POS	NEG	POS	NEG	NEG	POS
CC12-MRSA, WA-59	1	POS	POS	POS	POS	POS	POS	POS	POS	POS	POS	NEG	POS	NEG	NEG	POS
CC12-MRSA-IV	1	POS	POS	POS	POS	POS	POS	POS	POS	POS	POS	NEG	POS	NEG	NEG	POS
CC20-MRSA-V	1	POS	POS	POS	POS	POS	POS	POS	POS	POS	POS	POS	NEG	NEG	NEG	POS
ST22-MRSA-IV, Barnim/UK-15	543	POS	POS	POS	POS	POS	POS	POS	POS	POS	POS	POS	NEG	NEG	NEG	POS
ST22-MRSA-IV [ACME+]	23	POS	POS	POS	POS	POS	POS	POS	POS	POS	POS	POS	NEG	NEG	AMB	POS
ST22-MRSA-IV [PVL+]	15	POS	POS	POS	POS	POS	POS	POS	POS	POS	POS	POS	NEG	NEG	AMB	POS
ST22-MRSA-V	2	POS	POS	POS	POS	POS	POS	POS	POS	POS	POS	POS	NEG	NEG	NEG	POS
ST22-MRSA-V [PVL+]	1	POS	POS	POS	POS	POS	POS	POS	POS	POS	POS	POS	NEG	NEG	AMB	POS
ST36-MRSA-II, UK-16	43	POS	POS	POS	POS	POS	POS	POS	POS	POS	POS	NEG	NEG	POS	NEG	POS
ST30-MRSA-IV [PVL+], Southwest Pacific Clone	40	POS	POS	POS	POS	POS	POS	POS	POS	POS	POS	NEG	NEG	POS	NEG	POS
ST30-MRSA-IV [tst1+]	9	POS	POS	POS	POS	POS	POS	POS	POS	POS	POS	NEG	NEG	POS	NEG	POS
CC30-MRSA-V [PVL+]	1	POS	POS	POS	POS	POS	POS	POS	POS	POS	POS	NEG	NEG	POS	NEG	POS
ST45-MRSA-II, USA600	4	POS	POS	POS	POS	POS	POS	POS	POS	POS	POS	POS	NEG	NEG	NEG	POS
ST45-MRSA-IV, Berlin EMRSA	150	POS	POS	POS	POS	POS	POS	POS	POS	POS	POS	POS	NEG	NEG	NEG	POS
CC45-MRSA-IV [ACME+]	3	POS	POS	POS	POS	POS	POS	POS	POS	POS	POS	POS	NEG	NEG	NEG	POS
ST45-MRSA-V, WA-4	13	POS	POS	POS	POS	POS	POS	POS	POS	POS	POS	POS	NEG	NEG	NEG	POS
CC45/agr IV-MRSA-IV, WA-23	10	POS	POS	POS	POS	POS	POS	POS	POS	POS	POS	AMB	NEG	NEG	POS	POS
CC45/agr IV-MRSA-V	40	POS	POS	POS	POS	POS	POS	POS	POS	POS	POS	NEG	NEG	NEG	POS	POS
CC45/agr IV-MRSA-IV/V	3	POS	POS	POS	POS	POS	POS	POS	POS	POS	POS	NEG	NEG	NEG	POS	POS
CC59-MRSA-IV [PVL+], USA1000	5	POS	POS	POS	POS	POS	POS	POS	POS	POS	POS	POS	NEG	NEG	NEG	POS
ST59-MRSA-IV, WA-73	2	POS	POS	POS	POS	POS	POS	POS	POS	POS	POS	POS	NEG	NEG	NEG	POS
ST87-MRSA-IV, WA-24	4	POS	POS	POS	POS	POS	POS	POS	POS	POS	POS	POS	NEG	NEG	AMB	POS
ST59-MRSA-IV [PVL+], WA-55/56	10	POS	POS	POS	POS	POS	POS	POS	POS	POS	POS	POS	NEG	NEG	AMB	POS
ST59/ST952-MRSA-V(T) [PVL+], Taiwan Clone	42	POS	POS	POS	POS	POS	POS	POS	POS	POS	POS	POS	NEG	NEG	AMB	POS
ST59-MRSA-V	4	POS	POS	POS	POS	POS	POS	POS	POS	POS	POS	POS	NEG	NEG	AMB	POS
CC59-MRSA-V [PVL+]	2	POS	POS	POS	POS	POS	POS	POS	POS	POS	POS	POS	NEG	NEG	NEG	POS
ST59-MRSA-IV/V, WA-15	9	POS	POS	POS	POS	POS	POS	POS	POS	POS	POS	POS	NEG	NEG	NEG	POS
ST72-MRSA-IV, USA700	12	POS	POS	POS	POS	POS	POS	POS	POS	POS	POS	POS	NEG	NEG	NEG	POS
ST72-MRSA-IV [PVL+], WA-44	4	POS	POS	POS	POS	POS	POS	POS	POS	POS	POS	POS	NEG	NEG	AMB	POS
CC75-MRSA-IV	19	POS	POS	AMB	NEG	POS	NEG	AMB	POS	POS	POS	NEG	NEG	NEG	NEG	POS
ST883-MRSA-IV, WA-47	3	POS	POS	AMB	NEG	AMB	POS	POS	POS	POS	POS	NEG	NEG	NEG	NEG	POS
ST1303-MRSA-IV	1	POS	POS	NEG	NEG	POS	NEG	AMB	POS	POS	POS	NEG	NEG	AMB	NEG	POS
CC80-MRSA-IV	4	POS	POS	POS	POS	POS	POS	POS	POS	POS	POS	NEG	NEG	POS	NEG	POS
CC80-MRSA-IV [PVL+], European Clone	112	POS	POS	POS	POS	POS	POS	POS	POS	POS	POS	NEG	NEG	POS	NEG	POS
CC88-MRSA-IV, WA-2	60	POS	POS	POS	POS	POS	POS	POS	POS	POS	POS	NEG	NEG	POS	NEG	POS
CC88-MRSA-IV [PVL+]	11	POS	POS	POS	POS	POS	POS	POS	POS	POS	POS	NEG	NEG	POS	NEG	POS
CC88-MRSA-V [PVL+]	1	POS	POS	POS	POS	POS	POS	POS	POS	POS	POS	NEG	NEG	POS	NEG	POS
CC88-MRSA-VI	1	POS	POS	POS	POS	POS	POS	POS	POS	POS	POS	NEG	NEG	POS	NEG	POS
ST93-MRSA-IV [PVL-]	2	POS	POS	POS	POS	POS	POS	AMB	POS	POS	POS	NEG	NEG	POS	NEG	POS
ST93-MRSA-IV [PVL+], Queensland Clone	47	POS	POS	POS	POS	POS	POS	POS	POS	POS	POS	NEG	NEG	POS	NEG	POS
ST93-MRSA-V [PVL+]	1	POS	POS	POS	POS	POS	POS	POS	POS	POS	POS	NEG	NEG	POS	NEG	POS
CC97-MRSA-IV, WA-54	5	POS	POS	POS	POS	POS	POS	POS	POS	POS	POS	POS	NEG	NEG	NEG	POS
CC97-MRSA-V	1	POS	POS	POS	POS	POS	POS	POS	POS	POS	POS	POS	NEG	NEG	NEG	POS
CC97-MRSA-V [ACME+]	2	POS	POS	POS	POS	POS	POS	POS	POS	POS	POS	POS	NEG	NEG	AMB	POS
CC97-MRSA-I/V	2	POS	POS	POS	POS	POS	POS	POS	POS	POS	POS	POS	NEG	NEG	AMB	POS
CC121-MRSA-V, WA-22	1	POS	POS	POS	POS	POS	POS	POS	POS	POS	POS	NEG	NEG	NEG	POS	POS
CC152-MRSA-V [PVL+]	7	POS	POS	POS	POS	POS	POS	AMB	POS	POS	POS	POS	NEG	NEG	AMB	POS
ST154-MRSA [PVL+]	2	POS	POS	POS	POS	POS	POS	POS	POS	POS	POS	NEG	NEG	POS	NEG	POS
ST188-MRSA-IV, WA-38/78	2	POS	POS	POS	POS	POS	POS	POS	POS	POS	POS	POS	NEG	NEG	NEG	POS
ST188-MRSA-V	1	POS	POS	POS	POS	POS	POS	POS	POS	POS	POS	POS	NEG	NEG	AMB	POS
ST239-MRSA-III	204	POS	POS	POS	POS	POS	POS	POS	POS	POS	POS	POS	NEG	NEG	NEG	POS
CC361-MRSA-IV, WA-29	3	POS	POS	POS	POS	POS	POS	POS	POS	POS	POS	POS	NEG	NEG	NEG	POS
CC361-MRSA-V	1	POS	POS	POS	POS	POS	POS	POS	POS	POS	POS	POS	NEG	NEG	NEG	POS
CC361-MRSA-VIII, WA-28	1	POS	POS	POS	POS	POS	POS	POS	POS	POS	POS	POS	NEG	NEG	NEG	POS
CC398-MRSA-IV	18	POS	POS	POS	POS	POS	POS	POS	POS	POS	POS	POS	NEG	NEG	NEG	POS
ST398-MRSA-V	228	POS	POS	POS	POS	POS	POS	POS	POS	POS	POS	POS	NEG	NEG	NEG	POS
ST398-MRSA-V [PVL+]	2	POS	POS	POS	POS	POS	POS	POS	POS	POS	POS	POS	NEG	NEG	NEG	POS
CC509/ST207-MRSA-V	1	POS	POS	POS	POS	POS	POS	POS	POS	POS	POS	NEG	NEG	POS	NEG	POS
ST573-MRSA-V, WA-10	1	POS	POS	POS	POS	POS	POS	POS	POS	POS	POS	NEG	POS	NEG	NEG	POS
ST772-MRSA-V [PVL+], WA-60/Bengal Bay Clone	9	POS	POS	POS	POS	POS	POS	POS	POS	POS	POS	NEG	POS	NEG	NEG	POS
ST779-MRSA	4	POS	POS	POS	POS	POS	POS	POS	POS	POS	POS	NEG	NEG	POS	NEG	POS
ST834-MRSA-IV, WA-13	3	POS	POS	POS	POS	POS	POS	POS	POS	POS	POS	POS	NEG	NEG	NEG	POS
CC913-MRSA-IV	1	POS	POS	POS	POS	POS	POS	POS	POS	POS	POS	NEG	POS	NEG	NEG	POS
ST1774-MRSA-IV [ACME+]	21	POS	POS	POS	POS	POS	POS	POS	POS	POS	POS	POS	NEG	NEG	NEG	POS
CC15-MSSA	247	POS	POS	POS	POS	POS	POS	POS	POS	POS	POS	NEG	POS	NEG	NEG	POS
ST426-MSSA	12	POS	POS	POS	POS	NEG	POS	POS	POS	POS	POS	POS	NEG	NEG	NEG	POS

Legend:

Positive :	POS
Ambiguous :	AMB
Negative :	NEG
Rare (in >0 to 33% of isolates) :	RARE
Variable (in >33 to 66% of isolates) :	VAR
Common (in >66 to <100% of isolates) :	COMM

	METHICILLIN RESISTANCE AND SCCmec TYPING													
	mecA	mecR1 / delta mecR1	ugpQ	ccrA-1	ccrB-1	plsSCC (COL)	Q9XB68-dcs	ccrA-2	ccrB-2	kdpA-SCC	kdpB-SCC	kdpC-SCC	kdpD-SCC	kdpE-SCC
CC1-MRSA-IV [PVL-], WA-1/57	POS	POS	POS	NEG	NEG	NEG	NEG	POS	POS	NEG	NEG	NEG	NEG	NEG
CC1-MRSA-IV [PVL+], USA400	POS	POS	POS	NEG	NEG	NEG	AMB	POS	POS	NEG	NEG	NEG	NEG	NEG
CC1-MRSA-IV/SCCfus [PVL-]	POS	POS	POS	POS	POS	NEG	NEG	POS	POS	NEG	NEG	NEG	NEG	NEG
CC1-MRSA-IV/SCCfus [PVL+]	POS	POS	POS	POS	POS	NEG	NEG	POS	POS	NEG	NEG	NEG	NEG	NEG
CC1-MRSA-V [PVL+]	POS	NEG	POS	NEG	NEG	NEG	NEG	NEG	NEG	NEG	NEG	NEG	NEG	NEG
CC1-MRSA-V/SCCfus [PVL-]	POS	NEG	POS	POS	POS	NEG	NEG	NEG	NEG	NEG	NEG	NEG	NEG	NEG
CC1-MRSA-V/SCCfus [PVL+]	POS	NEG	POS	POS	POS	NEG	NEG	NEG	NEG	NEG	NEG	NEG	NEG	NEG
ST228-MRSA-I, South German/Italian MRSA	POS	POS	POS	POS	POS	POS	AMB	NEG	NEG	NEG	NEG	NEG	NEG	NEG
ST5-MRSA-I, Geraldine Clone	POS	POS	POS	POS	POS	NEG	POS	NEG	NEG	NEG	NEG	NEG	NEG	NEG
CC5-MRSA-Ivar., WA-18/21/48	POS	POS	POS	AMB	POS	NEG	NEG	NEG	NEG	NEG	NEG	NEG	NEG	NEG
CC5-MRSA-II, Rhine-Hesse /UK-3	POS	POS	POS	NEG	NEG	NEG	POS	POS	POS	POS	POS	POS	POS	POS
CC5-MRSA-II [ACME+]	POS	POS	POS	NEG	NEG	NEG	POS	POS	POS	POS	POS	POS	POS	POS
CC5-MRSA-III	POS	POS	POS	NEG	NEG	NEG	POS	NEG	NEG	NEG	NEG	NEG	NEG	NEG
CC5-MRSA-IV, Paediatric clone	POS	POS	POS	NEG	NEG	NEG	AMB	POS	POS	NEG	NEG	NEG	NEG	NEG
CC5-MRSA-IV [PVL+]	POS	POS	POS	NEG	NEG	NEG	AMB	POS	POS	NEG	NEG	NEG	NEG	NEG
CC5-MRSA-V	POS	NEG	POS	NEG	NEG	NEG	NEG	NEG	NEG	NEG	NEG	NEG	NEG	NEG
CC5-MRSA-V [PVL+]	POS	NEG	POS	NEG	NEG	NEG	NEG	NEG	NEG	NEG	NEG	NEG	NEG	NEG
CC5-MRSA-VI, New Paediatric Clone	POS	POS	POS	NEG	NEG	NEG	NEG	NEG	NEG	NEG	NEG	NEG	NEG	NEG
ST5-MRSA-VII (SCC-JCSC6082)	POS	POS	POS	NEG	NEG	NEG	NEG	NEG	NEG	NEG	NEG	NEG	NEG	NEG
CC5-MRSA-IVvar, Maltese Clone	POS	POS	POS	NEG	NEG	NEG	POS	POS	POS	NEG	NEG	NEG	NEG	NEG
CC5-MRSA-SCC(MRSAZH47)	POS	POS	POS	NEG	NEG	NEG	NEG	POS	POS	NEG	NEG	NEG	NEG	NEG
CC5-MRSA-IV/VI	POS	POS	POS	NEG	NEG	NEG	AMB	POS	POS	NEG	NEG	NEG	NEG	NEG
CC5-MRSA-IV/SCCfus	POS	POS	POS	POS	POS	NEG	AMB	POS	POS	NEG	NEG	NEG	NEG	NEG
CC5/ST835-MRSA, WA-40/46	POS	NEG	POS	NEG	NEG	NEG	NEG	POS	POS	NEG	NEG	NEG	NEG	NEG
ST6-MRSA-IV, WA-51	POS	POS	POS	NEG	NEG	NEG	AMB	POS	POS	NEG	NEG	NEG	NEG	NEG
ST6-MRSA-V	POS	NEG	POS	NEG	NEG	NEG	NEG	NEG	NEG	NEG	NEG	NEG	NEG	NEG
CC7-MRSA-IV	POS	POS	POS	NEG	NEG	NEG	POS	POS	POS	NEG	NEG	NEG	NEG	NEG
CC7-MRSA-V	POS	NEG	POS	NEG	NEG	NEG	NEG	NEG	NEG	NEG	NEG	NEG	NEG	NEG
ST250-MRSA-I, Early/Ancestral MRSA	POS	POS	POS	POS	POS	VAR	POS	NEG	NEG	NEG	NEG	NEG	NEG	NEG
ST247-MRSA-I, North German/Iberian MRSA	POS	POS	POS	POS	POS	POS	POS	NEG	NEG	NEG	NEG	NEG	NEG	NEG
CC8-MRSA-IV, UK-14/WA-5	POS	POS	POS	NEG	NEG	NEG	POS	POS	POS	NEG	NEG	NEG	NEG	NEG
CC8-MRSA-IV, Lyon Clone/UK-2	POS	POS	POS	NEG	NEG	NEG	AMB	POS	POS	NEG	NEG	NEG	NEG	NEG
CC8-MRSA-IV, UK-6	POS	POS	POS	NEG	NEG	NEG	POS	POS	POS	NEG	NEG	NEG	NEG	NEG
CC8-MRSA-IV, USA500	POS	POS	POS	NEG	NEG	NEG	POS	POS	POS	NEG	NEG	NEG	NEG	NEG
ST8-MRSA-IV [PVL/ACME+], USA300	POS	POS	POS	NEG	NEG	NEG	POS	POS	POS	NEG	NEG	NEG	NEG	NEG
ST8-MRSA-IV [PVL+], USA300	POS	POS	POS	NEG	NEG	NEG	AMB	POS	POS	NEG	NEG	NEG	NEG	NEG
CC8-MRSA-IV [PVL/sed/j/r/k/q+]	POS	POS	POS	NEG	NEG	NEG	POS	POS	POS	NEG	NEG	NEG	NEG	NEG
CC8-MRSA-IV [tst1/sec/sel+]	POS	POS	POS	NEG	NEG	NEG	POS	POS	POS	NEG	NEG	NEG	NEG	NEG
CC8-MRSA-V	POS	NEG	POS	NEG	NEG	NEG	NEG	NEG	NEG	NEG	NEG	NEG	NEG	NEG
CC8-MRSA-VIII	POS	POS	POS	NEG	NEG	NEG	AMB	NEG	NEG	NEG	NEG	NEG	NEG	NEG
ST8-MRSA-IIA/B/C/D/E, Irish AR13/14	POS	POS	POS	NEG	NEG	NEG	AMB	POS	POS	NEG	NEG	NEG	NEG	NEG
ST8-MRSA, UK-12/13, Irish AR43	POS	POS	POS	NEG	NEG	NEG	RARE	VAR	VAR	NEG	NEG	NEG	NEG	NEG
ST254-MRSA, Hannover EMRSA	POS	POS	POS	NEG	NEG	NEG	AMB	NEG	NEG	NEG	NEG	NEG	NEG	NEG
ST254-MRSA-IV/V, Hannover/UK-10	POS	POS	POS	NEG	NEG	NEG	AMB	POS	POS	NEG	NEG	NEG	NEG	NEG
CC9-MRSA-IV	POS	POS	POS	NEG	NEG	NEG	POS	POS	POS	NEG	NEG	NEG	NEG	NEG
CC12-MRSA, WA-59	POS	POS	POS	NEG	NEG	NEG	NEG	NEG	NEG	NEG	NEG	NEG	NEG	NEG
CC12-MRSA-IV	POS	POS	POS	NEG	NEG	NEG	AMB	POS	POS	NEG	NEG	NEG	NEG	NEG
CC20-MRSA-V	POS	NEG	POS	NEG	NEG	NEG	NEG	NEG	NEG	NEG	NEG	NEG	NEG	NEG
ST22-MRSA-IV, Barnim/UK-15	POS	POS	POS	NEG	NEG	NEG	AMB	POS	POS	NEG	NEG	NEG	NEG	NEG
ST22-MRSA-IV [ACME+]	POS	POS	POS	NEG	NEG	NEG	POS	POS	POS	NEG	NEG	NEG	NEG	NEG
ST22-MRSA-IV [PVL+]	POS	POS	POS	NEG	NEG	NEG	POS	POS	POS	NEG	NEG	NEG	NEG	NEG
ST22-MRSA-V	POS	NEG	POS	NEG	NEG	NEG	NEG	NEG	NEG	NEG	NEG	NEG	NEG	NEG
ST22-MRSA-V [PVL+]	POS	NEG	POS	NEG	NEG	NEG	NEG	NEG	NEG	NEG	NEG	NEG	NEG	NEG
ST36-MRSA-II, UK-16	POS	POS	POS	NEG	NEG	NEG	POS	POS	POS	POS	POS	POS	POS	POS
ST30-MRSA-IV [PVL+], Southwest Pacific Clone	POS	POS	POS	NEG	NEG	NEG	POS	POS	POS	NEG	NEG	NEG	NEG	NEG
ST30-MRSA-IV [tst1+]	POS	POS	POS	NEG	NEG	NEG	AMB	POS	POS	NEG	NEG	NEG	NEG	NEG
CC30-MRSA-V [PVL+]	POS	NEG	POS	NEG	NEG	NEG	NEG	NEG	NEG	NEG	NEG	NEG	NEG	NEG
ST45-MRSA-II, USA600	POS	POS	POS	NEG	NEG	NEG	POS	POS	POS	POS	POS	POS	POS	POS
ST45-MRSA-IV, Berlin EMRSA	POS	POS	POS	NEG	NEG	NEG	AMB	POS	POS	NEG	NEG	NEG	NEG	NEG
CC45-MRSA-IV [ACME+]	POS	POS	POS	NEG	NEG	NEG	AMB	POS	POS	NEG	NEG	NEG	NEG	NEG
ST45-MRSA-V, WA-4	POS	NEG	POS	NEG	NEG	NEG	NEG	NEG	NEG	NEG	NEG	NEG	NEG	NEG
CC45/agr IV-MRSA-IV, WA-23	POS	POS	POS	NEG	NEG	NEG	POS	POS	POS	NEG	NEG	NEG	NEG	NEG
CC45/agr IV-MRSA-V	POS	NEG	POS	NEG	NEG	NEG	NEG	NEG	NEG	NEG	NEG	NEG	NEG	NEG
CC45/agr IV-MRSA-IV/V	POS	POS	POS	NEG	NEG	NEG	POS	POS	POS	NEG	NEG	NEG	NEG	NEG
CC59-MRSA-IV [PVL+], USA1000	POS	POS	POS	NEG	NEG	NEG	POS	POS	POS	NEG	NEG	NEG	NEG	NEG
ST59-MRSA-IV, WA-73	POS	POS	POS	NEG	NEG	NEG	AMB	POS	POS	NEG	NEG	NEG	NEG	NEG
ST87-MRSA-IV, WA-24	POS	POS	POS	NEG	NEG	NEG	AMB	POS	POS	NEG	NEG	NEG	NEG	NEG
ST59-MRSA-IV [PVL+], WA-55/56	POS	POS	POS	NEG	NEG	NEG	POS	POS	POS	NEG	NEG	NEG	NEG	NEG
ST59/ST952-MRSA-V(T) [PVL+], Taiwan Clone	POS	NEG	POS	NEG	NEG	NEG	NEG	NEG	NEG	NEG	NEG	NEG	NEG	NEG
ST59-MRSA-V	POS	NEG	POS	NEG	NEG	NEG	NEG	NEG	NEG	NEG	NEG	NEG	NEG	NEG
CC59-MRSA-V [PVL+]	POS	NEG	POS	NEG	NEG	NEG	NEG	NEG	NEG	NEG	NEG	NEG	NEG	NEG
ST59-MRSA-IV/V, WA-15	POS	POS	POS	NEG	NEG	NEG	POS	POS	POS	NEG	NEG	NEG	NEG	NEG
ST72-MRSA-IV, USA700	POS	POS	POS	NEG	NEG	NEG	POS	POS	POS	NEG	NEG	NEG	NEG	NEG
ST72-MRSA-IV [PVL+], WA-44	POS	POS	POS	NEG	NEG	NEG	AMB	POS	POS	NEG	NEG	NEG	NEG	NEG
CC75-MRSA-IV	POS	POS	POS	NEG	NEG	NEG	POS	POS	POS	NEG	NEG	NEG	NEG	NEG
ST883-MRSA-IV, WA-47	POS	POS	POS	NEG	NEG	NEG	AMB	POS	POS	NEG	NEG	NEG	NEG	NEG
ST1303-MRSA-IV	POS	POS	POS	NEG	NEG	NEG	POS	POS	POS	NEG	NEG	NEG	NEG	NEG
CC80-MRSA-IV	POS	POS	POS	NEG	NEG	NEG	POS	AMB	AMB	NEG	NEG	NEG	NEG	NEG
CC80-MRSA-IV [PVL+], European Clone	POS	POS	POS	NEG	NEG	NEG	POS	POS	POS	NEG	NEG	NEG	NEG	NEG
CC88-MRSA-IV, WA-2	POS	POS	POS	NEG	NEG	NEG	NEG	POS	POS	NEG	NEG	NEG	NEG	NEG
CC88-MRSA-IV [PVL+]	POS	POS	POS	NEG	NEG	NEG	POS	POS	POS	NEG	NEG	NEG	NEG	NEG
CC88-MRSA-V [PVL+]	POS	NEG	POS	NEG	NEG	NEG	NEG	NEG	NEG	NEG	NEG	NEG	NEG	NEG
CC88-MRSA-VI	POS	POS	POS	NEG	NEG	NEG	NEG	NEG	NEG	NEG	NEG	NEG	NEG	NEG
ST93-MRSA-IV [PVL-]	POS	POS	POS	NEG	NEG	NEG	AMB	POS	POS	NEG	NEG	NEG	NEG	NEG
ST93-MRSA-IV [PVL+], Queensland Clone	POS	POS	POS	NEG	NEG	NEG	POS	POS	POS	NEG	NEG	NEG	NEG	NEG
ST93-MRSA-V [PVL+]	POS	NEG	POS	NEG	NEG	NEG	NEG	NEG	NEG	NEG	NEG	NEG	NEG	NEG
CC97-MRSA-IV, WA-54	POS	POS	POS	NEG	NEG	NEG	AMB	POS	POS	NEG	NEG	NEG	NEG	NEG
CC97-MRSA-V	POS	NEG	POS	NEG	NEG	NEG	NEG	NEG	NEG	NEG	NEG	NEG	NEG	NEG
CC97-MRSA-V [ACME+]	POS	NEG	POS	NEG	NEG	NEG	NEG	NEG	AMB	NEG	NEG	NEG	NEG	NEG
CC97-MRSA-I/V	POS	NEG	POS	POS	POS	NEG	NEG	NEG	NEG	NEG	NEG	NEG	NEG	NEG
CC121-MRSA-V, WA-22	POS	NEG	POS	NEG	NEG	NEG	NEG	NEG	NEG	NEG	NEG	NEG	NEG	NEG
CC152-MRSA-V [PVL+]	POS	NEG	POS	NEG	NEG	NEG	NEG	NEG	NEG	NEG	NEG	NEG	NEG	NEG
ST154-MRSA [PVL+]	POS	POS	POS	NEG	NEG	NEG	AMB	VAR	VAR	NEG	NEG	NEG	NEG	NEG
ST188-MRSA-IV, WA-38/78	POS	POS	POS	NEG	NEG	NEG	AMB	POS	POS	NEG	NEG	NEG	NEG	NEG
ST188-MRSA-V	POS	NEG	POS	NEG	NEG	NEG	NEG	NEG	NEG	NEG	NEG	NEG	NEG	NEG
ST239-MRSA-III	POS	COMM	POS	NEG	AMB	NEG	NEG	NEG	NEG	NEG	NEG	NEG	NEG	NEG
CC361-MRSA-IV, WA-29	POS	POS	POS	NEG	NEG	NEG	AMB	POS	POS	NEG	NEG	NEG	NEG	NEG
CC361-MRSA-V	POS	NEG	POS	NEG	NEG	NEG	NEG	NEG	NEG	NEG	NEG	NEG	NEG	NEG
CC361-MRSA-VIII, WA-28	POS	POS	POS	NEG	NEG	NEG	NEG	NEG	NEG	NEG	NEG	NEG	NEG	NEG
CC398-MRSA-IV	POS	POS	POS	NEG	NEG	NEG	AMB	POS	POS	NEG	NEG	NEG	NEG	NEG
ST398-MRSA-V	POS	NEG	POS	RARE	NEG	NEG	NEG	NEG	NEG	NEG	NEG	NEG	NEG	NEG
ST398-MRSA-V [PVL+]	POS	NEG	POS	NEG	NEG	NEG	NEG	NEG	NEG	NEG	NEG	NEG	NEG	NEG
CC509/ST207-MRSA-V	POS	NEG	POS	NEG	NEG	NEG	NEG	NEG	NEG	NEG	NEG	NEG	NEG	NEG
ST573-MRSA-V, WA-10	POS	NEG	POS	NEG	NEG	NEG	NEG	NEG	NEG	NEG	NEG	NEG	NEG	NEG
ST772-MRSA-V [PVL+], WA-60/Bengal Bay Clone	POS	NEG	POS	NEG	NEG	NEG	NEG	NEG	NEG	NEG	NEG	NEG	NEG	NEG
ST779-MRSA	POS	NEG	POS	NEG	NEG	NEG	NEG	NEG	NEG	NEG	NEG	NEG	NEG	NEG
ST834-MRSA-IV, WA-13	POS	POS	POS	NEG	NEG	NEG	AMB	POS	POS	NEG	NEG	NEG	NEG	NEG
CC913-MRSA-IV	POS	POS	POS	NEG	NEG	NEG	POS	POS	POS	NEG	NEG	NEG	NEG	NEG
ST1774-MRSA-IV [ACME+]	POS	POS	POS	NEG	NEG	NEG	POS	POS	POS	NEG	NEG	NEG	NEG	NEG

CC15-MSSA	NEG	NEG	NEG	NEG	NEG	NEG	NEG	NEG	NEG	NEG	NEG	NEG	NEG	NEG
ST426-MSSA	NEG	NEG	NEG	NEG	NEG	NEG	NEG	NEG	NEG	NEG	NEG	NEG	NEG	NEG

Legend:

Positive :	POS
Ambiguous :	AMB
Negative :	NEG
Rare (in >0 to 33% of isolates) :	RARE
Variable (in >33 to 66% of isolates) :	VAR
Common (in >66 to <100% of isolates) :	COMM

	METHICILLIN RESISTANCE AND SCCmec TYPING, continued												RESISTANCE : PENICILLINASE		
	mecI	mecR1 (un-truncated only)	xylR	ccrA-3	ccrB-3	merA	merB	ccrAA (85-2082)	ccrAA (MRSAZH47)	ccrC (85-2082)	ccrA-4	ccrB-4	blaZ	blaI	blaR
CC1-MRSA-IV [PVL-], WA-1/57	NEG	NEG	NEG	NEG	NEG	NEG	NEG	NEG	NEG	NEG	NEG	NEG	COMM	COMM	COMM
CC1-MRSA-IV [PVL+], USA400	NEG	NEG	NEG	NEG	NEG	NEG	NEG	NEG	NEG	NEG	NEG	NEG	POS	POS	POS
CC1-MRSA-IV/SCCfus [PVL-]	NEG	NEG	NEG	NEG	NEG	NEG	NEG	NEG	NEG	NEG	NEG	NEG	POS	POS	POS
CC1-MRSA-IV/SCCfus [PVL+]	NEG	NEG	NEG	NEG	AMB	NEG	NEG	n.t.	NEG	NEG	NEG	NEG	POS	POS	POS
CC1-MRSA-V [PVL+]	NEG	NEG	NEG	NEG	NEG	NEG	NEG	n.t.	POS	POS	NEG	NEG	POS	POS	POS
CC1-MRSA-V/SCCfus [PVL-]	NEG	NEG	NEG	NEG	NEG	NEG	NEG	n.t.	POS	POS	NEG	NEG	POS	POS	POS
CC1-MRSA-V/SCCfus [PVL+]	NEG	NEG	NEG	NEG	NEG	NEG	NEG	n.t.	POS	POS	NEG	NEG	POS	POS	POS
ST228-MRSA-I, South German/Italian MRSA	NEG	NEG	NEG	NEG	NEG	COMM	COMM	NEG	NEG	NEG	NEG	NEG	COMM	COMM	COMM
ST5-MRSA-I, Geraldine Clone	NEG	NEG	NEG	NEG	NEG	NEG	NEG	NEG	NEG	NEG	NEG	NEG	COMM	COMM	COMM
CC5-MRSA-Ivar., WA-18/21/48	NEG	NEG	NEG	NEG	NEG	NEG	NEG	NEG	NEG	NEG	NEG	NEG	POS	POS	POS
CC5-MRSA-II, Rhine-Hesse /UK-3	POS	POS	POS	NEG	NEG	RARE	RARE	NEG	NEG	NEG	NEG	NEG	COMM	COMM	COMM
CC5-MRSA-II [ACME+]	POS	POS	POS	NEG	NEG	NEG	NEG	n.t.	NEG	NEG	AMB	POS	VAR	VAR	VAR
CC5-MRSA-III	POS	POS	POS	POS	POS	NEG	NEG	n.t.	NEG	NEG	NEG	NEG	COMM	COMM	COMM
CC5-MRSA-IV, Paediatric clone	NEG	NEG	NEG	NEG	NEG	RARE	RARE	NEG	NEG	NEG	NEG	NEG	COMM	COMM	COMM
CC5-MRSA-IV [PVL+]	NEG	NEG	NEG	NEG	NEG	NEG	NEG	NEG	NEG	NEG	NEG	NEG	COMM	COMM	COMM
CC5-MRSA-V	NEG	NEG	NEG	NEG	NEG	RARE	RARE	AMB	POS	POS	NEG	NEG	COMM	COMM	COMM
CC5-MRSA-V [PVL+]	NEG	NEG	NEG	NEG	NEG	NEG	NEG	n.t.	POS	POS	NEG	NEG	POS	POS	POS
CC5-MRSA-VI, New Paediatric Clone	NEG	NEG	NEG	NEG	NEG	NEG	NEG	NEG	NEG	NEG	AMB	POS	POS	POS	POS
ST5-MRSA-VII (SCC-JCSC6082)	NEG	NEG	NEG	NEG	NEG	NEG	NEG	n.t.	NEG	POS	NEG	NEG	POS	POS	POS
CC5-MRSA-IVvar, Maltese Clone	NEG	NEG	NEG	POS	NEG	NEG	NEG	NEG	NEG	NEG	NEG	NEG	POS	POS	POS
CC5-MRSA-SCC(MRSAZH47)	NEG	NEG	NEG	NEG	NEG	NEG	NEG	n.t.	POS	POS	NEG	NEG	POS	POS	POS
CC5-MRSA-IV/VI	NEG	NEG	NEG	NEG	NEG	NEG	NEG	NEG	NEG	NEG	AMB	POS	COMM	COMM	COMM
CC5-MRSA-IV/SCCfus	NEG	NEG	NEG	NEG	NEG	NEG	NEG	n.t.	NEG	NEG	NEG	NEG	POS	POS	POS
CC5/ST835-MRSA, WA-40/46	NEG	NEG	NEG	NEG	NEG	NEG	NEG	POS	POS	POS	NEG	NEG	COMM	COMM	COMM
ST6-MRSA-IV, WA-51	NEG	NEG	NEG	NEG	NEG	NEG	NEG	NEG	NEG	NEG	NEG	NEG	COMM	COMM	COMM
ST6-MRSA-V	NEG	NEG	NEG	NEG	NEG	NEG	NEG	n.t.	POS	POS	NEG	NEG	VAR	VAR	VAR
CC7-MRSA-IV	NEG	NEG	NEG	NEG	NEG	NEG	NEG	NEG	NEG	NEG	NEG	NEG	POS	POS	POS
CC7-MRSA-V	NEG	NEG	NEG	NEG	NEG	NEG	NEG	NEG	POS	POS	NEG	NEG	POS	POS	POS
ST250-MRSA-I, Early/Ancestral MRSA	NEG	NEG	NEG	NEG	NEG	COMM	COMM	NEG	NEG	NEG	NEG	NEG	COMM	COMM	COMM
ST247-MRSA-I, North German/Iberian MRSA	NEG	NEG	NEG	NEG	NEG	COMM	COMM	NEG	NEG	NEG	NEG	NEG	COMM	COMM	COMM
CC8-MRSA-IV, UK-14/WA-5	NEG	NEG	NEG	NEG	NEG	RARE	RARE	NEG	NEG	NEG	NEG	NEG	COMM	COMM	COMM
CC8-MRSA-IV, Lyon Clone/UK-2	NEG	NEG	NEG	NEG	NEG	RARE	RARE	NEG	NEG	NEG	NEG	NEG	COMM	COMM	COMM
CC8-MRSA-IV, UK-6	NEG	NEG	NEG	NEG	NEG	NEG	NEG	n.t.	NEG	NEG	NEG	NEG	POS	POS	POS
CC8-MRSA-IV, USA500	NEG	NEG	NEG	NEG	NEG	RARE	RARE	NEG	NEG	NEG	NEG	NEG	POS	POS	POS
ST8-MRSA-IV [PVL/ACME+], USA300	NEG	NEG	NEG	NEG	NEG	NEG	NEG	NEG	NEG	NEG	NEG	NEG	COMM	COMM	COMM
ST8-MRSA-IV [PVL+], USA300	NEG	NEG	NEG	NEG	NEG	COMM	COMM	NEG	NEG	NEG	NEG	NEG	COMM	COMM	COMM
CC8-MRSA-IV [PVL/sed/j/r/k/q+]	NEG	NEG	NEG	NEG	NEG	NEG	NEG	n.t.	NEG	NEG	NEG	NEG	POS	POS	POS
CC8-MRSA-IV [tst1/sec/sel+]	NEG	NEG	NEG	NEG	NEG	NEG	NEG	n.t.	NEG	NEG	NEG	NEG	POS	POS	POS
CC8-MRSA-V	NEG	NEG	NEG	NEG	NEG	NEG	NEG	POS	COMM	POS	NEG	NEG	VAR	VAR	VAR
CC8-MRSA-VIII	POS	POS	POS	NEG	NEG	VAR	VAR	NEG	NEG	NEG	AMB	POS	POS	AMB	POS
ST8-MRSA-IIA/B/C/D/E, Irish AR13/14	VAR	POS	VAR	NEG	NEG	COMM	COMM	NEG	NEG	NEG	NEG	POS	COMM	COMM	COMM
ST8-MRSA, UK-12/13, Irish AR43	NEG	NEG	NEG	NEG	NEG	NEG	NEG	NEG	NEG	NEG	POS	POS	POS	POS	POS
ST254-MRSA, Hannover EMRSA	NEG	NEG	NEG	NEG	NEG	COMM	COMM	NEG	NEG	NEG	NEG	NEG	COMM	COMM	COMM
ST254-MRSA-IV/V, Hannover/UK-10	NEG	NEG	NEG	NEG	NEG	RARE	RARE	POS	NEG	POS	NEG	NEG	POS	POS	POS
CC9-MRSA-IV	NEG	NEG	NEG	NEG	NEG	NEG	NEG	n.t.	NEG	NEG	NEG	NEG	POS	POS	POS
CC12-MRSA, WA-59	POS	POS	NEG	NEG	NEG	NEG	NEG	NEG	NEG	NEG	NEG	NEG	NEG	NEG	NEG
CC12-MRSA-IV	NEG	NEG	NEG	NEG	NEG	NEG	NEG	NEG	NEG	NEG	NEG	NEG	POS	POS	POS
CC20-MRSA-V	NEG	NEG	NEG	NEG	NEG	NEG	NEG	POS	POS	POS	NEG	NEG	POS	POS	POS
ST22-MRSA-IV, Barnim/UK-15	NEG	NEG	NEG	NEG	NEG	NEG	NEG	NEG	NEG	NEG	NEG	NEG	COMM	COMM	COMM
ST22-MRSA-IV [ACME+]	NEG	NEG	NEG	NEG	NEG	NEG	NEG	NEG	NEG	NEG	NEG	NEG	COMM	COMM	COMM
ST22-MRSA-IV [PVL+]	NEG	NEG	NEG	NEG	NEG	NEG	NEG	NEG	NEG	NEG	NEG	NEG	COMM	COMM	COMM
ST22-MRSA-V	NEG	NEG	NEG	NEG	NEG	NEG	NEG	n.t.	POS	POS	NEG	NEG	POS	POS	POS
ST22-MRSA-V [PVL+]	NEG	NEG	NEG	NEG	NEG	NEG	NEG	NEG	POS	POS	NEG	NEG	POS	POS	POS
ST36-MRSA-II, UK-16	POS	POS	POS	NEG	NEG	NEG	NEG	NEG	NEG	NEG	NEG	NEG	POS	POS	POS
ST30-MRSA-IV [PVL+], Southwest Pacific Clone	NEG	NEG	NEG	NEG	NEG	NEG	NEG	NEG	NEG	NEG	NEG	NEG	POS	POS	POS
ST30-MRSA-IV [tst1+]	NEG	NEG	NEG	NEG	NEG	NEG	NEG	n.t.	NEG	NEG	NEG	NEG	POS	POS	POS
CC30-MRSA-V [PVL+]	NEG	NEG	NEG	NEG	NEG	NEG	NEG	n.t.	POS	POS	NEG	NEG	POS	POS	POS
ST45-MRSA-II, USA600	POS	POS	POS	NEG	NEG	NEG	NEG		NEG	NEG	NEG	NEG	POS	POS	POS
ST45-MRSA-IV, Berlin EMRSA	NEG	NEG	NEG	NEG	NEG	NEG	NEG	NEG	NEG	NEG	NEG	NEG	COMM	COMM	COMM
CC45-MRSA-IV [ACME+]	NEG	NEG	NEG	NEG	NEG	NEG	NEG	n.t.	NEG	NEG	NEG	NEG	COMM	COMM	COMM
ST45-MRSA-V, WA-4	NEG	NEG	NEG	NEG	NEG	NEG	NEG	POS	POS	POS	NEG	NEG	COMM	COMM	COMM
CC45/agr IV-MRSA-IV, WA-23	NEG	NEG	NEG	NEG	NEG	RARE	RARE	NEG	NEG	NEG	NEG	NEG	POS	POS	POS
CC45/agr IV-MRSA-V	NEG	NEG	NEG	NEG	NEG	NEG	NEG	n.t.	POS	POS	NEG	NEG	COMM	COMM	COMM
CC45/agr IV-MRSA-IV/V	NEG	NEG	NEG	NEG	NEG	NEG	NEG	n.t.	POS	POS	NEG	NEG	POS	POS	POS
CC59-MRSA-IV [PVL+], USA1000	NEG	NEG	NEG	NEG	NEG	NEG	NEG	NEG	NEG	NEG	NEG	NEG	COMM	COMM	COMM
ST59-MRSA-IV, WA-73	NEG	NEG	NEG	NEG	NEG	NEG	NEG	NEG	NEG	NEG	NEG	NEG	POS	POS	POS
ST87-MRSA-IV, WA-24	NEG	NEG	NEG	NEG	NEG	NEG	NEG	NEG	NEG	NEG	NEG	NEG	POS	POS	POS
ST59-MRSA-IV [PVL+], WA-55/56	NEG	AMB	NEG	NEG	NEG	NEG	NEG	NEG	NEG	NEG	NEG	NEG	COMM	COMM	COMM
ST59/ST952-MRSA-V(T) [PVL+], Taiwan Clone	NEG	NEG	NEG	NEG	NEG	NEG	NEG	POS	POS	POS	NEG	NEG	COMM	COMM	COMM
ST59-MRSA-V	NEG	NEG	NEG	NEG	NEG	NEG	NEG	POS	NEG	POS	NEG	NEG	COMM	COMM	COMM
CC59-MRSA-V [PVL+]	NEG	NEG	NEG	NEG	NEG	NEG	NEG	POS	NEG	POS	NEG	NEG	RARE	RARE	RARE
ST59-MRSA-IV/V, WA-15	NEG	NEG	NEG	NEG	NEG	NEG	NEG	POS	NEG	POS	NEG	NEG	POS	POS	POS
ST72-MRSA-IV, USA700	NEG	NEG	NEG	NEG	NEG	NEG	NEG	n.t.	NEG	NEG	NEG	NEG	POS	POS	POS
ST72-MRSA-IV [PVL+], WA-44	NEG	NEG	NEG	NEG	NEG	NEG	NEG	NEG	NEG	NEG	NEG	NEG	POS	POS	POS
CC75-MRSA-IV	NEG	NEG	NEG	NEG	NEG	NEG	NEG	NEG	NEG	NEG	NEG	NEG	POS	POS	POS
ST883-MRSA-IV, WA-47	NEG	NEG	NEG	NEG	NEG	NEG	NEG	NEG	NEG	NEG	NEG	NEG	COMM	COMM	COMM
ST1303-MRSA-IV	NEG	NEG	NEG	NEG	AMB	NEG	NEG	NEG	NEG	NEG	NEG	NEG	POS	POS	POS
CC80-MRSA-IV	NEG	NEG	NEG	NEG	NEG	NEG	NEG	n.t.	NEG	NEG	NEG	NEG	VAR	VAR	VAR
CC80-MRSA-IV [PVL+], European Clone	NEG	NEG	NEG	NEG	NEG	NEG	NEG	NEG	NEG	NEG	NEG	NEG	COMM	COMM	COMM
CC88-MRSA-IV, WA-2	NEG	NEG	NEG	NEG	NEG	NEG	NEG	NEG	NEG	NEG	NEG	NEG	COMM	COMM	COMM
CC88-MRSA-IV [PVL+]	NEG	NEG	NEG	NEG	NEG	NEG	NEG	NEG	NEG	NEG	NEG	NEG	COMM	COMM	COMM
CC88-MRSA-V [PVL+]	NEG	NEG	NEG	NEG	NEG	NEG	NEG	n.t.	POS	POS	NEG	NEG	NEG	NEG	NEG
CC88-MRSA-VI	NEG	NEG	NEG	NEG	NEG	NEG	NEG	n.t.	NEG	NEG	POS	POS	NEG	NEG	NEG
ST93-MRSA-IV [PVL-]	NEG	NEG	NEG	NEG	NEG	NEG	NEG	n.t.	NEG	NEG	NEG	NEG	POS	POS	POS
ST93-MRSA-IV [PVL+], Queensland Clone	NEG	NEG	NEG	NEG	NEG	NEG	NEG	NEG	NEG	NEG	NEG	NEG	POS	POS	POS
ST93-MRSA-V [PVL+]	NEG	NEG	NEG	NEG	NEG	NEG	NEG	NEG	POS	POS	NEG	NEG	POS	POS	POS
CC97-MRSA-IV, WA-54	NEG	NEG	NEG	NEG	NEG	NEG	NEG	NEG	NEG	NEG	NEG	NEG	POS	POS	POS
CC97-MRSA-V	NEG	NEG	NEG	NEG	NEG	NEG	NEG	n.t.	POS	POS	NEG	NEG	NEG	NEG	NEG
CC97-MRSA-V [ACME+]	NEG	NEG	NEG	NEG	NEG	NEG	NEG	n.t.	POS	POS	AMB	POS	POS	POS	POS
CC97-MRSA-I/V	NEG	NEG	NEG	NEG	NEG	NEG	NEG	n.t.	POS	POS	NEG	NEG	POS	POS	POS
CC121-MRSA-V, WA-22	NEG	NEG	NEG	NEG	NEG	NEG	NEG	POS	POS	POS	NEG	NEG	POS	POS	POS
CC152-MRSA-V [PVL+]	NEG	NEG	NEG	NEG	NEG	NEG	NEG	POS	POS	POS	NEG	NEG	POS	POS	POS
ST154-MRSA [PVL+]	NEG	NEG	NEG	NEG	NEG	NEG	NEG	n.t.	NEG	NEG	NEG	NEG	POS	POS	POS
ST188-MRSA-IV, WA-38/78	NEG	NEG	NEG	NEG	NEG	NEG	NEG	NEG	NEG	NEG	NEG	NEG	POS	POS	POS
ST188-MRSA-V	NEG	NEG	NEG	NEG	NEG	NEG	NEG	n.t.	POS	POS	NEG	NEG	POS	POS	POS
ST239-MRSA-III	POS	POS	POS	POS	POS	COMM	COMM	COMM	NEG	COMM	NEG	NEG	POS	POS	POS
CC361-MRSA-IV, WA-29	NEG	NEG	NEG	NEG	NEG	NEG	NEG	NEG	NEG	NEG	NEG	NEG	POS	POS	POS
CC361-MRSA-V	NEG	NEG	NEG	NEG	NEG	NEG	NEG	n.t.	POS	POS	NEG	NEG	POS	POS	POS
CC361-MRSA-VIII, WA-28	POS	POS	POS	NEG	NEG	NEG	NEG	NEG	NEG	NEG	NEG	POS	POS	POS	POS
CC398-MRSA-IV	NEG	NEG	NEG	NEG	NEG	NEG	NEG	n.t.	NEG	NEG	NEG	NEG	POS	POS	POS
ST398-MRSA-V	NEG	NEG	NEG	NEG	NEG	NEG	NEG	VAR	COMM	COMM	NEG	NEG	COMM	COMM	COMM
ST398-MRSA-V [PVL+]	NEG	NEG	NEG	NEG	NEG	NEG	NEG	n.t.	POS	POS	NEG	NEG	POS	POS	POS
CC509/ST207-MRSA-V	NEG	NEG	NEG	NEG	NEG	NEG	NEG	NEG	POS	POS	NEG	NEG	POS	POS	POS
ST573-MRSA-V, WA-10	NEG	NEG	NEG	NEG	NEG	NEG	NEG	NEG	POS	POS	NEG	NEG	POS	POS	POS
ST772-MRSA-V [PVL+], WA-60/Bengal Bay Clone	NEG	NEG	NEG	NEG	NEG	NEG	NEG	NEG	POS	POS	NEG	NEG	COMM	COMM	COMM
ST779-MRSA	NEG	NEG	NEG	NEG	NEG	NEG	NEG	VAR	COMM	RARE	NEG	VAR	POS	POS	POS
ST834-MRSA-IV, WA-13	NEG	NEG	NEG	NEG	NEG	NEG	NEG	NEG	NEG	NEG	NEG	NEG	VAR	VAR	VAR
CC913-MRSA-IV	NEG	NEG	NEG	NEG	NEG	NEG	NEG	NEG	NEG	NEG	NEG	NEG	AMB	POS	POS
ST1774-MRSA-IV [ACME+]	NEG	NEG	NEG	NEG	NEG	NEG	NEG	n.t.	NEG	POS	NEG	NEG	COMM	COMM	COMM
CC15-MSSA	NEG	NEG	NEG	NEG	NEG	NEG	NEG	NEG	NEG	NEG	NEG	NEG	COMM	COMM	COMM
ST426-MSSA	NEG	NEG	NEG	NEG	NEG	NEG	NEG	NEG	NEG	NEG	NEG	NEG	RARE	RARE	RARE

Legend:

Positive :	POS
Ambiguous :	AMB
Negative :	NEG
Rare (in >0 to 33% of isolates) :	RARE
Variable (in >33 to 66% of isolates) :	VAR
Common (in >66 to <100% of isolates) :	COMM

	RESISTANCE : MLS-ANTIBIOTICS												RESISTANCE : AMINOGLYSOSIDES		
	erm(A)	erm(B)	erm(C)	lnu(A)	msr(A)	mef(A)	mpb(C)	vat(A)	vat(B)	vga(A)	vga(A) BM 3327	vgb(A)	aacA-aphD	aadD	aphA3
CC1-MRSA-IV [PVL-], WA-1/57	NEG	NEG	RARE	NEG	NEG	NEG	NEG	NEG	NEG	NEG	NEG	NEG	RARE	NEG	NEG
CC1-MRSA-IV [PVL+], USA400	NEG	NEG	RARE	NEG	NEG	NEG	NEG	NEG	NEG	NEG	NEG	NEG	NEG	NEG	NEG
CC1-MRSA-IV/SCCfus [PVL-]	NEG	NEG	COMM	NEG	NEG	NEG	NEG	NEG	NEG	NEG	NEG	NEG	NEG	RARE	NEG
CC1-MRSA-IV/SCCfus [PVL+]	NEG	NEG	NEG	NEG	NEG	NEG	NEG	NEG	NEG	NEG	NEG	NEG	NEG	NEG	NEG
CC1-MRSA-V [PVL+]	NEG	NEG	NEG	NEG	NEG	NEG	NEG	NEG	NEG	NEG	NEG	NEG	NEG	NEG	NEG
CC1-MRSA-V/SCCfus [PVL-]	NEG	NEG	COMM	NEG	NEG	NEG	NEG	NEG	NEG	NEG	NEG	NEG	NEG	NEG	NEG
CC1-MRSA-V/SCCfus [PVL+]	NEG	NEG	NEG	NEG	NEG	NEG	NEG	NEG	NEG	NEG	NEG	NEG	NEG	NEG	POS
ST228-MRSA-I, South German/Italian MRSA	COMM	NEG	NEG	NEG	NEG	NEG	NEG	NEG	NEG	NEG	NEG	NEG	POS	NEG	POS
ST5-MRSA-I, Geraldine Clone	NEG	NEG	RARE	NEG	NEG	NEG	NEG	NEG	NEG	NEG	NEG	NEG	NEG	COMM	NEG
CC5-MRSA-Ivar., WA-18/21/48	NEG	NEG	NEG	NEG	NEG	NEG	NEG	NEG	NEG	NEG	NEG	NEG	NEG	NEG	NEG
CC5-MRSA-II, Rhine-Hesse /UK-3	POS	NEG	RARE	NEG	NEG	NEG	RARE	NEG	NEG	NEG	NEG	NEG	RARE	COMM	NEG
CC5-MRSA-II [ACME+]	POS	NEG	NEG	NEG	NEG	NEG	NEG	NEG	NEG	NEG	NEG	NEG	POS	POS	NEG
CC5-MRSA-III	POS	NEG	NEG	NEG	NEG	NEG	NEG	NEG	NEG	NEG	NEG	NEG	NEG	NEG	NEG
CC5-MRSA-IV, Paediatric clone	NEG	RARE	RARE	NEG	RARE	NEG	RARE	NEG	NEG	NEG	NEG	NEG	RARE	VAR	RARE
CC5-MRSA-IV [PVL+]	NEG	NEG	RARE	NEG	NEG	NEG	NEG	NEG	NEG	NEG	RARE	NEG	NEG	RARE	NEG
CC5-MRSA-V	NEG	NEG	RARE	NEG	NEG	NEG	NEG	NEG	NEG	NEG	NEG	NEG	VAR	RARE	NEG
CC5-MRSA-V [PVL+]	NEG	NEG	COMM	NEG	NEG	NEG	NEG	NEG	NEG	NEG	NEG	NEG	VAR	NEG	NEG
CC5-MRSA-VI, New Paediatric Clone	NEG	NEG	NEG	NEG	NEG	NEG	NEG	NEG	NEG	NEG	NEG	NEG	NEG	NEG	RARE
ST5-MRSA-VII (SCC-JCSC6082)	NEG	NEG	NEG	NEG	NEG	NEG	NEG	NEG	NEG	NEG	NEG	NEG	NEG	NEG	NEG
CC5-MRSA-IVvar, Maltese Clone	NEG	NEG	RARE	NEG	NEG	NEG	NEG	NEG	NEG	NEG	NEG	NEG	NEG	NEG	NEG
CC5-MRSA-SCC(MRSAZH47)	NEG	NEG	NEG	NEG	NEG	NEG	NEG	NEG	NEG	NEG	NEG	NEG	POS	NEG	NEG
CC5-MRSA-IV/VI	NEG	NEG	VAR	NEG	VAR	NEG	VAR	NEG	NEG	NEG	NEG	NEG	RARE	COMM	VAR
CC5-MRSA-IV/SCCfus	NEG	NEG	POS	NEG	NEG	NEG	NEG	NEG	NEG	NEG	NEG	NEG	NEG	NEG	NEG
CC5/ST835-MRSA, WA-40/46	NEG	NEG	NEG	NEG	NEG	NEG	NEG	NEG	NEG	NEG	NEG	NEG	NEG	NEG	NEG
ST6-MRSA-IV, WA-51	NEG	NEG	NEG	NEG	NEG	NEG	NEG	NEG	NEG	NEG	NEG	NEG	NEG	NEG	NEG
ST6-MRSA-V	NEG	NEG	NEG	NEG	NEG	NEG	NEG	NEG	NEG	NEG	NEG	NEG	NEG	NEG	NEG
CC7-MRSA-IV	NEG	NEG	NEG	NEG	NEG	NEG	NEG	NEG	NEG	NEG	NEG	NEG	NEG	NEG	NEG
CC7-MRSA-V	NEG	POS	NEG	NEG	NEG	NEG	NEG	NEG	NEG	NEG	NEG	NEG	POS	NEG	NEG
ST250-MRSA-I, Early/Ancestral MRSA	COMM	NEG	NEG	NEG	NEG	NEG	NEG	NEG	NEG	NEG	NEG	NEG	VAR	NEG	RARE
ST247-MRSA-I, North German/Iberian MRSA	VAR	NEG	RARE	NEG	NEG	NEG	NEG	NEG	NEG	NEG	NEG	NEG	COMM	VAR	NEG
CC8-MRSA-IV, UK-14/WA-5	RARE	NEG	RARE	NEG	RARE	NEG	RARE	NEG	NEG	NEG	NEG	NEG	RARE	RARE	RARE
CC8-MRSA-IV, Lyon Clone/UK-2	VAR	NEG	RARE	NEG	NEG	NEG	NEG	NEG	NEG	NEG	RARE	NEG	RARE	VAR	NEG
CC8-MRSA-IV, UK-6	VAR	NEG	RARE	NEG	VAR	NEG	VAR	NEG	NEG	NEG	NEG	NEG	RARE	VAR	POS
CC8-MRSA-IV, USA500	RARE	NEG	VAR	NEG	RARE	NEG	RARE	NEG	NEG	NEG	NEG	NEG	COMM	RARE	RARE
ST8-MRSA-IV [PVL/ACME+], USA300	NEG	NEG	RARE	NEG	COMM	NEG	COMM	NEG	NEG	NEG	NEG	NEG	NEG	RARE	COMM
ST8-MRSA-IV [PVL+], USA300	NEG	NEG	NEG	RARE	RARE	NEG	RARE	NEG	NEG	NEG	NEG	NEG	RARE	RARE	RARE
CC8-MRSA-IV [PVL/sed/j/r/k/q+]	NEG	NEG	NEG	NEG	POS	NEG	POS	NEG	NEG	NEG	NEG	NEG	NEG	NEG	NEG
CC8-MRSA-IV [tst1/sec/sel+]	NEG	NEG	NEG	NEG	NEG	NEG	NEG	NEG	NEG	NEG	NEG	NEG	COMM	POS	NEG
CC8-MRSA-V	NEG	NEG	COMM	NEG	NEG	NEG	NEG	NEG	NEG	NEG	NEG	NEG	VAR	NEG	NEG
CC8-MRSA-VIII	POS	NEG	NEG	NEG	NEG	NEG	NEG	NEG	NEG	NEG	NEG	NEG	POS	NEG	NEG
ST8-MRSA-IIA/B/C/D/E, Irish AR13/14	POS	NEG	RARE	NEG	NEG	NEG	NEG	NEG	NEG	NEG	NEG	NEG	POS	VAR	COMM
ST8-MRSA, UK-12/13, Irish AR43	COMM	NEG	NEG	NEG	NEG	NEG	NEG	NEG	NEG	NEG	NEG	NEG	RARE	NEG	COMM
ST254-MRSA, Hannover EMRSA	RARE	NEG	NEG	NEG	NEG	NEG	NEG	NEG	NEG	NEG	NEG	NEG	POS	NEG	POS
ST254-MRSA-IV/V, Hannover/UK-10	POS	NEG	NEG	NEG	NEG	NEG	NEG	NEG	NEG	NEG	NEG	NEG	POS	NEG	COMM
CC9-MRSA-IV	NEG	VAR	NEG	NEG	NEG	NEG	NEG	NEG	NEG	NEG	NEG	NEG	NEG	VAR	NEG
CC12-MRSA, WA-59	NEG	NEG	NEG	NEG	NEG	NEG	NEG	NEG	NEG	NEG	NEG	NEG	NEG	NEG	NEG
CC12-MRSA-IV	NEG	NEG	NEG	NEG	NEG	NEG	NEG	NEG	NEG	NEG	NEG	NEG	NEG	NEG	NEG
CC20-MRSA-V	NEG	NEG	NEG	NEG	NEG	NEG	NEG	NEG	NEG	NEG	NEG	NEG	NEG	NEG	NEG
ST22-MRSA-IV, Barnim/UK-15	NEG	NEG	VAR	NEG	NEG	NEG	NEG	NEG	NEG	RARE	NEG	NEG	RARE	RARE	NEG
ST22-MRSA-IV [ACME+]	NEG	NEG	POS	VAR	NEG	NEG	NEG	NEG	NEG	NEG	NEG	NEG	VAR	VAR	NEG
ST22-MRSA-IV [PVL+]	NEG	NEG	VAR	NEG	NEG	NEG	NEG	NEG	NEG	NEG	NEG	NEG	VAR	VAR	NEG
ST22-MRSA-V	NEG	NEG	NEG	NEG	NEG	NEG	NEG	NEG	NEG	NEG	NEG	NEG	NEG	NEG	NEG
ST22-MRSA-V [PVL+]	NEG	NEG	POS	NEG	NEG	NEG	NEG	NEG	NEG	NEG	NEG	NEG	NEG	NEG	NEG
ST36-MRSA-II, UK-16	POS	NEG	RARE	RARE	NEG	NEG	NEG	NEG	NEG	NEG	NEG	NEG	RARE	COMM	NEG
ST30-MRSA-IV [PVL+], Southwest Pacific Clone	NEG	NEG	RARE	NEG	NEG	NEG	NEG	NEG	NEG	NEG	NEG	NEG	RARE	RARE	NEG
ST30-MRSA-IV [tst1+]	NEG	NEG	NEG	NEG	NEG	NEG	NEG	NEG	NEG	NEG	NEG	NEG	NEG	RARE	NEG
CC30-MRSA-V [PVL+]	NEG	NEG	NEG	NEG	NEG	NEG	NEG	NEG	NEG	NEG	NEG	NEG	NEG	NEG	NEG
ST45-MRSA-II, USA600	POS	NEG	NEG	NEG	NEG	NEG	NEG	NEG	NEG	NEG	NEG	NEG	RARE	POS	NEG
ST45-MRSA-IV, Berlin EMRSA	RARE	NEG	RARE	NEG	NEG	NEG	NEG	NEG	NEG	NEG	NEG	NEG	RARE	RARE	VAR
CC45-MRSA-IV [ACME+]	NEG	NEG	NEG	NEG	NEG	NEG	NEG	NEG	NEG	NEG	NEG	NEG	NEG	NEG	NEG
ST45-MRSA-V, WA-4	NEG	NEG	RARE	NEG	NEG	NEG	NEG	NEG	NEG	NEG	NEG	NEG	NEG	NEG	NEG
CC45/agr IV-MRSA-IV, WA-23	NEG	NEG	RARE	NEG	NEG	NEG	NEG	NEG	NEG	NEG	NEG	NEG	NEG	RARE	NEG
CC45/agr IV-MRSA-V	NEG	NEG	VAR	NEG	NEG	NEG	NEG	NEG	NEG	NEG	NEG	NEG	VAR	RARE	NEG
CC45/agr IV-MRSA-IV/V	NEG	NEG	COMM	NEG	NEG	NEG	NEG	NEG	NEG	NEG	NEG	NEG	NEG	COMM	NEG
CC59-MRSA-IV [PVL+], USA1000	RARE	NEG	NEG	NEG	NEG	NEG	NEG	NEG	NEG	NEG	NEG	NEG	NEG	NEG	NEG
ST59-MRSA-IV, WA-73	NEG	NEG	NEG	NEG	NEG	NEG	NEG	NEG	NEG	NEG	NEG	NEG	NEG	NEG	NEG
ST87-MRSA-IV, WA-24	NEG	NEG	NEG	NEG	POS	NEG	POS	NEG	NEG	NEG	NEG	NEG	NEG	NEG	POS
ST59-MRSA-IV [PVL+], WA-55/56	NEG	POS	NEG	NEG	NEG	NEG	NEG	NEG	NEG	NEG	NEG	NEG	NEG	NEG	POS
ST59/ST952-MRSA-V(T) [PVL+], Taiwan Clone	NEG	POS	NEG	NEG	NEG	NEG	NEG	NEG	NEG	NEG	NEG	NEG	NEG	NEG	POS
ST59-MRSA-V	NEG	NEG	RARE	NEG	NEG	NEG	NEG	NEG	NEG	NEG	NEG	NEG	NEG	NEG	NEG
CC59-MRSA-V [PVL+]	NEG	NEG	NEG	NEG	NEG	NEG	NEG	NEG	NEG	NEG	NEG	NEG	NEG	NEG	NEG
ST59-MRSA-IV/V, WA-15	NEG	NEG	NEG	NEG	RARE	NEG	RARE	NEG	NEG	NEG	NEG	NEG	NEG	NEG	RARE
ST72-MRSA-IV, USA700	NEG	NEG	RARE	NEG	RARE	NEG	RARE	NEG	NEG	NEG	NEG	NEG	RARE	VAR	RARE
ST72-MRSA-IV [PVL+], WA-44	NEG	NEG	NEG	NEG	NEG	NEG	NEG	NEG	NEG	NEG	NEG	NEG	NEG	NEG	NEG
CC75-MRSA-IV	NEG	NEG	NEG	NEG	RARE	NEG	NEG	NEG	NEG	NEG	NEG	NEG	NEG	NEG	NEG
ST883-MRSA-IV, WA-47	NEG	NEG	COMM	NEG	NEG	NEG	NEG	NEG	NEG	NEG	NEG	NEG	NEG	NEG	NEG
ST1303-MRSA-IV	NEG	NEG	NEG	NEG	NEG	NEG	NEG	NEG	NEG	NEG	NEG	NEG	NEG	NEG	NEG
CC80-MRSA-IV	NEG	NEG	NEG	NEG	NEG	NEG	NEG	NEG	NEG	NEG	NEG	NEG	RARE	NEG	COMM
CC80-MRSA-IV [PVL+], European Clone	NEG	NEG	RARE	RARE	NEG	NEG	NEG	NEG	NEG	NEG	NEG	NEG	RARE	NEG	POS
CC88-MRSA-IV, WA-2	COMM	NEG	RARE	NEG	NEG	NEG	NEG	NEG	NEG	NEG	RARE	NEG	RARE	NEG	NEG
CC88-MRSA-IV [PVL+]	RARE	NEG	RARE	NEG	NEG	NEG	NEG	NEG	NEG	NEG	NEG	NEG	NEG	NEG	NEG
CC88-MRSA-V [PVL+]	NEG	NEG	NEG	NEG	NEG	NEG	NEG	NEG	NEG	NEG	NEG	NEG	POS	NEG	NEG
CC88-MRSA-VI	POS	NEG	NEG	NEG	NEG	NEG	NEG	NEG	NEG	NEG	NEG	NEG	NEG	NEG	NEG
ST93-MRSA-IV [PVL-]	NEG	NEG	NEG	NEG	VAR	NEG	NEG	NEG	NEG	NEG	NEG	NEG	NEG	NEG	NEG
ST93-MRSA-IV [PVL+], Queensland Clone	NEG	NEG	RARE	NEG	NEG	NEG	NEG	NEG	NEG	NEG	NEG	NEG	NEG	NEG	NEG
ST93-MRSA-V [PVL+]	NEG	NEG	NEG	NEG	NEG	NEG	NEG	NEG	NEG	NEG	NEG	NEG	NEG	NEG	NEG
CC97-MRSA-IV, WA-54	NEG	NEG	NEG	NEG	NEG	NEG	NEG	NEG	NEG	NEG	NEG	NEG	NEG	RARE	NEG
CC97-MRSA-V	NEG	NEG	NEG	NEG	NEG	NEG	NEG	NEG	NEG	NEG	NEG	NEG	NEG	NEG	NEG
CC97-MRSA-V [ACME+]	NEG	NEG	POS	NEG	NEG	NEG	NEG	NEG	NEG	NEG	NEG	NEG	NEG	NEG	NEG
CC97-MRSA-I/V	NEG	NEG	VAR	NEG	NEG	NEG	NEG	NEG	NEG	NEG	VAR	NEG	NEG	NEG	NEG
CC121-MRSA-V, WA-22	POS	NEG	NEG	NEG	NEG	NEG	NEG	NEG	NEG	NEG	NEG	NEG	NEG	NEG	NEG
CC152-MRSA-V [PVL+]	NEG	NEG	NEG	NEG	NEG	NEG	NEG	NEG	NEG	NEG	NEG	NEG	POS	NEG	NEG
ST154-MRSA [PVL+]	NEG	NEG	NEG	NEG	NEG	NEG	NEG	NEG	NEG	NEG	NEG	NEG	NEG	NEG	NEG
ST188-MRSA-IV, WA-38/78	NEG	COMM	NEG	NEG	NEG	NEG	NEG	NEG	NEG	NEG	NEG	NEG	POS	NEG	NEG
ST188-MRSA-V	NEG	NEG	NEG	NEG	NEG	NEG	NEG	NEG	NEG	NEG	NEG	NEG	NEG	NEG	NEG
ST239-MRSA-III	COMM	NEG	RARE	NEG	NEG	NEG	NEG	NEG	NEG	NEG	NEG	NEG	COMM	RARE	COMM
CC361-MRSA-IV, WA-29	NEG	NEG	NEG	NEG	NEG	NEG	NEG	NEG	NEG	NEG	NEG	NEG	VAR	NEG	VAR
CC361-MRSA-V	NEG	NEG	NEG	NEG	NEG	NEG	NEG	NEG	NEG	NEG	NEG	NEG	POS	NEG	POS
CC361-MRSA-VIII, WA-28	NEG	NEG	NEG	NEG	NEG	NEG	NEG	NEG	NEG	NEG	NEG	NEG	NEG	NEG	POS
CC398-MRSA-IV	RARE	RARE	VAR	NEG	NEG	NEG	NEG	NEG	NEG	RARE	RARE	NEG	COMM	VAR	NEG
ST398-MRSA-V	RARE	RARE	RARE	RARE	NEG	NEG	NEG	NEG	NEG	RARE	RARE	NEG	RARE	RARE	NEG
ST398-MRSA-V [PVL+]	NEG	NEG	VAR	NEG	NEG	NEG	NEG	NEG	NEG	NEG	NEG	NEG	NEG	NEG	NEG
CC509/ST207-MRSA-V	NEG	NEG	POS	NEG	NEG	NEG	NEG	NEG	NEG	NEG	NEG	NEG	NEG	NEG	NEG
ST573-MRSA-V, WA-10	NEG	NEG	NEG	NEG	NEG	NEG	NEG	NEG	NEG	NEG	NEG	NEG	NEG	NEG	NEG
ST772-MRSA-V [PVL+], WA-60/Bengal Bay Clone	NEG	NEG	RARE	NEG	COMM	NEG	COMM	NEG	NEG	NEG	NEG	NEG	VAR	NEG	COMM
ST779-MRSA	NEG	NEG	NEG	NEG	NEG	NEG	NEG	NEG	NEG	NEG	NEG	NEG	NEG	RARE	NEG
ST834-MRSA-IV, WA-13	NEG	n.t.	NEG	NEG	VAR	NEG	NEG	NEG	NEG	NEG	NEG	NEG	NEG	NEG	NEG
CC913-MRSA-IV	NEG	NEG	NEG	NEG	NEG	NEG	NEG	NEG	NEG	NEG	NEG	NEG	NEG	NEG	POS
ST1774-MRSA-IV [ACME+]	NEG	NEG	NEG	NEG	NEG	NEG	NEG	NEG	NEG	NEG	NEG	NEG	NEG	NEG	NEG
CC15-MSSA	NEG	NEG	RARE	NEG	NEG	NEG	NEG	NEG	NEG	NEG	NEG	NEG	NEG	NEG	NEG
ST426-MSSA	NEG	NEG	NEG	NEG	NEG	NEG	NEG	NEG	NEG	NEG	NEG	NEG	NEG	NEG	NEG

Legend:

Positive :	POS
Ambiguous :	AMB
Negative :	NEG
Rare (in >0 to 33% of isolates) :	RARE
Variable (in >33 to 66% of isolates) :	VAR
Common (in >66 to <100% of isolates) :	COMM

	RESISTANCE : MISCELLANEOUS GENES															
	sat	dfrA	far1	Q6GD50	mupA	tet(K)	tet(M)	cat (total)	cat (pC221)	cat (pc223)	cat (pMC524)	cat (pSBK 203R)	cfr	fexA	fosB	fosB (plasmid)
CC1-MRSA-IV [PVL-], WA-1/57	NEG	RARE	NEG	NEG	NEG	RARE	RARE	NEG	NEG	NEG	NEG	NEG	NEG	NEG	NEG	NEG
CC1-MRSA-IV [PVL+], USA400	NEG	NEG	NEG	NEG	NEG	NEG	RARE	NEG	NEG	NEG	NEG	NEG	NEG	NEG	NEG	NEG
CC1-MRSA-IV/SCCfus [PVL-]	NEG	RARE	NEG	POS	RARE	RARE	NEG	NEG	NEG	NEG	NEG	NEG	NEG	NEG	NEG	NEG
CC1-MRSA-IV/SCCfus [PVL+]	NEG	NEG	NEG	POS	NEG	NEG	NEG	NEG	NEG	NEG	NEG	NEG	NEG	NEG	NEG	NEG
CC1-MRSA-V [PVL+]	NEG	NEG	NEG	NEG	NEG	VAR	NEG	NEG	NEG	NEG	NEG	NEG	NEG	NEG	NEG	NEG
CC1-MRSA-V/SCCfus [PVL-]	NEG	NEG	NEG	POS	NEG	COMM	NEG	NEG	NEG	NEG	NEG	NEG	NEG	NEG	NEG	NEG
CC1-MRSA-V/SCCfus [PVL+]	POS	NEG	NEG	POS	NEG	NEG	NEG	NEG	NEG	NEG	NEG	NEG	NEG	NEG	NEG	NEG
ST228-MRSA-I, South German/Italian MRSA	POS	RARE	NEG	NEG	RARE	NEG	NEG	NEG	NEG	NEG	NEG	NEG	NEG	NEG	POS	NEG
ST5-MRSA-I, Geraldine Clone	NEG	NEG	NEG	COMM	NEG	NEG	NEG	NEG	NEG	NEG	NEG	NEG	NEG	NEG	POS	NEG
CC5-MRSA-Ivar., WA-18/21/48	NEG	NEG	NEG	NEG	NEG	NEG	NEG	NEG	NEG	NEG	NEG	NEG	NEG	NEG	POS	NEG
CC5-MRSA-II, Rhine-Hesse /UK-3	NEG	RARE	NEG	NEG	RARE	RARE	RARE	NEG	NEG	NEG	NEG	NEG	NEG	NEG	POS	NEG
CC5-MRSA-II [ACME+]	NEG	NEG	NEG	NEG	NEG	NEG	POS	NEG	NEG	NEG	NEG	NEG	NEG	NEG	POS	NEG
CC5-MRSA-III	NEG	NEG	NEG	NEG	NEG	NEG	POS	NEG	NEG	NEG	NEG	NEG	NEG	NEG	POS	NEG
CC5-MRSA-IV, Paediatric clone	RARE	RARE	NEG	RARE	RARE	RARE	NEG	RARE	RARE	NEG	NEG	NEG	NEG	NEG	POS	NEG
CC5-MRSA-IV [PVL+]	NEG	VAR	NEG	RARE	NEG	NEG	COMM	NEG	NEG	NEG	NEG	NEG	NEG	NEG	POS	NEG
CC5-MRSA-V	NEG	NEG	NEG	RARE	NEG	RARE	RARE	NEG	NEG	NEG	NEG	NEG	NEG	RARE	POS	NEG
CC5-MRSA-V [PVL+]	NEG	NEG	NEG	COMM	NEG	NEG	NEG	NEG	NEG	NEG	NEG	NEG	NEG	NEG	POS	NEG
CC5-MRSA-VI, New Paediatric Clone	RARE	NEG	NEG	NEG	NEG	NEG	NEG	NEG	NEG	NEG	NEG	NEG	NEG	NEG	POS	NEG
ST5-MRSA-VII (SCC-JCSC6082)	NEG	NEG	NEG	NEG	NEG	NEG	POS	NEG	NEG	NEG	NEG	NEG	NEG	NEG	POS	NEG
CC5-MRSA-IVvar, Maltese Clone	NEG	NEG	NEG	POS	NEG	RARE	NEG	NEG	NEG	NEG	NEG	NEG	NEG	NEG	POS	NEG
CC5-MRSA-SCC(MRSAZH47)	NEG	NEG	NEG	NEG	NEG	NEG	NEG	NEG	NEG	NEG	NEG	NEG	NEG	NEG	POS	NEG
CC5-MRSA-IV/VI	VAR	NEG	NEG	RARE	RARE	NEG	NEG	NEG	RARE	NEG	NEG	NEG	NEG	NEG	POS	NEG
CC5-MRSA-IV/SCCfus	NEG	NEG	NEG	POS	NEG	NEG	NEG	NEG	NEG	NEG	NEG	NEG	NEG	NEG	POS	NEG
CC5/ST835-MRSA, WA-40/46	NEG	RARE	NEG	NEG	RARE	NEG	NEG	NEG	NEG	NEG	NEG	NEG	NEG	NEG	POS	NEG
ST6-MRSA-IV, WA-51	NEG	NEG	NEG	NEG	NEG	NEG	NEG	NEG	NEG	NEG	NEG	NEG	NEG	NEG	POS	NEG
ST6-MRSA-V	NEG	NEG	NEG	NEG	NEG	NEG	NEG	NEG	NEG	NEG	NEG	NEG	NEG	NEG	POS	AMB
CC7-MRSA-IV	NEG	NEG	NEG	NEG	NEG	NEG	NEG	NEG	NEG	NEG	NEG	NEG	NEG	NEG	NEG	NEG
CC7-MRSA-V	NEG	NEG	NEG	NEG	NEG	NEG	NEG	NEG	NEG	NEG	NEG	NEG	NEG	NEG	NEG	NEG
ST250-MRSA-I, Early/Ancestral MRSA	RARE	RARE	NEG	NEG	NEG	COMM	RARE	NEG	NEG	NEG	NEG	NEG	NEG	NEG	POS	NEG
ST247-MRSA-I, North German/Iberian MRSA	NEG	NEG	NEG	NEG	NEG	RARE	POS	NEG	NEG	NEG	NEG	NEG	NEG	NEG	POS	NEG
CC8-MRSA-IV, UK-14/WA-5	RARE	RARE	NEG	NEG	NEG	RARE	RARE	RARE	RARE	NEG	RARE	NEG	NEG	NEG	POS	NEG
CC8-MRSA-IV, Lyon Clone/UK-2	NEG	RARE	RARE	RARE	RARE	RARE	RARE	RARE	RARE	NEG	NEG	NEG	NEG	NEG	POS	NEG
CC8-MRSA-IV, UK-6	POS	NEG	NEG	NEG	NEG	RARE	RARE	NEG	NEG	NEG	NEG	NEG	NEG	NEG	POS	NEG
CC8-MRSA-IV, USA500	RARE	COMM	NEG	NEG	NEG	NEG	COMM	RARE	RARE	NEG	NEG	NEG	NEG	NEG	POS	NEG
ST8-MRSA-IV [PVL/ACME+], USA300	COMM	NEG	NEG	NEG	RARE	RARE	NEG	NEG	NEG	NEG	NEG	NEG	NEG	NEG	POS	NEG
ST8-MRSA-IV [PVL+], USA300	RARE	NEG	NEG	NEG	RARE	RARE	NEG	NEG	NEG	NEG	NEG	NEG	NEG	NEG	POS	NEG
CC8-MRSA-IV [PVL/sed/j/r/k/q+]	NEG	NEG	NEG	NEG	NEG	POS	NEG	NEG	NEG	NEG	NEG	NEG	NEG	NEG	POS	AMB
CC8-MRSA-IV [tst1/sec/sel+]	NEG	NEG	NEG	NEG	NEG	NEG	NEG	NEG	NEG	NEG	NEG	NEG	NEG	NEG	POS	AMB
CC8-MRSA-V	NEG	NEG	NEG	NEG	NEG	RARE	NEG	NEG	NEG	NEG	NEG	NEG	NEG	NEG	POS	NEG
CC8-MRSA-VIII	NEG	NEG	NEG	NEG	VAR	NEG	NEG	NEG	NEG	NEG	NEG	NEG	NEG	NEG	POS	NEG
ST8-MRSA-IIA/B/C/D/E, Irish AR13/14	COMM	NEG	RARE	NEG	RARE	RARE	NEG	RARE	RARE	NEG	NEG	NEG	NEG	NEG	POS	NEG
ST8-MRSA, UK-12/13, Irish AR43	COMM	RARE	NEG	NEG	NEG	NEG	VAR	NEG	NEG	NEG	NEG	NEG	NEG	NEG	POS	NEG
ST254-MRSA, Hannover EMRSA	POS	RARE	NEG	NEG	NEG	RARE	POS	RARE	RARE	NEG	RARE	NEG	NEG	NEG	POS	NEG
ST254-MRSA-IV/V, Hannover/UK-10	COMM	NEG	NEG	NEG	NEG	RARE	POS	VAR	NEG	NEG	VAR	NEG	NEG	NEG	POS	NEG
CC9-MRSA-IV	NEG	NEG	NEG	NEG	NEG	NEG	NEG	NEG	NEG	NEG	NEG	NEG	NEG	NEG	POS	NEG
CC12-MRSA, WA-59	NEG	NEG	NEG	NEG	NEG	NEG	NEG	NEG	NEG	NEG	NEG	NEG	NEG	NEG	POS	NEG
CC12-MRSA-IV	NEG	NEG	NEG	NEG	NEG	NEG	NEG	NEG	NEG	NEG	NEG	NEG	NEG	NEG	POS	NEG
CC20-MRSA-V	NEG	NEG	NEG	NEG	NEG	NEG	NEG	NEG	NEG	NEG	NEG	NEG	NEG	NEG	POS	NEG
ST22-MRSA-IV, Barnim/UK-15	NEG	NEG	RARE	RARE	RARE	RARE	RARE	NEG	NEG	NEG	NEG	NEG	NEG	RARE	NEG	RARE
ST22-MRSA-IV [ACME+]	NEG	NEG	NEG	NEG	VAR	NEG	NEG	NEG	NEG	NEG	NEG	NEG	NEG	NEG	NEG	NEG
ST22-MRSA-IV [PVL+]	NEG	VAR	NEG	RARE	NEG	NEG	NEG	NEG	NEG	NEG	NEG	NEG	NEG	NEG	NEG	NEG
ST22-MRSA-V	NEG	NEG	NEG	POS	NEG	POS	NEG	NEG	NEG	NEG	NEG	NEG	NEG	NEG	NEG	NEG
ST22-MRSA-V [PVL+]	NEG	POS	NEG	NEG	NEG	NEG	NEG	NEG	NEG	NEG	NEG	NEG	NEG	NEG	NEG	NEG
ST36-MRSA-II, UK-16	NEG	RARE	NEG	NEG	RARE	NEG	NEG	NEG	NEG	NEG	NEG	NEG	NEG	NEG	POS	NEG
ST30-MRSA-IV [PVL+], Southwest Pacific Clone	NEG	NEG	NEG	NEG	RARE	RARE	NEG	NEG	NEG	NEG	NEG	NEG	NEG	NEG	POS	NEG
ST30-MRSA-IV [tst1+]	NEG	NEG	NEG	VAR	NEG	NEG	NEG	NEG	NEG	NEG	NEG	NEG	NEG	NEG	POS	NEG
CC30-MRSA-V [PVL+]	NEG	NEG	NEG	NEG	NEG	NEG	NEG	NEG	NEG	NEG	NEG	NEG	NEG	NEG	POS	NEG
ST45-MRSA-II, USA600	NEG	RARE	NEG	NEG	NEG	NEG	NEG	NEG	NEG	NEG	NEG	NEG	NEG	NEG	NEG	NEG
ST45-MRSA-IV, Berlin EMRSA	VAR	RARE	NEG	NEG	RARE	RARE	NEG	NEG	NEG	NEG	NEG	NEG	NEG	NEG	NEG	NEG
CC45-MRSA-IV [ACME+]	NEG	NEG	NEG	NEG	NEG	NEG	NEG	NEG	NEG	NEG	NEG	NEG	NEG	NEG	NEG	NEG
ST45-MRSA-V, WA-4	NEG	NEG	NEG	NEG	NEG	RARE	NEG	NEG	NEG	NEG	NEG	NEG	NEG	NEG	NEG	NEG
CC45/agr IV-MRSA-IV, WA-23	NEG	NEG	RARE	NEG	NEG	NEG	RARE	NEG	NEG	NEG	NEG	NEG	NEG	NEG	NEG	NEG
CC45/agr IV-MRSA-V	NEG	NEG	NEG	NEG	NEG	COMM	RARE	NEG	NEG	NEG	NEG	NEG	NEG	NEG	NEG	NEG
CC45/agr IV-MRSA-IV/V	NEG	NEG	NEG	NEG	NEG	COMM	NEG	NEG	NEG	NEG	NEG	NEG	NEG	NEG	NEG	NEG
CC59-MRSA-IV [PVL+], USA1000	NEG	NEG	NEG	NEG	NEG	NEG	NEG	NEG	NEG	NEG	NEG	NEG	NEG	NEG	NEG	NEG
ST59-MRSA-IV, WA-73	NEG	NEG	NEG	NEG	NEG	NEG	NEG	NEG	NEG	NEG	NEG	NEG	NEG	NEG	NEG	NEG
ST87-MRSA-IV, WA-24	POS	NEG	NEG	NEG	NEG	NEG	NEG	NEG	NEG	NEG	NEG	NEG	NEG	NEG	NEG	NEG
ST59-MRSA-IV [PVL+], WA-55/56	POS	NEG	NEG	NEG	NEG	COMM	NEG	RARE	NEG	RARE	NEG	RARE	NEG	NEG	NEG	NEG
ST59/ST952-MRSA-V(T) [PVL+], Taiwan Clone	POS	NEG	NEG	NEG	NEG	VAR	NEG	COMM	NEG	COMM	NEG	COMM	NEG	RARE	NEG	NEG
ST59-MRSA-V	NEG	NEG	NEG	NEG	NEG	NEG	NEG	RARE	RARE	NEG	NEG	NEG	NEG	NEG	NEG	NEG
CC59-MRSA-V [PVL+]	NEG	NEG	NEG	NEG	NEG	NEG	NEG	NEG	NEG	NEG	NEG	NEG	NEG	NEG	NEG	NEG
ST59-MRSA-IV/V, WA-15	RARE	NEG	NEG	NEG	NEG	RARE	NEG	NEG	NEG	NEG	NEG	NEG	NEG	NEG	NEG	NEG
ST72-MRSA-IV, USA700	RARE	NEG	NEG	NEG	NEG	NEG	NEG	NEG	NEG	NEG	NEG	NEG	NEG	NEG	POS	NEG
ST72-MRSA-IV [PVL+], WA-44	NEG	POS	NEG	NEG	NEG	NEG	NEG	NEG	NEG	NEG	NEG	NEG	NEG	NEG	POS	NEG
CC75-MRSA-IV	NEG	NEG	NEG	NEG	RARE	NEG	NEG	NEG	NEG	NEG	NEG	NEG	NEG	NEG	POS	NEG
ST883-MRSA-IV, WA-47	NEG	NEG	NEG	NEG	NEG	NEG	NEG	NEG	NEG	NEG	NEG	NEG	NEG	NEG	NEG	NEG
ST1303-MRSA-IV	NEG	NEG	NEG	NEG	NEG	NEG	NEG	NEG	NEG	NEG	NEG	NEG	NEG	NEG	POS	NEG
CC80-MRSA-IV	COMM	NEG	RARE	NEG	NEG	RARE	RARE	NEG	NEG	NEG	NEG	NEG	NEG	NEG	NEG	NEG
CC80-MRSA-IV [PVL+], European Clone	POS	NEG	COMM	NEG	NEG	VAR	NEG	NEG	NEG	NEG	NEG	NEG	NEG	NEG	NEG	NEG
CC88-MRSA-IV, WA-2	NEG	RARE	NEG	NEG	RARE	RARE	RARE	NEG	NEG	NEG	NEG	NEG	NEG	NEG	NEG	NEG
CC88-MRSA-IV [PVL+]	NEG	NEG	NEG	NEG	NEG	COMM	NEG	NEG	NEG	NEG	NEG	NEG	NEG	NEG	NEG	NEG
CC88-MRSA-V [PVL+]	NEG	NEG	NEG	NEG	NEG	NEG	NEG	NEG	NEG	NEG	NEG	NEG	NEG	NEG	NEG	NEG
CC88-MRSA-VI	NEG	NEG	NEG	NEG	NEG	NEG	NEG	NEG	NEG	NEG	NEG	NEG	NEG	NEG	NEG	NEG
ST93-MRSA-IV [PVL-]	NEG	NEG	NEG	NEG	NEG	NEG	NEG	NEG	NEG	NEG	NEG	NEG	NEG	NEG	NEG	NEG
ST93-MRSA-IV [PVL+], Queensland Clone	NEG	NEG	NEG	NEG	NEG	RARE	NEG	NEG	NEG	NEG	NEG	NEG	NEG	NEG	NEG	NEG
ST93-MRSA-V [PVL+]	NEG	NEG	NEG	NEG	NEG	NEG	NEG	NEG	NEG	NEG	NEG	NEG	NEG	NEG	NEG	NEG
CC97-MRSA-IV, WA-54	NEG	NEG	NEG	NEG	NEG	RARE	NEG	NEG	NEG	NEG	NEG	NEG	NEG	NEG	NEG	NEG
CC97-MRSA-V	NEG	NEG	NEG	NEG	NEG	NEG	NEG	NEG	NEG	NEG	NEG	NEG	NEG	NEG	NEG	NEG
CC97-MRSA-V [ACME+]	NEG	NEG	NEG	NEG	NEG	NEG	NEG	NEG	NEG	NEG	NEG	NEG	NEG	NEG	NEG	NEG
CC97-MRSA-I/V	NEG	NEG	NEG	NEG	NEG	POS	POS	VAR	VAR	NEG	NEG	NEG	NEG	NEG	NEG	NEG
CC121-MRSA-V, WA-22	NEG	NEG	NEG	NEG	NEG	NEG	NEG	NEG	NEG	NEG	NEG	NEG	NEG	NEG	POS	NEG
CC152-MRSA-V [PVL+]	NEG	NEG	NEG	NEG	NEG	NEG	NEG	NEG	NEG	NEG	NEG	NEG	NEG	NEG	NEG	NEG
ST154-MRSA [PVL+]	NEG	NEG	VAR	NEG	NEG	NEG	POS	POS	NEG	NEG	POS	NEG	NEG	NEG	NEG	NEG
ST188-MRSA-IV, WA-38/78	NEG	NEG	NEG	NEG	NEG	VAR	NEG	VAR	VAR	NEG	NEG	NEG	NEG	NEG	NEG	NEG
ST188-MRSA-V	NEG	NEG	NEG	NEG	NEG	NEG	NEG	NEG	NEG	NEG	NEG	NEG	NEG	NEG	NEG	NEG
ST239-MRSA-III	COMM	RARE	NEG	NEG	RARE	VAR	POS	RARE	RARE	NEG	RARE	NEG	NEG	NEG	POS	NEG
CC361-MRSA-IV, WA-29	VAR	NEG	NEG	NEG	NEG	VAR	NEG	NEG	NEG	NEG	NEG	NEG	NEG	NEG	POS	NEG
CC361-MRSA-V	POS	NEG	NEG	NEG	NEG	POS	NEG	NEG	NEG	NEG	NEG	NEG	NEG	NEG	POS	NEG
CC361-MRSA-VIII, WA-28	POS	NEG	NEG	NEG	NEG	POS	NEG	NEG	NEG	NEG	NEG	NEG	NEG	NEG	POS	NEG
CC398-MRSA-IV	NEG	RARE	NEG	NEG	NEG	RARE	POS	NEG	NEG	NEG	NEG	NEG	NEG	NEG	NEG	NEG
ST398-MRSA-V	NEG	NEG	NEG	NEG	NEG	COMM	COMM	NEG	NEG	NEG	NEG	NEG	RARE	RARE	NEG	NEG
ST398-MRSA-V [PVL+]	NEG	NEG	NEG	NEG	NEG	VAR	NEG	NEG	NEG	NEG	NEG	NEG	NEG	NEG	NEG	NEG
CC509/ST207-MRSA-V	NEG	NEG	NEG	NEG	NEG	NEG	NEG	NEG	NEG	NEG	NEG	NEG	NEG	NEG	NEG	NEG
ST573-MRSA-V, WA-10	NEG	NEG	NEG	NEG	NEG	NEG	NEG	NEG	NEG	NEG	NEG	NEG	NEG	NEG	POS	NEG
ST772-MRSA-V [PVL+], WA-60/Bengal Bay Clone	COMM	NEG	NEG	NEG	NEG	RARE	NEG	NEG	NEG	NEG	NEG	NEG	NEG	NEG	POS	NEG
ST779-MRSA	NEG	NEG	NEG	POS	RARE	RARE	NEG	NEG	NEG	NEG	NEG	NEG	NEG	NEG	NEG	NEG
ST834-MRSA-IV, WA-13	NEG	NEG	NEG	NEG	NEG	NEG	NEG	NEG	NEG	NEG	NEG	NEG	NEG	NEG	POS	NEG
CC913-MRSA-IV	POS	NEG	NEG	NEG	NEG	NEG	NEG	NEG	NEG	NEG	NEG	NEG	NEG	NEG	NEG	NEG
ST1774-MRSA-IV [ACME+]	NEG	NEG	NEG	NEG	NEG	NEG	NEG	NEG	NEG	NEG	NEG	NEG	NEG	NEG	NEG	NEG
CC15-MSSA	NEG	NEG	NEG	NEG	NEG	RARE	NEG	NEG	NEG	NEG	NEG	NEG	NEG	NEG	POS	NEG
ST426-MSSA	NEG	NEG	NEG	NEG	NEG	NEG	NEG	NEG	NEG	NEG	NEG	NEG	NEG	NEG	NEG	NEG

Legend:

Positive :	POS
Ambiguous :	AMB
Negative :	NEG
Rare (in >0 to 33% of isolates) :	RARE
Variable (in >33 to 66% of isolates) :	VAR
Common (in >66 to <100% of isolates) :	COMM

	RESISTANCE : EFFLUX SYSTEMS								RESISTANCE : GLYCOPEPTIDES		
	qacA	qacC (total)	qacC (cons)	qacC (equine)	qacC (SA5)	qacC (Ssap)	qacC (ST94)	tetEfflux	vanA	vanB	vanZ
CC1-MRSA-IV [PVL-], WA-1/57	NEG	RARE	RARE	NEG	RARE	NEG	NEG	POS	NEG	NEG	NEG
CC1-MRSA-IV [PVL+], USA400	NEG	RARE	RARE	NEG	NEG	NEG	NEG	POS	NEG	NEG	NEG
CC1-MRSA-IV/SCCfus [PVL-]	NEG	NEG	NEG	NEG	NEG	NEG	NEG	POS	NEG	NEG	NEG
CC1-MRSA-IV/SCCfus [PVL+]	NEG	NEG	NEG	NEG	NEG	NEG	NEG	POS	NEG	NEG	NEG
CC1-MRSA-V [PVL+]	NEG	NEG	NEG	NEG	NEG	NEG	NEG	POS	NEG	NEG	NEG
CC1-MRSA-V/SCCfus [PVL-]	NEG	NEG	NEG	NEG	NEG	NEG	NEG	POS	NEG	NEG	NEG
CC1-MRSA-V/SCCfus [PVL+]	NEG	NEG	NEG	NEG	NEG	NEG	NEG	POS	NEG	NEG	NEG
ST228-MRSA-I, South German/Italian MRSA	POS	NEG	NEG	NEG	NEG	NEG	NEG	POS	NEG	NEG	NEG
ST5-MRSA-I, Geraldine Clone	NEG	NEG	NEG	NEG	NEG	NEG	NEG	POS	NEG	NEG	NEG
CC5-MRSA-Ivar., WA-18/21/48	NEG	VAR	VAR	NEG	NEG	NEG	NEG	POS	NEG	NEG	NEG
CC5-MRSA-II, Rhine-Hesse /UK-3	RARE	RARE	RARE	NEG	NEG	NEG	NEG	POS	NEG	NEG	NEG
CC5-MRSA-II [ACME+]	NEG	NEG	NEG	NEG	NEG	NEG	NEG	POS	NEG	NEG	NEG
CC5-MRSA-III	NEG	NEG	NEG	NEG	NEG	NEG	NEG	POS	NEG	NEG	NEG
CC5-MRSA-IV, Paediatric clone	RARE	RARE	RARE	NEG	RARE	NEG	RARE	POS	NEG	NEG	NEG
CC5-MRSA-IV [PVL+]	NEG	NEG	NEG	NEG	NEG	NEG	NEG	POS	NEG	NEG	NEG
CC5-MRSA-V	RARE	RARE	RARE	NEG	NEG	NEG	NEG	POS	NEG	NEG	NEG
CC5-MRSA-V [PVL+]	NEG	NEG	NEG	NEG	NEG	NEG	NEG	POS	NEG	NEG	NEG
CC5-MRSA-VI, New Paediatric Clone	NEG	NEG	RARE	NEG	NEG	NEG	NEG	POS	NEG	NEG	NEG
ST5-MRSA-VII (SCC-JCSC6082)	NEG	NEG	NEG	NEG	NEG	NEG	NEG	POS	NEG	NEG	NEG
CC5-MRSA-IVvar, Maltese Clone	NEG	NEG	NEG	NEG	NEG	NEG	NEG	POS	NEG	NEG	NEG
CC5-MRSA-SCC(MRSAZH47)	NEG	NEG	NEG	NEG	NEG	NEG	NEG	POS	NEG	NEG	NEG
CC5-MRSA-IV/VI	RARE	NEG	RARE	NEG	NEG	NEG	NEG	POS	NEG	NEG	NEG
CC5-MRSA-IV/SCCfus	NEG	NEG	NEG	NEG	NEG	NEG	NEG	POS	NEG	NEG	NEG
CC5/ST835-MRSA, WA-40/46	NEG	POS	POS	NEG	NEG	NEG	NEG	POS	NEG	NEG	NEG
ST6-MRSA-IV, WA-51	NEG	NEG	NEG	NEG	NEG	NEG	NEG	POS	NEG	NEG	NEG
ST6-MRSA-V	NEG	NEG	NEG	NEG	NEG	NEG	NEG	POS	NEG	NEG	NEG
CC7-MRSA-IV	NEG	NEG	NEG	NEG	NEG	NEG	NEG	POS	NEG	NEG	NEG
CC7-MRSA-V	NEG	NEG	NEG	NEG	NEG	NEG	NEG	POS	NEG	NEG	NEG
ST250-MRSA-I, Early/Ancestral MRSA	COMM	NEG	NEG	NEG	NEG	NEG	NEG	POS	NEG	NEG	NEG
ST247-MRSA-I, North German/Iberian MRSA	COMM	NEG	NEG	NEG	NEG	NEG	NEG	POS	NEG	NEG	NEG
CC8-MRSA-IV, UK-14/WA-5	RARE	NEG	NEG	NEG	NEG	NEG	NEG	POS	NEG	NEG	NEG
CC8-MRSA-IV, Lyon Clone/UK-2	RARE	RARE	RARE	RARE	NEG	NEG	NEG	POS	NEG	NEG	NEG
CC8-MRSA-IV, UK-6	NEG	RARE	RARE	NEG	NEG	NEG	NEG	POS	NEG	NEG	NEG
CC8-MRSA-IV, USA500	RARE	RARE	NEG	RARE	RARE	NEG	RARE	POS	NEG	NEG	NEG
ST8-MRSA-IV [PVL/ACME+], USA300	NEG	NEG	NEG	NEG	NEG	NEG	NEG	POS	NEG	NEG	NEG
ST8-MRSA-IV [PVL+], USA300	NEG	NEG	NEG	NEG	NEG	NEG	NEG	POS	NEG	NEG	NEG
CC8-MRSA-IV [PVL/sed/j/r/k/q+]	NEG	NEG	NEG	NEG	NEG	NEG	NEG	POS	NEG	NEG	NEG
CC8-MRSA-IV [tst1/sec/sel+]	VAR	NEG	NEG	NEG	NEG	NEG	NEG	POS	NEG	NEG	NEG
CC8-MRSA-V	NEG	RARE	RARE	NEG	NEG	NEG	NEG	POS	NEG	NEG	NEG
CC8-MRSA-VIII	VAR	VAR	VAR	NEG	NEG	NEG	NEG	POS	NEG	NEG	NEG
ST8-MRSA-IIA/B/C/D/E, Irish AR13/14	COMM	RARE	RARE	NEG	NEG	NEG	NEG	POS	NEG	NEG	NEG
ST8-MRSA, UK-12/13, Irish AR43	NEG	NEG	NEG	NEG	NEG	NEG	NEG	POS	NEG	NEG	NEG
ST254-MRSA, Hannover EMRSA	NEG	NEG	NEG	NEG	NEG	NEG	NEG	POS	NEG	NEG	NEG
ST254-MRSA-IV/V, Hannover/UK-10	NEG	NEG	NEG	NEG	NEG	NEG	NEG	POS	NEG	NEG	NEG
CC9-MRSA-IV	NEG	VAR	NEG	NEG	NEG	NEG	VAR	POS	NEG	NEG	NEG
CC12-MRSA, WA-59	NEG	NEG	NEG	NEG	NEG	NEG	NEG	POS	NEG	NEG	NEG
CC12-MRSA-IV	NEG	NEG	NEG	NEG	NEG	NEG	NEG	POS	NEG	NEG	NEG
CC20-MRSA-V	NEG	NEG	NEG	NEG	NEG	NEG	NEG	POS	NEG	NEG	NEG
ST22-MRSA-IV, Barnim/UK-15	RARE	NEG	RARE	NEG	NEG	NEG	NEG	NEG	NEG	NEG	NEG
ST22-MRSA-IV [ACME+]	NEG	NEG	NEG	NEG	NEG	NEG	NEG	NEG	NEG	NEG	NEG
ST22-MRSA-IV [PVL+]	NEG	NEG	NEG	NEG	NEG	NEG	NEG	NEG	NEG	NEG	NEG
ST22-MRSA-V	NEG	NEG	NEG	NEG	NEG	NEG	NEG	NEG	NEG	NEG	NEG
ST22-MRSA-V [PVL+]	NEG	NEG	NEG	NEG	NEG	NEG	NEG	NEG	NEG	NEG	NEG
ST36-MRSA-II, UK-16	NEG	NEG	RARE	NEG	NEG	NEG	NEG	POS	NEG	NEG	NEG
ST30-MRSA-IV [PVL+], Southwest Pacific Clone	NEG	RARE	RARE	NEG	NEG	NEG	NEG	POS	NEG	NEG	NEG
ST30-MRSA-IV [tst1+]	NEG	NEG	NEG	NEG	NEG	NEG	NEG	POS	NEG	NEG	NEG
CC30-MRSA-V [PVL+]	NEG	NEG	NEG	NEG	NEG	NEG	NEG	POS	NEG	NEG	NEG
ST45-MRSA-II, USA600	NEG	RARE	RARE	NEG	NEG	NEG	NEG	POS	NEG	NEG	NEG
ST45-MRSA-IV, Berlin EMRSA	NEG	RARE	RARE	NEG	NEG	NEG	NEG	POS	NEG	NEG	NEG
CC45-MRSA-IV [ACME+]	NEG	NEG	NEG	NEG	NEG	NEG	NEG	POS	NEG	NEG	NEG
ST45-MRSA-V, WA-4	NEG	NEG	NEG	NEG	NEG	NEG	NEG	POS	NEG	NEG	NEG
CC45/agr IV-MRSA-IV, WA-23	NEG	NEG	NEG	NEG	NEG	NEG	NEG	POS	NEG	NEG	NEG
CC45/agr IV-MRSA-V	VAR	RARE	RARE	NEG	NEG	NEG	NEG	POS	NEG	NEG	NEG
CC45/agr IV-MRSA-IV/V	NEG	COMM	NEG	NEG	COMM	NEG	NEG	POS	NEG	NEG	NEG
CC59-MRSA-IV [PVL+], USA1000	NEG	NEG	NEG	NEG	NEG	NEG	NEG	POS	NEG	NEG	NEG
ST59-MRSA-IV, WA-73	NEG	NEG	NEG	NEG	NEG	NEG	NEG	POS	NEG	NEG	NEG
ST87-MRSA-IV, WA-24	NEG	NEG	NEG	NEG	NEG	NEG	NEG	POS	NEG	NEG	NEG
ST59-MRSA-IV [PVL+], WA-55/56	NEG	NEG	NEG	NEG	NEG	NEG	NEG	POS	NEG	NEG	NEG
ST59/ST952-MRSA-V(T) [PVL+], Taiwan Clone	NEG	NEG	NEG	NEG	NEG	NEG	NEG	POS	NEG	NEG	NEG
ST59-MRSA-V	NEG	NEG	NEG	NEG	NEG	NEG	NEG	POS	NEG	NEG	NEG
CC59-MRSA-V [PVL+]	NEG	NEG	NEG	NEG	NEG	NEG	NEG	POS	NEG	NEG	NEG
ST59-MRSA-IV/V, WA-15	NEG	NEG	NEG	NEG	NEG	NEG	NEG	POS	NEG	NEG	NEG
ST72-MRSA-IV, USA700	NEG	NEG	NEG	NEG	NEG	NEG	NEG	POS	NEG	NEG	NEG
ST72-MRSA-IV [PVL+], WA-44	NEG	NEG	NEG	NEG	NEG	NEG	NEG	POS	NEG	NEG	NEG
CC75-MRSA-IV	VAR	RARE	RARE	RARE	RARE	NEG	NEG	NEG	NEG	NEG	NEG
ST883-MRSA-IV, WA-47	NEG	VAR	VAR	NEG	NEG	NEG	NEG	NEG	NEG	NEG	NEG
ST1303-MRSA-IV	NEG	NEG	NEG	NEG	NEG	NEG	NEG	NEG	NEG	NEG	NEG
CC80-MRSA-IV	NEG	NEG	NEG	NEG	NEG	NEG	NEG	POS	NEG	NEG	NEG
CC80-MRSA-IV [PVL+], European Clone	NEG	NEG	NEG	NEG	NEG	NEG	NEG	POS	NEG	NEG	NEG
CC88-MRSA-IV, WA-2	NEG	RARE	RARE	NEG	NEG	NEG	NEG	POS	NEG	NEG	NEG
CC88-MRSA-IV [PVL+]	NEG	NEG	NEG	NEG	NEG	NEG	NEG	POS	NEG	NEG	NEG
CC88-MRSA-V [PVL+]	NEG	NEG	NEG	NEG	NEG	NEG	NEG	POS	NEG	NEG	NEG
CC88-MRSA-VI	NEG	NEG	NEG	NEG	NEG	NEG	NEG	POS	NEG	NEG	NEG
ST93-MRSA-IV [PVL-]	NEG	NEG	NEG	NEG	NEG	NEG	NEG	POS	NEG	NEG	NEG
ST93-MRSA-IV [PVL+], Queensland Clone	NEG	NEG	RARE	NEG	NEG	NEG	NEG	POS	NEG	NEG	NEG
ST93-MRSA-V [PVL+]	NEG	NEG	NEG	NEG	NEG	NEG	NEG	POS	NEG	NEG	NEG
CC97-MRSA-IV, WA-54	NEG	NEG	NEG	NEG	NEG	NEG	NEG	POS	NEG	NEG	NEG
CC97-MRSA-V	NEG	NEG	NEG	NEG	NEG	NEG	NEG	POS	NEG	NEG	NEG
CC97-MRSA-V [ACME+]	POS	NEG	NEG	NEG	NEG	NEG	NEG	POS	NEG	NEG	NEG
CC97-MRSA-I/V	NEG	NEG	NEG	NEG	NEG	NEG	NEG	POS	NEG	NEG	NEG
CC121-MRSA-V, WA-22	NEG	NEG	NEG	NEG	NEG	NEG	NEG	POS	NEG	NEG	NEG
CC152-MRSA-V [PVL+]	NEG	NEG	NEG	NEG	NEG	NEG	NEG	POS	NEG	NEG	NEG
ST154-MRSA [PVL+]	NEG	NEG	NEG	NEG	NEG	NEG	NEG	POS	NEG	NEG	NEG
ST188-MRSA-IV, WA-38/78	NEG	NEG	NEG	NEG	NEG	NEG	NEG	POS	NEG	NEG	NEG
ST188-MRSA-V	NEG	NEG	NEG	NEG	NEG	NEG	NEG	POS	NEG	NEG	NEG
ST239-MRSA-III	VAR	RARE	RARE	NEG	RARE	NEG	NEG	POS	NEG	NEG	NEG
CC361-MRSA-IV, WA-29	NEG	NEG	NEG	NEG	NEG	NEG	NEG	POS	NEG	NEG	NEG
CC361-MRSA-V	NEG	NEG	NEG	NEG	NEG	NEG	NEG	POS	NEG	NEG	NEG
CC361-MRSA-VIII, WA-28	NEG	NEG	NEG	NEG	NEG	NEG	NEG	POS	NEG	NEG	NEG
CC398-MRSA-IV	NEG	NEG	NEG	NEG	NEG	NEG	NEG	POS	NEG	NEG	NEG
ST398-MRSA-V	NEG	RARE	NEG	NEG	NEG	NEG	RARE	POS	NEG	NEG	NEG
ST398-MRSA-V [PVL+]	NEG	NEG	NEG	NEG	NEG	NEG	NEG	POS	NEG	NEG	NEG
CC509/ST207-MRSA-V	NEG	NEG	NEG	NEG	NEG	NEG	NEG	NEG	NEG	NEG	NEG
ST573-MRSA-V, WA-10	NEG	NEG	NEG	NEG	NEG	NEG	NEG	POS	NEG	NEG	NEG
ST772-MRSA-V [PVL+], WA-60/Bengal Bay Clone	NEG	NEG	NEG	NEG	NEG	NEG	NEG	POS	NEG	NEG	NEG
ST779-MRSA	NEG	NEG	NEG	NEG	NEG	NEG	NEG	POS	NEG	NEG	NEG
ST834-MRSA-IV, WA-13	NEG	NEG	NEG	NEG	NEG	NEG	NEG	POS	NEG	NEG	NEG
CC913-MRSA-IV	NEG	NEG	NEG	NEG	NEG	NEG	NEG	POS	NEG	NEG	NEG
ST1774-MRSA-IV [ACME+]	NEG	RARE	NEG	NEG	RARE	NEG	NEG	POS	NEG	NEG	NEG
CC15-MSSA	RARE	RARE	RARE	NEG	NEG	NEG	NEG	POS	NEG	NEG	NEG
ST426-MSSA	NEG	NEG	NEG	NEG	NEG	NEG	NEG	COMM	NEG	NEG	NEG

Legend:

Positive :	POS
Ambiguous :	AMB
Negative :	NEG
Rare (in >0 to 33% of isolates) :	RARE
Variable (in >33 to 66% of isolates) :	VAR
Common (in >66 to <100% of isolates) :	COMM

	VIRULENCE : TOXIC SCHOCK TOXIN			VIRULENCE : ENTEROTOXINS															
	tst1 (cons)	tst1 ("human" allele)	tst1 ("bovine" allele)	sea	sea (320E)	sea (N315) = sep	seb	sec	sed	see	seg	seh	sei	sej	sek	sel	sem	sen (cons)	sen (other than RF122)
CC1-MRSA-IV [PVL-], WA-1/57	NEG	NEG	NEG	VAR	NEG	RARE	RARE	NEG	NEG	NEG	NEG	POS	NEG	NEG	VAR	NEG	NEG	NEG	NEG
CC1-MRSA-IV [PVL+], USA400	NEG	NEG	NEG	COMM	NEG	NEG	RARE	RARE	NEG	NEG	NEG	POS	NEG	NEG	COMM	RARE	NEG	NEG	NEG
CC1-MRSA-IV/SCCfus [PVL-]	NEG	NEG	NEG	COMM	NEG	NEG	NEG	NEG	NEG	NEG	NEG	POS	NEG	NEG	COMM	NEG	NEG	NEG	NEG
CC1-MRSA-IV/SCCfus [PVL+]	NEG	NEG	NEG	POS	NEG	NEG	NEG	NEG	NEG	NEG	NEG	POS	NEG	NEG	POS	NEG	NEG	NEG	NEG
CC1-MRSA-V [PVL+]	NEG	NEG	NEG	POS	NEG	NEG	NEG	NEG	NEG	NEG	NEG	POS	NEG	NEG	POS	NEG	NEG	NEG	NEG
CC1-MRSA-V/SCCfus [PVL-]	NEG	NEG	NEG	VAR	NEG	NEG	NEG	NEG	NEG	NEG	NEG	POS	NEG	NEG	VAR	NEG	NEG	NEG	NEG
CC1-MRSA-V/SCCfus [PVL+]	NEG	NEG	NEG	NEG	NEG	NEG	NEG	NEG	NEG	NEG	NEG	POS	NEG	NEG	POS	NEG	NEG	NEG	NEG
ST228-MRSA-I, South German/Italian MRSA	RARE	RARE	NEG	COMM	NEG	NEG	NEG	NEG	NEG	NEG	COMM	NEG	COMM	NEG	NEG	NEG	COMM	COMM	COMM
ST5-MRSA-I, Geraldine Clone	COMM	COMM	NEG	RARE	NEG	NEG	NEG	COMM	COMM	NEG	POS	NEG	POS	POS	NEG	COMM	POS	POS	POS
CC5-MRSA-Ivar., WA-18/21/48	NEG	NEG	NEG	RARE	NEG	RARE	NEG	NEG	POS	NEG	POS	NEG	POS	POS	NEG	NEG	POS	POS	POS
CC5-MRSA-II, Rhine-Hesse /UK-3	RARE	RARE	NEG	RARE	NEG	COMM	NEG	RARE	COMM	NEG	POS	NEG	POS	COMM	NEG	RARE	POS	POS	POS
CC5-MRSA-II [ACME+]	POS	POS	NEG	NEG	NEG	POS	NEG	COMM	NEG	NEG	POS	NEG	POS	NEG	NEG	POS	POS	POS	POS
CC5-MRSA-III	NEG	NEG	NEG	VAR	NEG	NEG	NEG	NEG	NEG	NEG	POS	NEG	POS	NEG	NEG	NEG	POS	POS	POS
CC5-MRSA-IV, Paediatric clone	RARE	RARE	NEG	NEG	NEG	VAR	RARE	NEG	VAR	NEG	POS	NEG	POS	VAR	RARE	NEG	POS	POS	POS
CC5-MRSA-IV [PVL+]	NEG	NEG	NEG	RARE	NEG	RARE	NEG	NEG	RARE	NEG	POS	NEG	POS	RARE	NEG	NEG	POS	POS	POS
CC5-MRSA-V	NEG	NEG	NEG	NEG	NEG	VAR	NEG	NEG	VAR	NEG	POS	NEG	POS	VAR	NEG	NEG	POS	POS	POS
CC5-MRSA-V [PVL+]	NEG	NEG	NEG	NEG	NEG	VAR	COMM	NEG	VAR	NEG	POS	NEG	POS	VAR	NEG	NEG	POS	POS	POS
CC5-MRSA-VI, New Paediatric Clone	NEG	NEG	NEG	NEG	NEG	COMM	NEG	NEG	POS	NEG	POS	NEG	POS	POS	NEG	NEG	POS	POS	POS
ST5-MRSA-VII (SCC-JCSC6082)	NEG	NEG	NEG	NEG	NEG	NEG	NEG	NEG	NEG	NEG	POS	NEG	NEG	NEG	NEG	NEG	POS	POS	AMB
CC5-MRSA-IVvar, Maltese Clone	COMM	COMM	NEG	POS	NEG	NEG	NEG	COMM	NEG	NEG	POS	NEG	AMB	NEG	NEG	COMM	POS	POS	POS
CC5-MRSA-SCC(MRSAZH47)	NEG	NEG	NEG	NEG	NEG	NEG	NEG	NEG	NEG	NEG	POS	NEG	POS	NEG	NEG	NEG	POS	POS	POS
CC5-MRSA-IV/VI	NEG	NEG	NEG	NEG	NEG	VAR	NEG	NEG	RARE	NEG	POS	NEG	POS	RARE	NEG	NEG	POS	POS	POS
CC5-MRSA-IV/SCCfus	NEG	NEG	NEG	NEG	NEG	NEG	NEG	NEG	POS	NEG	POS	NEG	POS	POS	NEG	NEG	POS	POS	POS
CC5/ST835-MRSA, WA-40/46	NEG	NEG	NEG	NEG	NEG	NEG	NEG	NEG	COMM	NEG	POS	NEG	POS	COMM	NEG	NEG	POS	POS	POS
ST6-MRSA-IV, WA-51	NEG	NEG	NEG	POS	NEG	NEG	NEG	NEG	NEG	NEG	NEG	NEG	NEG	NEG	NEG	NEG	NEG	NEG	NEG
ST6-MRSA-V	NEG	NEG	NEG	NEG	NEG	NEG	NEG	NEG	NEG	NEG	NEG	NEG	NEG	NEG	NEG	NEG	NEG	NEG	NEG
CC7-MRSA-IV	NEG	NEG	NEG	NEG	NEG	POS	NEG	NEG	NEG	NEG	NEG	NEG	NEG	NEG	NEG	NEG	NEG	NEG	NEG
CC7-MRSA-V	NEG	NEG	NEG	NEG	NEG	POS	NEG	NEG	NEG	NEG	NEG	NEG	NEG	NEG	NEG	NEG	NEG	NEG	NEG
ST250-MRSA-I, Early/Ancestral MRSA	NEG	NEG	NEG	NEG	NEG	NEG	COMM	NEG	NEG	NEG	NEG	NEG	NEG	NEG	COMM	NEG	NEG	NEG	NEG
ST247-MRSA-I, North German/Iberian MRSA	NEG	NEG	NEG	VAR	NEG	NEG	RARE	NEG	NEG	NEG	NEG	NEG	NEG	NEG	RARE	NEG	NEG	NEG	NEG
CC8-MRSA-IV, UK-14/WA-5	NEG	NEG	NEG	NEG	NEG	NEG	NEG	NEG	NEG	NEG	NEG	NEG	NEG	NEG	NEG	NEG	NEG	NEG	NEG
CC8-MRSA-IV, Lyon Clone/UK-2	NEG	NEG	NEG	COMM	NEG	NEG	NEG	NEG	VAR	NEG	NEG	NEG	NEG	VAR	NEG	NEG	NEG	NEG	NEG
CC8-MRSA-IV, UK-6	NEG	NEG	NEG	POS	NEG	NEG	NEG	NEG	NEG	NEG	NEG	NEG	NEG	NEG	NEG	NEG	NEG	NEG	NEG
CC8-MRSA-IV, USA500	NEG	NEG	NEG	COMM	NEG	NEG	POS	NEG	NEG	NEG	NEG	NEG	NEG	NEG	POS	NEG	NEG	NEG	NEG
ST8-MRSA-IV [PVL/ACME+], USA300	NEG	NEG	NEG	NEG	NEG	NEG	NEG	NEG	NEG	NEG	NEG	NEG	NEG	NEG	COMM	NEG	NEG	NEG	NEG
ST8-MRSA-IV [PVL+], USA300	NEG	NEG	NEG	NEG	NEG	NEG	NEG	NEG	NEG	NEG	NEG	NEG	NEG	NEG	COMM	NEG	NEG	NEG	NEG
CC8-MRSA-IV [PVL/sed/j/r/k/q+]	NEG	NEG	NEG	NEG	NEG	NEG	NEG	NEG	POS	NEG	NEG	NEG	NEG	POS	POS	NEG	NEG	NEG	NEG
CC8-MRSA-IV [tst1/sec/sel+]	POS	POS	NEG	NEG	NEG	NEG	NEG	POS	NEG	NEG	NEG	NEG	NEG	NEG	NEG	POS	NEG	NEG	NEG
CC8-MRSA-V	NEG	NEG	NEG	VAR	NEG	NEG	VAR	NEG	NEG	NEG	NEG	NEG	NEG	NEG	VAR	NEG	NEG	NEG	NEG
CC8-MRSA-VIII	NEG	NEG	NEG	POS	NEG	NEG	NEG	NEG	NEG	NEG	NEG	NEG	NEG	NEG	NEG	NEG	NEG	NEG	NEG
ST8-MRSA-IIA/B/C/D/E, Irish AR13/14	NEG	NEG	NEG	COMM	NEG	NEG	NEG	NEG	NEG	NEG	NEG	NEG	NEG	NEG	NEG	NEG	NEG	NEG	NEG
ST8-MRSA, UK-12/13, Irish AR43	NEG	NEG	NEG	RARE	NEG	NEG	NEG	NEG	RARE	NEG	NEG	NEG	NEG	RARE	NEG	NEG	NEG	NEG	NEG
ST254-MRSA, Hannover EMRSA	NEG	NEG	NEG	RARE	NEG	NEG	POS	NEG	NEG	NEG	NEG	NEG	NEG	NEG	POS	NEG	NEG	NEG	NEG
ST254-MRSA-IV/V, Hannover/UK-10	NEG	NEG	NEG	VAR	NEG	NEG	POS	NEG	NEG	NEG	NEG	NEG	NEG	NEG	POS	NEG	NEG	NEG	NEG
CC9-MRSA-IV	NEG	NEG	NEG	NEG	NEG	NEG	NEG	NEG	NEG	NEG	POS	NEG	POS	NEG	NEG	NEG	POS	POS	POS
CC12-MRSA, WA-59	NEG	NEG	NEG	NEG	NEG	POS	POS	NEG	NEG	NEG	NEG	NEG	NEG	NEG	NEG	NEG	NEG	NEG	NEG
CC12-MRSA-IV	NEG	NEG	NEG	NEG	NEG	POS	POS	NEG	NEG	NEG	NEG	NEG	NEG	NEG	NEG	NEG	NEG	NEG	NEG
CC20-MRSA-V	NEG	NEG	NEG	NEG	NEG	NEG	NEG	NEG	NEG	NEG	POS	NEG	POS	NEG	NEG	NEG	POS	POS	POS
ST22-MRSA-IV, Barnim/UK-15	RARE	RARE	NEG	NEG	NEG	NEG	NEG	RARE	NEG	NEG	POS	NEG	POS	NEG	NEG	RARE	POS	POS	POS
ST22-MRSA-IV [ACME+]	NEG	NEG	NEG	NEG	NEG	NEG	NEG	NEG	NEG	NEG	POS	NEG	POS	NEG	NEG	NEG	POS	POS	POS
ST22-MRSA-IV [PVL+]	NEG	NEG	NEG	NEG	NEG	NEG	NEG	NEG	NEG	NEG	POS	NEG	POS	NEG	NEG	NEG	POS	POS	POS
ST22-MRSA-V	NEG	NEG	NEG	NEG	NEG	NEG	NEG	POS	NEG	NEG	POS	NEG	POS	NEG	NEG	POS	POS	POS	POS
ST22-MRSA-V [PVL+]	NEG	NEG	NEG	NEG	NEG	NEG	NEG	NEG	NEG	NEG	POS	NEG	POS	NEG	NEG	NEG	POS	POS	POS
ST36-MRSA-II, UK-16	COMM	COMM	NEG	COMM	NEG	NEG	NEG	RARE	NEG	NEG	POS	NEG	POS	NEG	NEG	RARE	POS	POS	POS
ST30-MRSA-IV [PVL+], Southwest Pacific Clone	RARE	RARE	NEG	RARE	NEG	NEG	NEG	RARE	NEG	NEG	POS	NEG	POS	NEG	NEG	RARE	POS	POS	POS
ST30-MRSA-IV [tst1+]	POS	POS	NEG	RARE	NEG	NEG	NEG	NEG	NEG	NEG	POS	NEG	POS	NEG	NEG	NEG	POS	POS	POS
CC30-MRSA-V [PVL+]	NEG	NEG	NEG	NEG	NEG	NEG	NEG	NEG	NEG	NEG	POS	NEG	NEG	NEG	NEG	NEG	POS	POS	POS
ST45-MRSA-II, USA600	NEG	NEG	NEG	NEG	NEG	NEG	NEG	NEG	NEG	NEG	POS	NEG	POS	NEG	NEG	NEG	POS	POS	POS
ST45-MRSA-IV, Berlin EMRSA	NEG	NEG	NEG	NEG	NEG	NEG	NEG	RARE	NEG	NEG	COMM	NEG	POS	NEG	NEG	RARE	POS	POS	POS
CC45-MRSA-IV [ACME+]	COMM	COMM	NEG	NEG	NEG	NEG	NEG	POS	NEG	NEG	POS	NEG	POS	NEG	NEG	POS	POS	POS	POS
ST45-MRSA-V, WA-4	COMM	COMM	NEG	NEG	NEG	NEG	NEG	RARE	NEG	NEG	POS	NEG	POS	NEG	COMM	RARE	POS	POS	POS
CC45/agr IV-MRSA-IV, WA-23	NEG	NEG	NEG	NEG	NEG	NEG	NEG	NEG	NEG	NEG	POS	NEG	POS	COMM	NEG	NEG	POS	POS	POS
CC45/agr IV-MRSA-V	NEG	NEG	NEG	NEG	NEG	NEG	NEG	NEG	NEG	NEG	POS	NEG	POS	POS	NEG	NEG	POS	POS	POS
CC45/agr IV-MRSA-IV/V	NEG	NEG	NEG	NEG	NEG	NEG	NEG	NEG	NEG	NEG	POS	NEG	POS	POS	NEG	NEG	POS	POS	POS
CC59-MRSA-IV [PVL+], USA1000	NEG	NEG	NEG	NEG	NEG	NEG	COMM	NEG	NEG	NEG	NEG	NEG	NEG	NEG	COMM	NEG	NEG	NEG	NEG
ST59-MRSA-IV, WA-73	NEG	NEG	NEG	NEG	NEG	NEG	POS	NEG	NEG	NEG	NEG	NEG	NEG	NEG	POS	NEG	NEG	NEG	NEG
ST87-MRSA-IV, WA-24	NEG	NEG	NEG	NEG	NEG	NEG	POS	NEG	NEG	NEG	NEG	NEG	NEG	NEG	POS	NEG	NEG	NEG	NEG
ST59-MRSA-IV [PVL+], WA-55/56	NEG	NEG	NEG	RARE	NEG	NEG	COMM	NEG	NEG	NEG	NEG	NEG	NEG	NEG	COMM	NEG	NEG	NEG	NEG
ST59/ST952-MRSA-V(T) [PVL+], Taiwan Clone	NEG	NEG	NEG	NEG	NEG	NEG	COMM	NEG	NEG	NEG	NEG	NEG	NEG	NEG	COMM	NEG	NEG	NEG	NEG
ST59-MRSA-V	NEG	NEG	NEG	NEG	NEG	NEG	POS	NEG	NEG	NEG	NEG	NEG	NEG	NEG	POS	NEG	NEG	NEG	NEG
CC59-MRSA-V [PVL+]	NEG	NEG	NEG	NEG	NEG	NEG	POS	NEG	NEG	NEG	NEG	NEG	NEG	NEG	POS	NEG	NEG	NEG	NEG
ST59-MRSA-IV/V, WA-15	NEG	NEG	NEG	POS	NEG	NEG	COMM	NEG	NEG	NEG	NEG	NEG	NEG	NEG	COMM	NEG	NEG	NEG	NEG
ST72-MRSA-IV, USA700	NEG	NEG	NEG	NEG	NEG	NEG	NEG	NEG	NEG	NEG	POS	NEG	POS	NEG	NEG	NEG	POS	POS	POS
ST72-MRSA-IV [PVL+], WA-44	NEG	NEG	NEG	NEG	NEG	NEG	NEG	POS	NEG	NEG	POS	NEG	POS	NEG	NEG	POS	POS	POS	POS
CC75-MRSA-IV	NEG	NEG	NEG	NEG	NEG	NEG	VAR	NEG	RARE	NEG	POS	NEG	POS	RARE	NEG	NEG	POS	AMB	NEG
ST883-MRSA-IV, WA-47	NEG	NEG	NEG	NEG	NEG	NEG	NEG	NEG	NEG	NEG	NEG	NEG	NEG	NEG	NEG	NEG	NEG	NEG	NEG
ST1303-MRSA-IV	NEG	NEG	NEG	NEG	NEG	NEG	POS	NEG	NEG	NEG	AMB	NEG	POS	NEG	NEG	NEG	POS	NEG	AMB
CC80-MRSA-IV	NEG	NEG	NEG	NEG	NEG	NEG	NEG	NEG	NEG	NEG	NEG	NEG	NEG	NEG	NEG	NEG	NEG	NEG	NEG
CC80-MRSA-IV [PVL+], European Clone	NEG	NEG	NEG	NEG	NEG	NEG	NEG	NEG	NEG	NEG	NEG	NEG	NEG	NEG	NEG	NEG	NEG	NEG	NEG
CC88-MRSA-IV, WA-2	NEG	NEG	NEG	NEG	NEG	NEG	NEG	COMM	NEG	NEG	NEG	NEG	NEG	NEG	NEG	COMM	NEG	NEG	NEG
CC88-MRSA-IV [PVL+]	NEG	NEG	NEG	NEG	NEG	COMM	NEG	RARE	NEG	NEG	NEG	NEG	NEG	NEG	VAR	RARE	NEG	NEG	NEG
CC88-MRSA-V [PVL+]	NEG	NEG	NEG	NEG	NEG	POS	POS	NEG	NEG	NEG	NEG	NEG	NEG	NEG	POS	NEG	NEG	NEG	NEG
CC88-MRSA-VI	NEG	NEG	NEG	NEG	NEG	NEG	NEG	POS	NEG	NEG	NEG	NEG	NEG	NEG	NEG	POS	NEG	NEG	NEG
ST93-MRSA-IV [PVL-]	NEG	NEG	NEG	NEG	NEG	NEG	NEG	NEG	NEG	NEG	NEG	NEG	NEG	NEG	NEG	NEG	NEG	NEG	NEG
ST93-MRSA-IV [PVL+], Queensland Clone	NEG	NEG	NEG	NEG	NEG	NEG	NEG	NEG	NEG	NEG	NEG	NEG	NEG	NEG	NEG	NEG	NEG	NEG	NEG
ST93-MRSA-V [PVL+]	NEG	NEG	NEG	NEG	NEG	NEG	NEG	NEG	NEG	NEG	NEG	NEG	NEG	NEG	NEG	NEG	NEG	NEG	NEG
CC97-MRSA-IV, WA-54	NEG	NEG	NEG	NEG	NEG	NEG	NEG	RARE	NEG	NEG	NEG	NEG	NEG	NEG	NEG	RARE	NEG	NEG	NEG
CC97-MRSA-V	NEG	NEG	NEG	NEG	NEG	NEG	NEG	NEG	NEG	NEG	NEG	NEG	NEG	NEG	NEG	NEG	NEG	NEG	NEG
CC97-MRSA-V [ACME+]	NEG	NEG	NEG	NEG	NEG	NEG	NEG	NEG	POS	NEG	AMB	NEG	NEG	POS	NEG	NEG	NEG	NEG	NEG
CC97-MRSA-I/V	NEG	NEG	NEG	NEG	NEG	NEG	NEG	NEG	NEG	NEG	NEG	NEG	NEG	NEG	NEG	NEG	NEG	NEG	NEG
CC121-MRSA-V, WA-22	NEG	NEG	NEG	NEG	NEG	NEG	NEG	NEG	NEG	NEG	POS	NEG	POS	NEG	NEG	NEG	POS	POS	POS
CC152-MRSA-V [PVL+]	NEG	NEG	NEG	NEG	NEG	NEG	NEG	NEG	NEG	NEG	NEG	NEG	NEG	NEG	NEG	NEG	NEG	NEG	NEG
ST154-MRSA [PVL+]	NEG	NEG	NEG	NEG	NEG	NEG	NEG	VAR	NEG	NEG	NEG	NEG	NEG	NEG	NEG	VAR	NEG	NEG	NEG
ST188-MRSA-IV, WA-38/78	NEG	NEG	NEG	NEG	NEG	NEG	NEG	NEG	NEG	NEG	NEG	NEG	NEG	NEG	NEG	NEG	NEG	NEG	NEG
ST188-MRSA-V	NEG	NEG	NEG	NEG	NEG	NEG	NEG	NEG	NEG	NEG	NEG	NEG	NEG	NEG	NEG	NEG	NEG	NEG	NEG
ST239-MRSA-III	NEG	NEG	NEG	VAR	NEG	NEG	NEG	NEG	NEG	NEG	NEG	NEG	NEG	NEG	COMM	NEG	NEG	NEG	NEG
CC361-MRSA-IV, WA-29	VAR	VAR	NEG	NEG	NEG	NEG	VAR	NEG	NEG	NEG	POS	NEG	COMM	NEG	NEG	NEG	COMM	COMM	COMM
CC361-MRSA-V	NEG	NEG	NEG	NEG	NEG	NEG	NEG	NEG	NEG	NEG	POS	NEG	POS	NEG	NEG	NEG	POS	POS	POS
CC361-MRSA-VIII, WA-28	NEG	NEG	NEG	NEG	NEG	NEG	NEG	NEG	NEG	NEG	POS	NEG	POS	NEG	NEG	NEG	POS	POS	AMB
CC398-MRSA-IV	NEG	NEG	NEG	NEG	NEG	NEG	NEG	NEG	NEG	NEG	NEG	NEG	NEG	NEG	NEG	NEG	NEG	NEG	NEG
ST398-MRSA-V	NEG	NEG	NEG	RARE	NEG	NEG	RARE	NEG	NEG	NEG	NEG	NEG	NEG	NEG	RARE	NEG	NEG	NEG	NEG
ST398-MRSA-V [PVL+]	NEG	NEG	NEG	VAR	NEG	NEG	NEG	NEG	NEG	NEG	NEG	NEG	NEG	NEG	NEG	NEG	NEG	NEG	NEG
CC509/ST207-MRSA-V	NEG	NEG	NEG	NEG	NEG	NEG	NEG	NEG	NEG	NEG	NEG	NEG	NEG	NEG	NEG	NEG	POS	NEG	NEG
ST573-MRSA-V, WA-10	NEG	NEG	NEG	VAR	NEG	NEG	NEG	POS	NEG	NEG	POS	NEG	POS	NEG	NEG	POS	POS	POS	POS
ST772-MRSA-V [PVL+], WA-60/Bengal Bay Clone	NEG	NEG	NEG	POS	NEG	NEG	NEG	POS	RARE	NEG	COMM	NEG	POS	NEG	NEG	POS	POS	POS	POS
ST779-MRSA	NEG	NEG	NEG	NEG	NEG	NEG	COMM	NEG	RARE	NEG	NEG	NEG	NEG	RARE	NEG	NEG	NEG	NEG	NEG
ST834-MRSA-IV, WA-13	POS	POS	NEG	NEG	NEG	NEG	NEG	POS	NEG	NEG	NEG	NEG	NEG	NEG	NEG	POS	NEG	NEG	NEG
CC913-MRSA-IV	NEG	NEG	NEG	NEG	NEG	NEG	NEG	NEG	NEG	NEG	NEG	NEG	NEG	NEG	NEG	NEG	NEG	NEG	NEG
ST1774-MRSA-IV [ACME+]	NEG	NEG	NEG	NEG	NEG	NEG	NEG	NEG	NEG	NEG	NEG	NEG	NEG	NEG	NEG	NEG	NEG	NEG	NEG
CC15-MSSA	NEG	NEG	NEG	RARE	NEG	NEG	NEG	NEG	NEG	NEG	NEG	NEG	NEG	NEG	NEG	NEG	NEG	NEG	NEG
ST426-MSSA	POS	POS	NEG	POS	NEG	NEG	NEG	COMM	NEG	RARE	NEG	NEG	NEG	NEG	RARE	COMM	NEG	NEG	NEG

Legend:

Positive :	POS
Ambiguous :	AMB
Negative :	NEG
Rare (in >0 to 33% of isolates) :	RARE
Variable (in >33 to 66% of isolates) :	VAR
Common (in >66 to <100% of isolates) :	COMM

	VIRULENCE : ENTEROTOXINS, continued						VIRULENCE : HLG AND LEUKOCIDINS												
	seo	seq	ser	seu	ORF CM14 probe1	ORF CM14 probe2	lukF	lukS	lukS (ST22+45)	hlgA	lukF-PV	lukS-PV	lukF-PV (P83)	lukM	lukD	lukE	lukX	lukY	lukY (ST30+45)
CC1-MRSA-IV [PVL-], WA-1/57	NEG	VAR	NEG	NEG	NEG	NEG	POS	POS	AMB	AMB	NEG	NEG	NEG	NEG	POS	POS	POS	POS	NEG
CC1-MRSA-IV [PVL+], USA400	NEG	COMM	NEG	NEG	NEG	NEG	POS	POS	AMB	POS	POS	POS	NEG	NEG	POS	POS	POS	POS	NEG
CC1-MRSA-IV/SCCfus [PVL-]	NEG	COMM	NEG	NEG	NEG	NEG	POS	POS	AMB	POS	NEG	NEG	NEG	NEG	POS	POS	POS	POS	NEG
CC1-MRSA-IV/SCCfus [PVL+]	NEG	POS	NEG	NEG	NEG	NEG	POS	POS	AMB	AMB	POS	POS	NEG	NEG	POS	POS	AMB	POS	NEG
CC1-MRSA-V [PVL+]	NEG	POS	NEG	NEG	NEG	NEG	POS	POS	AMB	POS	POS	POS	NEG	NEG	POS	POS	POS	POS	NEG
CC1-MRSA-V/SCCfus [PVL-]	NEG	VAR	NEG	NEG	NEG	NEG	POS	POS	AMB	POS	NEG	NEG	NEG	NEG	POS	POS	POS	POS	NEG
CC1-MRSA-V/SCCfus [PVL+]	NEG	POS	NEG	NEG	NEG	NEG	POS	POS	AMB	POS	POS	POS	NEG	NEG	POS	POS	POS	POS	NEG
ST228-MRSA-I, South German/Italian MRSA	COMM	NEG	NEG	COMM	NEG	NEG	POS	POS	AMB	POS	NEG	NEG	NEG	NEG	COMM	COMM	POS	POS	NEG
ST5-MRSA-I, Geraldine Clone	POS	NEG	POS	POS	NEG	NEG	POS	POS	AMB	POS	NEG	NEG	NEG	NEG	POS	POS	POS	POS	NEG
CC5-MRSA-Ivar., WA-18/21/48	POS	NEG	POS	POS	NEG	NEG	POS	POS	AMB	POS	NEG	NEG	NEG	NEG	POS	POS	POS	POS	NEG
CC5-MRSA-II, Rhine-Hesse /UK-3	POS	NEG	VAR	POS	NEG	NEG	POS	POS	AMB	POS	NEG	NEG	NEG	NEG	COMM	POS	POS	POS	NEG
CC5-MRSA-II [ACME+]	POS	NEG	NEG	POS	NEG	NEG	POS	POS	POS	POS	NEG	NEG	NEG	NEG	POS	POS	POS	POS	NEG
CC5-MRSA-III	POS	NEG	NEG	POS	NEG	NEG	POS	POS	AMB	POS	NEG	NEG	NEG	NEG	POS	POS	POS	POS	NEG
CC5-MRSA-IV, Paediatric clone	POS	RARE	RARE	POS	NEG	NEG	POS	POS	AMB	POS	NEG	NEG	NEG	NEG	POS	POS	POS	POS	NEG
CC5-MRSA-IV [PVL+]	POS	NEG	RARE	POS	NEG	NEG	POS	POS	AMB	POS	POS	POS	NEG	NEG	POS	POS	POS	POS	NEG
CC5-MRSA-V	POS	NEG	VAR	POS	NEG	NEG	POS	POS	AMB	POS	NEG	NEG	NEG	NEG	POS	POS	POS	POS	NEG
CC5-MRSA-V [PVL+]	POS	NEG	VAR	POS	NEG	NEG	POS	POS	AMB	POS	POS	POS	NEG	NEG	POS	POS	AMB	POS	NEG
CC5-MRSA-VI, New Paediatric Clone	POS	NEG	POS	POS	NEG	NEG	POS	POS	AMB	POS	NEG	NEG	NEG	NEG	POS	POS	POS	POS	NEG
ST5-MRSA-VII (SCC-JCSC6082)	NEG	NEG	NEG	POS	NEG	NEG	POS	POS	NEG	POS	NEG	NEG	NEG	NEG	POS	POS	AMB	POS	NEG
CC5-MRSA-IVvar, Maltese Clone	POS	NEG	NEG	POS	NEG	NEG	POS	POS	AMB	POS	NEG	NEG	NEG	NEG	POS	POS	POS	POS	NEG
CC5-MRSA-SCC(MRSAZH47)	POS	NEG	NEG	POS	NEG	NEG	POS	POS	AMB	POS	NEG	NEG	NEG	NEG	POS	POS	POS	POS	NEG
CC5-MRSA-IV/VI	POS	NEG	RARE	POS	NEG	NEG	POS	POS	AMB	POS	NEG	NEG	NEG	NEG	POS	POS	POS	POS	NEG
CC5-MRSA-IV/SCCfus	POS	NEG	POS	POS	NEG	NEG	POS	POS	AMB	POS	NEG	NEG	NEG	NEG	POS	POS	POS	POS	NEG
CC5/ST835-MRSA, WA-40/46	POS	NEG	COMM	POS	NEG	NEG	POS	POS	AMB	POS	NEG	NEG	NEG	NEG	POS	POS	AMB	POS	NEG
ST6-MRSA-IV, WA-51	NEG	NEG	NEG	NEG	NEG	NEG	AMB	POS	NEG	AMB	NEG	NEG	NEG	NEG	POS	AMB	AMB	POS	NEG
ST6-MRSA-V	NEG	NEG	NEG	NEG	NEG	NEG	POS	POS	POS	POS	NEG	NEG	NEG	NEG	POS	POS	POS	POS	NEG
CC7-MRSA-IV	NEG	NEG	NEG	NEG	NEG	NEG	POS	NEG	POS	POS	NEG	NEG	NEG	NEG	POS	POS	POS	POS	NEG
CC7-MRSA-V	NEG	NEG	NEG	NEG	NEG	NEG	POS	NEG	POS	POS	NEG	NEG	NEG	NEG	POS	POS	POS	POS	NEG
ST250-MRSA-I, Early/Ancestral MRSA	NEG	COMM	NEG	NEG	NEG	NEG	POS	POS	NEG	POS	NEG	NEG	NEG	NEG	POS	POS	VAR	COMM	NEG
ST247-MRSA-I, North German/Iberian MRSA	NEG	RARE	NEG	NEG	NEG	NEG	POS	POS	AMB	POS	NEG	NEG	NEG	NEG	POS	POS	POS	POS	NEG
CC8-MRSA-IV, UK-14/WA-5	NEG	NEG	NEG	NEG	NEG	NEG	POS	POS	AMB	POS	NEG	NEG	NEG	NEG	POS	POS	POS	POS	NEG
CC8-MRSA-IV, Lyon Clone/UK-2	NEG	NEG	VAR	NEG	NEG	NEG	POS	POS	AMB	POS	NEG	NEG	NEG	NEG	POS	POS	POS	POS	NEG
CC8-MRSA-IV, UK-6	NEG	NEG	NEG	NEG	NEG	NEG	POS	POS	AMB	POS	NEG	NEG	NEG	NEG	POS	POS	POS	POS	NEG
CC8-MRSA-IV, USA500	NEG	POS	NEG	NEG	NEG	NEG	POS	POS	AMB	POS	NEG	NEG	NEG	NEG	POS	POS	POS	POS	NEG
ST8-MRSA-IV [PVL/ACME+], USA300	NEG	COMM	NEG	NEG	NEG	NEG	POS	POS	AMB	POS	POS	POS	NEG	NEG	POS	POS	POS	POS	NEG
ST8-MRSA-IV [PVL+], USA300	NEG	COMM	NEG	NEG	NEG	NEG	POS	POS	NEG	POS	POS	POS	NEG	NEG	POS	POS	POS	POS	NEG
CC8-MRSA-IV [PVL/sed/j/r/k/q+]	NEG	POS	POS	NEG	NEG	NEG	POS	POS	AMB	POS	POS	POS	NEG	NEG	POS	POS	POS	POS	NEG
CC8-MRSA-IV [tst1/sec/sel+]	NEG	NEG	NEG	NEG	NEG	NEG	POS	AMB	POS	POS	NEG	NEG	NEG	NEG	POS	POS	POS	POS	NEG
CC8-MRSA-V	NEG	VAR	NEG	NEG	NEG	NEG	POS	POS	AMB	POS	NEG	NEG	NEG	NEG	POS	POS	POS	POS	NEG
CC8-MRSA-VIII	NEG	NEG	NEG	NEG	NEG	NEG	POS	POS	NEG	POS	NEG	NEG	NEG	NEG	POS	POS	POS	POS	NEG
ST8-MRSA-IIA/B/C/D/E, Irish AR13/14	NEG	NEG	NEG	NEG	NEG	NEG	POS	POS	AMB	POS	NEG	NEG	NEG	NEG	POS	POS	AMB	POS	NEG
ST8-MRSA, UK-12/13, Irish AR43	NEG	NEG	RARE	NEG	NEG	NEG	POS	POS	AMB	POS	NEG	NEG	NEG	NEG	POS	POS	POS	POS	NEG
ST254-MRSA, Hannover EMRSA	NEG	POS	NEG	NEG	NEG	NEG	POS	POS	AMB	POS	NEG	NEG	NEG	NEG	POS	POS	POS	POS	NEG
ST254-MRSA-IV/V, Hannover/UK-10	NEG	POS	NEG	NEG	NEG	NEG	POS	POS	AMB	POS	NEG	NEG	NEG	NEG	POS	POS	POS	POS	NEG
CC9-MRSA-IV	POS	NEG	NEG	POS	NEG	NEG	POS	POS	AMB	POS	NEG	NEG	NEG	NEG	NEG	NEG	POS	POS	NEG
CC12-MRSA, WA-59	NEG	NEG	NEG	NEG	POS	POS	POS	POS	NEG	POS	NEG	NEG	NEG	NEG	POS	POS	POS	POS	NEG
CC12-MRSA-IV	NEG	NEG	NEG	NEG	POS	POS	POS	POS	AMB	POS	NEG	NEG	NEG	NEG	POS	POS	POS	POS	NEG
CC20-MRSA-V	POS	NEG	NEG	POS	NEG	NEG	POS	POS	AMB	POS	NEG	NEG	NEG	NEG	NEG	POS	POS	POS	NEG
ST22-MRSA-IV, Barnim/UK-15	POS	NEG	NEG	POS	NEG	NEG	AMB	NEG	POS	AMB	NEG	NEG	NEG	NEG	NEG	NEG	POS	POS	NEG
ST22-MRSA-IV [ACME+]	POS	NEG	NEG	POS	NEG	NEG	POS	AMB	POS	AMB	NEG	NEG	NEG	NEG	NEG	NEG	POS	POS	NEG
ST22-MRSA-IV [PVL+]	POS	NEG	NEG	POS	NEG	NEG	AMB	AMB	POS	POS	POS	POS	NEG	NEG	NEG	NEG	POS	POS	NEG
ST22-MRSA-V	POS	NEG	NEG	POS	NEG	NEG	AMB	AMB	POS	AMB	NEG	NEG	NEG	NEG	NEG	NEG	POS	POS	NEG
ST22-MRSA-V [PVL+]	POS	NEG	NEG	POS	NEG	NEG	AMB	NEG	POS	AMB	POS	POS	NEG	NEG	NEG	NEG	POS	POS	NEG
ST36-MRSA-II, UK-16	POS	NEG	NEG	POS	NEG	NEG	POS	POS	AMB	POS	NEG	NEG	NEG	NEG	NEG	NEG	POS	NEG	POS
ST30-MRSA-IV [PVL+], Southwest Pacific Clone	POS	NEG	NEG	POS	NEG	NEG	POS	POS	AMB	POS	POS	POS	NEG	NEG	NEG	NEG	POS	NEG	POS
ST30-MRSA-IV [tst1+]	POS	NEG	NEG	POS	NEG	NEG	POS	POS	AMB	POS	NEG	NEG	NEG	NEG	NEG	NEG	AMB	NEG	POS
CC30-MRSA-V [PVL+]	POS	NEG	NEG	POS	NEG	NEG	POS	POS	AMB	POS	POS	POS	NEG	NEG	NEG	NEG	POS	NEG	POS
ST45-MRSA-II, USA600	POS	NEG	NEG	POS	NEG	NEG	POS	AMB	POS	POS	NEG	NEG	NEG	NEG	NEG	NEG	POS	NEG	POS
ST45-MRSA-IV, Berlin EMRSA	POS	NEG	NEG	POS	NEG	NEG	POS	NEG	POS	POS	NEG	NEG	NEG	NEG	NEG	NEG	POS	NEG	POS
CC45-MRSA-IV [ACME+]	POS	NEG	NEG	POS	NEG	NEG	POS	AMB	POS	POS	NEG	NEG	NEG	NEG	NEG	NEG	POS	NEG	POS
ST45-MRSA-V, WA-4	POS	COMM	NEG	POS	NEG	NEG	POS	AMB	POS	POS	NEG	NEG	NEG	NEG	NEG	NEG	POS	NEG	POS
CC45/agr IV-MRSA-IV, WA-23	POS	NEG	AMB	POS	NEG	NEG	POS	AMB	POS	POS	NEG	NEG	NEG	NEG	NEG	NEG	POS	NEG	POS
CC45/agr IV-MRSA-V	POS	NEG	AMB	POS	NEG	NEG	POS	AMB	POS	POS	NEG	NEG	NEG	NEG	NEG	NEG	POS	NEG	POS
CC45/agr IV-MRSA-IV/V	POS	NEG	POS	POS	NEG	NEG	POS	AMB	POS	POS	NEG	NEG	NEG	NEG	NEG	NEG	POS	NEG	POS
CC59-MRSA-IV [PVL+], USA1000	NEG	COMM	NEG	NEG	NEG	NEG	POS	POS	AMB	POS	POS	POS	NEG	NEG	NEG	NEG	POS	POS	NEG
ST59-MRSA-IV, WA-73	NEG	POS	NEG	NEG	NEG	NEG	POS	POS	AMB	POS	NEG	NEG	NEG	NEG	NEG	NEG	AMB	POS	NEG
ST87-MRSA-IV, WA-24	NEG	POS	NEG	NEG	NEG	NEG	POS	POS	NEG	POS	NEG	NEG	NEG	NEG	NEG	NEG	POS	POS	NEG
ST59-MRSA-IV [PVL+], WA-55/56	NEG	COMM	NEG	NEG	NEG	NEG	POS	POS	AMB	POS	POS	POS	NEG	NEG	NEG	NEG	POS	POS	NEG
ST59/ST952-MRSA-V(T) [PVL+], Taiwan Clone	NEG	COMM	NEG	NEG	NEG	NEG	POS	POS	AMB	POS	POS	POS	NEG	NEG	NEG	NEG	POS	POS	NEG
ST59-MRSA-V	NEG	POS	NEG	NEG	NEG	NEG	POS	POS	NEG	POS	NEG	NEG	NEG	NEG	NEG	NEG	POS	POS	NEG
CC59-MRSA-V [PVL+]	NEG	POS	NEG	NEG	NEG	NEG	POS	POS	NEG	AMB	POS	POS	NEG	NEG	NEG	NEG	AMB	AMB	NEG
ST59-MRSA-IV/V, WA-15	NEG	COMM	NEG	NEG	NEG	NEG	POS	POS	AMB	POS	NEG	NEG	NEG	NEG	NEG	NEG	POS	POS	NEG
ST72-MRSA-IV, USA700	POS	NEG	NEG	POS	NEG	NEG	POS	POS	AMB	POS	NEG	NEG	NEG	NEG	POS	POS	POS	POS	NEG
ST72-MRSA-IV [PVL+], WA-44	POS	NEG	NEG	POS	NEG	NEG	POS	POS	AMB	POS	POS	POS	NEG	NEG	POS	POS	POS	POS	NEG
CC75-MRSA-IV	POS	NEG	RARE	POS	NEG	NEG	NEG	NEG	NEG	NEG	NEG	NEG	NEG	NEG	NEG	NEG	NEG	NEG	NEG
ST883-MRSA-IV, WA-47	AMB	NEG	NEG	NEG	NEG	NEG	NEG	NEG	NEG	NEG	NEG	NEG	NEG	NEG	NEG	NEG	NEG	NEG	NEG
ST1303-MRSA-IV	POS	NEG	NEG	POS	NEG	NEG	NEG	NEG	NEG	NEG	NEG	NEG	NEG	NEG	NEG	NEG	NEG	NEG	NEG
CC80-MRSA-IV	NEG	NEG	NEG	NEG	NEG	NEG	POS	POS	NEG	POS	NEG	NEG	NEG	NEG	POS	POS	POS	POS	NEG
CC80-MRSA-IV [PVL+], European Clone	NEG	NEG	NEG	NEG	NEG	NEG	POS	POS	AMB	POS	POS	POS	NEG	NEG	POS	POS	POS	POS	NEG
CC88-MRSA-IV, WA-2	NEG	NEG	NEG	NEG	NEG	NEG	POS	POS	AMB	POS	NEG	NEG	NEG	NEG	POS	POS	POS	POS	NEG
CC88-MRSA-IV [PVL+]	NEG	VAR	NEG	NEG	NEG	NEG	POS	POS	AMB	POS	POS	POS	NEG	NEG	POS	POS	POS	POS	NEG
CC88-MRSA-V [PVL+]	NEG	POS	NEG	NEG	NEG	NEG	POS	POS	AMB	POS	POS	POS	NEG	NEG	POS	POS	POS	POS	NEG
CC88-MRSA-VI	NEG	NEG	NEG	NEG	NEG	NEG	POS	POS	AMB	POS	NEG	NEG	NEG	NEG	POS	POS	POS	POS	NEG
ST93-MRSA-IV [PVL-]	NEG	NEG	NEG	NEG	NEG	POS	NEG	NEG	NEG	NEG	NEG	NEG	NEG	NEG	NEG	NEG	POS	AMB	NEG
ST93-MRSA-IV [PVL+], Queensland Clone	NEG	NEG	NEG	NEG	AMB	POS	NEG	NEG	NEG	NEG	POS	POS	NEG	NEG	AMB	NEG	POS	POS	NEG
ST93-MRSA-V [PVL+]	NEG	NEG	NEG	NEG	AMB	POS	NEG	NEG	NEG	NEG	NEG	NEG	NEG	NEG	AMB	NEG	POS	POS	NEG
CC97-MRSA-IV, WA-54	NEG	NEG	NEG	NEG	NEG	NEG	POS	POS	NEG	POS	NEG	NEG	NEG	NEG	POS	POS	POS	POS	NEG
CC97-MRSA-V	NEG	NEG	NEG	NEG	NEG	NEG	POS	POS	AMB	POS	NEG	NEG	NEG	NEG	POS	POS	POS	POS	NEG
CC97-MRSA-V [ACME+]	NEG	NEG	POS	NEG	NEG	NEG	POS	POS	AMB	POS	NEG	NEG	NEG	NEG	POS	POS	POS	POS	NEG
CC97-MRSA-I/V	NEG	NEG	NEG	NEG	NEG	NEG	POS	POS	AMB	POS	NEG	NEG	NEG	NEG	POS	POS	POS	POS	NEG
CC121-MRSA-V, WA-22	POS	NEG	NEG	POS	POS	POS	POS	POS	POS	POS	NEG	NEG	NEG	NEG	NEG	POS	POS	POS	NEG
CC152-MRSA-V [PVL+]	NEG	NEG	NEG	NEG	NEG	NEG	AMB	NEG	NEG	AMB	POS	POS	NEG	NEG	AMB	NEG	NEG	POS	NEG
ST154-MRSA [PVL+]	NEG	NEG	NEG	NEG	NEG	NEG	POS	POS	AMB	AMB	POS	POS	NEG	NEG	POS	AMB	AMB	POS	NEG
ST188-MRSA-IV, WA-38/78	NEG	NEG	NEG	NEG	NEG	NEG	POS	POS	AMB	POS	NEG	NEG	NEG	NEG	POS	POS	AMB	POS	NEG
ST188-MRSA-V	NEG	NEG	NEG	NEG	NEG	NEG	POS	POS	POS	POS	NEG	NEG	NEG	NEG	POS	POS	POS	POS	NEG
ST239-MRSA-III	NEG	COMM	NEG	NEG	NEG	NEG	POS	POS	AMB	POS	NEG	NEG	NEG	NEG	COMM	COMM	POS	POS	NEG
CC361-MRSA-IV, WA-29	COMM	NEG	NEG	AMB	NEG	NEG	POS	POS	AMB	POS	NEG	NEG	NEG	NEG	POS	POS	POS	POS	NEG
CC361-MRSA-V	POS	NEG	NEG	POS	NEG	NEG	POS	POS	AMB	POS	NEG	NEG	NEG	NEG	POS	POS	POS	POS	NEG
CC361-MRSA-VIII, WA-28	POS	NEG	NEG	POS	NEG	NEG	POS	POS	NEG	POS	NEG	NEG	NEG	NEG	POS	POS	POS	POS	NEG
CC398-MRSA-IV	NEG	NEG	NEG	NEG	NEG	NEG	POS	POS	AMB	POS	NEG	NEG	NEG	NEG	NEG	NEG	POS	POS	NEG
ST398-MRSA-V	NEG	RARE	NEG	NEG	NEG	NEG	POS	POS	AMB	POS	NEG	NEG	NEG	NEG	NEG	NEG	POS	POS	NEG
ST398-MRSA-V [PVL+]	NEG	NEG	NEG	NEG	NEG	NEG	POS	POS	NEG	POS	POS	POS	NEG	NEG	NEG	NEG	AMB	POS	NEG
CC509/ST207-MRSA-V	POS	NEG	NEG	NEG	POS	POS	POS	NEG	POS	POS	NEG	NEG	NEG	NEG	NEG	NEG	NEG	NEG	POS
ST573-MRSA-V, WA-10	POS	NEG	NEG	POS	POS	POS	POS	POS	AMB	POS	NEG	NEG	NEG	NEG	NEG	NEG	POS	POS	NEG
ST772-MRSA-V [PVL+], WA-60/Bengal Bay Clone	POS	NEG	NEG	POS	POS	POS	POS	POS	AMB	POS	POS	POS	NEG	NEG	NEG	NEG	POS	POS	NEG
ST779-MRSA	NEG	NEG	RARE	NEG	NEG	NEG	COMM	COMM	RARE	COMM	NEG	NEG	NEG	NEG	POS	AMB	POS	POS	NEG
ST834-MRSA-IV, WA-13	NEG	NEG	NEG	NEG	NEG	NEG	POS	POS	AMB	POS	NEG	NEG	NEG	NEG	POS	POS	AMB	POS	NEG
CC913-MRSA-IV	NEG	NEG	NEG	NEG	NEG	NEG	POS	POS	NEG	POS	NEG	NEG	NEG	NEG	POS	POS	POS	POS	NEG
ST1774-MRSA-IV [ACME+]	NEG	NEG	NEG	NEG	NEG	NEG	POS	POS	POS	POS	NEG	NEG	NEG	NEG	POS	POS	POS	POS	NEG
CC15-MSSA	NEG	NEG	NEG	NEG	NEG	NEG	POS	POS	AMB	POS	RARE	RARE	NEG	NEG	POS	AMB	POS	POS	NEG
ST426-MSSA	NEG	RARE	NEG	NEG	POS	AMB	POS	NEG	POS	POS	NEG	NEG	NEG	NEG	NEG	POS	AMB	AMB	NEG

Legend:

Positive :	POS
Ambiguous :	AMB
Negative :	NEG
Rare (in >0 to 33% of isolates) :	RARE
Variable (in >33 to 66% of isolates) :	VAR
Common (in >66 to <100% of isolates) :	COMM

	VIRULENCE : HAEMOLYSINS								VIRULENCE : HLB-CONVERTING PHAGES			VIRULENCE : EXFOLIATIVE TOXINS			VIRULENCE : EPITHEL. DIFF. INHIBITORS		
	hl	hla	hlIII (cons)	hlIII (other than RF122)	hlb-probe 1	hlb-probe 2	hlb-probe 3	un-truncated hlb	sak	chp	scn	etA	etB	etD	edinA	edinB	edinC
CC1-MRSA-IV [PVL-], WA-1/57	POS	POS	POS	POS	POS	POS	POS	RARE	COMM	RARE	COMM	NEG	NEG	NEG	NEG	NEG	NEG
CC1-MRSA-IV [PVL+], USA400	POS	POS	POS	POS	POS	POS	POS	NEG	POS	NEG	POS	NEG	NEG	NEG	NEG	NEG	NEG
CC1-MRSA-IV/SCCfus [PVL-]	POS	POS	POS	POS	POS	POS	POS	NEG	COMM	NEG	COMM	NEG	NEG	NEG	NEG	NEG	NEG
CC1-MRSA-IV/SCCfus [PVL+]	POS	POS	POS	POS	AMB	POS	AMB	NEG	POS	NEG	POS	NEG	NEG	NEG	NEG	NEG	NEG
CC1-MRSA-V [PVL+]	POS	POS	POS	POS	POS	POS	POS	NEG	POS	NEG	POS	NEG	NEG	NEG	NEG	NEG	NEG
CC1-MRSA-V/SCCfus [PVL-]	POS	POS	POS	POS	POS	POS	POS	NEG	POS	NEG	POS	NEG	NEG	NEG	NEG	NEG	NEG
CC1-MRSA-V/SCCfus [PVL+]	POS	POS	POS	POS	POS	POS	POS	NEG	POS	NEG	POS	NEG	NEG	NEG	NEG	NEG	NEG
ST228-MRSA-I, South German/Italian MRSA	POS	POS	POS	POS	POS	POS	AMB	NEG	POS	RARE	POS	NEG	NEG	NEG	NEG	NEG	NEG
ST5-MRSA-I, Geraldine Clone	POS	POS	POS	POS	POS	POS	POS	NEG	POS	POS	POS	NEG	NEG	NEG	NEG	NEG	NEG
CC5-MRSA-Ivar., WA-18/21/48	POS	POS	POS	POS	POS	POS	AMB	NEG	POS	POS	POS	NEG	NEG	NEG	NEG	NEG	NEG
CC5-MRSA-II, Rhine-Hesse /UK-3	POS	POS	POS	POS	POS	POS	AMB	NEG	POS	COMM	POS	NEG	NEG	NEG	NEG	NEG	NEG
CC5-MRSA-II [ACME+]	POS	POS	POS	POS	POS	POS	POS	NEG	POS	POS	POS	NEG	NEG	NEG	NEG	NEG	NEG
CC5-MRSA-III	POS	POS	POS	POS	POS	POS	AMB	NEG	VAR	NEG	NEG	NEG	NEG	NEG	NEG	NEG	NEG
CC5-MRSA-IV, Paediatric clone	POS	POS	POS	POS	POS	POS	AMB	RARE	COMM	COMM	COMM	NEG	NEG	NEG	RARE	NEG	NEG
CC5-MRSA-IV [PVL+]	POS	POS	POS	POS	POS	POS	AMB	NEG	POS	POS	POS	NEG	NEG	NEG	VAR	NEG	NEG
CC5-MRSA-V	POS	POS	POS	POS	POS	POS	AMB	NEG	POS	VAR	POS	NEG	NEG	NEG	NEG	NEG	NEG
CC5-MRSA-V [PVL+]	POS	POS	POS	POS	POS	POS	AMB	NEG	POS	NEG	POS	NEG	NEG	NEG	NEG	NEG	NEG
CC5-MRSA-VI, New Paediatric Clone	POS	POS	POS	POS	AMB	POS	AMB	VAR	COMM	COMM	COMM	NEG	NEG	NEG	NEG	NEG	NEG
ST5-MRSA-VII (SCC-JCSC6082)	POS	POS	POS	POS	NEG	POS	NEG	NEG	POS	POS	POS	NEG	NEG	NEG	NEG	NEG	NEG
CC5-MRSA-IVvar, Maltese Clone	POS	POS	POS	POS	POS	POS	POS	NEG	POS	NEG	POS	NEG	NEG	NEG	NEG	NEG	NEG
CC5-MRSA-SCC(MRSAZH47)	POS	POS	POS	POS	POS	POS	AMB	NEG	POS	NEG	POS	NEG	NEG	NEG	NEG	NEG	NEG
CC5-MRSA-IV/VI	POS	POS	POS	POS	AMB	POS	AMB	VAR	VAR	VAR	VAR	NEG	NEG	NEG	RARE	NEG	NEG
CC5-MRSA-IV/SCCfus	POS	POS	POS	POS	AMB	POS	NEG	NEG	POS	POS	POS	NEG	NEG	NEG	NEG	NEG	NEG
CC5/ST835-MRSA, WA-40/46	POS	POS	POS	POS	POS	POS	POS	NEG	POS	NEG	POS	NEG	NEG	NEG	NEG	NEG	NEG
ST6-MRSA-IV, WA-51	POS	POS	POS	POS	AMB	POS	AMB	NEG	POS	NEG	POS	NEG	NEG	NEG	NEG	NEG	NEG
ST6-MRSA-V	POS	POS	POS	POS	POS	POS	POS	POS	NEG	AMB	NEG	NEG	NEG	NEG	NEG	NEG	NEG
CC7-MRSA-IV	POS	POS	POS	POS	POS	POS	AMB	NEG	POS	NEG	POS	NEG	NEG	NEG	NEG	NEG	NEG
CC7-MRSA-V	POS	POS	POS	POS	POS	POS	AMB	NEG	POS	NEG	POS	NEG	NEG	NEG	NEG	NEG	NEG
ST250-MRSA-I, Early/Ancestral MRSA	POS	POS	POS	POS	POS	POS	POS	RARE	COMM	COMM	COMM	NEG	NEG	NEG	NEG	NEG	NEG
ST247-MRSA-I, North German/Iberian MRSA	POS	POS	POS	POS	POS	POS	POS	RARE	POS	COMM	POS	NEG	NEG	NEG	NEG	NEG	NEG
CC8-MRSA-IV, UK-14/WA-5	POS	POS	POS	POS	POS	POS	POS	VAR	COMM	COMM	COMM	NEG	NEG	NEG	NEG	NEG	NEG
CC8-MRSA-IV, Lyon Clone/UK-2	POS	POS	POS	POS	POS	POS	POS	NEG	POS	RARE	POS	NEG	NEG	NEG	NEG	NEG	NEG
CC8-MRSA-IV, UK-6	POS	POS	POS	POS	POS	POS	POS	NEG	POS	RARE	POS	NEG	NEG	NEG	NEG	NEG	NEG
CC8-MRSA-IV, USA500	POS	POS	POS	POS	POS	POS	POS	RARE	COMM	RARE	COMM	NEG	NEG	NEG	NEG	NEG	NEG
ST8-MRSA-IV [PVL/ACME+], USA300	POS	POS	POS	POS	POS	POS	POS	RARE	COMM	COMM	COMM	NEG	NEG	NEG	NEG	NEG	NEG
ST8-MRSA-IV [PVL+], USA300	POS	POS	POS	POS	POS	POS	POS	RARE	COMM	COMM	COMM	NEG	NEG	NEG	NEG	NEG	NEG
CC8-MRSA-IV [PVL/sed/j/r/k/q+]	POS	POS	POS	POS	POS	POS	POS	NEG	POS	POS	POS	NEG	NEG	NEG	NEG	NEG	NEG
CC8-MRSA-IV [tst1/sec/sel+]	POS	POS	POS	POS	POS	POS	POS	COMM	COMM	COMM	COMM	NEG	NEG	NEG	VAR	NEG	NEG
CC8-MRSA-V	POS	POS	POS	POS	POS	POS	POS	AMB	POS	RARE	POS	NEG	NEG	NEG	NEG	NEG	NEG
CC8-MRSA-VIII	POS	POS	POS	POS	POS	POS	AMB	NEG	POS	NEG	POS	NEG	NEG	NEG	NEG	NEG	NEG
ST8-MRSA-IIA/B/C/D/E, Irish AR13/14	POS	POS	POS	POS	POS	POS	POS	RARE	COMM	RARE	COMM	NEG	NEG	NEG	NEG	NEG	NEG
ST8-MRSA, UK-12/13, Irish AR43	POS	POS	POS	POS	POS	POS	POS	RARE	COMM	RARE	COMM	NEG	NEG	NEG	NEG	NEG	NEG
ST254-MRSA, Hannover EMRSA	POS	POS	POS	POS	POS	POS	POS	COMM	RARE	RARE	VAR	NEG	NEG	NEG	NEG	NEG	NEG
ST254-MRSA-IV/V, Hannover/UK-10	POS	POS	POS	POS	POS	POS	POS	VAR	VAR	RARE	VAR	NEG	NEG	NEG	NEG	NEG	NEG
CC9-MRSA-IV	POS	POS	POS	POS	POS	POS	POS	POS	NEG	NEG	NEG	NEG	NEG	NEG	NEG	NEG	NEG
CC12-MRSA, WA-59	POS	POS	POS	POS	POS	POS	AMB	NEG	POS	NEG	POS	NEG	NEG	NEG	NEG	NEG	NEG
CC12-MRSA-IV	POS	POS	POS	POS	POS	POS	POS	NEG	POS	NEG	POS	NEG	NEG	NEG	NEG	NEG	NEG
CC20-MRSA-V	POS	POS	POS	POS	AMB	POS	AMB	NEG	POS	POS	POS	NEG	NEG	NEG	NEG	NEG	NEG
ST22-MRSA-IV, Barnim/UK-15	POS	POS	NEG	NEG	POS	POS	POS	RARE	COMM	COMM	COMM	NEG	NEG	NEG	NEG	NEG	NEG
ST22-MRSA-IV [ACME+]	POS	POS	NEG	NEG	POS	POS	POS	NEG	POS	POS	POS	NEG	NEG	NEG	NEG	NEG	NEG
ST22-MRSA-IV [PVL+]	POS	POS	NEG	NEG	POS	POS	POS	NEG	POS	POS	POS	NEG	NEG	NEG	NEG	NEG	NEG
ST22-MRSA-V	POS	AMB	NEG	NEG	POS	POS	POS	NEG	POS	POS	POS	NEG	NEG	NEG	NEG	NEG	NEG
ST22-MRSA-V [PVL+]	POS	POS	NEG	NEG	POS	POS	POS	NEG	POS	POS	POS	NEG	NEG	NEG	NEG	NEG	NEG
ST36-MRSA-II, UK-16	POS	POS	POS	POS	AMB	POS	AMB	NEG	POS	COMM	POS	NEG	NEG	NEG	NEG	NEG	NEG
ST30-MRSA-IV [PVL+], Southwest Pacific Clone	POS	POS	POS	POS	POS	POS	AMB	NEG	POS	POS	POS	NEG	NEG	NEG	NEG	NEG	NEG
ST30-MRSA-IV [tst1+]	POS	POS	POS	POS	POS	POS	AMB	RARE	COMM	COMM	COMM	NEG	NEG	NEG	NEG	NEG	NEG
CC30-MRSA-V [PVL+]	POS	POS	POS	POS	POS	POS	POS	NEG	POS	POS	POS	NEG	NEG	NEG	NEG	NEG	NEG
ST45-MRSA-II, USA600	POS	POS	POS	NEG	AMB	AMB	NEG	NEG	POS	POS	POS	NEG	NEG	NEG	NEG	NEG	NEG
ST45-MRSA-IV, Berlin EMRSA	POS	POS	POS	NEG	AMB	AMB	NEG	RARE	COMM	COMM	COMM	NEG	NEG	NEG	NEG	NEG	NEG
CC45-MRSA-IV [ACME+]	POS	POS	POS	NEG	NEG	AMB	NEG	NEG	POS	POS	POS	NEG	NEG	NEG	NEG	NEG	NEG
ST45-MRSA-V, WA-4	POS	POS	POS	NEG	AMB	POS	NEG	NEG	POS	POS	POS	NEG	NEG	NEG	NEG	NEG	NEG
CC45/agr IV-MRSA-IV, WA-23	POS	POS	POS	NEG	NEG	NEG	NEG	NEG	POS	POS	POS	NEG	NEG	NEG	NEG	NEG	NEG
CC45/agr IV-MRSA-V	POS	POS	POS	NEG	NEG	NEG	NEG	NEG	COMM	COMM	COMM	NEG	NEG	NEG	NEG	NEG	NEG
CC45/agr IV-MRSA-IV/V	POS	POS	POS	NEG	NEG	NEG	NEG	NEG	POS	POS	POS	NEG	NEG	NEG	NEG	NEG	NEG
CC59-MRSA-IV [PVL+], USA1000	POS	POS	POS	NEG	NEG	NEG	AMB	POS	NEG	POS	POS	NEG	NEG	NEG	NEG	NEG	NEG
ST59-MRSA-IV, WA-73	POS	POS	POS	NEG	NEG	NEG	AMB	POS	VAR	POS	POS	NEG	NEG	NEG	NEG	NEG	NEG
ST87-MRSA-IV, WA-24	POS	POS	POS	NEG	NEG	NEG	AMB	POS	POS	POS	POS	NEG	NEG	NEG	NEG	NEG	NEG
ST59-MRSA-IV [PVL+], WA-55/56	POS	POS	POS	NEG	AMB	NEG	AMB	POS	COMM	POS	POS	NEG	NEG	NEG	NEG	NEG	NEG
ST59/ST952-MRSA-V(T) [PVL+], Taiwan Clone	POS	POS	POS	NEG	AMB	NEG	AMB	POS	NEG	POS	POS	NEG	NEG	NEG	NEG	NEG	NEG
ST59-MRSA-V	POS	POS	POS	NEG	NEG	NEG	AMB	AMB	VAR	POS	POS	NEG	NEG	NEG	NEG	NEG	NEG
CC59-MRSA-V [PVL+]	POS	POS	POS	NEG	NEG	NEG	NEG	POS	NEG	POS	POS	NEG	NEG	NEG	NEG	NEG	NEG
ST59-MRSA-IV/V, WA-15	POS	POS	POS	NEG	AMB	NEG	AMB	POS	POS	POS	POS	NEG	NEG	NEG	NEG	NEG	NEG
ST72-MRSA-IV, USA700	POS	POS	POS	POS	POS	POS	POS	RARE	COMM	COMM	COMM	NEG	NEG	NEG	NEG	NEG	NEG
ST72-MRSA-IV [PVL+], WA-44	POS	POS	POS	POS	POS	POS	POS	AMB	POS	POS	POS	NEG	NEG	NEG	NEG	NEG	NEG
CC75-MRSA-IV	NEG	AMB	AMB	NEG	NEG	NEG	NEG	NEG	POS	NEG	POS	NEG	NEG	NEG	NEG	NEG	NEG
ST883-MRSA-IV, WA-47	NEG	NEG	NEG	NEG	NEG	POS	NEG	NEG	COMM	COMM	COMM	NEG	NEG	NEG	NEG	NEG	NEG
ST1303-MRSA-IV	NEG	POS	NEG	NEG	NEG	NEG	NEG	NEG	NEG	NEG	POS	NEG	NEG	NEG	NEG	NEG	NEG
CC80-MRSA-IV	POS	POS	POS	POS	POS	POS	AMB	NEG	POS	NEG	POS	NEG	NEG	POS	NEG	POS	NEG
CC80-MRSA-IV [PVL+], European Clone	POS	POS	POS	POS	AMB	POS	AMB	NEG	POS	NEG	POS	NEG	NEG	POS	NEG	POS	NEG
CC88-MRSA-IV, WA-2	POS	POS	POS	POS	POS	POS	POS	NEG	POS	RARE	POS	RARE	NEG	NEG	NEG	NEG	NEG
CC88-MRSA-IV [PVL+]	POS	POS	POS	POS	POS	POS	POS	RARE	COMM	COMM	COMM	NEG	NEG	NEG	NEG	NEG	NEG
CC88-MRSA-V [PVL+]	POS	POS	POS	POS	POS	POS	POS	NEG	POS	POS	POS	NEG	NEG	NEG	NEG	NEG	NEG
CC88-MRSA-VI	POS	POS	POS	POS	POS	POS	POS	NEG	POS	NEG	POS	NEG	NEG	NEG	NEG	NEG	NEG
ST93-MRSA-IV [PVL-]	AMB	AMB	POS	POS	POS	POS	POS	NEG	POS	POS	POS	NEG	NEG	NEG	NEG	NEG	NEG
ST93-MRSA-IV [PVL+], Queensland Clone	POS	POS	POS	POS	POS	POS	POS	NEG	POS	POS	POS	NEG	NEG	NEG	NEG	NEG	NEG
ST93-MRSA-V [PVL+]	POS	POS	POS	POS	POS	POS	POS	NEG	POS	POS	POS	NEG	NEG	NEG	NEG	NEG	NEG
CC97-MRSA-IV, WA-54	POS	POS	POS	POS	POS	POS	POS	NEG	POS	NEG	POS	NEG	NEG	NEG	NEG	NEG	NEG
CC97-MRSA-V	POS	POS	POS	POS	POS	POS	POS	NEG	POS	NEG	POS	NEG	NEG	NEG	NEG	NEG	NEG
CC97-MRSA-V [ACME+]	POS	POS	POS	POS	POS	POS	POS	AMB	POS	POS	POS	NEG	NEG	NEG	NEG	NEG	NEG
CC97-MRSA-I/V	POS	POS	POS	POS	POS	POS	POS	POS	VAR	NEG	NEG	NEG	NEG	NEG	NEG	NEG	NEG
CC121-MRSA-V, WA-22	POS	POS	POS	NEG	POS	POS	POS	NEG	POS	NEG	POS	POS	NEG	NEG	POS	NEG	NEG
CC152-MRSA-V [PVL+]	NEG	POS	POS	NEG	NEG	NEG	POS	POS	COMM	NEG	COMM	NEG	NEG	NEG	NEG	POS	NEG
ST154-MRSA [PVL+]	POS	POS	POS	POS	AMB	POS	AMB	VAR	VAR	VAR	VAR	NEG	NEG	NEG	NEG	NEG	NEG
ST188-MRSA-IV, WA-38/78	POS	POS	POS	POS	POS	POS	POS	NEG	POS	NEG	POS	NEG	NEG	NEG	NEG	NEG	NEG
ST188-MRSA-V	POS	POS	POS	POS	POS	POS	POS	AMB	POS	POS	POS	NEG	NEG	NEG	NEG	NEG	NEG
ST239-MRSA-III	POS	POS	POS	POS	POS	POS	POS	RARE	COMM	RARE	COMM	NEG	NEG	NEG	NEG	NEG	NEG
CC361-MRSA-IV, WA-29	POS	POS	POS	POS	POS	POS	AMB	NEG	POS	COMM	POS	NEG	NEG	NEG	NEG	NEG	NEG
CC361-MRSA-V	POS	POS	POS	POS	POS	POS	POS	NEG	POS	NEG	POS	NEG	NEG	NEG	NEG	NEG	NEG
CC361-MRSA-VIII, WA-28	POS	POS	POS	POS	NEG	POS	NEG	NEG	POS	POS	POS	NEG	NEG	NEG	NEG	NEG	NEG
CC398-MRSA-IV	POS	POS	POS	POS	NEG	NEG	NEG	POS	RARE	RARE	RARE	NEG	NEG	NEG	NEG	NEG	NEG
ST398-MRSA-V	POS	POS	POS	POS	NEG	NEG	NEG	POS	RARE	RARE	RARE	NEG	NEG	NEG	NEG	NEG	NEG
ST398-MRSA-V [PVL+]	POS	POS	POS	POS	NEG	NEG	NEG	NEG	POS	POS	POS	NEG	NEG	NEG	NEG	NEG	NEG
CC509/ST207-MRSA-V	POS	POS	NEG	POS	POS	POS	POS	NEG	POS	POS	POS	NEG	NEG	NEG	NEG	NEG	NEG
ST573-MRSA-V, WA-10	POS	POS	POS	POS	POS	POS	POS	NEG	NEG	NEG	POS	NEG	NEG	NEG	NEG	NEG	NEG
ST772-MRSA-V [PVL+], WA-60/Bengal Bay Clone	POS	POS	POS	POS	NEG	NEG	AMB	NEG	NEG	NEG	POS	NEG	NEG	NEG	NEG	NEG	NEG
ST779-MRSA	POS	POS	POS	POS	POS	POS	POS	NEG	POS	POS	POS	NEG	NEG	POS	NEG	POS	NEG
ST834-MRSA-IV, WA-13	POS	POS	POS	POS	POS	POS	POS	NEG	POS	POS	POS	NEG	NEG	NEG	NEG	NEG	NEG
CC913-MRSA-IV	POS	POS	POS	POS	POS	POS	POS	NEG	POS	POS	POS	POS	NEG	POS	NEG	POS	NEG
ST1774-MRSA-IV [ACME+]	POS	POS	POS	POS	POS	POS	POS	NEG	POS	POS	POS	NEG	NEG	NEG	NEG	NEG	NEG

CC15-MSSA	POS	POS	POS	POS	NEG	NEG	AMB	NEG	NEG	POS	POS	RARE	NEG	NEG	NEG	NEG	NEG
ST426-MSSA	POS	POS	POS	NEG	NEG	NEG	NEG	NEG	POS	POS	POS	NEG	NEG	NEG	NEG	NEG	NEG

Legend:

Positive :	POS
Ambiguous :	AMB
Negative :	NEG
Rare (in >0 to 33% of isolates) :	RARE
Variable (in >33 to 66% of isolates) :	VAR
Common (in >66 to <100% of isolates) :	COMM

	VIRULENCE : ACME LOCUS				VIRULENCE : PROTEASES									
	arcA-SCC	arcB-SCC	arcC-SCC	arcD-SCC	aur (cons)	aur (Other than MRSA252)	aur (MRSA252)	splA	splB	splE	sspA	sspB	sspP (cons)	sspP (other than ST93)
CC1-MRSA-IV [PVL-], WA-1/57	NEG	NEG	NEG	NEG	POS	POS	NEG	POS	POS	POS	POS	POS	POS	POS
CC1-MRSA-IV [PVL+], USA400	NEG	NEG	NEG	NEG	POS	POS	NEG	POS	POS	AMB	POS	POS	POS	POS
CC1-MRSA-IV/SCCfus [PVL-]	NEG	NEG	NEG	NEG	POS	POS	NEG	POS	POS	POS	POS	POS	POS	POS
CC1-MRSA-IV/SCCfus [PVL+]	NEG	NEG	NEG	NEG	POS	POS	NEG	POS	POS	AMB	POS	POS	POS	POS
CC1-MRSA-V [PVL+]	NEG	NEG	NEG	NEG	POS	POS	NEG	POS	POS	POS	POS	POS	POS	POS
CC1-MRSA-V/SCCfus [PVL-]	NEG	NEG	NEG	NEG	POS	POS	NEG	POS	POS	POS	POS	POS	POS	POS
CC1-MRSA-V/SCCfus [PVL+]	NEG	NEG	NEG	NEG	POS	POS	NEG	POS	POS	POS	POS	POS	POS	POS
ST228-MRSA-I, South German/Italian MRSA	NEG	NEG	NEG	NEG	POS	POS	NEG	POS	POS	NEG	POS	POS	POS	POS
ST5-MRSA-I, Geraldine Clone	NEG	NEG	NEG	NEG	POS	POS	NEG	POS	POS	NEG	POS	POS	POS	POS
CC5-MRSA-Ivar., WA-18/21/48	NEG	NEG	NEG	NEG	POS	POS	NEG	POS	POS	NEG	POS	POS	POS	POS
CC5-MRSA-II, Rhine-Hesse /UK-3	NEG	NEG	NEG	NEG	POS	POS	NEG	POS	POS	NEG	POS	POS	POS	POS
CC5-MRSA-II [ACME+]	POS	POS	POS	POS	POS	POS	NEG	POS	POS	NEG	POS	POS	POS	POS
CC5-MRSA-III	NEG	NEG	NEG	NEG	POS	POS	NEG	POS	POS	NEG	POS	POS	POS	POS
CC5-MRSA-IV, Paediatric clone	NEG	NEG	NEG	NEG	POS	POS	NEG	POS	POS	NEG	POS	POS	POS	POS
CC5-MRSA-IV [PVL+]	NEG	NEG	NEG	NEG	POS	POS	NEG	POS	POS	NEG	POS	POS	POS	POS
CC5-MRSA-V	NEG	NEG	NEG	NEG	POS	POS	NEG	POS	POS	NEG	POS	POS	POS	POS
CC5-MRSA-V [PVL+]	NEG	NEG	NEG	NEG	POS	POS	NEG	POS	POS	NEG	POS	POS	POS	POS
CC5-MRSA-VI, New Paediatric Clone	NEG	NEG	NEG	NEG	POS	POS	NEG	POS	POS	NEG	POS	POS	POS	POS
ST5-MRSA-VII (SCC-JCSC6082)	NEG	NEG	NEG	NEG	POS	POS	NEG	POS	POS	NEG	POS	POS	POS	POS
CC5-MRSA-IVvar, Maltese Clone	NEG	NEG	NEG	NEG	POS	POS	NEG	POS	POS	NEG	POS	POS	POS	POS
CC5-MRSA-SCC(MRSAZH47)	NEG	NEG	NEG	NEG	POS	POS	NEG	POS	POS	NEG	POS	POS	POS	POS
CC5-MRSA-IV/VI	NEG	NEG	NEG	NEG	POS	POS	NEG	POS	POS	NEG	POS	POS	POS	POS
CC5-MRSA-IV/SCCfus	NEG	NEG	NEG	NEG	POS	POS	NEG	POS	POS	NEG	POS	POS	POS	POS
CC5/ST835-MRSA, WA-40/46	NEG	NEG	NEG	NEG	POS	POS	NEG	POS	POS	NEG	POS	POS	POS	POS
ST6-MRSA-IV, WA-51	NEG	NEG	NEG	NEG	POS	AMB	NEG	POS	POS	POS	POS	POS	POS	POS
ST6-MRSA-V	NEG	NEG	NEG	NEG	POS	POS	NEG	POS	POS	POS	POS	POS	POS	POS
CC7-MRSA-IV	NEG	NEG	NEG	NEG	POS	POS	NEG	POS	POS	POS	POS	POS	POS	POS
CC7-MRSA-V	NEG	NEG	NEG	NEG	POS	POS	NEG	POS	POS	POS	POS	POS	POS	POS
ST250-MRSA-I, Early/Ancestral MRSA	NEG	NEG	NEG	NEG	POS	POS	NEG	POS	POS	POS	POS	POS	POS	POS
ST247-MRSA-I, North German/Iberian MRSA	NEG	NEG	NEG	NEG	POS	POS	NEG	COMM	COMM	COMM	POS	POS	POS	POS
CC8-MRSA-IV, UK-14/WA-5	NEG	NEG	NEG	NEG	POS	POS	NEG	POS	POS	POS	POS	POS	POS	POS
CC8-MRSA-IV, Lyon Clone/UK-2	NEG	NEG	NEG	NEG	POS	POS	NEG	POS	POS	POS	POS	POS	POS	POS
CC8-MRSA-IV, UK-6	NEG	NEG	NEG	NEG	POS	POS	NEG	POS	POS	POS	POS	POS	POS	POS
CC8-MRSA-IV, USA500	NEG	NEG	NEG	NEG	POS	POS	NEG	POS	POS	POS	POS	POS	POS	POS
ST8-MRSA-IV [PVL/ACME+], USA300	POS	POS	POS	POS	POS	POS	NEG	POS	POS	POS	POS	POS	POS	POS
ST8-MRSA-IV [PVL+], USA300	NEG	NEG	NEG	NEG	POS	POS	NEG	POS	POS	POS	POS	POS	POS	POS
CC8-MRSA-IV [PVL/sed/j/r/k/q+]	NEG	NEG	NEG	NEG	POS	POS	NEG	NEG	NEG	NEG	POS	POS	POS	POS
CC8-MRSA-IV [tst1/sec/sel+]	NEG	NEG	NEG	NEG	POS	POS	NEG	POS	POS	NEG	POS	POS	POS	POS
CC8-MRSA-V	NEG	NEG	NEG	NEG	POS	POS	NEG	POS	POS	POS	POS	POS	POS	POS
CC8-MRSA-VIII	NEG	NEG	NEG	NEG	POS	POS	NEG	VAR	VAR	RARE	POS	POS	POS	POS
ST8-MRSA-IIA/B/C/D/E, Irish AR13/14	NEG	NEG	NEG	NEG	POS	POS	NEG	COMM	COMM	COMM	POS	POS	POS	POS
ST8-MRSA, UK-12/13, Irish AR43	NEG	NEG	NEG	NEG	POS	POS	NEG	POS	POS	POS	POS	POS	POS	POS
ST254-MRSA, Hannover EMRSA	NEG	NEG	NEG	NEG	POS	POS	NEG	POS	POS	POS	POS	POS	POS	POS
ST254-MRSA-IV/V, Hannover/UK-10	NEG	NEG	NEG	NEG	POS	POS	NEG	POS	POS	POS	POS	POS	POS	POS
CC9-MRSA-IV	NEG	NEG	NEG	NEG	POS	POS	NEG	NEG	NEG	NEG	POS	POS	POS	POS
CC12-MRSA, WA-59	NEG	NEG	NEG	NEG	POS	POS	NEG	POS	POS	POS	POS	POS	POS	POS
CC12-MRSA-IV	NEG	NEG	NEG	NEG	POS	POS	NEG	POS	POS	POS	POS	POS	POS	POS
CC20-MRSA-V	NEG	NEG	NEG	NEG	POS	POS	NEG	POS	POS	NEG	POS	POS	POS	POS
ST22-MRSA-IV, Barnim/UK-15	NEG	NEG	NEG	NEG	POS	NEG	POS	NEG	NEG	NEG	POS	POS	POS	POS
ST22-MRSA-IV [ACME+]	POS	POS	POS	POS	POS	NEG	POS	NEG	NEG	NEG	POS	POS	POS	POS
ST22-MRSA-IV [PVL+]	NEG	NEG	NEG	NEG	POS	NEG	POS	NEG	NEG	NEG	POS	POS	POS	POS
ST22-MRSA-V	NEG	NEG	NEG	NEG	POS	NEG	POS	NEG	NEG	NEG	POS	POS	POS	POS
ST22-MRSA-V [PVL+]	NEG	NEG	NEG	NEG	POS	NEG	POS	NEG	NEG	NEG	POS	POS	POS	POS
ST36-MRSA-II, UK-16	NEG	NEG	NEG	NEG	POS	NEG	POS	NEG	NEG	POS	POS	POS	POS	POS
ST30-MRSA-IV [PVL+], Southwest Pacific Clone	NEG	NEG	NEG	NEG	POS	NEG	POS	NEG	NEG	POS	POS	POS	POS	POS
ST30-MRSA-IV [tst1+]	NEG	NEG	NEG	NEG	POS	NEG	POS	NEG	NEG	POS	POS	POS	POS	POS
CC30-MRSA-V [PVL+]	NEG	NEG	NEG	NEG	POS	NEG	POS	NEG	NEG	POS	POS	POS	POS	POS
ST45-MRSA-II, USA600	NEG	NEG	NEG	NEG	AMB	NEG	POS	NEG	NEG	NEG	POS	POS	POS	POS
ST45-MRSA-IV, Berlin EMRSA	NEG	NEG	NEG	NEG	AMB	NEG	POS	NEG	NEG	NEG	POS	POS	POS	POS
CC45-MRSA-IV [ACME+]	POS	POS	POS	POS	AMB	NEG	POS	NEG	NEG	NEG	POS	POS	POS	POS
ST45-MRSA-V, WA-4	NEG	NEG	NEG	NEG	POS	NEG	POS	NEG	NEG	NEG	POS	POS	POS	POS
CC45/agr IV-MRSA-IV, WA-23	NEG	NEG	NEG	NEG	POS	NEG	POS	NEG	NEG	NEG	POS	POS	POS	POS
CC45/agr IV-MRSA-V	NEG	NEG	NEG	NEG	POS	NEG	POS	NEG	NEG	NEG	POS	POS	POS	POS
CC45/agr IV-MRSA-IV/V	VAR	VAR	VAR	VAR	POS	NEG	POS	NEG	NEG	NEG	POS	POS	POS	POS
CC59-MRSA-IV [PVL+], USA1000	NEG	NEG	NEG	NEG	POS	AMB	NEG	NEG	NEG	NEG	POS	POS	POS	POS
ST59-MRSA-IV, WA-73	NEG	NEG	NEG	NEG	POS	NEG	NEG	NEG	NEG	NEG	POS	POS	POS	POS
ST87-MRSA-IV, WA-24	NEG	NEG	NEG	NEG	POS	AMB	NEG	NEG	NEG	NEG	POS	POS	POS	POS
ST59-MRSA-IV [PVL+], WA-55/56	NEG	NEG	NEG	NEG	POS	AMB	NEG	NEG	NEG	NEG	POS	POS	POS	POS
ST59/ST952-MRSA-V(T) [PVL+], Taiwan Clone	NEG	NEG	NEG	NEG	POS	AMB	NEG	NEG	NEG	NEG	POS	POS	POS	POS
ST59-MRSA-V	NEG	NEG	NEG	NEG	POS	AMB	NEG	NEG	NEG	NEG	POS	POS	POS	POS
CC59-MRSA-V [PVL+]	NEG	NEG	NEG	NEG	POS	NEG	NEG	NEG	NEG	NEG	POS	POS	POS	POS
ST59-MRSA-IV/V, WA-15	NEG	NEG	NEG	NEG	POS	AMB	NEG	NEG	NEG	NEG	POS	POS	POS	POS
ST72-MRSA-IV, USA700	NEG	NEG	NEG	NEG	POS	POS	NEG	POS	POS	VAR	POS	POS	POS	POS
ST72-MRSA-IV [PVL+], WA-44	NEG	NEG	NEG	NEG	AMB	POS	NEG	POS	POS	POS	POS	POS	POS	POS
CC75-MRSA-IV	NEG	NEG	NEG	NEG	AMB	AMB	NEG	NEG	NEG	NEG	NEG	AMB	NEG	NEG
ST883-MRSA-IV, WA-47	NEG	NEG	NEG	NEG	POS	POS	NEG	NEG	NEG	NEG	NEG	NEG	NEG	NEG
ST1303-MRSA-IV	NEG	NEG	NEG	NEG	NEG	NEG	NEG	NEG	NEG	NEG	NEG	NEG	NEG	NEG
CC80-MRSA-IV	NEG	NEG	NEG	NEG	POS	POS	NEG	POS	POS	NEG	POS	POS	POS	POS
CC80-MRSA-IV [PVL+], European Clone	NEG	NEG	NEG	NEG	POS	POS	NEG	POS	POS	NEG	POS	POS	POS	POS
CC88-MRSA-IV, WA-2	NEG	NEG	NEG	NEG	POS	POS	NEG	POS	POS	NEG	POS	POS	POS	POS
CC88-MRSA-IV [PVL+]	NEG	NEG	NEG	NEG	POS	POS	NEG	POS	POS	NEG	POS	POS	POS	POS
CC88-MRSA-V [PVL+]	NEG	NEG	NEG	NEG	POS	POS	NEG	POS	POS	NEG	POS	POS	POS	POS
CC88-MRSA-VI	NEG	NEG	NEG	NEG	POS	POS	NEG	POS	POS	NEG	POS	POS	POS	POS
ST93-MRSA-IV [PVL-]	NEG	NEG	NEG	NEG	POS	POS	NEG	POS	NEG	POS	POS	POS	POS	NEG
ST93-MRSA-IV [PVL+], Queensland Clone	NEG	NEG	NEG	NEG	POS	POS	NEG	POS	NEG	AMB	POS	POS	POS	NEG
ST93-MRSA-V [PVL+]	NEG	NEG	NEG	NEG	POS	POS	NEG	POS	NEG	POS	POS	POS	POS	NEG
CC97-MRSA-IV, WA-54	NEG	NEG	NEG	NEG	POS	POS	NEG	POS	POS	POS	POS	POS	POS	POS
CC97-MRSA-V	NEG	NEG	NEG	NEG	POS	POS	NEG	POS	POS	POS	POS	POS	POS	POS
CC97-MRSA-V [ACME+]	POS	POS	POS	POS	POS	POS	NEG	POS	POS	POS	POS	POS	POS	POS
CC97-MRSA-I/V	NEG	NEG	NEG	NEG	POS	POS	NEG	POS	POS	POS	POS	POS	POS	POS
CC121-MRSA-V, WA-22	NEG	NEG	NEG	NEG	POS	POS	NEG	POS	POS	NEG	POS	POS	POS	POS
CC152-MRSA-V [PVL+]	NEG	NEG	NEG	NEG	POS	POS	NEG	NEG	NEG	NEG	POS	POS	POS	POS
ST154-MRSA [PVL+]	NEG	NEG	NEG	NEG	POS	POS	NEG	POS	POS	POS	POS	POS	POS	POS
ST188-MRSA-IV, WA-38/78	NEG	NEG	NEG	NEG	POS	POS	NEG	AMB	POS	AMB	POS	POS	POS	POS
ST188-MRSA-V	NEG	NEG	NEG	NEG	POS	POS	NEG	POS	POS	POS	POS	POS	POS	POS
ST239-MRSA-III	RARE	RARE	RARE	RARE	POS	NEG	POS	COMM	COMM	COMM	POS	POS	POS	POS
CC361-MRSA-IV, WA-29	NEG	NEG	NEG	NEG	POS	POS	AMB	NEG	NEG	NEG	POS	POS	POS	POS
CC361-MRSA-V	NEG	NEG	NEG	NEG	POS	POS	NEG	NEG	NEG	NEG	POS	POS	POS	POS
CC361-MRSA-VIII, WA-28	NEG	NEG	NEG	NEG	NEG	AMB	NEG	NEG	NEG	NEG	POS	POS	POS	POS
CC398-MRSA-IV	NEG	NEG	NEG	NEG	POS	NEG	POS	NEG	NEG	NEG	POS	POS	POS	POS
ST398-MRSA-V	NEG	NEG	NEG	NEG	POS	NEG	POS	NEG	NEG	NEG	POS	POS	POS	POS
ST398-MRSA-V [PVL+]	NEG	NEG	NEG	NEG	POS	NEG	POS	NEG	NEG	NEG	POS	AMB	POS	POS
CC509/ST207-MRSA-V	NEG	NEG	NEG	NEG	POS	NEG	POS	NEG	NEG	NEG	POS	POS	POS	POS
ST573-MRSA-V, WA-10	NEG	NEG	NEG	NEG	POS	POS	NEG	NEG	NEG	NEG	POS	POS	POS	POS
ST772-MRSA-V [PVL+], WA-60/Bengal Bay Clone	NEG	NEG	NEG	NEG	POS	POS	NEG	NEG	NEG	NEG	POS	POS	POS	POS
ST779-MRSA	NEG	NEG	NEG	NEG	POS	POS	NEG	AMB	POS	POS	POS	POS	POS	POS
ST834-MRSA-IV, WA-13	NEG	NEG	NEG	NEG	POS	POS	NEG	POS	POS	POS	POS	POS	POS	POS
CC913-MRSA-IV	NEG	NEG	NEG	NEG	POS	POS	NEG	POS	POS	POS	POS	POS	POS	POS
ST1774-MRSA-IV [ACME+]	POS	POS	POS	POS	POS	NEG	POS	POS	POS	POS	POS	POS	POS	POS
CC15-MSSA	NEG	NEG	NEG	NEG	POS	POS	NEG	POS	POS	POS	POS	POS	POS	POS
ST426-MSSA	NEG	NEG	NEG	NEG	POS	NEG	POS	POS	AMB	POS	POS	POS	POS	POS

Legend:

Positive :	POS
Ambiguous :	AMB
Negative :	NEG
Rare (in >0 to 33% of isolates) :	RARE
Variable (in >33 to 66% of isolates) :	VAR
Common (in >66 to <100% of isolates) :	COMM

	VIRULENCE : STAPHYLOCOCCAL SUPERANTIGEN/ENTEROTOXIN-LIKE GENES (SET/SSL)											
	setC	ssl1/set6 probe 1_11	ssl1/set6 probe 2_11	ssl1/set6 probe 1_12	ssl1/set6 probe 2_12	ssl1/set6 probe 4_11	ssl01-RF122	ssl02/set7	ssl02/set7 (MRSA252)	ssl03/set8 probe 1	ssl03/set8 probe 2	ssl03/set8 (MRSA252, SAR0424)
CC1-MRSA-IV [PVL-], WA-1/57	POS	NEG	POS	NEG	POS	NEG	NEG	POS	NEG	POS	POS	NEG
CC1-MRSA-IV [PVL+], USA400	POS	NEG	POS	NEG	POS	NEG	NEG	POS	NEG	POS	POS	NEG
CC1-MRSA-IV/SCCfus [PVL-]	POS	NEG	POS	NEG	POS	NEG	NEG	POS	NEG	POS	POS	NEG
CC1-MRSA-IV/SCCfus [PVL+]	POS	NEG	POS	NEG	POS	NEG	NEG	POS	AMB	POS	POS	NEG
CC1-MRSA-V [PVL+]	POS	NEG	POS	NEG	POS	NEG	NEG	POS	AMB	POS	POS	NEG
CC1-MRSA-V/SCCfus [PVL-]	POS	NEG	POS	NEG	POS	NEG	NEG	POS	AMB	POS	POS	NEG
CC1-MRSA-V/SCCfus [PVL+]	POS	NEG	POS	NEG	POS	NEG	NEG	POS	AMB	POS	POS	NEG
ST228-MRSA-I, South German/Italian MRSA	POS	POS	NEG	POS	NEG	POS	NEG	POS	AMB	POS	POS	NEG
ST5-MRSA-I, Geraldine Clone	POS	POS	NEG	POS	NEG	POS	NEG	POS	AMB	POS	POS	NEG
CC5-MRSA-Ivar., WA-18/21/48	POS	POS	NEG	POS	NEG	POS	NEG	POS	NEG	POS	POS	NEG
CC5-MRSA-II, Rhine-Hesse /UK-3	POS	POS	NEG	POS	NEG	POS	NEG	POS	AMB	POS	POS	NEG
CC5-MRSA-II [ACME+]	POS	POS	NEG	POS	NEG	POS	NEG	POS	AMB	POS	POS	NEG
CC5-MRSA-III	POS	POS	NEG	POS	NEG	POS	NEG	POS	AMB	POS	POS	NEG
CC5-MRSA-IV, Paediatric clone	POS	POS	NEG	POS	NEG	POS	NEG	POS	AMB	POS	POS	NEG
CC5-MRSA-IV [PVL+]	POS	POS	NEG	POS	NEG	POS	NEG	POS	AMB	POS	POS	NEG
CC5-MRSA-V	POS	POS	NEG	POS	NEG	POS	NEG	POS	AMB	POS	POS	NEG
CC5-MRSA-V [PVL+]	POS	POS	AMB	POS	NEG	POS	NEG	POS	AMB	POS	POS	NEG
CC5-MRSA-VI, New Paediatric Clone	POS	POS	NEG	POS	NEG	POS	NEG	POS	NEG	POS	POS	NEG
ST5-MRSA-VII (SCC-JCSC6082)	POS	POS	NEG	POS	NEG	POS	NEG	POS	NEG	POS	POS	NEG
CC5-MRSA-IVvar, Maltese Clone	POS	POS	NEG	POS	NEG	POS	NEG	POS	AMB	POS	POS	NEG
CC5-MRSA-SCC(MRSAZH47)	POS	POS	NEG	POS	NEG	POS	NEG	POS	NEG	POS	POS	NEG
CC5-MRSA-IV/VI	POS	POS	NEG	POS	NEG	POS	NEG	POS	NEG	POS	POS	NEG
CC5-MRSA-IV/SCCfus	POS	POS	NEG	POS	NEG	POS	NEG	POS	NEG	POS	POS	NEG
CC5/ST835-MRSA, WA-40/46	POS	POS	NEG	POS	NEG	POS	NEG	POS	NEG	POS	POS	NEG
ST6-MRSA-IV, WA-51	POS	NEG	AMB	AMB	NEG	POS	POS	POS	NEG	POS	POS	NEG
ST6-MRSA-V	POS	NEG	NEG	POS	NEG	POS	POS	POS	AMB	POS	POS	NEG
CC7-MRSA-IV	POS	AMB	NEG	NEG	POS	NEG	NEG	POS	NEG	AMB	AMB	NEG
CC7-MRSA-V	POS	AMB	NEG	NEG	POS	NEG	NEG	POS	NEG	AMB	AMB	NEG
ST250-MRSA-I, Early/Ancestral MRSA	POS	POS	NEG	AMB	NEG	POS	NEG	POS	NEG	POS	POS	NEG
ST247-MRSA-I, North German/Iberian MRSA	POS	POS	NEG	AMB	NEG	POS	NEG	POS	NEG	POS	POS	NEG
CC8-MRSA-IV, UK-14/WA-5	POS	POS	NEG	POS	NEG	POS	NEG	POS	AMB	POS	POS	NEG
CC8-MRSA-IV, Lyon Clone/UK-2	POS	POS	NEG	AMB	NEG	POS	NEG	POS	NEG	POS	POS	NEG
CC8-MRSA-IV, UK-6	POS	POS	NEG	POS	NEG	POS	NEG	POS	AMB	POS	POS	NEG
CC8-MRSA-IV, USA500	POS	POS	NEG	AMB	NEG	POS	NEG	POS	NEG	POS	POS	NEG
ST8-MRSA-IV [PVL/ACME+], USA300	POS	POS	NEG	AMB	NEG	POS	NEG	POS	NEG	POS	POS	NEG
ST8-MRSA-IV [PVL+], USA300	POS	POS	NEG	AMB	NEG	POS	NEG	POS	NEG	POS	POS	NEG
CC8-MRSA-IV [PVL/sed/j/r/k/q+]	POS	POS	NEG	POS	NEG	POS	NEG	POS	AMB	POS	POS	NEG
CC8-MRSA-IV [tst1/sec/sel+]	POS	POS	NEG	AMB	NEG	POS	NEG	POS	AMB	POS	POS	NEG
CC8-MRSA-V	POS	POS	NEG	AMB	NEG	POS	NEG	POS	AMB	POS	POS	NEG
CC8-MRSA-VIII	POS	POS	NEG	POS	NEG	POS	NEG	POS	NEG	VAR	VAR	NEG
ST8-MRSA-IIA/B/C/D/E, Irish AR13/14	POS	POS	NEG	AMB	NEG	POS	NEG	POS	NEG	POS	POS	NEG
ST8-MRSA, UK-12/13, Irish AR43	POS	POS	NEG	AMB	NEG	POS	NEG	POS	AMB	POS	POS	NEG
ST254-MRSA, Hannover EMRSA	POS	POS	NEG	AMB	NEG	POS	NEG	POS	AMB	POS	POS	NEG
ST254-MRSA-IV/V, Hannover/UK-10	POS	POS	NEG	AMB	NEG	POS	NEG	POS	AMB	POS	POS	NEG
CC9-MRSA-IV	POS	AMB	AMB	NEG	POS	NEG	POS	POS	NEG	POS	POS	NEG
CC12-MRSA, WA-59	POS	NEG	NEG	NEG	NEG	POS	POS	POS	NEG	NEG	AMB	NEG
CC12-MRSA-IV	POS	NEG	NEG	AMB	NEG	POS	POS	POS	NEG	AMB	AMB	NEG
CC20-MRSA-V	POS	NEG	NEG	NEG	POS	NEG	AMB	POS	NEG	AMB	AMB	NEG
ST22-MRSA-IV, Barnim/UK-15	POS	NEG	POS	POS	NEG	NEG	NEG	POS	POS	NEG	NEG	AMB
ST22-MRSA-IV [ACME+]	POS	NEG	POS	POS	NEG	NEG	NEG	AMB	AMB	NEG	NEG	AMB
ST22-MRSA-IV [PVL+]	POS	NEG	POS	POS	NEG	NEG	NEG	AMB	POS	NEG	NEG	POS
ST22-MRSA-V	POS	NEG	AMB	POS	NEG	NEG	NEG	AMB	POS	NEG	NEG	NEG
ST22-MRSA-V [PVL+]	POS	NEG	POS	AMB	NEG	NEG	NEG	POS	POS	NEG	NEG	AMB
ST36-MRSA-II, UK-16	NEG	NEG	POS	POS	NEG	POS	NEG	NEG	POS	NEG	NEG	POS
ST30-MRSA-IV [PVL+], Southwest Pacific Clone	NEG	NEG	POS	POS	NEG	POS	NEG	NEG	POS	NEG	AMB	COMM
ST30-MRSA-IV [tst1+]	NEG	NEG	POS	POS	NEG	POS	NEG	NEG	POS	NEG	NEG	POS
CC30-MRSA-V [PVL+]	NEG	NEG	POS	POS	NEG	POS	NEG	NEG	POS	NEG	NEG	NEG
ST45-MRSA-II, USA600	AMB	NEG	POS	NEG	NEG	AMB	NEG	NEG	POS	NEG	NEG	NEG
ST45-MRSA-IV, Berlin EMRSA	AMB	NEG	POS	AMB	NEG	AMB	NEG	AMB	POS	NEG	NEG	NEG
CC45-MRSA-IV [ACME+]	POS	NEG	POS	POS	NEG	POS	NEG	AMB	POS	NEG	NEG	NEG
ST45-MRSA-V, WA-4	AMB	NEG	POS	AMB	NEG	POS	NEG	NEG	POS	NEG	NEG	NEG
CC45/agr IV-MRSA-IV, WA-23	AMB	NEG	POS	AMB	NEG	AMB	NEG	NEG	POS	NEG	NEG	NEG
CC45/agr IV-MRSA-V	AMB	NEG	POS	AMB	NEG	AMB	NEG	NEG	POS	NEG	NEG	NEG
CC45/agr IV-MRSA-IV/V	AMB	NEG	POS	AMB	NEG	AMB	NEG	NEG	POS	NEG	NEG	NEG
CC59-MRSA-IV [PVL+], USA1000	POS	POS	NEG	POS	NEG	POS	NEG	POS	NEG	POS	POS	NEG
ST59-MRSA-IV, WA-73	POS	POS	NEG	POS	NEG	POS	NEG	POS	NEG	POS	POS	NEG
ST87-MRSA-IV, WA-24	POS	POS	NEG	POS	NEG	POS	NEG	POS	NEG	POS	POS	NEG
ST59-MRSA-IV [PVL+], WA-55/56	POS	POS	AMB	POS	NEG	POS	NEG	POS	NEG	POS	POS	NEG
ST59/ST952-MRSA-V(T) [PVL+], Taiwan Clone	POS	POS	AMB	POS	NEG	POS	NEG	POS	NEG	POS	POS	NEG
ST59-MRSA-V	POS	POS	NEG	POS	NEG	POS	NEG	AMB	NEG	POS	POS	NEG
CC59-MRSA-V [PVL+]	POS	POS	AMB	POS	NEG	POS	NEG	NEG	NEG	POS	POS	NEG
ST59-MRSA-IV/V, WA-15	POS	POS	AMB	POS	NEG	POS	NEG	AMB	NEG	POS	POS	NEG
ST72-MRSA-IV, USA700	POS	POS	NEG	NEG	NEG	NEG	NEG	POS	NEG	POS	POS	NEG
ST72-MRSA-IV [PVL+], WA-44	POS	POS	NEG	NEG	NEG	NEG	NEG	POS	NEG	POS	POS	NEG
CC75-MRSA-IV	POS	NEG	NEG	NEG	POS	NEG	NEG	NEG	NEG	NEG	NEG	NEG
ST883-MRSA-IV, WA-47	POS	NEG	AMB	NEG	AMB	NEG	AMB	NEG	NEG	NEG	NEG	NEG
ST1303-MRSA-IV	POS	NEG	POS	POS	NEG	POS	NEG	NEG	NEG	NEG	NEG	NEG
CC80-MRSA-IV	POS	NEG	NEG	NEG	NEG	POS	POS	POS	NEG	POS	POS	NEG
CC80-MRSA-IV [PVL+], European Clone	POS	NEG	NEG	NEG	NEG	POS	POS	POS	NEG	POS	POS	NEG
CC88-MRSA-IV, WA-2	POS	NEG	AMB	AMB	NEG	POS	NEG	POS	NEG	POS	POS	NEG
CC88-MRSA-IV [PVL+]	POS	NEG	AMB	AMB	NEG	POS	NEG	POS	NEG	POS	POS	NEG
CC88-MRSA-V [PVL+]	POS	NEG	POS	POS	NEG	POS	NEG	POS	AMB	POS	POS	NEG
CC88-MRSA-VI	POS	NEG	POS	AMB	NEG	POS	NEG	POS	AMB	POS	POS	NEG
ST93-MRSA-IV [PVL-]	POS	NEG	NEG	NEG	AMB	NEG	POS	NEG	NEG	NEG	NEG	NEG
ST93-MRSA-IV [PVL+], Queensland Clone	POS	NEG	AMB	NEG	AMB	NEG	POS	NEG	NEG	NEG	NEG	NEG
ST93-MRSA-V [PVL+]	POS	NEG	AMB	NEG	AMB	NEG	POS	NEG	NEG	NEG	NEG	NEG
CC97-MRSA-IV, WA-54	POS	NEG	AMB	NEG	NEG	NEG	POS	POS	NEG	AMB	AMB	NEG
CC97-MRSA-V	POS	NEG	NEG	NEG	NEG	NEG	POS	POS	NEG	AMB	AMB	NEG
CC97-MRSA-V [ACME+]	POS	NEG	NEG	NEG	NEG	NEG	POS	POS	AMB	AMB	AMB	NEG
CC97-MRSA-I/V	POS	NEG	NEG	NEG	NEG	NEG	POS	POS	NEG	AMB	AMB	NEG
CC121-MRSA-V, WA-22	POS	POS	NEG	POS	NEG	POS	NEG	POS	NEG	POS	POS	NEG
CC152-MRSA-V [PVL+]	NEG	NEG	NEG	NEG	POS	NEG	POS	NEG	POS	NEG	NEG	NEG
ST154-MRSA [PVL+]	POS	NEG	POS	NEG	POS	NEG	NEG	POS	NEG	POS	POS	NEG
ST188-MRSA-IV, WA-38/78	POS	NEG	AMB	NEG	POS	NEG	NEG	POS	NEG	POS	POS	NEG
ST188-MRSA-V	POS	NEG	POS	NEG	POS	NEG	NEG	POS	AMB	POS	POS	NEG
ST239-MRSA-III	POS	POS	NEG	AMB	NEG	POS	NEG	POS	AMB	POS	POS	NEG
CC361-MRSA-IV, WA-29	POS	NEG	POS	NEG	POS	NEG	NEG	POS	NEG	POS	POS	NEG
CC361-MRSA-V	POS	NEG	POS	NEG	POS	NEG	NEG	POS	NEG	POS	POS	NEG
CC361-MRSA-VIII, WA-28	POS	NEG	POS	NEG	POS	NEG	NEG	POS	NEG	POS	POS	NEG
CC398-MRSA-IV	POS	POS	NEG	POS	NEG	AMB	NEG	NEG	POS	NEG	NEG	NEG
ST398-MRSA-V	POS	POS	NEG	POS	NEG	POS	NEG	NEG	POS	NEG	NEG	NEG
ST398-MRSA-V [PVL+]	POS	POS	NEG	POS	NEG	POS	NEG	NEG	POS	NEG	NEG	NEG
CC509/ST207-MRSA-V	POS	POS	POS	POS	NEG	POS	NEG	AMB	POS	NEG	NEG	NEG
ST573-MRSA-V, WA-10	POS	POS	NEG	AMB	NEG	POS	NEG	POS	AMB	POS	POS	NEG
ST772-MRSA-V [PVL+], WA-60/Bengal Bay Clone	POS	POS	AMB	AMB	NEG	POS	NEG	POS	AMB	POS	POS	NEG
ST779-MRSA	AMB	NEG	POS	NEG	NEG	NEG	NEG	POS	AMB	POS	AMB	NEG
ST834-MRSA-IV, WA-13	POS	NEG	POS	NEG	NEG	NEG	NEG	POS	NEG	AMB	AMB	NEG
CC913-MRSA-IV	POS	NEG	POS	NEG	POS	NEG	NEG	POS	NEG	POS	POS	NEG
ST1774-MRSA-IV [ACME+]	AMB	NEG	POS	AMB	NEG	POS	NEG	AMB	POS	NEG	NEG	NEG
CC15-MSSA	POS	NEG	AMB	NEG	NEG	POS	NEG	POS	NEG	POS	POS	NEG
ST426-MSSA	POS	POS	NEG	POS	NEG	POS	NEG	AMB	POS	NEG	NEG	AMB

Legend:

Positive :	POS
Ambiguous :	AMB
Negative :	NEG
Rare (in >0 to 33% of isolates) :	RARE
Variable (in >33 to 66% of isolates) :	VAR
Common (in >66 to <100% of isolates) :	COMM

	VIRULENCE : STAPHYLOCOCCAL SUPERANTIGEN/ENTEROTOXIN-LIKE GENES (SET/SSL), continued												
	ssl04/set9	ssl04/set9 (MRSA252, SAR0425)	ssl05/set3 probe 1	ssl05/set3 (RF122, probe-611)	ssl05/set3 (probe 612)	ssl05/set3 (MRSA252)	ssl06/set21	ssl06 (NCTC8325+ MW2)	ssl07/set1	ssl07/set1 (MRSA252)	ssl07/set1 (AF 188836)	ssl08/ set12 (probe 1)	ssl08/ set12 (probe 2)
CC1-MRSA-IV [PVL-], WA-1/57	POS	NEG	POS	NEG	POS	NEG	POS	POS	POS	AMB	NEG	POS	POS
CC1-MRSA-IV [PVL+], USA400	POS	NEG	POS	AMB	POS	NEG	POS	POS	POS	AMB	NEG	POS	POS
CC1-MRSA-IV/SCCfus [PVL-]	POS	NEG	POS	AMB	POS	NEG	POS	POS	POS	AMB	AMB	POS	POS
CC1-MRSA-IV/SCCfus [PVL+]	POS	NEG	POS	AMB	POS	NEG	POS	POS	POS	AMB	AMB	POS	POS
CC1-MRSA-V [PVL+]	POS	NEG	POS	AMB	POS	NEG	POS	POS	AMB	POS	AMB	POS	POS
CC1-MRSA-V/SCCfus [PVL-]	POS	NEG	POS	AMB	POS	NEG	POS	POS	AMB	POS	AMB	POS	POS
CC1-MRSA-V/SCCfus [PVL+]	POS	NEG	POS	AMB	POS	NEG	POS	POS	AMB	POS	AMB	POS	POS
ST228-MRSA-I, South German/Italian MRSA	POS	NEG	POS	AMB	POS	NEG	NEG	NEG	POS	AMB	AMB	POS	POS
ST5-MRSA-I, Geraldine Clone	POS	NEG	POS	AMB	POS	NEG	NEG	NEG	POS	AMB	AMB	POS	POS
CC5-MRSA-Ivar., WA-18/21/48	POS	NEG	POS	AMB	POS	NEG	NEG	NEG	POS	AMB	NEG	POS	POS
CC5-MRSA-II, Rhine-Hesse /UK-3	COMM	NEG	POS	AMB	POS	NEG	NEG	NEG	POS	AMB	AMB	POS	POS
CC5-MRSA-II [ACME+]	POS	NEG	POS	AMB	POS	NEG	NEG	NEG	POS	AMB	AMB	POS	POS
CC5-MRSA-III	POS	NEG	POS	AMB	POS	NEG	NEG	NEG	POS	AMB	NEG	POS	POS
CC5-MRSA-IV, Paediatric clone	POS	NEG	POS	AMB	POS	NEG	NEG	NEG	POS	AMB	NEG	POS	POS
CC5-MRSA-IV [PVL+]	POS	NEG	POS	AMB	POS	NEG	NEG	NEG	POS	AMB	NEG	POS	POS
CC5-MRSA-V	POS	NEG	POS	AMB	POS	NEG	NEG	NEG	POS	AMB	NEG	POS	POS
CC5-MRSA-V [PVL+]	POS	NEG	POS	AMB	POS	NEG	NEG	NEG	POS	AMB	NEG	POS	POS
CC5-MRSA-VI, New Paediatric Clone	POS	NEG	POS	AMB	POS	NEG	NEG	NEG	POS	AMB	NEG	POS	POS
ST5-MRSA-VII (SCC-JCSC6082)	POS	NEG	POS	NEG	POS	NEG	NEG	NEG	POS	NEG	NEG	POS	POS
CC5-MRSA-IVvar, Maltese Clone	POS	NEG	POS	AMB	POS	NEG	NEG	NEG	POS	AMB	AMB	POS	POS
CC5-MRSA-SCC(MRSAZH47)	AMB	NEG	POS	AMB	POS	NEG	NEG	NEG	POS	AMB	NEG	POS	POS
CC5-MRSA-IV/VI	POS	NEG	POS	AMB	POS	NEG	NEG	NEG	POS	AMB	NEG	POS	POS
CC5-MRSA-IV/SCCfus	POS	NEG	POS	AMB	POS	NEG	NEG	NEG	POS	AMB	NEG	POS	POS
CC5/ST835-MRSA, WA-40/46	POS	NEG	POS	AMB	POS	NEG	NEG	NEG	POS	AMB	AMB	POS	POS
ST6-MRSA-IV, WA-51	POS	NEG	POS	POS	AMB	NEG	NEG	NEG	POS	NEG	NEG	POS	POS
ST6-MRSA-V	POS	NEG	POS	AMB	POS	POS	NEG	NEG	POS	AMB	AMB	POS	POS
CC7-MRSA-IV	POS	NEG	POS	NEG	POS	NEG	AMB	POS	POS	AMB	NEG	POS	POS
CC7-MRSA-V	POS	NEG	POS	NEG	POS	NEG	AMB	POS	POS	AMB	NEG	POS	POS
ST250-MRSA-I, Early/Ancestral MRSA	POS	NEG	NEG	NEG	NEG	NEG	NEG	AMB	NEG	NEG	NEG	NEG	NEG
ST247-MRSA-I, North German/Iberian MRSA	POS	NEG	POS	AMB	POS	NEG	AMB	POS	POS	AMB	NEG	POS	POS
CC8-MRSA-IV, UK-14/WA-5	POS	NEG	POS	AMB	POS	NEG	POS	POS	POS	AMB	AMB	POS	POS
CC8-MRSA-IV, Lyon Clone/UK-2	POS	NEG	POS	AMB	POS	NEG	POS	POS	POS	AMB	NEG	POS	POS
CC8-MRSA-IV, UK-6	POS	NEG	POS	AMB	POS	NEG	POS	POS	POS	AMB	AMB	POS	POS
CC8-MRSA-IV, USA500	POS	NEG	POS	AMB	POS	NEG	AMB	POS	POS	AMB	AMB	POS	POS
ST8-MRSA-IV [PVL/ACME+], USA300	POS	NEG	POS	AMB	POS	NEG	AMB	POS	POS	AMB	NEG	POS	POS
ST8-MRSA-IV [PVL+], USA300	POS	NEG	POS	NEG	POS	NEG	POS	POS	POS	AMB	NEG	POS	POS
CC8-MRSA-IV [PVL/sed/j/r/k/q+]	POS	NEG	POS	AMB	POS	NEG	POS	POS	AMB	POS	AMB	POS	POS
CC8-MRSA-IV [tst1/sec/sel+]	POS	NEG	POS	POS	AMB	NEG	POS	POS	AMB	POS	AMB	POS	POS
CC8-MRSA-V	POS	NEG	POS	AMB	POS	NEG	AMB	POS	POS	AMB	NEG	POS	POS
CC8-MRSA-VIII	POS	NEG	POS	NEG	POS	NEG	AMB	POS	POS	AMB	NEG	POS	POS
ST8-MRSA-IIA/B/C/D/E, Irish AR13/14	POS	NEG	POS	AMB	POS	NEG	POS	POS	POS	AMB	NEG	POS	POS
ST8-MRSA, UK-12/13, Irish AR43	POS	NEG	POS	AMB	POS	NEG	POS	POS	POS	AMB	AMB	POS	POS
ST254-MRSA, Hannover EMRSA	POS	NEG	POS	AMB	POS	NEG	NEG	POS	POS	AMB	AMB	POS	POS
ST254-MRSA-IV/V, Hannover/UK-10	POS	NEG	POS	AMB	POS	NEG	AMB	POS	POS	AMB	AMB	POS	POS
CC9-MRSA-IV	POS	NEG	NEG	AMB	POS	NEG	POS	POS	POS	AMB	NEG	POS	POS
CC12-MRSA, WA-59	POS	NEG	POS	NEG	POS	NEG	NEG	NEG	POS	NEG	NEG	POS	POS
CC12-MRSA-IV	POS	NEG	POS	AMB	POS	NEG	NEG	NEG	POS	NEG	NEG	POS	POS
CC20-MRSA-V	POS	NEG	NEG	AMB	POS	NEG	AMB	POS	POS	AMB	NEG	POS	POS
ST22-MRSA-IV, Barnim/UK-15	NEG	POS	POS	NEG	NEG	POS	NEG	NEG	AMB	AMB	POS	NEG	NEG
ST22-MRSA-IV [ACME+]	NEG	POS	POS	POS	NEG	POS	NEG	AMB	AMB	AMB	POS	NEG	NEG
ST22-MRSA-IV [PVL+]	NEG	POS	POS	AMB	NEG	POS	NEG	AMB	AMB	POS	AMB	NEG	NEG
ST22-MRSA-V	NEG	AMB	POS	NEG	NEG	POS	NEG	AMB	AMB	AMB	POS	NEG	NEG
ST22-MRSA-V [PVL+]	NEG	AMB	POS	NEG	NEG	POS	NEG	AMB	AMB	AMB	POS	NEG	NEG
ST36-MRSA-II, UK-16	NEG	POS	NEG	NEG	NEG	POS	NEG	AMB	NEG	POS	AMB	NEG	NEG
ST30-MRSA-IV [PVL+], Southwest Pacific Clone	NEG	COMM	NEG	NEG	NEG	POS	NEG	AMB	NEG	POS	AMB	NEG	NEG
ST30-MRSA-IV [tst1+]	NEG	POS	NEG	NEG	NEG	POS	NEG	AMB	NEG	POS	NEG	NEG	NEG
CC30-MRSA-V [PVL+]	NEG	NEG	NEG	NEG	NEG	POS	NEG	POS	AMB	POS	AMB	NEG	NEG
ST45-MRSA-II, USA600	NEG	POS	NEG	NEG	NEG	POS	NEG	NEG	NEG	NEG	NEG	NEG	NEG
ST45-MRSA-IV, Berlin EMRSA	NEG	AMB	NEG	NEG	NEG	POS	NEG	AMB	NEG	NEG	NEG	NEG	NEG
CC45-MRSA-IV [ACME+]	NEG	POS	NEG	NEG	NEG	POS	NEG	AMB	NEG	NEG	AMB	NEG	NEG
ST45-MRSA-V, WA-4	NEG	POS	NEG	NEG	NEG	POS	NEG	AMB	NEG	NEG	NEG	NEG	NEG
CC45/agr IV-MRSA-IV, WA-23	NEG	POS	NEG	NEG	NEG	POS	NEG	AMB	NEG	NEG	POS	NEG	NEG
CC45/agr IV-MRSA-V	NEG	POS	NEG	NEG	NEG	POS	NEG	AMB	NEG	AMB	POS	NEG	NEG
CC45/agr IV-MRSA-IV/V	NEG	POS	NEG	NEG	NEG	POS	NEG	AMB	NEG	NEG	POS	NEG	NEG
CC59-MRSA-IV [PVL+], USA1000	POS	NEG	POS	POS	AMB	NEG	NEG	NEG	POS	AMB	AMB	POS	POS
ST59-MRSA-IV, WA-73	POS	NEG	POS	POS	AMB	NEG	NEG	NEG	POS	AMB	AMB	POS	POS
ST87-MRSA-IV, WA-24	POS	NEG	POS	POS	AMB	NEG	NEG	NEG	POS	AMB	NEG	POS	POS
ST59-MRSA-IV [PVL+], WA-55/56	POS	NEG	POS	AMB	AMB	AMB	NEG	NEG	AMB	AMB	AMB	POS	POS
ST59/ST952-MRSA-V(T) [PVL+], Taiwan Clone	POS	NEG	POS	POS	AMB	NEG	NEG	NEG	POS	AMB	AMB	POS	POS
ST59-MRSA-V	POS	NEG	POS	POS	AMB	NEG	NEG	NEG	POS	AMB	NEG	POS	POS
CC59-MRSA-V [PVL+]	POS	NEG	POS	POS	NEG	NEG	NEG	NEG	POS	AMB	NEG	POS	POS
ST59-MRSA-IV/V, WA-15	POS	NEG	POS	POS	AMB	NEG	NEG	NEG	POS	AMB	AMB	POS	POS
ST72-MRSA-IV, USA700	POS	NEG	POS	POS	AMB	NEG	NEG	NEG	POS	AMB	NEG	POS	POS
ST72-MRSA-IV [PVL+], WA-44	POS	NEG	POS	AMB	AMB	NEG	NEG	NEG	POS	AMB	AMB	POS	POS
CC75-MRSA-IV	NEG	AMB	NEG	AMB	NEG	NEG	NEG	NEG	POS	NEG	NEG	NEG	NEG
ST883-MRSA-IV, WA-47	AMB	AMB	NEG	NEG	NEG	NEG	NEG	AMB	AMB	NEG	NEG	POS	POS
ST1303-MRSA-IV	NEG	AMB	NEG	NEG	NEG	NEG	NEG	POS	POS	AMB	NEG	NEG	POS
CC80-MRSA-IV	POS	NEG	POS	POS	AMB	NEG	NEG	NEG	POS	AMB	NEG	POS	POS
CC80-MRSA-IV [PVL+], European Clone	POS	NEG	POS	POS	AMB	NEG	NEG	NEG	POS	AMB	NEG	POS	POS
CC88-MRSA-IV, WA-2	POS	NEG	NEG	AMB	POS	NEG	POS	POS	POS	AMB	NEG	POS	POS
CC88-MRSA-IV [PVL+]	POS	NEG	NEG	AMB	POS	NEG	POS	POS	POS	AMB	AMB	POS	POS
CC88-MRSA-V [PVL+]	POS	AMB	NEG	AMB	POS	NEG	POS	POS	AMB	POS	AMB	POS	POS
CC88-MRSA-VI	POS	NEG	NEG	AMB	POS	NEG	POS	POS	POS	AMB	AMB	POS	POS
ST93-MRSA-IV [PVL-]	NEG	NEG	NEG	NEG	NEG	NEG	POS	AMB	NEG	NEG	NEG	NEG	AMB
ST93-MRSA-IV [PVL+], Queensland Clone	NEG	AMB	NEG	AMB	NEG	NEG	POS	AMB	NEG	NEG	NEG	AMB	AMB
ST93-MRSA-V [PVL+]	NEG	AMB	NEG	AMB	NEG	NEG	POS	AMB	NEG	NEG	NEG	AMB	AMB
CC97-MRSA-IV, WA-54	POS	NEG	POS	POS	AMB	NEG	NEG	NEG	POS	AMB	NEG	POS	POS
CC97-MRSA-V	POS	NEG	POS	POS	AMB	NEG	NEG	NEG	POS	AMB	NEG	POS	POS
CC97-MRSA-V [ACME+]	POS	NEG	POS	POS	AMB	NEG	NEG	AMB	POS	AMB	AMB	POS	POS
CC97-MRSA-I/V	POS	NEG	POS	POS	AMB	NEG	NEG	NEG	POS	AMB	NEG	POS	POS
CC121-MRSA-V, WA-22	POS	NEG	NEG	POS	AMB	NEG	POS	POS	POS	AMB	AMB	POS	POS
CC152-MRSA-V [PVL+]	NEG	NEG	NEG	NEG	NEG	NEG	NEG	NEG	NEG	NEG	NEG	NEG	NEG
ST154-MRSA [PVL+]	POS	NEG	POS	AMB	POS	NEG	NEG	NEG	POS	AMB	NEG	POS	POS
ST188-MRSA-IV, WA-38/78	POS	NEG	POS	AMB	POS	NEG	POS	POS	POS	AMB	NEG	POS	POS
ST188-MRSA-V	POS	NEG	POS	AMB	POS	NEG	POS	POS	AMB	POS	AMB	POS	POS
ST239-MRSA-III	POS	NEG	POS	AMB	POS	NEG	POS	POS	POS	AMB	AMB	POS	POS
CC361-MRSA-IV, WA-29	POS	NEG	POS	POS	AMB	NEG	NEG	NEG	POS	AMB	AMB	POS	POS
CC361-MRSA-V	POS	NEG	POS	POS	AMB	NEG	NEG	NEG	POS	AMB	NEG	POS	POS
CC361-MRSA-VIII, WA-28	POS	NEG	POS	POS	NEG	NEG	NEG	NEG	POS	AMB	AMB	POS	POS
CC398-MRSA-IV	NEG	POS	NEG	NEG	NEG	POS	NEG	AMB	AMB	AMB	POS	NEG	NEG
ST398-MRSA-V	NEG	POS	NEG	NEG	NEG	POS	NEG	NEG	AMB	AMB	POS	NEG	NEG
ST398-MRSA-V [PVL+]	NEG	POS	NEG	NEG	NEG	POS	NEG	NEG	NEG	AMB	POS	NEG	NEG
CC509/ST207-MRSA-V	NEG	POS	POS	NEG	NEG	POS	NEG	NEG	AMB	POS	POS	NEG	NEG
ST573-MRSA-V, WA-10	POS	NEG	POS	POS	POS	NEG	NEG	NEG	POS	NEG	NEG	POS	POS
ST772-MRSA-V [PVL+], WA-60/Bengal Bay Clone	POS	NEG	POS	POS	AMB	NEG	NEG	NEG	POS	NEG	NEG	POS	POS
ST779-MRSA	POS	NEG	POS	AMB	POS	NEG	NEG	NEG	POS	AMB	AMB	POS	POS
ST834-MRSA-IV, WA-13	POS	NEG	POS	POS	AMB	NEG	NEG	NEG	POS	AMB	NEG	POS	POS
CC913-MRSA-IV	POS	NEG	POS	POS	POS	NEG	NEG	POS	POS	AMB	NEG	POS	POS
ST1774-MRSA-IV [ACME+]	NEG	AMB	NEG	NEG	NEG	POS	NEG	AMB	NEG	AMB	POS	NEG	NEG

CC15-MSSA	POS	NEG	POS	POS	AMB	NEG	POS	POS	POS	AMB	NEG	AMB	POS
ST426-MSSA	NEG	POS	NEG	NEG	NEG	POS	NEG	AMB	AMB	AMB	POS	NEG	NEG

Legend:

Positive :	POS
Ambiguous :	AMB
Negative :	NEG
Rare (in >0 to 33% of isolates) :	RARE
Variable (in >33 to 66% of isolates) :	VAR
Common (in >66 to <100% of isolates) :	COMM

	VIRULENCE : STAPHYLOCOCCAL SUPERANTIGEN/ENTEROTOXIN-LIKE GENES (SET/SSL), continued														
	ssl09/set5 (probe 1)	ssl09/set5 (probe 2)	ssl09/set5 (MRSA252)	ssl10/set4	ssl10 (RF122)	ssl10/set4 (MRSA252)	ssl11/set2 (COL)	ssl11/set2 (Mu50+N315)	ssl11/set2 (MW2+MSSA476)	ssl11/set2 (MRSA252)	setB3	setB3 (MRSA252)	setB2	setB2 (MRSA252)	setB1
CC1-MRSA-IV [PVL-], WA-1/57	POS	POS	NEG	POS	NEG	NEG	NEG	NEG	POS	NEG	POS	NEG	POS	NEG	POS
CC1-MRSA-IV [PVL+], USA400	POS	POS	NEG	POS	NEG	NEG	NEG	NEG	POS	NEG	POS	NEG	POS	NEG	POS
CC1-MRSA-IV/SCCfus [PVL-]	POS	POS	NEG	POS	NEG	AMB	NEG	NEG	POS	NEG	POS	NEG	POS	NEG	POS
CC1-MRSA-IV/SCCfus [PVL+]	AMB	POS	NEG	POS	NEG	AMB	NEG	NEG	POS	NEG	POS	NEG	POS	NEG	POS
CC1-MRSA-V [PVL+]	POS	POS	NEG	POS	AMB	AMB	NEG	NEG	POS	NEG	POS	NEG	POS	NEG	POS
CC1-MRSA-V/SCCfus [PVL-]	POS	POS	NEG	POS	AMB	AMB	NEG	NEG	POS	NEG	POS	NEG	POS	NEG	POS
CC1-MRSA-V/SCCfus [PVL+]	POS	POS	NEG	POS	AMB	AMB	NEG	NEG	POS	NEG	POS	NEG	POS	NEG	POS
ST228-MRSA-I, South German/Italian MRSA	POS	AMB	NEG	POS	NEG	AMB	NEG	POS	NEG	NEG	POS	NEG	POS	NEG	POS
ST5-MRSA-I, Geraldine Clone	POS	POS	NEG	POS	NEG	AMB	NEG	POS	NEG	NEG	POS	NEG	POS	NEG	POS
CC5-MRSA-Ivar., WA-18/21/48	POS	POS	NEG	POS	NEG	NEG	NEG	POS	NEG	NEG	POS	NEG	POS	NEG	POS
CC5-MRSA-II, Rhine-Hesse /UK-3	POS	POS	NEG	POS	NEG	NEG	NEG	POS	NEG	NEG	POS	NEG	POS	NEG	POS
CC5-MRSA-II [ACME+]	POS	POS	NEG	POS	AMB	AMB	NEG	POS	NEG	NEG	POS	NEG	POS	NEG	POS
CC5-MRSA-III	POS	POS	NEG	POS	NEG	AMB	NEG	POS	NEG	NEG	POS	NEG	POS	NEG	POS
CC5-MRSA-IV, Paediatric clone	POS	POS	NEG	POS	NEG	AMB	NEG	POS	NEG	NEG	POS	NEG	POS	NEG	POS
CC5-MRSA-IV [PVL+]	COMM	COMM	NEG	POS	NEG	AMB	NEG	POS	NEG	NEG	POS	NEG	POS	NEG	POS
CC5-MRSA-V	POS	POS	NEG	POS	NEG	NEG	NEG	POS	NEG	NEG	POS	NEG	POS	NEG	POS
CC5-MRSA-V [PVL+]	POS	POS	NEG	POS	NEG	AMB	NEG	POS	NEG	NEG	POS	NEG	POS	NEG	POS
CC5-MRSA-VI, New Paediatric Clone	POS	POS	NEG	POS	NEG	NEG	NEG	POS	NEG	NEG	POS	NEG	POS	NEG	POS
ST5-MRSA-VII (SCC-JCSC6082)	AMB	POS	NEG	POS	NEG	NEG	NEG	POS	NEG	NEG	POS	NEG	POS	NEG	POS
CC5-MRSA-IVvar, Maltese Clone	POS	POS	NEG	POS	AMB	AMB	NEG	POS	NEG	NEG	POS	NEG	POS	NEG	POS
CC5-MRSA-SCC(MRSAZH47)	POS	POS	NEG	POS	NEG	AMB	NEG	POS	NEG	NEG	POS	NEG	POS	NEG	POS
CC5-MRSA-IV/VI	POS	POS	NEG	POS	NEG	NEG	NEG	POS	NEG	NEG	POS	NEG	POS	NEG	POS
CC5-MRSA-IV/SCCfus	POS	POS	NEG	POS	NEG	NEG	NEG	POS	NEG	NEG	POS	NEG	POS	NEG	POS
CC5/ST835-MRSA, WA-40/46	POS	AMB	NEG	POS	NEG	NEG	NEG	POS	NEG	NEG	POS	NEG	POS	NEG	POS
ST6-MRSA-IV, WA-51	POS	POS	NEG	AMB	POS	NEG	NEG	POS	NEG	NEG	POS	NEG	POS	NEG	POS
ST6-MRSA-V	POS	POS	NEG	POS	AMB	AMB	NEG	POS	NEG	NEG	POS	NEG	POS	NEG	POS
CC7-MRSA-IV	POS	POS	NEG	POS	NEG	NEG	NEG	NEG	NEG	NEG	POS	NEG	POS	NEG	POS
CC7-MRSA-V	POS	POS	NEG	POS	NEG	NEG	NEG	NEG	NEG	NEG	POS	NEG	POS	NEG	POS
ST250-MRSA-I, Early/Ancestral MRSA	POS	POS	NEG	POS	NEG	NEG	POS	NEG	NEG	NEG	POS	NEG	POS	NEG	POS
ST247-MRSA-I, North German/Iberian MRSA	POS	POS	NEG	POS	NEG	NEG	POS	NEG	NEG	NEG	POS	NEG	POS	NEG	POS
CC8-MRSA-IV, UK-14/WA-5	POS	POS	NEG	POS	AMB	AMB	POS	NEG	NEG	NEG	POS	NEG	POS	NEG	POS
CC8-MRSA-IV, Lyon Clone/UK-2	POS	POS	NEG	POS	NEG	AMB	POS	NEG	NEG	NEG	POS	NEG	POS	NEG	POS
CC8-MRSA-IV, UK-6	POS	POS	NEG	POS	NEG	AMB	POS	NEG	NEG	NEG	POS	NEG	POS	NEG	POS
CC8-MRSA-IV, USA500	POS	POS	NEG	POS	NEG	NEG	POS	NEG	NEG	NEG	POS	NEG	POS	NEG	POS
ST8-MRSA-IV [PVL/ACME+], USA300	POS	POS	NEG	POS	NEG	NEG	POS	NEG	NEG	NEG	POS	NEG	POS	NEG	POS
ST8-MRSA-IV [PVL+], USA300	POS	POS	NEG	POS	NEG	NEG	POS	NEG	NEG	NEG	POS	NEG	POS	NEG	POS
CC8-MRSA-IV [PVL/sed/j/r/k/q+]	POS	POS	NEG	POS	AMB	AMB	POS	NEG	NEG	NEG	POS	NEG	POS	NEG	POS
CC8-MRSA-IV [tst1/sec/sel+]	POS	POS	NEG	POS	AMB	AMB	POS	NEG	NEG	NEG	POS	NEG	POS	NEG	POS
CC8-MRSA-V	POS	POS	NEG	POS	NEG	NEG	POS	NEG	NEG	NEG	POS	NEG	POS	NEG	POS
CC8-MRSA-VIII	POS	AMB	NEG	POS	NEG	NEG	POS	NEG	NEG	NEG	POS	NEG	POS	NEG	POS
ST8-MRSA-IIA/B/C/D/E, Irish AR13/14	POS	POS	NEG	POS	NEG	NEG	POS	NEG	NEG	NEG	POS	NEG	POS	NEG	POS
ST8-MRSA, UK-12/13, Irish AR43	POS	POS	NEG	POS	NEG	AMB	POS	NEG	NEG	NEG	POS	NEG	POS	NEG	POS
ST254-MRSA, Hannover EMRSA	POS	POS	NEG	POS	AMB	NEG	POS	NEG	NEG	NEG	POS	NEG	POS	NEG	POS
ST254-MRSA-IV/V, Hannover/UK-10	POS	POS	NEG	POS	AMB	AMB	POS	NEG	NEG	NEG	POS	NEG	POS	NEG	POS
CC9-MRSA-IV	POS	POS	NEG	POS	NEG	AMB	NEG	NEG	NEG	NEG	POS	NEG	POS	NEG	POS
CC12-MRSA, WA-59	POS	POS	NEG	POS	NEG	NEG	POS	NEG	NEG	NEG	POS	NEG	POS	NEG	POS
CC12-MRSA-IV	POS	POS	NEG	POS	NEG	AMB	POS	NEG	NEG	NEG	POS	NEG	POS	NEG	POS
CC20-MRSA-V	POS	AMB	NEG	POS	NEG	NEG	NEG	NEG	NEG	NEG	POS	NEG	POS	NEG	POS
ST22-MRSA-IV, Barnim/UK-15	AMB	POS	NEG	AMB	NEG	NEG	NEG	NEG	NEG	NEG	NEG	NEG	NEG	NEG	NEG
ST22-MRSA-IV [ACME+]	POS	POS	NEG	AMB	NEG	AMB	NEG	NEG	NEG	NEG	NEG	NEG	NEG	NEG	AMB
ST22-MRSA-IV [PVL+]	POS	POS	NEG	AMB	NEG	AMB	NEG	NEG	NEG	NEG	NEG	NEG	NEG	NEG	AMB
ST22-MRSA-V	POS	POS	NEG	AMB	NEG	NEG	NEG	NEG	NEG	NEG	NEG	NEG	NEG	NEG	NEG
ST22-MRSA-V [PVL+]	AMB	AMB	NEG	NEG	NEG	NEG	NEG	NEG	NEG	NEG	NEG	NEG	NEG	NEG	NEG
ST36-MRSA-II, UK-16	NEG	NEG	POS	AMB	NEG	POS	NEG	NEG	NEG	POS	NEG	POS	NEG	POS	POS
ST30-MRSA-IV [PVL+], Southwest Pacific Clone	NEG	NEG	POS	AMB	NEG	POS	NEG	NEG	NEG	POS	NEG	POS	NEG	POS	POS
ST30-MRSA-IV [tst1+]	NEG	NEG	POS	AMB	NEG	POS	NEG	NEG	NEG	POS	NEG	POS	NEG	POS	POS
CC30-MRSA-V [PVL+]	NEG	NEG	POS	AMB	NEG	POS	NEG	NEG	NEG	POS	NEG	POS	NEG	POS	POS
ST45-MRSA-II, USA600	NEG	NEG	POS	AMB	NEG	POS	NEG	NEG	NEG	NEG	NEG	POS	NEG	AMB	AMB
ST45-MRSA-IV, Berlin EMRSA	NEG	NEG	POS	AMB	NEG	POS	NEG	NEG	NEG	NEG	NEG	POS	NEG	AMB	NEG
CC45-MRSA-IV [ACME+]	NEG	NEG	POS	AMB	NEG	POS	NEG	NEG	NEG	NEG	NEG	POS	NEG	AMB	NEG
ST45-MRSA-V, WA-4	NEG	NEG	POS	AMB	NEG	POS	NEG	NEG	NEG	NEG	NEG	POS	NEG	AMB	NEG
CC45/agr IV-MRSA-IV, WA-23	NEG	NEG	POS	AMB	NEG	POS	NEG	NEG	NEG	NEG	NEG	POS	NEG	AMB	NEG
CC45/agr IV-MRSA-V	NEG	NEG	POS	AMB	NEG	POS	NEG	NEG	NEG	NEG	NEG	POS	NEG	AMB	NEG
CC45/agr IV-MRSA-IV/V	NEG	NEG	POS	AMB	NEG	POS	NEG	NEG	NEG	NEG	NEG	POS	NEG	NEG	NEG
CC59-MRSA-IV [PVL+], USA1000	POS	POS	NEG	AMB	POS	NEG	NEG	NEG	NEG	NEG	POS	NEG	POS	NEG	POS
ST59-MRSA-IV, WA-73	POS	AMB	NEG	AMB	POS	NEG	NEG	NEG	NEG	NEG	POS	NEG	POS	NEG	POS
ST87-MRSA-IV, WA-24	POS	AMB	NEG	POS	POS	NEG	NEG	NEG	NEG	NEG	POS	NEG	POS	NEG	POS
ST59-MRSA-IV [PVL+], WA-55/56	POS	POS	NEG	POS	POS	NEG	NEG	NEG	NEG	NEG	POS	NEG	POS	NEG	POS
ST59/ST952-MRSA-V(T) [PVL+], Taiwan Clone	POS	POS	NEG	POS	POS	NEG	NEG	NEG	NEG	NEG	POS	NEG	POS	NEG	POS
ST59-MRSA-V	POS	AMB	NEG	POS	POS	NEG	NEG	NEG	NEG	NEG	POS	NEG	POS	NEG	POS
CC59-MRSA-V [PVL+]	AMB	POS	NEG	AMB	POS	NEG	NEG	NEG	NEG	NEG	POS	NEG	POS	NEG	POS
ST59-MRSA-IV/V, WA-15	POS	POS	NEG	POS	POS	NEG	NEG	NEG	NEG	NEG	POS	NEG	POS	NEG	POS
ST72-MRSA-IV, USA700	POS	POS	NEG	POS	NEG	AMB	NEG	NEG	NEG	NEG	POS	NEG	POS	NEG	POS
ST72-MRSA-IV [PVL+], WA-44	POS	AMB	NEG	POS	AMB	NEG	NEG	NEG	NEG	NEG	POS	NEG	POS	NEG	POS
CC75-MRSA-IV	NEG	NEG	NEG	AMB	NEG	AMB	NEG	NEG	NEG	NEG	NEG	NEG	NEG	NEG	NEG
ST883-MRSA-IV, WA-47	AMB	POS	NEG	POS	NEG	AMB	NEG	AMB	NEG	NEG	NEG	NEG	NEG	NEG	NEG
ST1303-MRSA-IV	POS	POS	NEG	POS	NEG	NEG	NEG	NEG	NEG	NEG	NEG	NEG	NEG	NEG	NEG
CC80-MRSA-IV	POS	POS	NEG	POS	NEG	AMB	NEG	NEG	NEG	NEG	POS	NEG	POS	NEG	POS
CC80-MRSA-IV [PVL+], European Clone	POS	POS	NEG	POS	NEG	NEG	NEG	NEG	NEG	NEG	POS	NEG	POS	NEG	POS
CC88-MRSA-IV, WA-2	POS	POS	NEG	POS	NEG	AMB	NEG	NEG	NEG	AMB	POS	NEG	POS	NEG	POS
CC88-MRSA-IV [PVL+]	POS	POS	NEG	POS	NEG	AMB	NEG	NEG	NEG	AMB	POS	NEG	POS	NEG	POS
CC88-MRSA-V [PVL+]	POS	POS	NEG	POS	AMB	AMB	NEG	NEG	NEG	AMB	POS	NEG	POS	NEG	POS
CC88-MRSA-VI	POS	POS	NEG	POS	AMB	AMB	NEG	NEG	NEG	AMB	POS	NEG	POS	NEG	POS
ST93-MRSA-IV [PVL-]	NEG	NEG	NEG	NEG	NEG	NEG	NEG	NEG	NEG	NEG	NEG	AMB	NEG	NEG	NEG
ST93-MRSA-IV [PVL+], Queensland Clone	NEG	NEG	NEG	AMB	NEG	AMB	NEG	NEG	NEG	NEG	NEG	POS	NEG	NEG	NEG
ST93-MRSA-V [PVL+]	NEG	NEG	NEG	AMB	NEG	AMB	NEG	NEG	NEG	NEG	NEG	POS	NEG	NEG	NEG
CC97-MRSA-IV, WA-54	POS	POS	NEG	POS	NEG	NEG	NEG	NEG	NEG	NEG	POS	NEG	POS	NEG	POS
CC97-MRSA-V	POS	POS	NEG	POS	NEG	NEG	NEG	NEG	NEG	NEG	POS	NEG	POS	NEG	POS
CC97-MRSA-V [ACME+]	POS	POS	NEG	POS	AMB	AMB	NEG	NEG	NEG	NEG	POS	NEG	POS	NEG	POS
CC97-MRSA-I/V	POS	POS	NEG	POS	NEG	AMB	NEG	NEG	NEG	NEG	POS	NEG	POS	NEG	POS
CC121-MRSA-V, WA-22	POS	NEG	NEG	POS	POS	AMB	NEG	NEG	NEG	NEG	POS	NEG	POS	NEG	POS
CC152-MRSA-V [PVL+]	NEG	NEG	NEG	AMB	NEG	NEG	NEG	NEG	NEG	NEG	POS	NEG	POS	NEG	POS
ST154-MRSA [PVL+]	POS	POS	NEG	POS	NEG	NEG	NEG	NEG	NEG	NEG	POS	NEG	POS	NEG	POS
ST188-MRSA-IV, WA-38/78	POS	AMB	NEG	POS	AMB	AMB	NEG	NEG	POS	NEG	POS	NEG	POS	NEG	POS
ST188-MRSA-V	POS	POS	NEG	POS	AMB	AMB	NEG	NEG	POS	NEG	POS	NEG	POS	NEG	POS
ST239-MRSA-III	POS	POS	NEG	POS	NEG	AMB	POS	NEG	NEG	NEG	POS	NEG	POS	NEG	POS
CC361-MRSA-IV, WA-29	POS	AMB	NEG	POS	NEG	AMB	NEG	NEG	NEG	NEG	POS	NEG	POS	NEG	POS
CC361-MRSA-V	POS	POS	NEG	POS	NEG	NEG	NEG	NEG	NEG	NEG	POS	NEG	POS	NEG	POS
CC361-MRSA-VIII, WA-28	POS	NEG	NEG	POS	NEG	NEG	NEG	NEG	NEG	NEG	POS	NEG	POS	NEG	AMB
CC398-MRSA-IV	NEG	NEG	POS	AMB	NEG	POS	NEG	NEG	NEG	AMB	NEG	POS	NEG	POS	POS
ST398-MRSA-V	NEG	NEG	POS	AMB	NEG	POS	NEG	NEG	NEG	AMB	NEG	POS	NEG	POS	POS
ST398-MRSA-V [PVL+]	NEG	NEG	NEG	AMB	NEG	POS	NEG	NEG	NEG	AMB	NEG	POS	NEG	POS	POS
CC509/ST207-MRSA-V	NEG	NEG	NEG	POS	NEG	NEG	NEG	NEG	NEG	NEG	NEG	NEG	NEG	NEG	NEG
ST573-MRSA-V, WA-10	POS	POS	NEG	POS	NEG	NEG	NEG	NEG	NEG	NEG	POS	NEG	POS	NEG	POS
ST772-MRSA-V [PVL+], WA-60/Bengal Bay Clone	POS	POS	NEG	POS	NEG	NEG	NEG	NEG	NEG	NEG	POS	NEG	POS	NEG	POS
ST779-MRSA	POS	AMB	NEG	POS	AMB	AMB	NEG	NEG	NEG	NEG	POS	NEG	POS	NEG	POS
ST834-MRSA-IV, WA-13	POS	AMB	NEG	POS	NEG	NEG	NEG	NEG	NEG	NEG	POS	NEG	POS	NEG	POS
CC913-MRSA-IV	POS	NEG	NEG	POS	POS	NEG	NEG	NEG	NEG	NEG	POS	NEG	POS	NEG	POS
ST1774-MRSA-IV [ACME+]	NEG	NEG	POS	AMB	AMB	POS	NEG	NEG	NEG	NEG	POS	NEG	POS	NEG	POS

CC15-MSSA	POS	POS	NEG	AMB	POS	NEG	NEG	NEG	NEG	POS	POS	NEG	POS	NEG	POS
ST426-MSSA	NEG	NEG	POS	AMB	NEG	POS	NEG	NEG	NEG	NEG	NEG	POS	NEG	AMB	NEG

Legend:

Positive :	POS
Ambiguous :	AMB
Negative :	NEG
Rare (in >0 to 33% of isolates) :	RARE
Variable (in >33 to 66% of isolates) :	VAR
Common (in >66 to <100% of isolates) :	COMM

	CAPSULE- AND BIOFILM-ASSOCIATED GENES													
	capH1	capJ1	capK1	capH5	capJ5	capK5	capH8	capI8	capJ8	capK8	icaA	icaC	icaD	bap
CC1-MRSA-IV [PVL-], WA-1/57	NEG	NEG	NEG	NEG	NEG	NEG	POS	POS	POS	POS	POS	POS	POS	NEG
CC1-MRSA-IV [PVL+], USA400	NEG	NEG	NEG	NEG	NEG	NEG	POS	POS	POS	POS	POS	POS	POS	NEG
CC1-MRSA-IV/SCCfus [PVL-]	NEG	NEG	NEG	NEG	NEG	NEG	POS	POS	POS	POS	POS	POS	POS	NEG
CC1-MRSA-IV/SCCfus [PVL+]	NEG	NEG	NEG	NEG	NEG	NEG	POS	POS	POS	POS	POS	POS	POS	NEG
CC1-MRSA-V [PVL+]	NEG	NEG	NEG	NEG	NEG	NEG	POS	POS	POS	POS	POS	POS	POS	NEG
CC1-MRSA-V/SCCfus [PVL-]	NEG	NEG	NEG	NEG	NEG	NEG	POS	POS	POS	POS	POS	POS	POS	NEG
CC1-MRSA-V/SCCfus [PVL+]	NEG	NEG	NEG	NEG	NEG	NEG	POS	POS	POS	POS	POS	POS	POS	NEG
ST228-MRSA-I, South German/Italian MRSA	NEG	NEG	NEG	POS	POS	POS	NEG	NEG	NEG	NEG	POS	POS	POS	NEG
ST5-MRSA-I, Geraldine Clone	NEG	NEG	NEG	POS	POS	POS	NEG	NEG	NEG	NEG	POS	POS	POS	NEG
CC5-MRSA-Ivar., WA-18/21/48	NEG	NEG	NEG	POS	POS	POS	NEG	NEG	NEG	NEG	POS	POS	POS	NEG
CC5-MRSA-II, Rhine-Hesse /UK-3	NEG	NEG	NEG	POS	POS	POS	NEG	NEG	NEG	NEG	POS	POS	POS	NEG
CC5-MRSA-II [ACME+]	NEG	NEG	NEG	POS	POS	POS	NEG	NEG	NEG	NEG	POS	POS	POS	NEG
CC5-MRSA-III	NEG	NEG	NEG	POS	POS	POS	NEG	NEG	NEG	NEG	POS	POS	POS	NEG
CC5-MRSA-IV, Paediatric clone	NEG	NEG	NEG	POS	POS	POS	NEG	NEG	NEG	NEG	POS	POS	POS	NEG
CC5-MRSA-IV [PVL+]	NEG	NEG	NEG	POS	POS	POS	NEG	NEG	NEG	NEG	POS	POS	POS	NEG
CC5-MRSA-V	NEG	NEG	NEG	POS	POS	POS	NEG	NEG	NEG	NEG	POS	POS	POS	NEG
CC5-MRSA-V [PVL+]	NEG	NEG	NEG	POS	POS	POS	NEG	NEG	NEG	NEG	POS	POS	POS	NEG
CC5-MRSA-VI, New Paediatric Clone	NEG	NEG	NEG	POS	POS	POS	NEG	NEG	NEG	NEG	POS	POS	POS	NEG
ST5-MRSA-VII (SCC-JCSC6082)	NEG	NEG	NEG	POS	POS	POS	NEG	NEG	NEG	NEG	POS	POS	POS	NEG
CC5-MRSA-IVvar, Maltese Clone	NEG	NEG	NEG	POS	POS	POS	NEG	NEG	NEG	NEG	POS	POS	POS	NEG
CC5-MRSA-SCC(MRSAZH47)	NEG	NEG	NEG	POS	POS	POS	NEG	NEG	NEG	NEG	POS	POS	POS	NEG
CC5-MRSA-IV/VI	NEG	NEG	NEG	POS	POS	POS	NEG	NEG	NEG	NEG	POS	POS	POS	NEG
CC5-MRSA-IV/SCCfus	NEG	NEG	NEG	POS	POS	POS	NEG	NEG	NEG	NEG	POS	POS	POS	NEG
CC5/ST835-MRSA, WA-40/46	NEG	NEG	NEG	POS	POS	POS	NEG	NEG	NEG	NEG	POS	POS	POS	NEG
ST6-MRSA-IV, WA-51	NEG	NEG	NEG	NEG	NEG	NEG	AMB	POS	POS	POS	POS	POS	POS	NEG
ST6-MRSA-V	NEG	NEG	NEG	NEG	NEG	NEG	POS	POS	POS	POS	POS	POS	POS	NEG
CC7-MRSA-IV	NEG	NEG	NEG	NEG	NEG	NEG	POS	POS	POS	POS	POS	POS	POS	NEG
CC7-MRSA-V	NEG	NEG	NEG	NEG	NEG	NEG	POS	POS	POS	POS	POS	POS	POS	NEG
ST250-MRSA-I, Early/Ancestral MRSA	NEG	NEG	NEG	POS	POS	POS	NEG	NEG	NEG	NEG	POS	POS	POS	NEG
ST247-MRSA-I, North German/Iberian MRSA	NEG	NEG	NEG	COMM	COMM	COMM	NEG	NEG	NEG	NEG	POS	POS	POS	NEG
CC8-MRSA-IV, UK-14/WA-5	NEG	NEG	NEG	POS	POS	POS	NEG	NEG	NEG	NEG	POS	POS	POS	NEG
CC8-MRSA-IV, Lyon Clone/UK-2	NEG	NEG	NEG	POS	POS	POS	NEG	NEG	NEG	NEG	POS	POS	POS	NEG
CC8-MRSA-IV, UK-6	NEG	NEG	NEG	POS	POS	POS	NEG	NEG	NEG	NEG	POS	POS	POS	NEG
CC8-MRSA-IV, USA500	NEG	NEG	NEG	POS	POS	POS	NEG	NEG	NEG	NEG	POS	POS	POS	NEG
ST8-MRSA-IV [PVL/ACME+], USA300	NEG	NEG	NEG	POS	POS	POS	NEG	NEG	NEG	NEG	POS	POS	POS	NEG
ST8-MRSA-IV [PVL+], USA300	NEG	NEG	NEG	POS	POS	POS	NEG	NEG	NEG	NEG	POS	POS	POS	NEG
CC8-MRSA-IV [PVL/sed/j/r/k/q+]	NEG	NEG	NEG	POS	POS	POS	NEG	NEG	NEG	NEG	POS	POS	POS	NEG
CC8-MRSA-IV [tst1/sec/sel+]	NEG	NEG	NEG	POS	POS	POS	NEG	NEG	NEG	NEG	POS	POS	POS	NEG
CC8-MRSA-V	NEG	NEG	NEG	POS	POS	POS	NEG	NEG	NEG	NEG	POS	POS	POS	NEG
CC8-MRSA-VIII	NEG	NEG	NEG	POS	POS	POS	NEG	NEG	NEG	NEG	AMB	POS	POS	NEG
ST8-MRSA-IIA/B/C/D/E, Irish AR13/14	NEG	NEG	NEG	POS	POS	POS	NEG	NEG	NEG	NEG	POS	POS	POS	NEG
ST8-MRSA, UK-12/13, Irish AR43	NEG	NEG	NEG	POS	POS	POS	NEG	NEG	NEG	NEG	POS	POS	POS	NEG
ST254-MRSA, Hannover EMRSA	NEG	NEG	NEG	POS	POS	POS	NEG	NEG	NEG	NEG	POS	POS	POS	NEG
ST254-MRSA-IV/V, Hannover/UK-10	NEG	NEG	NEG	POS	POS	POS	NEG	NEG	NEG	NEG	POS	POS	POS	NEG
CC9-MRSA-IV	NEG	NEG	NEG	POS	POS	POS	NEG	NEG	NEG	NEG	POS	POS	POS	NEG
CC12-MRSA, WA-59	NEG	NEG	NEG	NEG	NEG	NEG	POS	POS	POS	POS	POS	POS	POS	NEG
CC12-MRSA-IV	NEG	NEG	NEG	NEG	NEG	NEG	POS	POS	POS	POS	POS	POS	POS	NEG
CC20-MRSA-V	NEG	NEG	NEG	POS	POS	POS	NEG	NEG	NEG	NEG	POS	POS	POS	NEG
ST22-MRSA-IV, Barnim/UK-15	NEG	NEG	NEG	POS	POS	POS	NEG	NEG	NEG	NEG	POS	POS	POS	NEG
ST22-MRSA-IV [ACME+]	NEG	NEG	NEG	POS	POS	POS	NEG	NEG	NEG	NEG	POS	POS	POS	NEG
ST22-MRSA-IV [PVL+]	NEG	NEG	NEG	POS	POS	POS	NEG	NEG	NEG	NEG	POS	POS	POS	NEG
ST22-MRSA-V	NEG	NEG	NEG	POS	POS	POS	NEG	NEG	NEG	NEG	POS	POS	POS	NEG
ST22-MRSA-V [PVL+]	NEG	NEG	NEG	POS	POS	POS	NEG	NEG	NEG	NEG	POS	POS	POS	NEG
ST36-MRSA-II, UK-16	NEG	NEG	NEG	NEG	NEG	NEG	POS	POS	POS	POS	POS	POS	POS	NEG
ST30-MRSA-IV [PVL+], Southwest Pacific Clone	NEG	NEG	NEG	NEG	NEG	NEG	POS	POS	POS	POS	POS	POS	POS	NEG
ST30-MRSA-IV [tst1+]	NEG	NEG	NEG	NEG	NEG	NEG	AMB	POS	POS	POS	POS	POS	POS	NEG
CC30-MRSA-V [PVL+]	NEG	NEG	NEG	NEG	NEG	NEG	POS	POS	POS	POS	POS	POS	POS	NEG
ST45-MRSA-II, USA600	NEG	NEG	NEG	NEG	NEG	NEG	POS	POS	POS	POS	POS	POS	POS	NEG
ST45-MRSA-IV, Berlin EMRSA	NEG	NEG	NEG	NEG	NEG	NEG	POS	POS	POS	POS	POS	POS	POS	NEG
CC45-MRSA-IV [ACME+]	NEG	NEG	NEG	NEG	NEG	NEG	POS	POS	POS	POS	POS	POS	POS	NEG
ST45-MRSA-V, WA-4	NEG	NEG	NEG	NEG	NEG	NEG	POS	POS	POS	POS	POS	POS	POS	NEG
CC45/agr IV-MRSA-IV, WA-23	NEG	NEG	NEG	NEG	NEG	NEG	POS	POS	POS	POS	POS	POS	POS	NEG
CC45/agr IV-MRSA-V	NEG	NEG	NEG	NEG	NEG	NEG	POS	POS	POS	POS	POS	POS	POS	NEG
CC45/agr IV-MRSA-IV/V	NEG	NEG	NEG	NEG	NEG	NEG	POS	POS	POS	POS	POS	POS	POS	NEG
CC59-MRSA-IV [PVL+], USA1000	NEG	NEG	NEG	NEG	NEG	NEG	POS	POS	POS	POS	POS	POS	POS	NEG
ST59-MRSA-IV, WA-73	NEG	NEG	NEG	NEG	NEG	NEG	POS	POS	POS	POS	POS	POS	POS	NEG
ST87-MRSA-IV, WA-24	NEG	NEG	NEG	NEG	NEG	NEG	POS	POS	POS	POS	POS	POS	POS	NEG
ST59-MRSA-IV [PVL+], WA-55/56	NEG	NEG	NEG	NEG	NEG	NEG	POS	POS	POS	POS	POS	POS	POS	NEG
ST59/ST952-MRSA-V(T) [PVL+], Taiwan Clone	NEG	NEG	NEG	NEG	NEG	NEG	POS	POS	POS	POS	POS	POS	POS	NEG
ST59-MRSA-V	NEG	NEG	NEG	NEG	NEG	NEG	POS	POS	POS	POS	POS	POS	POS	NEG
CC59-MRSA-V [PVL+]	NEG	NEG	NEG	NEG	NEG	NEG	POS	POS	POS	POS	POS	POS	POS	NEG
ST59-MRSA-IV/V, WA-15	NEG	NEG	NEG	NEG	NEG	NEG	POS	POS	POS	POS	POS	POS	POS	NEG
ST72-MRSA-IV, USA700	NEG	NEG	NEG	POS	POS	POS	NEG	NEG	NEG	NEG	POS	POS	POS	NEG
ST72-MRSA-IV [PVL+], WA-44	NEG	AMB	NEG	POS	POS	POS	NEG	AMB	NEG	NEG	POS	POS	POS	NEG
CC75-MRSA-IV	NEG	NEG	NEG	NEG	NEG	NEG	NEG	NEG	NEG	NEG	AMB	NEG	NEG	NEG
ST883-MRSA-IV, WA-47	NEG	NEG	NEG	NEG	NEG	NEG	NEG	NEG	NEG	NEG	POS	NEG	NEG	NEG
ST1303-MRSA-IV	NEG	NEG	NEG	NEG	NEG	NEG	NEG	NEG	NEG	NEG	POS	NEG	NEG	NEG
CC80-MRSA-IV	NEG	NEG	NEG	NEG	NEG	NEG	POS	POS	POS	POS	POS	POS	POS	NEG
CC80-MRSA-IV [PVL+], European Clone	NEG	NEG	NEG	NEG	NEG	NEG	POS	POS	POS	POS	POS	POS	POS	NEG
CC88-MRSA-IV, WA-2	NEG	NEG	NEG	NEG	NEG	NEG	POS	POS	POS	POS	POS	POS	POS	NEG
CC88-MRSA-IV [PVL+]	NEG	NEG	NEG	NEG	NEG	NEG	POS	POS	POS	POS	POS	POS	POS	NEG
CC88-MRSA-V [PVL+]	NEG	NEG	NEG	NEG	NEG	NEG	POS	POS	POS	POS	POS	POS	POS	NEG
CC88-MRSA-VI	NEG	NEG	NEG	NEG	NEG	NEG	POS	POS	POS	POS	POS	POS	POS	NEG
ST93-MRSA-IV [PVL-]	NEG	NEG	NEG	NEG	NEG	NEG	POS	POS	POS	POS	POS	POS	POS	NEG
ST93-MRSA-IV [PVL+], Queensland Clone	NEG	NEG	NEG	NEG	NEG	NEG	POS	POS	POS	POS	POS	POS	POS	NEG
ST93-MRSA-V [PVL+]	NEG	NEG	NEG	NEG	NEG	NEG	POS	POS	POS	POS	POS	POS	POS	NEG
CC97-MRSA-IV, WA-54	NEG	NEG	NEG	POS	POS	POS	NEG	NEG	NEG	NEG	POS	POS	POS	NEG
CC97-MRSA-V	NEG	AMB	NEG	POS	POS	POS	NEG	AMB	NEG	NEG	POS	POS	POS	NEG
CC97-MRSA-V [ACME+]	NEG	NEG	NEG	POS	POS	POS	NEG	NEG	NEG	NEG	POS	POS	POS	NEG
CC97-MRSA-I/V	NEG	NEG	NEG	POS	POS	POS	NEG	NEG	NEG	NEG	POS	POS	POS	NEG
CC121-MRSA-V, WA-22	NEG	NEG	NEG	NEG	NEG	NEG	POS	POS	POS	POS	POS	POS	POS	NEG
CC152-MRSA-V [PVL+]	NEG	NEG	NEG	POS	POS	POS	NEG	NEG	NEG	NEG	POS	NEG	POS	NEG
ST154-MRSA [PVL+]	NEG	NEG	NEG	NEG	NEG	NEG	AMB	POS	POS	POS	POS	POS	POS	NEG
ST188-MRSA-IV, WA-38/78	NEG	NEG	NEG	NEG	NEG	NEG	POS	POS	POS	POS	POS	POS	POS	NEG
ST188-MRSA-V	NEG	NEG	NEG	NEG	NEG	NEG	POS	POS	POS	POS	POS	POS	POS	NEG
ST239-MRSA-III	NEG	NEG	NEG	NEG	NEG	NEG	POS	POS	POS	POS	POS	POS	POS	NEG
CC361-MRSA-IV, WA-29	NEG	NEG	NEG	NEG	NEG	NEG	POS	POS	POS	POS	POS	POS	POS	NEG
CC361-MRSA-V	NEG	NEG	NEG	NEG	NEG	NEG	POS	POS	POS	POS	POS	POS	POS	NEG
CC361-MRSA-VIII, WA-28	NEG	NEG	NEG	NEG	NEG	NEG	AMB	POS	POS	POS	POS	AMB	POS	NEG
CC398-MRSA-IV	NEG	NEG	NEG	POS	POS	POS	NEG	AMB	NEG	NEG	POS	POS	POS	NEG
ST398-MRSA-V	NEG	NEG	NEG	POS	POS	POS	NEG	NEG	NEG	NEG	POS	POS	POS	NEG
ST398-MRSA-V [PVL+]	NEG	NEG	NEG	POS	POS	POS	NEG	NEG	NEG	NEG	POS	POS	POS	NEG
CC509/ST207-MRSA-V	NEG	NEG	NEG	NEG	NEG	NEG	POS	POS	POS	POS	POS	POS	POS	NEG
ST573-MRSA-V, WA-10	NEG	NEG	NEG	POS	POS	POS	NEG	NEG	NEG	NEG	POS	POS	POS	NEG
ST772-MRSA-V [PVL+], WA-60/Bengal Bay Clone	NEG	NEG	NEG	POS	POS	POS	NEG	NEG	NEG	NEG	POS	POS	POS	NEG
ST779-MRSA	NEG	NEG	NEG	POS	POS	POS	NEG	NEG	NEG	NEG	POS	POS	POS	NEG
ST834-MRSA-IV, WA-13	NEG	NEG	NEG	NEG	NEG	NEG	POS	POS	POS	POS	POS	POS	POS	NEG
CC913-MRSA-IV	NEG	NEG	NEG	NEG	NEG	NEG	POS	POS	POS	POS	POS	POS	POS	NEG
ST1774-MRSA-IV [ACME+]	NEG	NEG	NEG	NEG	NEG	NEG	POS	POS	POS	POS	POS	POS	POS	NEG
CC15-MSSA	NEG	NEG	NEG	NEG	NEG	NEG	POS	POS	POS	POS	POS	POS	POS	NEG
ST426-MSSA	NEG	NEG	NEG	NEG	NEG	NEG	POS	POS	POS	POS	POS	POS	POS	NEG

Legend:

Positive :	POS
Ambiguous :	AMB
Negative :	NEG
Rare (in >0 to 33% of isolates) :	RARE
Variable (in >33 to 66% of isolates) :	VAR
Common (in >66 to <100% of isolates) :	COMM

	ADHAESION FACTORS / GENES ENCODING MICROBIAL SURFACE COMPONENTS RECOGNIZING ADHESIVE MATRIX MOLECULES (MSCRAMM GENES)															
	bbp (total)	bbp (cons)	bbp (COL+MW2)	bbp (MRSA252)	bbp (RF122)	bbp (ST45)	clfA (total)	clfA (cons)	clfA (COL+ RF122)	clfA (MRSA252)	clfA (Mu50+ MW2)	clfB (total)	clfB (cons)	clfB (COL+ Mu50)	clfB (MW2)	clfB (RF122)
CC1-MRSA-IV [PVL-], WA-1/57	POS	POS	POS	NEG	NEG	NEG	POS	POS	NEG	NEG	POS	POS	POS	NEG	POS	AMB
CC1-MRSA-IV [PVL+], USA400	POS	POS	POS	NEG	NEG	NEG	POS	POS	NEG	NEG	POS	POS	POS	NEG	POS	AMB
CC1-MRSA-IV/SCCfus [PVL-]	POS	POS	POS	NEG	NEG	NEG	POS	POS	NEG	NEG	POS	POS	POS	NEG	POS	AMB
CC1-MRSA-IV/SCCfus [PVL+]	POS	POS	POS	NEG	NEG	NEG	POS	POS	NEG	NEG	POS	POS	POS	NEG	POS	AMB
CC1-MRSA-V [PVL+]	POS	POS	POS	NEG	NEG	NEG	POS	POS	NEG	NEG	POS	POS	POS	NEG	AMB	POS
CC1-MRSA-V/SCCfus [PVL-]	POS	POS	POS	NEG	NEG	NEG	POS	POS	NEG	NEG	POS	POS	POS	NEG	AMB	POS
CC1-MRSA-V/SCCfus [PVL+]	POS	POS	POS	NEG	NEG	NEG	POS	POS	NEG	NEG	POS	POS	POS	NEG	AMB	POS
ST228-MRSA-I, South German/Italian MRSA	COMM	COMM	NEG	NEG	NEG	NEG	COMM	COMM	NEG	NEG	COMM	POS	POS	POS	NEG	NEG
ST5-MRSA-I, Geraldine Clone	COMM	COMM	NEG	NEG	NEG	NEG	POS	POS	NEG	NEG	POS	POS	POS	POS	NEG	NEG
CC5-MRSA-Ivar., WA-18/21/48	POS	POS	NEG	NEG	NEG	NEG	POS	POS	NEG	NEG	POS	POS	POS	POS	NEG	NEG
CC5-MRSA-II, Rhine-Hesse /UK-3	POS	POS	NEG	NEG	NEG	NEG	POS	POS	NEG	NEG	POS	POS	POS	POS	NEG	NEG
CC5-MRSA-II [ACME+]	POS	POS	NEG	NEG	NEG	NEG	POS	POS	NEG	NEG	POS	POS	POS	POS	NEG	NEG
CC5-MRSA-III	POS	POS	NEG	NEG	NEG	NEG	POS	POS	NEG	NEG	POS	POS	POS	POS	NEG	NEG
CC5-MRSA-IV, Paediatric clone	COMM	COMM	NEG	NEG	NEG	NEG	POS	POS	NEG	NEG	POS	POS	POS	POS	NEG	NEG
CC5-MRSA-IV [PVL+]	COMM	COMM	NEG	NEG	NEG	NEG	POS	POS	NEG	NEG	POS	POS	POS	POS	NEG	NEG
CC5-MRSA-V	POS	POS	NEG	NEG	NEG	NEG	POS	POS	NEG	NEG	POS	POS	POS	POS	NEG	NEG
CC5-MRSA-V [PVL+]	POS	POS	NEG	NEG	NEG	NEG	POS	POS	NEG	NEG	POS	POS	POS	POS	NEG	NEG
CC5-MRSA-VI, New Paediatric Clone	POS	POS	NEG	NEG	NEG	NEG	POS	POS	NEG	NEG	POS	POS	POS	POS	NEG	NEG
ST5-MRSA-VII (SCC-JCSC6082)	NEG	NEG	NEG	NEG	NEG	NEG	POS	POS	NEG	NEG	POS	POS	POS	POS	NEG	NEG
CC5-MRSA-IVvar, Maltese Clone	COMM	COMM	NEG	NEG	NEG	NEG	POS	POS	NEG	NEG	POS	POS	POS	POS	NEG	NEG
CC5-MRSA-SCC(MRSAZH47)	POS	POS	NEG	NEG	NEG	NEG	POS	POS	NEG	NEG	POS	POS	AMB	POS	NEG	NEG
CC5-MRSA-IV/VI	COMM	COMM	NEG	NEG	NEG	NEG	POS	POS	NEG	NEG	POS	POS	POS	POS	NEG	NEG
CC5-MRSA-IV/SCCfus	POS	POS	NEG	NEG	NEG	NEG	POS	POS	NEG	NEG	POS	POS	POS	POS	NEG	NEG
CC5/ST835-MRSA, WA-40/46	POS	POS	NEG	NEG	NEG	NEG	POS	POS	NEG	NEG	POS	POS	POS	POS	NEG	NEG
ST6-MRSA-IV, WA-51	COMM	COMM	COMM	NEG	NEG	NEG	POS	POS	NEG	NEG	POS	POS	POS	NEG	NEG	AMB
ST6-MRSA-V	POS	POS	POS	NEG	NEG	AMB	POS	POS	NEG	NEG	POS	POS	POS	NEG	NEG	POS
CC7-MRSA-IV	POS	POS	POS	AMB	NEG	NEG	POS	POS	POS	AMB	AMB	POS	POS	NEG	NEG	AMB
CC7-MRSA-V	POS	POS	POS	AMB	NEG	NEG	POS	POS	POS	AMB	AMB	POS	POS	NEG	NEG	AMB
ST250-MRSA-I, Early/Ancestral MRSA	POS	POS	POS	NEG	NEG	NEG	POS	POS	POS	AMB	AMB	POS	POS	POS	NEG	NEG
ST247-MRSA-I, North German/Iberian MRSA	RARE	RARE	RARE	NEG	NEG	NEG	COMM	COMM	COMM	AMB	AMB	POS	POS	POS	NEG	NEG
CC8-MRSA-IV, UK-14/WA-5	COMM	COMM	COMM	NEG	NEG	NEG	COMM	COMM	VAR	AMB	AMB	POS	POS	POS	NEG	NEG
CC8-MRSA-IV, Lyon Clone/UK-2	COMM	COMM	COMM	NEG	NEG	NEG	POS	POS	POS	AMB	AMB	POS	POS	POS	NEG	NEG
CC8-MRSA-IV, UK-6	POS	POS	POS	NEG	NEG	NEG	POS	POS	POS	AMB	AMB	POS	POS	POS	NEG	NEG
CC8-MRSA-IV, USA500	POS	POS	POS	NEG	NEG	NEG	POS	POS	POS	AMB	AMB	POS	POS	POS	NEG	NEG
ST8-MRSA-IV [PVL/ACME+], USA300	POS	POS	POS	NEG	NEG	NEG	POS	POS	POS	AMB	AMB	POS	POS	POS	NEG	NEG
ST8-MRSA-IV [PVL+], USA300	COMM	COMM	COMM	NEG	NEG	NEG	POS	POS	POS	NEG	AMB	POS	POS	POS	NEG	NEG
CC8-MRSA-IV [PVL/sed/j/r/k/q+]	POS	POS	POS	NEG	NEG	NEG	POS	POS	AMB	AMB	POS	POS	POS	POS	NEG	NEG
CC8-MRSA-IV [tst1/sec/sel+]	POS	POS	POS	NEG	AMB	NEG	POS	POS	AMB	AMB	AMB	POS	POS	POS	NEG	NEG
CC8-MRSA-V	POS	POS	POS	NEG	NEG	NEG	POS	POS	POS	AMB	AMB	POS	POS	POS	NEG	NEG
CC8-MRSA-VIII	POS	POS	POS	NEG	NEG	NEG	POS	POS	POS	NEG	AMB	POS	POS	POS	NEG	NEG
ST8-MRSA-IIA/B/C/D/E, Irish AR13/14	POS	POS	POS	NEG	NEG	NEG	COMM	COMM	COMM	RARE	RARE	POS	POS	POS	NEG	NEG
ST8-MRSA, UK-12/13, Irish AR43	POS	POS	POS	NEG	NEG	NEG	POS	POS	POS	AMB	AMB	POS	POS	POS	NEG	NEG
ST254-MRSA, Hannover EMRSA	NEG	NEG	NEG	NEG	NEG	NEG	POS	POS	POS	POS	POS	POS	POS	POS	NEG	NEG
ST254-MRSA-IV/V, Hannover/UK-10	NEG	NEG	NEG	NEG	NEG	NEG	POS	POS	POS	AMB	POS	POS	POS	POS	NEG	NEG
CC9-MRSA-IV	POS	POS	NEG	NEG	NEG	NEG	POS	POS	NEG	NEG	POS	POS	POS	NEG	NEG	POS
CC12-MRSA, WA-59	NEG	NEG	NEG	NEG	NEG	NEG	POS	POS	POS	NEG	POS	POS	POS	NEG	NEG	NEG
CC12-MRSA-IV	NEG	VAR	NEG	NEG	NEG	NEG	POS	POS	POS	AMB	AMB	POS	POS	NEG	NEG	AMB
CC20-MRSA-V	POS	POS	NEG	NEG	NEG	NEG	POS	POS	NEG	NEG	POS	POS	POS	NEG	NEG	AMB
ST22-MRSA-IV, Barnim/UK-15	POS	POS	NEG	NEG	NEG	NEG	POS	POS	NEG	NEG	POS	POS	POS	NEG	NEG	AMB
ST22-MRSA-IV [ACME+]	AMB	NEG	NEG	NEG	NEG	AMB	POS	POS	NEG	NEG	POS	POS	POS	NEG	NEG	POS
ST22-MRSA-IV [PVL+]	POS	POS	NEG	NEG	NEG	NEG	POS	POS	NEG	NEG	POS	POS	POS	NEG	NEG	AMB
ST22-MRSA-V	POS	POS	NEG	NEG	NEG	NEG	POS	POS	NEG	NEG	POS	POS	POS	NEG	NEG	AMB
ST22-MRSA-V [PVL+]	POS	POS	NEG	NEG	NEG	NEG	POS	POS	NEG	NEG	POS	POS	POS	NEG	NEG	AMB
ST36-MRSA-II, UK-16	POS	POS	NEG	POS	NEG	NEG	POS	POS	AMB	POS	NEG	POS	POS	NEG	NEG	AMB
ST30-MRSA-IV [PVL+], Southwest Pacific Clone	POS	POS	NEG	POS	NEG	NEG	POS	POS	AMB	POS	NEG	POS	POS	NEG	NEG	AMB
ST30-MRSA-IV [tst1+]	POS	POS	NEG	POS	NEG	NEG	POS	POS	AMB	POS	NEG	POS	POS	NEG	NEG	AMB
CC30-MRSA-V [PVL+]	POS	POS	NEG	POS	NEG	NEG	POS	POS	AMB	POS	NEG	POS	POS	NEG	NEG	POS
ST45-MRSA-II, USA600	POS	POS	NEG	NEG	NEG	POS	POS	POS	NEG	NEG	POS	POS	POS	NEG	POS	AMB
ST45-MRSA-IV, Berlin EMRSA	POS	POS	NEG	NEG	NEG	POS	POS	POS	AMB	NEG	POS	POS	POS	NEG	POS	AMB
CC45-MRSA-IV [ACME+]	POS	POS	AMB	NEG	NEG	POS	POS	POS	AMB	NEG	POS	POS	POS	NEG	POS	AMB
ST45-MRSA-V, WA-4	POS	POS	NEG	NEG	NEG	POS	POS	POS	AMB	NEG	POS	POS	POS	NEG	POS	AMB
CC45/agr IV-MRSA-IV, WA-23	COMM	COMM	NEG	NEG	NEG	COMM	POS	POS	NEG	NEG	POS	POS	POS	NEG	POS	AMB
CC45/agr IV-MRSA-V	POS	POS	NEG	NEG	NEG	POS	POS	POS	AMB	NEG	POS	POS	POS	NEG	POS	AMB
CC45/agr IV-MRSA-IV/V	POS	POS	NEG	NEG	NEG	POS	POS	POS	AMB	NEG	POS	POS	POS	NEG	POS	AMB
CC59-MRSA-IV [PVL+], USA1000	POS	POS	NEG	NEG	NEG	NEG	POS	POS	POS	AMB	AMB	POS	POS	NEG	AMB	POS
ST59-MRSA-IV, WA-73	POS	POS	AMB	NEG	NEG	NEG	POS	POS	POS	AMB	AMB	POS	POS	NEG	AMB	POS
ST87-MRSA-IV, WA-24	POS	POS	NEG	NEG	NEG	NEG	POS	POS	POS	AMB	AMB	POS	POS	NEG	AMB	POS
ST59-MRSA-IV [PVL+], WA-55/56	POS	POS	AMB	NEG	NEG	NEG	POS	POS	POS	AMB	AMB	POS	POS	NEG	AMB	AMB
ST59/ST952-MRSA-V(T) [PVL+], Taiwan Clone	POS	POS	AMB	NEG	NEG	NEG	POS	POS	POS	AMB	AMB	POS	POS	NEG	POS	POS
ST59-MRSA-V	POS	POS	NEG	NEG	NEG	NEG	POS	POS	POS	AMB	AMB	POS	POS	NEG	AMB	POS
CC59-MRSA-V [PVL+]	POS	POS	NEG	NEG	NEG	NEG	POS	POS	POS	NEG	POS	POS	POS	NEG	AMB	POS
ST59-MRSA-IV/V, WA-15	POS	POS	NEG	NEG	NEG	NEG	POS	POS	POS	AMB	AMB	POS	POS	NEG	AMB	POS
ST72-MRSA-IV, USA700	POS	POS	NEG	NEG	NEG	NEG	POS	POS	POS	AMB	AMB	POS	POS	NEG	NEG	AMB
ST72-MRSA-IV [PVL+], WA-44	POS	POS	NEG	NEG	NEG	NEG	POS	POS	POS	AMB	AMB	POS	POS	NEG	NEG	AMB
CC75-MRSA-IV	POS	POS	NEG	AMB	NEG	NEG	AMB	NEG	NEG	AMB	NEG	POS	AMB	POS	NEG	NEG
ST883-MRSA-IV, WA-47	POS	POS	NEG	POS	NEG	AMB	AMB	NEG	NEG	POS	NEG	POS	POS	POS	NEG	NEG
ST1303-MRSA-IV	POS	POS	NEG	NEG	NEG	NEG	POS	NEG	NEG	POS	NEG	POS	NEG	POS	NEG	NEG
CC80-MRSA-IV	POS	POS	NEG	NEG	NEG	NEG	POS	POS	NEG	NEG	POS	POS	POS	POS	NEG	NEG
CC80-MRSA-IV [PVL+], European Clone	POS	POS	NEG	NEG	NEG	NEG	POS	POS	NEG	NEG	POS	POS	POS	POS	NEG	NEG
CC88-MRSA-IV, WA-2	POS	POS	NEG	NEG	NEG	NEG	POS	POS	NEG	NEG	POS	POS	POS	NEG	NEG	AMB
CC88-MRSA-IV [PVL+]	POS	POS	NEG	NEG	NEG	NEG	POS	POS	NEG	NEG	POS	POS	POS	NEG	NEG	AMB
CC88-MRSA-V [PVL+]	POS	POS	NEG	NEG	NEG	NEG	POS	POS	NEG	NEG	POS	POS	POS	NEG	NEG	POS
CC88-MRSA-VI	POS	POS	NEG	NEG	NEG	NEG	POS	POS	NEG	NEG	POS	POS	POS	NEG	NEG	POS
ST93-MRSA-IV [PVL-]	AMB	AMB	NEG	NEG	NEG	NEG	POS	POS	NEG	NEG	POS	POS	POS	NEG	NEG	POS
ST93-MRSA-IV [PVL+], Queensland Clone	POS	POS	NEG	NEG	NEG	NEG	POS	POS	NEG	NEG	POS	POS	POS	NEG	NEG	POS
ST93-MRSA-V [PVL+]	POS	POS	NEG	NEG	NEG	NEG	POS	POS	NEG	NEG	POS	POS	POS	NEG	NEG	POS
CC97-MRSA-IV, WA-54	POS	POS	NEG	NEG	NEG	NEG	POS	POS	POS	NEG	AMB	AMB	POS	NEG	NEG	NEG
CC97-MRSA-V	POS	POS	NEG	NEG	NEG	NEG	POS	POS	POS	AMB	AMB	POS	POS	NEG	NEG	POS
CC97-MRSA-V [ACME+]	POS	POS	AMB	NEG	NEG	AMB	POS	POS	POS	AMB	AMB	POS	POS	NEG	NEG	POS
CC97-MRSA-I/V	POS	POS	NEG	NEG	NEG	NEG	POS	POS	POS	AMB	AMB	POS	POS	NEG	NEG	AMB
CC121-MRSA-V, WA-22	POS	POS	NEG	POS	NEG	NEG	POS	POS	NEG	NEG	POS	POS	POS	NEG	NEG	POS
CC152-MRSA-V [PVL+]	POS	POS	POS	NEG	NEG	NEG	POS	POS	POS	AMB	AMB	POS	POS	NEG	POS	POS
ST154-MRSA [PVL+]	POS	POS	NEG	NEG	NEG	NEG	POS	POS	NEG	NEG	POS	POS	POS	POS	NEG	NEG
ST188-MRSA-IV, WA-38/78	POS	POS	POS	NEG	NEG	NEG	POS	POS	NEG	NEG	POS	POS	POS	NEG	NEG	POS
ST188-MRSA-V	POS	POS	POS	NEG	NEG	AMB	POS	POS	NEG	NEG	POS	POS	POS	NEG	NEG	POS
ST239-MRSA-III	COMM	COMM	COMM	NEG	NEG	NEG	POS	POS	POS	AMB	AMB	POS	POS	NEG	NEG	AMB
CC361-MRSA-IV, WA-29	COMM	COMM	COMM	NEG	NEG	NEG	POS	POS	NEG	NEG	POS	POS	POS	POS	NEG	NEG
CC361-MRSA-V	POS	POS	POS	NEG	NEG	AMB	POS	POS	NEG	NEG	POS	POS	POS	POS	NEG	NEG
CC361-MRSA-VIII, WA-28	POS	POS	POS	NEG	NEG	NEG	POS	POS	NEG	NEG	POS	POS	POS	POS	NEG	NEG
CC398-MRSA-IV	POS	POS	NEG	NEG	NEG	POS	POS	POS	POS	AMB	AMB	POS	POS	NEG	POS	AMB
ST398-MRSA-V	POS	POS	NEG	NEG	NEG	POS	POS	POS	POS	AMB	AMB	POS	POS	NEG	POS	AMB
ST398-MRSA-V [PVL+]	POS	POS	NEG	NEG	NEG	POS	POS	POS	POS	NEG	AMB	POS	POS	NEG	AMB	POS
CC509/ST207-MRSA-V	POS	POS	POS	NEG	NEG	NEG	POS	POS	NEG	NEG	POS	POS	POS	NEG	NEG	NEG
ST573-MRSA-V, WA-10	POS	POS	NEG	NEG	NEG	NEG	POS	POS	NEG	NEG	POS	POS	POS	NEG	NEG	NEG
ST772-MRSA-V [PVL+], WA-60/Bengal Bay Clone	POS	POS	NEG	NEG	NEG	NEG	POS	POS	NEG	NEG	POS	POS	POS	NEG	NEG	AMB
ST779-MRSA	POS	POS	NEG	NEG	NEG	NEG	POS	POS	NEG	NEG	POS	AMB	AMB	NEG	NEG	AMB
ST834-MRSA-IV, WA-13	POS	POS	POS	NEG	NEG	AMB	POS	POS	POS	AMB	POS	AMB	AMB	NEG	NEG	AMB
CC913-MRSA-IV	NEG	NEG	NEG	NEG	NEG	NEG	POS	POS	NEG	NEG	POS	POS	POS	NEG	POS	POS
ST1774-MRSA-IV [ACME+]	POS	POS	POS	NEG	NEG	AMB	POS	POS	NEG	NEG	POS	POS	POS	NEG	POS	AMB
CC15-MSSA	POS	POS	NEG	NEG	NEG	NEG	POS	POS	NEG	NEG	POS	POS	POS	NEG	POS	AMB
ST426-MSSA	POS	COMM	NEG	NEG	NEG	COMM	POS	POS	AMB	NEG	POS	POS	POS	NEG	POS	AMB

Legend:

Positive :	POS
Ambiguous :	AMB
Negative :	NEG
Rare (in >0 to 33% of isolates) :	RARE
Variable (in >33 to 66% of isolates) :	VAR
Common (in >66 to <100% of isolates) :	COMM

	ADHAESION FACTORS / GENES ENCODING MICROBIAL SURFACE COMPONENTS RECOGNIZING ADHESIVE MATRIX MOLECULES (MSCRAMM GENES), continued															
	cna	ebh (cons)	ebpS (total)	ebpS (probe 612)	ebpS (probe 614)	ebpS (01-1111)	ebpS (COL)	eno	fib	fib (MRSA252)	fnbA (total)	fnbA (cons)	fnbA (COL)	fnbA (MRSA252)	fnbA (Mu50+ MW2)	fnbA (RF122)
CC1-MRSA-IV [PVL-], WA-1/57	POS	POS	POS	POS	POS	NEG	NEG	POS	POS	NEG	POS	POS	NEG	NEG	POS	NEG
CC1-MRSA-IV [PVL+], USA400	POS	POS	POS	POS	POS	NEG	NEG	POS	POS	NEG	POS	POS	NEG	NEG	POS	NEG
CC1-MRSA-IV/SCCfus [PVL-]	POS	POS	POS	POS	POS	NEG	NEG	POS	POS	NEG	POS	POS	NEG	NEG	POS	NEG
CC1-MRSA-IV/SCCfus [PVL+]	POS	POS	POS	POS	POS	NEG	NEG	POS	POS	NEG	POS	POS	NEG	NEG	POS	NEG
CC1-MRSA-V [PVL+]	POS	POS	POS	POS	POS	NEG	NEG	POS	POS	NEG	POS	POS	NEG	NEG	POS	NEG
CC1-MRSA-V/SCCfus [PVL-]	POS	POS	POS	POS	POS	NEG	NEG	POS	POS	NEG	POS	POS	NEG	NEG	POS	NEG
CC1-MRSA-V/SCCfus [PVL+]	POS	POS	POS	POS	POS	NEG	NEG	POS	POS	NEG	POS	POS	NEG	NEG	POS	NEG
ST228-MRSA-I, South German/Italian MRSA	NEG	POS	POS	POS	POS	NEG	NEG	POS	POS	NEG	POS	POS	NEG	NEG	POS	NEG
ST5-MRSA-I, Geraldine Clone	NEG	POS	POS	POS	POS	NEG	NEG	POS	POS	NEG	POS	POS	NEG	NEG	POS	NEG
CC5-MRSA-Ivar., WA-18/21/48	NEG	POS	POS	POS	POS	NEG	NEG	POS	POS	NEG	POS	POS	NEG	NEG	POS	NEG
CC5-MRSA-II, Rhine-Hesse /UK-3	NEG	POS	POS	POS	POS	NEG	NEG	POS	POS	NEG	POS	POS	NEG	NEG	POS	NEG
CC5-MRSA-II [ACME+]	NEG	POS	POS	POS	POS	NEG	NEG	POS	POS	NEG	POS	POS	NEG	NEG	POS	NEG
CC5-MRSA-III	NEG	POS	POS	POS	POS	NEG	NEG	POS	POS	NEG	POS	POS	NEG	NEG	POS	NEG
CC5-MRSA-IV, Paediatric clone	NEG	POS	POS	POS	POS	NEG	NEG	POS	POS	NEG	POS	POS	NEG	NEG	POS	NEG
CC5-MRSA-IV [PVL+]	NEG	POS	POS	POS	POS	NEG	NEG	POS	POS	NEG	POS	POS	NEG	NEG	POS	NEG
CC5-MRSA-V	NEG	POS	POS	POS	POS	NEG	NEG	POS	POS	NEG	POS	POS	NEG	NEG	POS	NEG
CC5-MRSA-V [PVL+]	NEG	POS	POS	AMB	POS	NEG	NEG	POS	POS	NEG	POS	POS	NEG	NEG	POS	NEG
CC5-MRSA-VI, New Paediatric Clone	NEG	POS	POS	POS	POS	NEG	NEG	POS	POS	NEG	POS	POS	NEG	NEG	POS	NEG
ST5-MRSA-VII (SCC-JCSC6082)	NEG	POS	POS	POS	POS	NEG	NEG	POS	POS	NEG	POS	POS	NEG	NEG	POS	NEG
CC5-MRSA-IVvar, Maltese Clone	NEG	POS	POS	POS	POS	NEG	NEG	POS	POS	NEG	POS	POS	NEG	NEG	POS	NEG
CC5-MRSA-SCC(MRSAZH47)	NEG	POS	POS	POS	POS	NEG	NEG	POS	POS	NEG	POS	POS	NEG	NEG	POS	NEG
CC5-MRSA-IV/VI	NEG	POS	POS	POS	POS	NEG	NEG	POS	POS	NEG	POS	COMM	NEG	NEG	COMM	NEG
CC5-MRSA-IV/SCCfus	NEG	POS	POS	POS	POS	NEG	NEG	POS	POS	NEG	POS	POS	NEG	NEG	POS	NEG
CC5/ST835-MRSA, WA-40/46	NEG	POS	POS	POS	POS	NEG	NEG	POS	POS	NEG	POS	POS	NEG	NEG	POS	NEG
ST6-MRSA-IV, WA-51	POS	POS	POS	AMB	POS	NEG	NEG	POS	POS	NEG	POS	POS	NEG	POS	NEG	NEG
ST6-MRSA-V	POS	POS	POS	POS	POS	NEG	NEG	POS	POS	NEG	POS	POS	NEG	POS	NEG	AMB
CC7-MRSA-IV	NEG	POS	POS	POS	POS	NEG	POS	POS	POS	NEG	POS	POS	NEG	NEG	NEG	NEG
CC7-MRSA-V	NEG	POS	POS	POS	POS	NEG	POS	POS	POS	NEG	POS	POS	NEG	NEG	NEG	NEG
ST250-MRSA-I, Early/Ancestral MRSA	NEG	POS	POS	POS	POS	NEG	POS	POS	POS	NEG	POS	POS	POS	NEG	NEG	NEG
ST247-MRSA-I, North German/Iberian MRSA	NEG	POS	POS	POS	POS	NEG	POS	POS	POS	NEG	POS	POS	POS	NEG	NEG	NEG
CC8-MRSA-IV, UK-14/WA-5	NEG	POS	POS	POS	POS	NEG	POS	POS	POS	NEG	POS	POS	POS	NEG	NEG	NEG
CC8-MRSA-IV, Lyon Clone/UK-2	NEG	POS	POS	POS	POS	NEG	POS	POS	POS	NEG	POS	POS	POS	NEG	NEG	NEG
CC8-MRSA-IV, UK-6	NEG	POS	POS	POS	POS	NEG	POS	POS	POS	NEG	POS	POS	POS	NEG	NEG	NEG
CC8-MRSA-IV, USA500	NEG	POS	POS	POS	POS	NEG	POS	POS	POS	NEG	POS	POS	POS	NEG	NEG	NEG
ST8-MRSA-IV [PVL/ACME+], USA300	NEG	POS	POS	POS	POS	NEG	POS	POS	POS	NEG	POS	POS	POS	NEG	NEG	NEG
ST8-MRSA-IV [PVL+], USA300	NEG	POS	POS	POS	POS	NEG	POS	POS	POS	NEG	POS	POS	POS	NEG	NEG	NEG
CC8-MRSA-IV [PVL/sed/j/r/k/q+]	NEG	POS	POS	POS	POS	NEG	POS	POS	POS	NEG	POS	POS	POS	NEG	NEG	NEG
CC8-MRSA-IV [tst1/sec/sel+]	NEG	POS	POS	POS	POS	NEG	POS	POS	POS	NEG	POS	POS	POS	NEG	NEG	NEG
CC8-MRSA-V	NEG	POS	POS	POS	POS	NEG	POS	POS	POS	NEG	POS	POS	POS	NEG	NEG	NEG
CC8-MRSA-VIII	NEG	POS	POS	POS	POS	NEG	AMB	POS	POS	NEG	POS	POS	POS	NEG	NEG	NEG
ST8-MRSA-IIA/B/C/D/E, Irish AR13/14	NEG	POS	POS	POS	POS	NEG	POS	POS	POS	NEG	POS	POS	POS	NEG	NEG	NEG
ST8-MRSA, UK-12/13, Irish AR43	NEG	POS	POS	POS	POS	NEG	POS	POS	POS	NEG	POS	POS	POS	NEG	NEG	NEG
ST254-MRSA, Hannover EMRSA	NEG	POS	POS	POS	POS	NEG	POS	POS	POS	NEG	POS	POS	POS	NEG	NEG	NEG
ST254-MRSA-IV/V, Hannover/UK-10	NEG	POS	POS	POS	POS	NEG	POS	POS	POS	NEG	POS	POS	POS	NEG	NEG	NEG
CC9-MRSA-IV	NEG	POS	POS	POS	POS	NEG	NEG	POS	POS	NEG	POS	POS	NEG	POS	NEG	NEG
CC12-MRSA, WA-59	POS	POS	POS	POS	POS	NEG	NEG	POS	POS	NEG	POS	POS	NEG	NEG	NEG	NEG
CC12-MRSA-IV	POS	POS	POS	POS	POS	NEG	NEG	POS	POS	NEG	POS	POS	NEG	NEG	NEG	NEG
CC20-MRSA-V	NEG	POS	POS	POS	POS	NEG	POS	POS	POS	NEG	POS	POS	NEG	POS	NEG	NEG
ST22-MRSA-IV, Barnim/UK-15	POS	NEG	POS	POS	POS	NEG	POS	POS	NEG	POS	POS	POS	NEG	NEG	POS	NEG
ST22-MRSA-IV [ACME+]	POS	NEG	POS	POS	POS	NEG	POS	POS	NEG	POS	POS	POS	NEG	NEG	POS	NEG
ST22-MRSA-IV [PVL+]	POS	NEG	POS	POS	POS	NEG	POS	POS	NEG	POS	POS	POS	NEG	NEG	POS	NEG
ST22-MRSA-V	POS	NEG	POS	POS	POS	NEG	POS	POS	NEG	POS	POS	POS	NEG	NEG	POS	NEG
ST22-MRSA-V [PVL+]	POS	NEG	POS	POS	POS	NEG	POS	POS	NEG	POS	POS	POS	NEG	NEG	POS	NEG
ST36-MRSA-II, UK-16	POS	POS	POS	POS	POS	NEG	NEG	POS	NEG	POS	POS	POS	NEG	POS	NEG	NEG
ST30-MRSA-IV [PVL+], Southwest Pacific Clone	POS	POS	POS	POS	POS	NEG	NEG	POS	NEG	POS	POS	POS	NEG	POS	NEG	NEG
ST30-MRSA-IV [tst1+]	POS	POS	POS	POS	POS	NEG	NEG	POS	NEG	POS	POS	POS	NEG	POS	NEG	NEG
CC30-MRSA-V [PVL+]	POS	POS	POS	POS	POS	NEG	NEG	POS	NEG	POS	POS	POS	NEG	POS	NEG	NEG
ST45-MRSA-II, USA600	POS	POS	POS	NEG	POS	POS	NEG	POS	NEG	POS	POS	POS	NEG	NEG	POS	NEG
ST45-MRSA-IV, Berlin EMRSA	POS	POS	POS	NEG	POS	POS	NEG	POS	NEG	POS	POS	POS	NEG	NEG	POS	NEG
CC45-MRSA-IV [ACME+]	POS	POS	POS	NEG	POS	POS	NEG	POS	NEG	POS	POS	POS	NEG	NEG	POS	NEG
ST45-MRSA-V, WA-4	POS	POS	POS	NEG	POS	POS	NEG	POS	NEG	POS	POS	POS	NEG	NEG	POS	NEG
CC45/agr IV-MRSA-IV, WA-23	POS	POS	POS	NEG	POS	POS	NEG	POS	NEG	POS	POS	POS	NEG	POS	NEG	NEG
CC45/agr IV-MRSA-V	POS	POS	POS	NEG	POS	POS	NEG	POS	NEG	POS	POS	POS	NEG	POS	NEG	NEG
CC45/agr IV-MRSA-IV/V	POS	POS	POS	NEG	POS	POS	NEG	POS	NEG	POS	POS	POS	NEG	POS	NEG	NEG
CC59-MRSA-IV [PVL+], USA1000	NEG	POS	POS	POS	POS	NEG	POS	POS	POS	NEG	POS	POS	NEG	POS	NEG	NEG
ST59-MRSA-IV, WA-73	NEG	POS	POS	POS	AMB	NEG	POS	POS	POS	NEG	POS	POS	NEG	POS	NEG	NEG
ST87-MRSA-IV, WA-24	NEG	POS	POS	POS	POS	NEG	POS	POS	POS	NEG	POS	POS	NEG	POS	NEG	NEG
ST59-MRSA-IV [PVL+], WA-55/56	AMB	POS	POS	POS	AMB	NEG	POS	POS	POS	NEG	POS	POS	NEG	POS	NEG	NEG
ST59/ST952-MRSA-V(T) [PVL+], Taiwan Clone	AMB	POS	POS	POS	POS	NEG	POS	POS	POS	NEG	POS	POS	NEG	POS	NEG	NEG
ST59-MRSA-V	NEG	POS	POS	POS	POS	NEG	POS	POS	POS	NEG	POS	POS	NEG	POS	NEG	NEG
CC59-MRSA-V [PVL+]	NEG	POS	POS	POS	NEG	NEG	POS	POS	POS	NEG	POS	POS	NEG	POS	NEG	NEG
ST59-MRSA-IV/V, WA-15	NEG	POS	POS	POS	POS	NEG	POS	POS	POS	NEG	POS	POS	NEG	POS	NEG	NEG
ST72-MRSA-IV, USA700	NEG	POS	POS	POS	POS	NEG	NEG	POS	POS	NEG	POS	POS	POS	NEG	NEG	NEG
ST72-MRSA-IV [PVL+], WA-44	NEG	POS	POS	POS	POS	NEG	NEG	POS	POS	NEG	POS	POS	POS	NEG	NEG	NEG
CC75-MRSA-IV	NEG	NEG	NEG	NEG	NEG	NEG	NEG	POS	NEG	NEG	POS	POS	NEG	NEG	NEG	POS
ST883-MRSA-IV, WA-47	NEG	NEG	NEG	NEG	NEG	NEG	NEG	POS	NEG	NEG	POS	POS	NEG	NEG	NEG	NEG
ST1303-MRSA-IV	NEG	NEG	NEG	NEG	NEG	NEG	NEG	POS	NEG	NEG	POS	POS	NEG	NEG	NEG	NEG
CC80-MRSA-IV	NEG	POS	POS	POS	POS	NEG	NEG	POS	POS	NEG	POS	POS	NEG	NEG	NEG	NEG
CC80-MRSA-IV [PVL+], European Clone	NEG	POS	POS	POS	POS	NEG	NEG	POS	POS	NEG	POS	POS	NEG	NEG	NEG	NEG
CC88-MRSA-IV, WA-2	NEG	POS	POS	POS	POS	NEG	POS	POS	POS	NEG	POS	POS	NEG	NEG	NEG	NEG
CC88-MRSA-IV [PVL+]	NEG	POS	POS	POS	POS	NEG	POS	POS	POS	NEG	POS	POS	NEG	NEG	NEG	NEG
CC88-MRSA-V [PVL+]	NEG	POS	POS	POS	POS	NEG	POS	POS	POS	NEG	POS	POS	NEG	NEG	NEG	NEG
CC88-MRSA-VI	NEG	POS	POS	POS	POS	NEG	POS	POS	POS	NEG	POS	POS	NEG	NEG	NEG	NEG
ST93-MRSA-IV [PVL-]	NEG	NEG	POS	POS	POS	NEG	NEG	POS	NEG	POS	POS	POS	NEG	NEG	NEG	NEG
ST93-MRSA-IV [PVL+], Queensland Clone	NEG	NEG	POS	POS	POS	NEG	AMB	POS	NEG	POS	POS	POS	NEG	NEG	NEG	NEG
ST93-MRSA-V [PVL+]	NEG	NEG	POS	POS	POS	NEG	AMB	POS	NEG	POS	POS	POS	NEG	NEG	NEG	NEG
CC97-MRSA-IV, WA-54	NEG	POS	POS	POS	POS	NEG	NEG	POS	POS	NEG	POS	POS	NEG	NEG	NEG	NEG
CC97-MRSA-V	NEG	POS	POS	POS	POS	NEG	NEG	POS	POS	NEG	POS	POS	NEG	NEG	NEG	NEG
CC97-MRSA-V [ACME+]	NEG	POS	POS	POS	POS	NEG	NEG	POS	POS	NEG	POS	POS	NEG	NEG	NEG	NEG
CC97-MRSA-I/V	NEG	POS	POS	POS	POS	NEG	NEG	POS	POS	NEG	POS	POS	NEG	NEG	NEG	NEG
CC121-MRSA-V, WA-22	POS	POS	POS	NEG	POS	POS	NEG	POS	POS	NEG	POS	POS	NEG	NEG	NEG	NEG
CC152-MRSA-V [PVL+]	POS	POS	POS	POS	POS	NEG	NEG	POS	NEG	NEG	POS	POS	NEG	NEG	NEG	NEG
ST154-MRSA [PVL+]	POS	POS	POS	AMB	POS	NEG	POS	POS	POS	NEG	POS	POS	NEG	POS	NEG	NEG
ST188-MRSA-IV, WA-38/78	POS	POS	POS	POS	POS	NEG	POS	POS	POS	NEG	POS	POS	NEG	NEG	NEG	NEG
ST188-MRSA-V	POS	POS	POS	POS	POS	NEG	POS	POS	POS	NEG	POS	POS	NEG	NEG	NEG	NEG
ST239-MRSA-III	POS	POS	POS	POS	POS	NEG	POS	POS	POS	NEG	COMM	COMM	POS	RARE	NEG	NEG
CC361-MRSA-IV, WA-29	NEG	POS	POS	POS	POS	NEG	POS	POS	POS	NEG	POS	POS	NEG	NEG	NEG	NEG
CC361-MRSA-V	NEG	POS	POS	POS	POS	NEG	POS	POS	POS	NEG	POS	POS	NEG	NEG	NEG	NEG
CC361-MRSA-VIII, WA-28	NEG	POS	POS	AMB	POS	NEG	POS	POS	POS	NEG	POS	POS	NEG	NEG	NEG	NEG
CC398-MRSA-IV	POS	POS	POS	NEG	POS	POS	POS	POS	NEG	POS	POS	POS	NEG	NEG	NEG	NEG
ST398-MRSA-V	POS	POS	POS	NEG	POS	POS	POS	POS	NEG	POS	COMM	COMM	NEG	NEG	NEG	NEG
ST398-MRSA-V [PVL+]	POS	POS	POS	NEG	POS	POS	POS	POS	NEG	POS	AMB	AMB	NEG	NEG	NEG	NEG
CC509/ST207-MRSA-V	POS	NEG	POS	POS	POS	NEG	NEG	POS	POS	POS	POS	POS	NEG	NEG	NEG	NEG
ST573-MRSA-V, WA-10	POS	POS	POS	POS	POS	NEG	NEG	POS	POS	NEG	POS	POS	NEG	NEG	AMB	NEG
ST772-MRSA-V [PVL+], WA-60/Bengal Bay Clone	POS	POS	POS	POS	POS	NEG	NEG	POS	POS	NEG	POS	POS	NEG	NEG	POS	NEG
ST779-MRSA	NEG	POS	POS	POS	POS	NEG	NEG	POS	POS	NEG	POS	POS	NEG	POS	NEG	NEG
ST834-MRSA-IV, WA-13	NEG	POS	POS	POS	POS	NEG	NEG	POS	POS	NEG	POS	POS	NEG	POS	NEG	NEG
CC913-MRSA-IV	NEG	POS	POS	POS	POS	NEG	POS	POS	POS	NEG	POS	POS	NEG	NEG	NEG	NEG
ST1774-MRSA-IV [ACME+]	POS	POS	POS	POS	POS	NEG	POS	POS	POS	NEG	POS	POS	NEG	NEG	NEG	NEG

CC15-MSSA	NEG	POS	POS	POS	POS	NEG	POS	POS	POS	NEG	POS	POS	POS	NEG	NEG	NEG
ST426-MSSA	COMM	COMM	POS	POS	POS	NEG	NEG	POS	NEG	POS	POS	POS	NEG	NEG	NEG	NEG

Legend:

Positive :	POS
Ambiguous :	AMB
Negative :	NEG
Rare (in >0 to 33% of isolates) :	RARE
Variable (in >33 to 66% of isolates) :	VAR
Common (in >66 to <100% of isolates) :	COMM

	ADHAESION FACTORS / GENES ENCODING MICROBIAL SURFACE COMPONENTS RECOGNIZING ADHESIVE MATRIX MOLECULES (MSCRAMM GENES), continued															
	fnbB (total)	fnbB (COL)	fnbB (COL+Mu50+ MW2)	fnbB (Mu50)	fnbB (MW2)	fnbB (ST15)	fnbB (ST45-2)	map (total)	map (COL)	map (MRSA252)	map (Mu50+ MW2)	sasG (total)	sasG (COL+Mu50)	sasG (MW2)	sasG (Other than MRSA252+ RF122)
CC1-MRSA-IV [PVL-], WA-1/57	POS	NEG	AMB	NEG	POS	NEG	NEG	POS	NEG	NEG	POS	POS	NEG	POS	POS
CC1-MRSA-IV [PVL+], USA400	POS	NEG	AMB	NEG	POS	NEG	NEG	POS	NEG	NEG	POS	POS	NEG	POS	POS
CC1-MRSA-IV/SCCfus [PVL-]	POS	NEG	AMB	NEG	POS	NEG	NEG	POS	NEG	NEG	POS	POS	NEG	POS	POS
CC1-MRSA-IV/SCCfus [PVL+]	POS	NEG	AMB	NEG	POS	NEG	NEG	POS	NEG	NEG	POS	POS	NEG	POS	POS
CC1-MRSA-V [PVL+]	NEG	NEG	AMB	NEG	AMB	NEG	NEG	POS	AMB	NEG	POS	POS	NEG	POS	POS
CC1-MRSA-V/SCCfus [PVL-]	AMB	NEG	AMB	NEG	AMB	NEG	NEG	POS	AMB	NEG	POS	POS	NEG	POS	POS
CC1-MRSA-V/SCCfus [PVL+]	NEG	NEG	AMB	NEG	AMB	NEG	NEG	POS	AMB	NEG	POS	POS	NEG	POS	POS
ST228-MRSA-I, South German/Italian MRSA	NEG	NEG	NEG	NEG	NEG	NEG	NEG	POS	AMB	NEG	POS	POS	POS	NEG	POS
ST5-MRSA-I, Geraldine Clone	COMM	NEG	RARE	VAR	NEG	NEG	NEG	POS	AMB	NEG	POS	POS	POS	NEG	POS
CC5-MRSA-Ivar., WA-18/21/48	POS	NEG	POS	AMB	NEG	NEG	NEG	POS	AMB	NEG	POS	POS	POS	NEG	POS
CC5-MRSA-II, Rhine-Hesse /UK-3	POS	NEG	AMB	AMB	NEG	NEG	NEG	POS	AMB	NEG	POS	POS	POS	NEG	POS
CC5-MRSA-II [ACME+]	POS	NEG	AMB	POS	NEG	AMB	NEG	POS	POS	NEG	POS	POS	POS	NEG	POS
CC5-MRSA-III	POS	NEG	POS	AMB	NEG	NEG	NEG	POS	AMB	NEG	POS	POS	POS	NEG	POS
CC5-MRSA-IV, Paediatric clone	POS	NEG	POS	AMB	NEG	NEG	NEG	POS	AMB	NEG	POS	POS	POS	NEG	POS
CC5-MRSA-IV [PVL+]	POS	NEG	AMB	POS	NEG	NEG	NEG	POS	AMB	NEG	POS	POS	POS	NEG	POS
CC5-MRSA-V	POS	NEG	POS	AMB	NEG	NEG	NEG	POS	AMB	NEG	POS	POS	POS	NEG	POS
CC5-MRSA-V [PVL+]	POS	NEG	AMB	POS	NEG	NEG	NEG	POS	POS	NEG	POS	POS	POS	NEG	POS
CC5-MRSA-VI, New Paediatric Clone	POS	NEG	AMB	POS	NEG	NEG	NEG	POS	AMB	NEG	POS	POS	POS	NEG	POS
ST5-MRSA-VII (SCC-JCSC6082)	POS	NEG	POS	AMB	NEG	NEG	NEG	POS	NEG	NEG	POS	POS	POS	NEG	POS
CC5-MRSA-IVvar, Maltese Clone	POS	NEG	AMB	POS	NEG	NEG	NEG	POS	POS	NEG	POS	POS	POS	NEG	POS
CC5-MRSA-SCC(MRSAZH47)	POS	NEG	AMB	POS	NEG	NEG	NEG	POS	AMB	NEG	POS	POS	POS	NEG	POS
CC5-MRSA-IV/VI	POS	NEG	POS	AMB	NEG	NEG	NEG	POS	AMB	NEG	POS	POS	POS	NEG	POS
CC5-MRSA-IV/SCCfus	POS	NEG	POS	AMB	NEG	NEG	NEG	POS	POS	NEG	POS	POS	POS	NEG	POS
CC5/ST835-MRSA, WA-40/46	POS	NEG	POS	POS	NEG	NEG	NEG	POS	AMB	NEG	POS	POS	POS	NEG	POS
ST6-MRSA-IV, WA-51	POS	NEG	AMB	AMB	NEG	NEG	NEG	POS	NEG	NEG	POS	POS	NEG	POS	POS
ST6-MRSA-V	POS	NEG	AMB	POS	NEG	NEG	NEG	POS	AMB	NEG	POS	POS	NEG	POS	POS
CC7-MRSA-IV	POS	NEG	AMB	NEG	NEG	NEG	POS	POS	NEG	NEG	POS	NEG	NEG	NEG	NEG
CC7-MRSA-V	POS	NEG	AMB	NEG	NEG	NEG	POS	POS	NEG	NEG	POS	NEG	NEG	NEG	NEG
ST250-MRSA-I, Early/Ancestral MRSA	POS	POS	AMB	NEG	NEG	NEG	NEG	POS	POS	NEG	NEG	POS	POS	NEG	POS
ST247-MRSA-I, North German/Iberian MRSA	COMM	COMM	AMB	NEG	NEG	NEG	NEG	POS	POS	NEG	NEG	POS	POS	NEG	POS
CC8-MRSA-IV, UK-14/WA-5	POS	POS	AMB	AMB	NEG	NEG	NEG	POS	POS	NEG	AMB	POS	POS	NEG	POS
CC8-MRSA-IV, Lyon Clone/UK-2	COMM	COMM	AMB	NEG	NEG	NEG	NEG	POS	POS	NEG	NEG	POS	POS	NEG	POS
CC8-MRSA-IV, UK-6	POS	POS	AMB	AMB	NEG	NEG	NEG	POS	POS	NEG	AMB	POS	POS	NEG	POS
CC8-MRSA-IV, USA500	POS	POS	AMB	AMB	NEG	NEG	NEG	POS	POS	NEG	AMB	POS	POS	NEG	POS
ST8-MRSA-IV [PVL/ACME+], USA300	POS	POS	POS	NEG	NEG	NEG	NEG	POS	POS	NEG	NEG	POS	POS	NEG	POS
ST8-MRSA-IV [PVL+], USA300	POS	POS	POS	NEG	NEG	NEG	NEG	POS	POS	NEG	NEG	POS	POS	NEG	POS
CC8-MRSA-IV [PVL/sed/j/r/k/q+]	POS	POS	AMB	AMB	NEG	NEG	NEG	POS	POS	NEG	POS	POS	POS	NEG	POS
CC8-MRSA-IV [tst1/sec/sel+]	POS	POS	AMB	AMB	NEG	NEG	NEG	POS	POS	NEG	AMB	POS	POS	NEG	POS
CC8-MRSA-V	POS	POS	AMB	NEG	NEG	NEG	NEG	POS	POS	NEG	NEG	POS	POS	NEG	POS
CC8-MRSA-VIII	POS	AMB	AMB	NEG	NEG	NEG	NEG	POS	POS	NEG	NEG	VAR	VAR	NEG	VAR
ST8-MRSA-IIA/B/C/D/E, Irish AR13/14	POS	POS	AMB	NEG	NEG	NEG	NEG	POS	POS	NEG	NEG	NEG	NEG	NEG	NEG
ST8-MRSA, UK-12/13, Irish AR43	POS	POS	AMB	AMB	NEG	NEG	NEG	POS	POS	NEG	AMB	POS	POS	NEG	POS
ST254-MRSA, Hannover EMRSA	POS	POS	POS	AMB	NEG	NEG	NEG	POS	POS	NEG	AMB	POS	POS	NEG	POS
ST254-MRSA-IV/V, Hannover/UK-10	POS	POS	AMB	AMB	NEG	NEG	NEG	POS	POS	NEG	AMB	POS	POS	NEG	POS
CC9-MRSA-IV	POS	NEG	POS	AMB	NEG	NEG	NEG	POS	POS	AMB	POS	NEG	NEG	NEG	NEG
CC12-MRSA, WA-59	POS	NEG	POS	NEG	NEG	NEG	POS	POS	POS	NEG	POS	NEG	NEG	NEG	NEG
CC12-MRSA-IV	POS	NEG	AMB	NEG	NEG	NEG	POS	POS	POS	NEG	POS	NEG	NEG	NEG	NEG
CC20-MRSA-V	POS	NEG	AMB	POS	NEG	AMB	NEG	POS	NEG	NEG	POS	POS	POS	NEG	POS
ST22-MRSA-IV, Barnim/UK-15	NEG	NEG	RARE	NEG	RARE	NEG	NEG	AMB	NEG	NEG	AMB	POS	NEG	POS	POS
ST22-MRSA-IV [ACME+]	NEG	NEG	NEG	NEG	NEG	NEG	NEG	POS	NEG	NEG	POS	POS	NEG	POS	POS
ST22-MRSA-IV [PVL+]	POS	NEG	AMB	NEG	POS	NEG	NEG	POS	NEG	NEG	POS	POS	NEG	POS	POS
ST22-MRSA-V	POS	NEG	AMB	NEG	POS	NEG	NEG	POS	NEG	NEG	POS	POS	NEG	POS	POS
ST22-MRSA-V [PVL+]	POS	NEG	NEG	NEG	POS	NEG	NEG	POS	NEG	NEG	POS	POS	NEG	POS	POS
ST36-MRSA-II, UK-16	NEG	NEG	RARE	RARE	NEG	NEG	NEG	POS	NEG	POS	NEG	NEG	NEG	NEG	NEG
ST30-MRSA-IV [PVL+], Southwest Pacific Clone	POS	NEG	AMB	POS	NEG	NEG	NEG	POS	NEG	POS	NEG	NEG	NEG	NEG	NEG
ST30-MRSA-IV [tst1+]	NEG	NEG	NEG	NEG	NEG	NEG	NEG	POS	NEG	POS	NEG	NEG	NEG	NEG	NEG
CC30-MRSA-V [PVL+]	POS	NEG	POS	AMB	NEG	NEG	NEG	POS	NEG	POS	NEG	NEG	NEG	NEG	NEG
ST45-MRSA-II, USA600	POS	NEG	POS	AMB	NEG	NEG	NEG	POS	NEG	POS	NEG	NEG	NEG	NEG	NEG
ST45-MRSA-IV, Berlin EMRSA	POS	NEG	POS	AMB	NEG	NEG	NEG	POS	NEG	POS	NEG	NEG	NEG	NEG	NEG
CC45-MRSA-IV [ACME+]	AMB	NEG	AMB	NEG	NEG	NEG	NEG	POS	AMB	POS	NEG	NEG	NEG	NEG	NEG
ST45-MRSA-V, WA-4	POS	NEG	POS	AMB	NEG	NEG	NEG	POS	NEG	POS	NEG	NEG	NEG	NEG	NEG
CC45/agr IV-MRSA-IV, WA-23	POS	NEG	AMB	NEG	NEG	NEG	POS	POS	NEG	POS	NEG	POS	NEG	POS	POS
CC45/agr IV-MRSA-V	POS	NEG	AMB	NEG	NEG	NEG	POS	POS	NEG	POS	NEG	POS	NEG	POS	POS
CC45/agr IV-MRSA-IV/V	COMM	NEG	AMB	NEG	NEG	NEG	COMM	POS	NEG	POS	NEG	POS	NEG	POS	POS
CC59-MRSA-IV [PVL+], USA1000	POS	NEG	POS	NEG	NEG	NEG	NEG	POS	POS	NEG	POS	POS	NEG	POS	POS
ST59-MRSA-IV, WA-73	POS	NEG	POS	NEG	NEG	NEG	NEG	POS	POS	NEG	AMB	POS	AMB	POS	POS
ST87-MRSA-IV, WA-24	POS	NEG	POS	AMB	NEG	NEG	NEG	POS	POS	NEG	POS	POS	NEG	POS	POS
ST59-MRSA-IV [PVL+], WA-55/56	POS	NEG	POS	AMB	NEG	NEG	NEG	POS	POS	NEG	AMB	POS	NEG	POS	POS
ST59/ST952-MRSA-V(T) [PVL+], Taiwan Clone	POS	NEG	POS	AMB	NEG	NEG	NEG	POS	POS	NEG	POS	POS	NEG	POS	POS
ST59-MRSA-V	POS	NEG	POS	AMB	NEG	NEG	NEG	POS	POS	NEG	POS	POS	NEG	POS	POS
CC59-MRSA-V [PVL+]	POS	NEG	POS	NEG	NEG	NEG	NEG	POS	POS	NEG	NEG	NEG	NEG	NEG	NEG
ST59-MRSA-IV/V, WA-15	POS	NEG	POS	AMB	NEG	NEG	NEG	POS	POS	NEG	POS	POS	NEG	POS	POS
ST72-MRSA-IV, USA700	POS	NEG	POS	AMB	NEG	NEG	NEG	POS	NEG	NEG	POS	POS	NEG	POS	POS
ST72-MRSA-IV [PVL+], WA-44	POS	NEG	POS	AMB	NEG	NEG	NEG	POS	AMB	NEG	POS	POS	NEG	POS	POS
CC75-MRSA-IV	POS	NEG	NEG	POS	NEG	POS	NEG	NEG	NEG	NEG	NEG	POS	NEG	POS	NEG
ST883-MRSA-IV, WA-47	AMB	NEG	AMB	POS	NEG	AMB	NEG	NEG	NEG	NEG	NEG	NEG	NEG	NEG	NEG
ST1303-MRSA-IV	POS	NEG	POS	AMB	NEG	POS	NEG	NEG	NEG	NEG	NEG	NEG	NEG	NEG	NEG
CC80-MRSA-IV	POS	NEG	AMB	NEG	POS	NEG	NEG	POS	NEG	NEG	POS	POS	NEG	POS	POS
CC80-MRSA-IV [PVL+], European Clone	POS	NEG	AMB	NEG	POS	NEG	NEG	POS	NEG	NEG	POS	POS	NEG	POS	POS
CC88-MRSA-IV, WA-2	POS	NEG	AMB	POS	NEG	NEG	NEG	POS	NEG	NEG	POS	POS	NEG	POS	POS
CC88-MRSA-IV [PVL+]	POS	NEG	AMB	POS	NEG	NEG	NEG	POS	NEG	NEG	POS	POS	NEG	POS	POS
CC88-MRSA-V [PVL+]	POS	NEG	AMB	POS	NEG	NEG	NEG	POS	AMB	NEG	POS	POS	NEG	POS	POS
CC88-MRSA-VI	POS	NEG	AMB	POS	NEG	NEG	NEG	POS	NEG	NEG	POS	POS	NEG	POS	POS
ST93-MRSA-IV [PVL-]	POS	NEG	POS	NEG	NEG	NEG	NEG	NEG	NEG	NEG	NEG	NEG	NEG	NEG	NEG
ST93-MRSA-IV [PVL+], Queensland Clone	POS	NEG	POS	AMB	NEG	NEG	NEG	NEG	NEG	NEG	NEG	NEG	NEG	NEG	NEG
ST93-MRSA-V [PVL+]	POS	NEG	POS	AMB	NEG	NEG	NEG	NEG	NEG	NEG	NEG	NEG	NEG	NEG	NEG
CC97-MRSA-IV, WA-54	POS	NEG	POS	POS	NEG	NEG	NEG	POS	POS	NEG	POS	POS	POS	NEG	POS
CC97-MRSA-V	POS	NEG	POS	AMB	NEG	NEG	NEG	POS	POS	NEG	POS	POS	POS	NEG	POS
CC97-MRSA-V [ACME+]	POS	NEG	AMB	AMB	NEG	NEG	NEG	POS	POS	AMB	POS	POS	POS	NEG	POS
CC97-MRSA-I/V	POS	NEG	AMB	AMB	NEG	NEG	NEG	POS	POS	NEG	POS	POS	POS	NEG	POS
CC121-MRSA-V, WA-22	POS	NEG	POS	NEG	NEG	NEG	NEG	POS	POS	NEG	AMB	NEG	NEG	NEG	NEG
CC152-MRSA-V [PVL+]	POS	NEG	POS	AMB	NEG	NEG	NEG	NEG	NEG	NEG	NEG	NEG	NEG	NEG	NEG
ST154-MRSA [PVL+]	POS	NEG	AMB	AMB	NEG	AMB	NEG	POS	NEG	NEG	POS	POS	POS	NEG	POS
ST188-MRSA-IV, WA-38/78	POS	NEG	AMB	POS	NEG	AMB	NEG	POS	AMB	NEG	POS	NEG	NEG	NEG	NEG
ST188-MRSA-V	POS	NEG	AMB	POS	NEG	AMB	NEG	POS	POS	NEG	POS	NEG	NEG	AMB	NEG
ST239-MRSA-III	COMM	COMM	RARE	AMB	NEG	NEG	NEG	POS	POS	NEG	AMB	POS	POS	NEG	POS
CC361-MRSA-IV, WA-29	POS	NEG	POS	AMB	NEG	AMB	NEG	POS	NEG	NEG	POS	POS	NEG	POS	POS
CC361-MRSA-V	POS	NEG	POS	NEG	NEG	AMB	NEG	POS	NEG	NEG	POS	POS	NEG	POS	POS
CC361-MRSA-VIII, WA-28	POS	NEG	POS	NEG	NEG	NEG	NEG	POS	NEG	NEG	POS	POS	NEG	POS	POS
CC398-MRSA-IV	POS	NEG	POS	NEG	NEG	NEG	NEG	POS	POS	POS	NEG	NEG	NEG	NEG	NEG
ST398-MRSA-V	COMM	NEG	COMM	NEG	NEG	NEG	NEG	POS	POS	POS	NEG	NEG	NEG	NEG	NEG
ST398-MRSA-V [PVL+]	POS	NEG	POS	AMB	NEG	NEG	NEG	POS	POS	POS	NEG	NEG	NEG	NEG	NEG
CC509/ST207-MRSA-V	POS	NEG	POS	NEG	NEG	NEG	NEG	POS	NEG	NEG	AMB	POS	NEG	POS	POS
ST573-MRSA-V, WA-10	POS	NEG	POS	POS	NEG	POS	NEG	POS	NEG	NEG	POS	POS	NEG	POS	POS
ST772-MRSA-V [PVL+], WA-60/Bengal Bay Clone	POS	NEG	POS	NEG	NEG	AMB	NEG	POS	NEG	NEG	POS	POS	NEG	POS	POS
ST779-MRSA	POS	NEG	AMB	POS	NEG	NEG	NEG	AMB	AMB	NEG	NEG	POS	POS	NEG	POS
ST834-MRSA-IV, WA-13	POS	NEG	POS	POS	NEG	AMB	NEG	POS	NEG	NEG	POS	POS	POS	NEG	POS
CC913-MRSA-IV	POS	NEG	POS	NEG	NEG	NEG	NEG	POS	NEG	NEG	POS	POS	NEG	POS	POS
ST1774-MRSA-IV [ACME+]	POS	NEG	AMB	POS	NEG	AMB	NEG	POS	POS	NEG	POS	NEG	NEG	NEG	NEG
CC15-MSSA	POS	NEG	AMB	AMB	NEG	AMB	NEG	POS	AMB	NEG	POS	POS	NEG	POS	POS
ST426-MSSA	AMB	NEG	AMB	NEG	NEG	NEG	COMM	POS	NEG	POS	NEG	NEG	NEG	NEG	NEG

Legend:

Positive :	POS
Ambiguous :	AMB
Negative :	NEG
Rare (in >0 to 33% of isolates) :	RARE
Variable (in >33 to 66% of isolates) :	VAR
Common (in >66 to <100% of isolates) :	COMM

	ADHAESION FACTORS / GENES ENCODING MICROBIAL SURFACE COMPONENTS RECOGNIZING ADHESIVE MATRIX MOLECULES (MSCRAMM GENES), continued																	
	sdrC (total)	sdrC (cons)	sdrC (B1)	sdrC (COL)	sdrC (Mu50)	sdrC (MW2+ MRSA252+ RF122)	sdrC (Other than MRSA252+ RF122)	sdrD (total)	sdrD (cons)	sdrD (COL+MW2)	sdrD (Mu50)	sdrD (other)	vwb (total)	vwb (cons)	vwb (COL+ MW2)	vwb (MRSA252)	vwb (Mu50)	vwb (RF122)
CC1-MRSA-IV [PVL-], WA-1/57	POS	POS	NEG	NEG	NEG	POS	POS	POS	POS	POS	NEG	NEG	POS	POS	POS	NEG	NEG	NEG
CC1-MRSA-IV [PVL+], USA400	POS	POS	NEG	NEG	NEG	POS	POS	POS	POS	POS	NEG	NEG	POS	POS	POS	NEG	NEG	NEG
CC1-MRSA-IV/SCCfus [PVL-]	POS	POS	NEG	NEG	NEG	POS	POS	POS	POS	POS	NEG	NEG	POS	POS	POS	NEG	NEG	NEG
CC1-MRSA-IV/SCCfus [PVL+]	POS	POS	NEG	NEG	NEG	POS	POS	POS	POS	AMB	NEG	NEG	POS	POS	POS	NEG	NEG	NEG
CC1-MRSA-V [PVL+]	POS	POS	NEG	NEG	NEG	POS	POS	POS	POS	POS	NEG	NEG	POS	POS	POS	NEG	NEG	NEG
CC1-MRSA-V/SCCfus [PVL-]	POS	POS	NEG	NEG	NEG	POS	POS	POS	POS	POS	NEG	NEG	POS	POS	POS	NEG	NEG	NEG
CC1-MRSA-V/SCCfus [PVL+]	POS	POS	NEG	NEG	NEG	POS	POS	POS	POS	POS	NEG	NEG	POS	POS	POS	NEG	NEG	NEG
ST228-MRSA-I, South German/Italian MRSA	POS	POS	NEG	NEG	POS	NEG	POS	POS	POS	NEG	POS	NEG	POS	POS	NEG	NEG	POS	NEG
ST5-MRSA-I, Geraldine Clone	POS	POS	NEG	NEG	POS	NEG	POS	COMM	COMM	NEG	COMM	NEG	POS	POS	NEG	NEG	POS	NEG
CC5-MRSA-Ivar., WA-18/21/48	POS	POS	NEG	NEG	POS	NEG	POS	POS	POS	NEG	POS	NEG	POS	POS	NEG	NEG	POS	NEG
CC5-MRSA-II, Rhine-Hesse /UK-3	POS	POS	NEG	NEG	POS	NEG	POS	POS	POS	NEG	POS	NEG	POS	POS	NEG	NEG	POS	NEG
CC5-MRSA-II [ACME+]	POS	POS	NEG	NEG	POS	NEG	POS	POS	POS	NEG	POS	NEG	POS	POS	NEG	NEG	POS	NEG
CC5-MRSA-III	POS	POS	NEG	NEG	POS	NEG	POS	POS	POS	NEG	POS	NEG	POS	POS	NEG	NEG	POS	NEG
CC5-MRSA-IV, Paediatric clone	POS	POS	NEG	NEG	POS	NEG	POS	COMM	COMM	NEG	COMM	NEG	POS	POS	NEG	NEG	POS	NEG
CC5-MRSA-IV [PVL+]	POS	POS	NEG	NEG	POS	NEG	POS	COMM	COMM	NEG	COMM	NEG	POS	POS	NEG	NEG	POS	NEG
CC5-MRSA-V	POS	POS	NEG	NEG	POS	NEG	POS	POS	POS	NEG	POS	NEG	POS	POS	NEG	NEG	POS	NEG
CC5-MRSA-V [PVL+]	POS	POS	NEG	NEG	POS	NEG	POS	POS	POS	NEG	POS	NEG	POS	POS	NEG	NEG	POS	NEG
CC5-MRSA-VI, New Paediatric Clone	COMM	COMM	NEG	NEG	COMM	NEG	COMM	RARE	RARE	NEG	RARE	NEG	POS	POS	NEG	NEG	POS	NEG
ST5-MRSA-VII (SCC-JCSC6082)	POS	POS	NEG	NEG	POS	NEG	POS	POS	POS	NEG	POS	NEG	POS	POS	NEG	NEG	POS	NEG
CC5-MRSA-IVvar, Maltese Clone	POS	POS	NEG	NEG	POS	NEG	POS	POS	POS	NEG	POS	NEG	POS	POS	NEG	NEG	POS	NEG
CC5-MRSA-SCC(MRSAZH47)	POS	POS	NEG	NEG	POS	NEG	POS	POS	POS	NEG	POS	NEG	POS	POS	NEG	NEG	POS	NEG
CC5-MRSA-IV/VI	POS	POS	NEG	NEG	POS	NEG	POS	POS	COMM	NEG	COMM	NEG	POS	POS	NEG	NEG	POS	NEG
CC5-MRSA-IV/SCCfus	POS	POS	NEG	NEG	POS	NEG	POS	POS	POS	NEG	POS	NEG	POS	POS	NEG	NEG	POS	NEG
CC5/ST835-MRSA, WA-40/46	POS	POS	NEG	NEG	POS	NEG	POS	POS	POS	NEG	POS	NEG	POS	POS	NEG	NEG	POS	NEG
ST6-MRSA-IV, WA-51	POS	POS	NEG	POS	NEG	NEG	POS	POS	POS	NEG	POS	NEG	POS	POS	NEG	POS	NEG	NEG
ST6-MRSA-V	POS	POS	NEG	POS	NEG	AMB	POS	POS	POS	NEG	POS	NEG	POS	POS	NEG	POS	NEG	NEG
CC7-MRSA-IV	POS	POS	NEG	POS	NEG	NEG	POS	POS	POS	NEG	POS	NEG	POS	POS	NEG	NEG	AMB	NEG
CC7-MRSA-V	POS	POS	NEG	POS	NEG	NEG	POS	POS	POS	NEG	POS	NEG	POS	POS	NEG	NEG	AMB	NEG
ST250-MRSA-I, Early/Ancestral MRSA	POS	POS	NEG	POS	NEG	NEG	POS	POS	POS	POS	NEG	NEG	POS	POS	POS	NEG	NEG	NEG
ST247-MRSA-I, North German/Iberian MRSA	POS	POS	NEG	POS	NEG	NEG	POS	POS	POS	POS	NEG	NEG	POS	POS	POS	NEG	NEG	NEG
CC8-MRSA-IV, UK-14/WA-5	POS	POS	NEG	POS	NEG	NEG	POS	COMM	COMM	COMM	NEG	NEG	COMM	COMM	COMM	NEG	NEG	NEG
CC8-MRSA-IV, Lyon Clone/UK-2	POS	POS	NEG	POS	NEG	NEG	POS	COMM	COMM	AMB	NEG	NEG	POS	POS	POS	NEG	NEG	NEG
CC8-MRSA-IV, UK-6	POS	POS	NEG	POS	NEG	AMB	POS	POS	POS	POS	NEG	NEG	POS	POS	POS	NEG	NEG	NEG
CC8-MRSA-IV, USA500	POS	POS	NEG	POS	NEG	NEG	POS	POS	POS	POS	NEG	NEG	POS	POS	POS	NEG	NEG	NEG
ST8-MRSA-IV [PVL/ACME+], USA300	POS	POS	NEG	POS	NEG	NEG	POS	POS	POS	POS	NEG	NEG	POS	POS	POS	NEG	NEG	NEG
ST8-MRSA-IV [PVL+], USA300	POS	POS	NEG	POS	NEG	NEG	POS	POS	POS	AMB	NEG	NEG	POS	POS	POS	NEG	NEG	NEG
CC8-MRSA-IV [PVL/sed/j/r/k/q+]	POS	POS	NEG	POS	NEG	NEG	POS	POS	POS	POS	NEG	NEG	POS	POS	POS	NEG	NEG	NEG
CC8-MRSA-IV [tst1/sec/sel+]	POS	POS	NEG	POS	NEG	AMB	POS	POS	POS	POS	NEG	NEG	POS	POS	POS	NEG	NEG	NEG
CC8-MRSA-V	POS	POS	NEG	POS	NEG	NEG	POS	POS	POS	POS	NEG	NEG	POS	POS	POS	NEG	NEG	NEG
CC8-MRSA-VIII	VAR	VAR	NEG	VAR	NEG	NEG	VAR	POS	POS	AMB	NEG	NEG	POS	POS	POS	NEG	NEG	NEG
ST8-MRSA-IIA/B/C/D/E, Irish AR13/14	NEG	NEG	NEG	NEG	NEG	NEG	NEG	POS	POS	POS	NEG	NEG	COMM	COMM	COMM	NEG	NEG	NEG
ST8-MRSA, UK-12/13, Irish AR43	POS	POS	NEG	POS	NEG	NEG	POS	POS	POS	POS	NEG	NEG	POS	POS	POS	NEG	NEG	NEG
ST254-MRSA, Hannover EMRSA	POS	POS	NEG	POS	NEG	AMB	POS	POS	POS	POS	NEG	NEG	POS	POS	POS	NEG	NEG	NEG
ST254-MRSA-IV/V, Hannover/UK-10	POS	POS	NEG	POS	NEG	AMB	POS	POS	POS	POS	NEG	NEG	POS	POS	POS	NEG	NEG	NEG
CC9-MRSA-IV	POS	POS	NEG	NEG	POS	POS	POS	POS	POS	NEG	NEG	POS	POS	POS	NEG	POS	NEG	NEG
CC12-MRSA, WA-59	POS	POS	NEG	POS	NEG	NEG	POS	NEG	AMB	AMB	NEG	NEG	POS	POS	NEG	NEG	NEG	NEG
CC12-MRSA-IV	POS	POS	NEG	POS	NEG	NEG	POS	NEG	AMB	AMB	NEG	NEG	POS	POS	NEG	NEG	NEG	NEG
CC20-MRSA-V	POS	POS	NEG	NEG	POS	AMB	POS	POS	POS	NEG	NEG	POS	POS	POS	NEG	NEG	POS	NEG
ST22-MRSA-IV, Barnim/UK-15	POS	POS	NEG	NEG	POS	NEG	POS	POS	POS	NEG	NEG	POS	POS	POS	NEG	NEG	NEG	POS
ST22-MRSA-IV [ACME+]	POS	POS	NEG	NEG	POS	NEG	POS	POS	POS	NEG	NEG	POS	POS	POS	NEG	NEG	NEG	POS
ST22-MRSA-IV [PVL+]	POS	POS	NEG	NEG	POS	NEG	POS	POS	POS	NEG	NEG	POS	POS	POS	NEG	NEG	NEG	POS
ST22-MRSA-V	POS	POS	NEG	NEG	POS	NEG	POS	POS	POS	NEG	NEG	POS	POS	POS	NEG	NEG	NEG	POS
ST22-MRSA-V [PVL+]	POS	POS	NEG	NEG	POS	NEG	POS	POS	POS	NEG	NEG	POS	POS	POS	NEG	NEG	NEG	POS
ST36-MRSA-II, UK-16	POS	POS	NEG	NEG	NEG	NEG	NEG	COMM	COMM	NEG	NEG	COMM	POS	POS	NEG	POS	NEG	NEG
ST30-MRSA-IV [PVL+], Southwest Pacific Clone	POS	POS	NEG	NEG	NEG	AMB	NEG	POS	POS	NEG	NEG	POS	POS	POS	NEG	POS	NEG	NEG
ST30-MRSA-IV [tst1+]	POS	POS	NEG	NEG	NEG	NEG	NEG	POS	POS	NEG	NEG	POS	POS	POS	NEG	POS	NEG	NEG
CC30-MRSA-V [PVL+]	POS	POS	NEG	NEG	NEG	NEG	NEG	POS	POS	NEG	NEG	POS	POS	POS	NEG	POS	NEG	NEG
ST45-MRSA-II, USA600	POS	POS	POS	NEG	NEG	NEG	NEG	POS	POS	POS	NEG	NEG	POS	AMB	NEG	POS	NEG	NEG
ST45-MRSA-IV, Berlin EMRSA	POS	POS	POS	NEG	NEG	NEG	NEG	POS	POS	POS	NEG	NEG	POS	NEG	NEG	POS	NEG	NEG
CC45-MRSA-IV [ACME+]	POS	POS	POS	NEG	NEG	NEG	NEG	POS	POS	POS	NEG	NEG	POS	AMB	NEG	POS	NEG	NEG
ST45-MRSA-V, WA-4	POS	POS	POS	NEG	NEG	NEG	NEG	POS	POS	POS	NEG	NEG	POS	AMB	NEG	POS	NEG	NEG
CC45/agr IV-MRSA-IV, WA-23	POS	POS	POS	NEG	NEG	NEG	NEG	POS	POS	NEG	NEG	POS	POS	POS	NEG	NEG	NEG	NEG
CC45/agr IV-MRSA-V	POS	POS	POS	NEG	NEG	NEG	NEG	COMM	COMM	NEG	NEG	COMM	POS	POS	NEG	NEG	NEG	NEG
CC45/agr IV-MRSA-IV/V	POS	POS	POS	NEG	NEG	NEG	NEG	POS	POS	NEG	NEG	POS	POS	POS	NEG	NEG	NEG	NEG
CC59-MRSA-IV [PVL+], USA1000	POS	POS	NEG	NEG	NEG	POS	NEG	COMM	COMM	NEG	NEG	NEG	POS	POS	NEG	NEG	NEG	NEG
ST59-MRSA-IV, WA-73	POS	POS	NEG	NEG	NEG	AMB	NEG	POS	POS	NEG	NEG	NEG	POS	POS	NEG	NEG	NEG	NEG
ST87-MRSA-IV, WA-24	POS	POS	NEG	NEG	NEG	POS	NEG	POS	POS	NEG	NEG	NEG	POS	POS	NEG	NEG	NEG	NEG
ST59-MRSA-IV [PVL+], WA-55/56	POS	POS	NEG	NEG	NEG	AMB	NEG	POS	POS	NEG	AMB	NEG	POS	POS	NEG	NEG	NEG	NEG
ST59/ST952-MRSA-V(T) [PVL+], Taiwan Clone	POS	POS	NEG	NEG	NEG	POS	NEG	POS	POS	NEG	AMB	NEG	POS	POS	NEG	NEG	NEG	NEG
ST59-MRSA-V	POS	POS	NEG	NEG	NEG	POS	NEG	POS	POS	NEG	AMB	NEG	POS	POS	NEG	NEG	NEG	NEG
CC59-MRSA-V [PVL+]	POS	POS	NEG	NEG	NEG	NEG	NEG	POS	POS	NEG	NEG	NEG	POS	POS	NEG	NEG	NEG	NEG
ST59-MRSA-IV/V, WA-15	POS	POS	NEG	NEG	NEG	POS	NEG	POS	POS	NEG	AMB	NEG	POS	POS	NEG	NEG	NEG	NEG
ST72-MRSA-IV, USA700	POS	POS	NEG	NEG	POS	AMB	POS	POS	POS	NEG	POS	NEG	POS	POS	NEG	NEG	NEG	NEG
ST72-MRSA-IV [PVL+], WA-44	POS	POS	NEG	NEG	POS	POS	POS	POS	POS	NEG	POS	NEG	POS	POS	NEG	NEG	NEG	NEG
CC75-MRSA-IV	POS	POS	NEG	NEG	NEG	NEG	NEG	POS	AMB	NEG	NEG	POS	NEG	NEG	NEG	NEG	NEG	NEG
ST883-MRSA-IV, WA-47	AMB	AMB	NEG	NEG	POS	AMB	NEG	AMB	POS	NEG	NEG	AMB	AMB	AMB	NEG	NEG	NEG	NEG
ST1303-MRSA-IV	POS	POS	NEG	NEG	POS	AMB	NEG	NEG	NEG	NEG	NEG	NEG	NEG	NEG	NEG	NEG	NEG	NEG
CC80-MRSA-IV	POS	POS	NEG	POS	NEG	NEG	POS	POS	POS	NEG	NEG	POS	POS	POS	NEG	POS	NEG	NEG
CC80-MRSA-IV [PVL+], European Clone	POS	POS	NEG	POS	NEG	NEG	POS	COMM	COMM	NEG	NEG	COMM	POS	POS	NEG	POS	NEG	NEG
CC88-MRSA-IV, WA-2	POS	POS	NEG	NEG	POS	POS	POS	POS	POS	NEG	NEG	POS	POS	POS	NEG	NEG	POS	NEG
CC88-MRSA-IV [PVL+]	POS	POS	NEG	NEG	POS	AMB	POS	POS	POS	NEG	NEG	POS	POS	POS	NEG	NEG	POS	NEG
CC88-MRSA-V [PVL+]	POS	POS	NEG	NEG	POS	POS	POS	POS	POS	NEG	NEG	POS	POS	POS	NEG	NEG	POS	NEG
CC88-MRSA-VI	POS	POS	NEG	NEG	POS	POS	POS	POS	POS	NEG	NEG	POS	POS	POS	NEG	NEG	POS	NEG
ST93-MRSA-IV [PVL-]	POS	POS	NEG	POS	NEG	NEG	NEG	POS	AMB	NEG	NEG	POS	POS	POS	NEG	NEG	NEG	NEG
ST93-MRSA-IV [PVL+], Queensland Clone	POS	POS	NEG	POS	NEG	NEG	NEG	POS	POS	NEG	NEG	POS	POS	POS	NEG	NEG	NEG	NEG
ST93-MRSA-V [PVL+]	POS	POS	NEG	POS	NEG	NEG	NEG	POS	POS	NEG	NEG	POS	POS	POS	NEG	NEG	NEG	NEG
CC97-MRSA-IV, WA-54	POS	POS	NEG	NEG	POS	AMB	POS	VAR	VAR	NEG	VAR	NEG	POS	POS	NEG	NEG	NEG	POS
CC97-MRSA-V	POS	POS	NEG	NEG	POS	POS	POS	POS	POS	NEG	POS	NEG	POS	POS	NEG	NEG	NEG	POS
CC97-MRSA-V [ACME+]	POS	POS	NEG	NEG	POS	POS	POS	POS	POS	NEG	POS	NEG	POS	POS	NEG	NEG	NEG	POS
CC97-MRSA-I/V	POS	POS	NEG	NEG	POS	AMB	POS	POS	POS	NEG	POS	NEG	POS	POS	NEG	NEG	NEG	POS
CC121-MRSA-V, WA-22	POS	POS	NEG	POS	NEG	NEG	NEG	POS	POS	NEG	NEG	NEG	POS	POS	POS	NEG	NEG	NEG
CC152-MRSA-V [PVL+]	NEG	NEG	NEG	NEG	NEG	NEG	NEG	POS	POS	POS	NEG	NEG	POS	POS	NEG	NEG	NEG	NEG
ST154-MRSA [PVL+]	POS	POS	NEG	NEG	POS	AMB	NEG	POS	POS	NEG	NEG	POS	POS	POS	POS	NEG	NEG	NEG
ST188-MRSA-IV, WA-38/78	POS	POS	NEG	POS	NEG	AMB	NEG	POS	POS	NEG	POS	NEG	POS	POS	NEG	POS	NEG	NEG
ST188-MRSA-V	POS	POS	NEG	POS	NEG	AMB	NEG	POS	POS	NEG	POS	NEG	POS	POS	NEG	POS	NEG	NEG
ST239-MRSA-III	POS	POS	NEG	POS	NEG	NEG	POS	POS	POS	POS	NEG	NEG	POS	POS	POS	NEG	NEG	NEG
CC361-MRSA-IV, WA-29	POS	POS	NEG	POS	NEG	NEG	POS	POS	AMB	NEG	NEG	POS	POS	POS	NEG	NEG	POS	NEG
CC361-MRSA-V	POS	POS	NEG	POS	NEG	NEG	POS	POS	POS	NEG	NEG	POS	POS	POS	NEG	NEG	POS	NEG
CC361-MRSA-VIII, WA-28	POS	POS	NEG	POS	NEG	NEG	POS	POS	NEG	NEG	NEG	POS	POS	POS	NEG	NEG	POS	NEG
CC398-MRSA-IV	POS	POS	NEG	NEG	AMB	AMB	NEG	POS	POS	NEG	NEG	NEG	POS	POS	NEG	NEG	NEG	NEG
ST398-MRSA-V	POS	POS	NEG	NEG	AMB	AMB	NEG	POS	POS	NEG	NEG	NEG	POS	POS	NEG	NEG	NEG	NEG
ST398-MRSA-V [PVL+]	POS	POS	NEG	NEG	NEG	NEG	NEG	POS	POS	NEG	NEG	NEG	POS	POS	NEG	NEG	NEG	NEG
CC509/ST207-MRSA-V	POS	POS	NEG	POS	NEG	NEG	POS	NEG	NEG	NEG	NEG	NEG	POS	POS	NEG	NEG	NEG	NEG
ST573-MRSA-V, WA-10	POS	POS	NEG	NEG	POS	NEG	POS	POS	POS	NEG	NEG	POS	POS	POS	NEG	NEG	NEG	NEG
ST772-MRSA-V [PVL+], WA-60/Bengal Bay Clone	POS	POS	NEG	NEG	POS	AMB	POS	POS	POS	NEG	NEG	POS	POS	POS	NEG	NEG	NEG	NEG
ST779-MRSA	POS	POS	NEG	POS	NEG	NEG	POS	AMB	AMB	AMB	NEG	NEG	POS	POS	POS	NEG	NEG	NEG
ST834-MRSA-IV, WA-13	POS	POS	NEG	NEG	POS	AMB	POS	POS	POS	NEG	NEG	POS	POS	POS	POS	NEG	NEG	NEG
CC913-MRSA-IV	POS	POS	NEG	NEG	POS	NEG	POS	POS	POS	NEG	NEG	POS	POS	POS	NEG	NEG	NEG	NEG
ST1774-MRSA-IV [ACME+]	POS	POS	NEG	POS	NEG	NEG	NEG	POS	POS	NEG	POS	NEG	POS	POS	NEG	POS	NEG	NEG
CC15-MSSA	POS	POS	NEG	POS	NEG	NEG	POS	COMM	COMM	NEG	COMM	NEG	POS	POS	NEG	NEG	NEG	NEG
ST426-MSSA	POS	POS	POS	NEG	NEG	AMB	NEG	POS	POS	NEG	NEG	POS	POS	POS	POS	POS	NEG	NEG

Legend:

Positive :	POS
Ambiguous :	AMB
Negative :	NEG
Rare (in >0 to 33% of isolates) :	RARE
Variable (in >33 to 66% of isolates) :	VAR
Common (in >66 to <100% of isolates) :	COMM

	TYPE I RESTRICTION-MODIFICATION SYSTEM, SINGLE SEQUENCE SPECIFICITY PROTEIN												
	hsdS1 (RF122)	hsdS2 (ST5+ST8)	hsdS2 (MW2+476)	hsdS2 (RF122)	hsdS2 (MRSA252)	hsdS3 (all other than RF122+ MRSA252)	hsdS3 (ST8+ST1+ RF122)	hsdS3 (Mu50+ N315)	hsdS3 (CC51+ MRSA252)	hsdS3 (MRSA252)	hsdSx (CC25)	hsdSx (CC15)	hsdSx (etd)
CC1-MRSA-IV [PVL-], WA-1/57	NEG	NEG	POS	NEG	NEG	POS	POS	NEG	NEG	NEG	POS	NEG	NEG
CC1-MRSA-IV [PVL+], USA400	NEG	NEG	POS	NEG	NEG	POS	POS	NEG	NEG	NEG	POS	NEG	NEG
CC1-MRSA-IV/SCCfus [PVL-]	NEG	NEG	POS	NEG	NEG	POS	POS	NEG	NEG	NEG	POS	NEG	NEG
CC1-MRSA-IV/SCCfus [PVL+]	NEG	NEG	AMB	NEG	NEG	POS	POS	NEG	NEG	NEG	POS	NEG	NEG
CC1-MRSA-V [PVL+]	NEG	NEG	POS	NEG	NEG	POS	POS	NEG	NEG	NEG	POS	NEG	NEG
CC1-MRSA-V/SCCfus [PVL-]	NEG	NEG	POS	NEG	NEG	POS	POS	NEG	NEG	NEG	POS	NEG	NEG
CC1-MRSA-V/SCCfus [PVL+]	NEG	NEG	POS	NEG	NEG	POS	POS	NEG	NEG	NEG	POS	NEG	NEG
ST228-MRSA-I, South German/Italian MRSA	NEG	NEG	NEG	NEG	NEG	POS	NEG	POS	NEG	NEG	POS	NEG	NEG
ST5-MRSA-I, Geraldine Clone	NEG	POS	AMB	NEG	NEG	POS	NEG	POS	NEG	NEG	POS	NEG	NEG
CC5-MRSA-Ivar., WA-18/21/48	NEG	POS	NEG	NEG	NEG	POS	NEG	POS	NEG	NEG	POS	NEG	NEG
CC5-MRSA-II, Rhine-Hesse /UK-3	NEG	POS	NEG	NEG	NEG	POS	NEG	POS	NEG	NEG	POS	NEG	NEG
CC5-MRSA-II [ACME+]	NEG	POS	AMB	NEG	NEG	POS	NEG	NEG	NEG	NEG	POS	AMB	NEG
CC5-MRSA-III	NEG	POS	AMB	NEG	NEG	POS	NEG	POS	NEG	NEG	POS	NEG	NEG
CC5-MRSA-IV, Paediatric clone	NEG	POS	NEG	NEG	NEG	POS	NEG	POS	NEG	NEG	POS	NEG	NEG
CC5-MRSA-IV [PVL+]	NEG	POS	NEG	NEG	NEG	POS	NEG	POS	NEG	NEG	POS	NEG	NEG
CC5-MRSA-V	NEG	POS	NEG	NEG	NEG	POS	NEG	POS	NEG	NEG	POS	NEG	NEG
CC5-MRSA-V [PVL+]	NEG	NEG	NEG	NEG	NEG	POS	NEG	POS	NEG	NEG	POS	NEG	NEG
CC5-MRSA-VI, New Paediatric Clone	NEG	POS	NEG	NEG	NEG	POS	NEG	POS	NEG	NEG	POS	NEG	NEG
ST5-MRSA-VII (SCC-JCSC6082)	NEG	NEG	NEG	NEG	NEG	POS	NEG	POS	NEG	NEG	POS	NEG	NEG
CC5-MRSA-IVvar, Maltese Clone	NEG	POS	NEG	NEG	NEG	POS	NEG	POS	NEG	NEG	POS	NEG	NEG
CC5-MRSA-SCC(MRSAZH47)	NEG	POS	NEG	NEG	NEG	POS	NEG	POS	NEG	NEG	POS	NEG	NEG
CC5-MRSA-IV/VI	NEG	POS	NEG	NEG	NEG	POS	NEG	POS	NEG	NEG	POS	NEG	NEG
CC5-MRSA-IV/SCCfus	NEG	POS	NEG	NEG	NEG	POS	NEG	POS	NEG	NEG	POS	NEG	NEG
CC5/ST835-MRSA, WA-40/46	NEG	POS	NEG	NEG	NEG	POS	NEG	POS	NEG	NEG	POS	NEG	NEG
ST6-MRSA-IV, WA-51	NEG	NEG	NEG	NEG	NEG	POS	POS	NEG	POS	NEG	POS	NEG	NEG
ST6-MRSA-V	NEG	NEG	POS	NEG	NEG	POS	POS	NEG	POS	NEG	POS	POS	NEG
CC7-MRSA-IV	NEG	NEG	NEG	NEG	NEG	NEG	NEG	NEG	NEG	NEG	AMB	NEG	AMB
CC7-MRSA-V	NEG	NEG	NEG	NEG	NEG	NEG	NEG	NEG	NEG	NEG	AMB	NEG	AMB
ST250-MRSA-I, Early/Ancestral MRSA	NEG	POS	NEG	NEG	NEG	POS	POS	NEG	NEG	NEG	POS	NEG	NEG
ST247-MRSA-I, North German/Iberian MRSA	NEG	POS	NEG	NEG	NEG	POS	POS	NEG	NEG	NEG	POS	NEG	NEG
CC8-MRSA-IV, UK-14/WA-5	NEG	POS	NEG	NEG	NEG	POS	POS	NEG	NEG	NEG	POS	NEG	NEG
CC8-MRSA-IV, Lyon Clone/UK-2	NEG	POS	AMB	NEG	NEG	POS	POS	NEG	NEG	NEG	POS	NEG	NEG
CC8-MRSA-IV, UK-6	NEG	POS	NEG	NEG	NEG	POS	POS	NEG	NEG	NEG	POS	NEG	NEG
CC8-MRSA-IV, USA500	NEG	POS	NEG	NEG	NEG	POS	POS	NEG	NEG	NEG	POS	NEG	NEG
ST8-MRSA-IV [PVL/ACME+], USA300	NEG	POS	NEG	NEG	NEG	POS	POS	NEG	NEG	NEG	POS	NEG	NEG
ST8-MRSA-IV [PVL+], USA300	NEG	POS	NEG	NEG	NEG	POS	POS	NEG	NEG	NEG	POS	NEG	NEG
CC8-MRSA-IV [PVL/sed/j/r/k/q+]	NEG	POS	NEG	NEG	NEG	AMB	NEG	NEG	NEG	NEG	POS	NEG	NEG
CC8-MRSA-IV [tst1/sec/sel+]	NEG	POS	NEG	NEG	NEG	POS	POS	NEG	NEG	NEG	POS	NEG	NEG
CC8-MRSA-V	NEG	POS	AMB	NEG	NEG	POS	POS	NEG	NEG	NEG	POS	NEG	NEG
CC8-MRSA-VIII	NEG	POS	NEG	NEG	NEG	AMB	AMB	NEG	NEG	NEG	POS	NEG	NEG
ST8-MRSA-IIA/B/C/D/E, Irish AR13/14	NEG	POS	NEG	NEG	NEG	AMB	AMB	NEG	NEG	NEG	POS	NEG	NEG
ST8-MRSA, UK-12/13, Irish AR43	NEG	POS	NEG	NEG	NEG	POS	POS	NEG	NEG	NEG	POS	NEG	NEG
ST254-MRSA, Hannover EMRSA	NEG	POS	NEG	NEG	NEG	POS	POS	NEG	NEG	NEG	POS	NEG	NEG
ST254-MRSA-IV/V, Hannover/UK-10	NEG	POS	NEG	NEG	NEG	POS	POS	NEG	NEG	NEG	POS	NEG	NEG
CC9-MRSA-IV	NEG	NEG	POS	NEG	NEG	NEG	NEG	NEG	NEG	NEG	NEG	NEG	NEG
CC12-MRSA, WA-59	NEG	POS	NEG	NEG	NEG	NEG	NEG	NEG	POS	NEG	POS	NEG	NEG
CC12-MRSA-IV	NEG	POS	NEG	NEG	NEG	AMB	NEG	NEG	POS	NEG	POS	NEG	NEG
CC20-MRSA-V	NEG	NEG	AMB	NEG	NEG	NEG	NEG	NEG	NEG	NEG	NEG	NEG	NEG
ST22-MRSA-IV, Barnim/UK-15	NEG	POS	NEG	NEG	NEG	AMB	NEG	NEG	NEG	NEG	POS	NEG	NEG
ST22-MRSA-IV [ACME+]	NEG	POS	AMB	NEG	NEG	AMB	NEG	NEG	NEG	NEG	POS	AMB	NEG
ST22-MRSA-IV [PVL+]	NEG	POS	NEG	NEG	NEG	POS	NEG	NEG	NEG	NEG	POS	NEG	NEG
ST22-MRSA-V	NEG	POS	NEG	NEG	NEG	AMB	NEG	NEG	NEG	NEG	POS	NEG	NEG
ST22-MRSA-V [PVL+]	NEG	POS	NEG	NEG	NEG	AMB	NEG	NEG	NEG	NEG	POS	NEG	NEG
ST36-MRSA-II, UK-16	NEG	NEG	AMB	NEG	POS	NEG	NEG	NEG	POS	POS	POS	NEG	NEG
ST30-MRSA-IV [PVL+], Southwest Pacific Clone	NEG	NEG	NEG	NEG	POS	AMB	NEG	NEG	POS	POS	POS	AMB	NEG
ST30-MRSA-IV [tst1+]	NEG	NEG	NEG	NEG	POS	NEG	NEG	NEG	POS	POS	POS	NEG	NEG
CC30-MRSA-V [PVL+]	NEG	NEG	NEG	NEG	POS	NEG	NEG	NEG	POS	POS	POS	NEG	NEG
ST45-MRSA-II, USA600	NEG	NEG	NEG	NEG	POS	NEG	NEG	NEG	NEG	NEG	AMB	NEG	NEG
ST45-MRSA-IV, Berlin EMRSA	NEG	NEG	NEG	NEG	POS	NEG	NEG	NEG	NEG	NEG	AMB	NEG	NEG
CC45-MRSA-IV [ACME+]	NEG	NEG	NEG	NEG	POS	NEG	NEG	NEG	NEG	NEG	POS	AMB	NEG
ST45-MRSA-V, WA-4	NEG	NEG	NEG	NEG	POS	NEG	NEG	NEG	NEG	NEG	AMB	NEG	NEG
CC45/agr IV-MRSA-IV, WA-23	NEG	NEG	NEG	NEG	POS	POS	NEG	NEG	POS	NEG	AMB	NEG	NEG
CC45/agr IV-MRSA-V	NEG	NEG	AMB	NEG	AMB	POS	NEG	NEG	POS	NEG	POS	AMB	NEG
CC45/agr IV-MRSA-IV/V	NEG	NEG	AMB	NEG	POS	POS	NEG	NEG	POS	NEG	POS	AMB	NEG
CC59-MRSA-IV [PVL+], USA1000	NEG	NEG	NEG	NEG	NEG	NEG	NEG	NEG	POS	NEG	NEG	NEG	POS
ST59-MRSA-IV, WA-73	NEG	NEG	AMB	NEG	NEG	NEG	NEG	NEG	POS	NEG	NEG	AMB	POS
ST87-MRSA-IV, WA-24	NEG	NEG	NEG	NEG	NEG	NEG	NEG	NEG	POS	NEG	AMB	NEG	POS
ST59-MRSA-IV [PVL+], WA-55/56	NEG	NEG	AMB	NEG	NEG	AMB	NEG	NEG	POS	NEG	AMB	AMB	POS
ST59/ST952-MRSA-V(T) [PVL+], Taiwan Clone	NEG	NEG	NEG	NEG	NEG	AMB	NEG	NEG	POS	NEG	AMB	AMB	POS
ST59-MRSA-V	NEG	NEG	NEG	NEG	NEG	NEG	NEG	NEG	POS	NEG	NEG	NEG	POS
CC59-MRSA-V [PVL+]	NEG	NEG	NEG	NEG	NEG	NEG	NEG	NEG	POS	NEG	NEG	NEG	POS
ST59-MRSA-IV/V, WA-15	NEG	NEG	NEG	NEG	NEG	AMB	NEG	NEG	POS	NEG	AMB	NEG	POS
ST72-MRSA-IV, USA700	NEG	NEG	NEG	NEG	NEG	AMB	NEG	NEG	POS	NEG	POS	NEG	NEG
ST72-MRSA-IV [PVL+], WA-44	NEG	NEG	NEG	NEG	NEG	AMB	NEG	NEG	POS	NEG	POS	NEG	NEG
CC75-MRSA-IV	NEG	NEG	NEG	POS	NEG	NEG	NEG	NEG	NEG	NEG	AMB	NEG	NEG
ST883-MRSA-IV, WA-47	POS	AMB	NEG	NEG	NEG	AMB	NEG	NEG	NEG	NEG	AMB	NEG	NEG
ST1303-MRSA-IV	NEG	NEG	NEG	NEG	NEG	NEG	NEG	NEG	NEG	NEG	NEG	NEG	POS
CC80-MRSA-IV	NEG	NEG	AMB	NEG	NEG	NEG	NEG	NEG	NEG	NEG	NEG	NEG	POS
CC80-MRSA-IV [PVL+], European Clone	NEG	NEG	NEG	NEG	NEG	NEG	NEG	NEG	NEG	NEG	NEG	NEG	POS
CC88-MRSA-IV, WA-2	POS	NEG	NEG	NEG	NEG	POS	NEG	NEG	NEG	POS	POS	NEG	NEG
CC88-MRSA-IV [PVL+]	AMB	NEG	NEG	NEG	NEG	POS	NEG	NEG	NEG	POS	AMB	NEG	NEG
CC88-MRSA-V [PVL+]	POS	NEG	NEG	NEG	NEG	POS	NEG	NEG	NEG	POS	POS	NEG	NEG
CC88-MRSA-VI	POS	NEG	NEG	NEG	NEG	POS	NEG	NEG	NEG	POS	POS	AMB	NEG
ST93-MRSA-IV [PVL-]	NEG	NEG	NEG	NEG	NEG	NEG	NEG	NEG	NEG	POS	NEG	NEG	NEG
ST93-MRSA-IV [PVL+], Queensland Clone	NEG	NEG	AMB	NEG	NEG	NEG	NEG	NEG	NEG	POS	NEG	NEG	NEG
ST93-MRSA-V [PVL+]	NEG	NEG	AMB	NEG	NEG	NEG	NEG	NEG	NEG	POS	NEG	NEG	NEG
CC97-MRSA-IV, WA-54	NEG	NEG	NEG	NEG	NEG	NEG	NEG	NEG	NEG	NEG	POS	NEG	NEG
CC97-MRSA-V	NEG	NEG	NEG	NEG	NEG	NEG	NEG	NEG	NEG	NEG	POS	NEG	NEG
CC97-MRSA-V [ACME+]	NEG	NEG	POS	NEG	NEG	AMB	NEG	NEG	NEG	NEG	POS	AMB	AMB
CC97-MRSA-I/V	NEG	NEG	NEG	NEG	NEG	AMB	NEG	NEG	NEG	NEG	POS	NEG	NEG
CC121-MRSA-V, WA-22	NEG	POS	NEG	NEG	NEG	NEG	NEG	NEG	POS	NEG	POS	NEG	NEG
CC152-MRSA-V [PVL+]	NEG	POS	NEG	NEG	NEG	AMB	NEG	NEG	POS	POS	POS	NEG	POS
ST154-MRSA [PVL+]	NEG	NEG	NEG	NEG	NEG	NEG	NEG	NEG	NEG	NEG	NEG	NEG	POS
ST188-MRSA-IV, WA-38/78	NEG	NEG	POS	NEG	NEG	POS	NEG	NEG	POS	NEG	POS	AMB	NEG
ST188-MRSA-V	NEG	NEG	POS	NEG	NEG	POS	NEG	NEG	POS	NEG	POS	POS	NEG
ST239-MRSA-III	NEG	POS	AMB	NEG	NEG	POS	POS	NEG	NEG	NEG	POS	NEG	NEG
CC361-MRSA-IV, WA-29	NEG	NEG	POS	NEG	NEG	NEG	NEG	NEG	NEG	NEG	NEG	NEG	NEG
CC361-MRSA-V	NEG	NEG	POS	NEG	NEG	NEG	NEG	NEG	NEG	NEG	NEG	NEG	NEG
CC361-MRSA-VIII, WA-28	NEG	NEG	NEG	NEG	NEG	NEG	NEG	NEG	NEG	NEG	NEG	NEG	NEG
CC398-MRSA-IV	NEG	NEG	AMB	NEG	NEG	NEG	NEG	NEG	NEG	NEG	NEG	POS	NEG
ST398-MRSA-V	NEG	NEG	NEG	NEG	NEG	NEG	NEG	NEG	NEG	NEG	NEG	POS	NEG
ST398-MRSA-V [PVL+]	NEG	NEG	NEG	NEG	NEG	NEG	NEG	NEG	NEG	NEG	NEG	POS	NEG
CC509/ST207-MRSA-V	NEG	NEG	NEG	NEG	NEG	NEG	NEG	NEG	NEG	NEG	NEG	NEG	NEG
ST573-MRSA-V, WA-10	NEG	NEG	NEG	NEG	NEG	NEG	NEG	NEG	NEG	NEG	NEG	NEG	NEG
ST772-MRSA-V [PVL+], WA-60/Bengal Bay Clone	NEG	NEG	NEG	NEG	NEG	NEG	NEG	NEG	NEG	NEG	NEG	NEG	NEG
ST779-MRSA	NEG	NEG	AMB	NEG	NEG	NEG	NEG	NEG	POS	NEG	AMB	NEG	POS
ST834-MRSA-IV, WA-13	NEG	NEG	AMB	NEG	NEG	AMB	NEG	NEG	NEG	NEG	NEG	NEG	NEG
CC913-MRSA-IV	NEG	NEG	NEG	NEG	NEG	NEG	NEG	NEG	NEG	NEG	NEG	NEG	NEG
ST1774-MRSA-IV [ACME+]	NEG	NEG	AMB	NEG	POS	POS	NEG	NEG	POS	NEG	POS	AMB	NEG

CC15-MSSA	NEG	NEG	NEG	NEG	NEG	NEG	NEG	NEG	NEG	NEG	AMB	POS	NEG
ST426-MSSA	AMB	NEG	NEG	NEG	NEG	NEG	NEG	NEG	NEG	POS	AMB	NEG	NEG

Legend:

Positive :	POS
Ambiguous :	AMB
Negative :	NEG
Rare (in >0 to 33% of isolates) :	RARE
Variable (in >33 to 66% of isolates) :	VAR
Common (in >66 to <100% of isolates) :	COMM

	HYALURONATE LYASE							IMMUNODOMINANT ANTIGEN B		DEFENSIN RESISTANCE		TRANSFERRIN BINDING PROTEIN			PUTATIVE TRANSPORTER		MISCELLANEOUS GENES		
	hysA1 (MRSA252)	hysA1 (MRSA252+RF122) and/or hysA2 (cons)	hysA1 (MRSA252+RF122) and/or hysA2 (COL+USA300)	hysA2 (Other than MRSA252)	hysA2 (COL+USA300+NCTC8325)	hysA2 (Other than COL+USA300+NCTC8325)	hysA2 (MRSA252)	isaB	isaB (MRSA252)	mprF (COL+MW2)	mprF (Mu50+MRSA252)	isdA (cons)	isdA (MRSA252)	isdA (Other than MRSA252)	lmrP (Other than RF122)	lmrP (RF122)	Q2FXC0	Q2YUB3	Q7A4X2
CC1-MRSA-IV [PVL-], WA-1/57	NEG	POS	NEG	POS	NEG	POS	NEG	POS	AMB	POS	AMB	POS	NEG	POS	POS	NEG	POS	NEG	NEG
CC1-MRSA-IV [PVL+], USA400	NEG	POS	NEG	POS	NEG	POS	NEG	POS	AMB	POS	AMB	POS	NEG	POS	POS	NEG	POS	NEG	NEG
CC1-MRSA-IV/SCCfus [PVL-]	NEG	POS	NEG	POS	NEG	POS	NEG	POS	AMB	POS	AMB	POS	NEG	POS	POS	NEG	POS	NEG	NEG
CC1-MRSA-IV/SCCfus [PVL+]	NEG	POS	NEG	POS	NEG	POS	NEG	POS	AMB	POS	AMB	POS	NEG	POS	POS	NEG	AMB	NEG	NEG
CC1-MRSA-V [PVL+]	NEG	POS	NEG	POS	NEG	POS	AMB	AMB	POS	POS	AMB	POS	NEG	POS	POS	NEG	POS	NEG	NEG
CC1-MRSA-V/SCCfus [PVL-]	NEG	POS	NEG	POS	NEG	POS	AMB	AMB	POS	POS	AMB	POS	NEG	POS	POS	NEG	POS	NEG	NEG
CC1-MRSA-V/SCCfus [PVL+]	NEG	POS	NEG	POS	NEG	POS	AMB	AMB	POS	POS	AMB	POS	NEG	POS	POS	NEG	POS	NEG	NEG
ST228-MRSA-I, South German/Italian MRSA	NEG	POS	NEG	AMB	NEG	POS	NEG	POS	AMB	AMB	POS	POS	NEG	POS	POS	NEG	NEG	NEG	COMM
ST5-MRSA-I, Geraldine Clone	NEG	POS	NEG	POS	NEG	POS	NEG	POS	AMB	AMB	POS	POS	NEG	POS	POS	NEG	NEG	NEG	POS
CC5-MRSA-Ivar., WA-18/21/48	NEG	POS	NEG	POS	NEG	POS	NEG	POS	AMB	AMB	POS	POS	NEG	POS	POS	NEG	NEG	NEG	POS
CC5-MRSA-II, Rhine-Hesse /UK-3	NEG	POS	NEG	POS	NEG	POS	NEG	POS	AMB	AMB	POS	POS	NEG	POS	POS	NEG	NEG	NEG	POS
CC5-MRSA-II [ACME+]	NEG	POS	NEG	POS	NEG	POS	NEG	POS	AMB	AMB	POS	POS	AMB	POS	POS	NEG	NEG	AMB	POS
CC5-MRSA-III	NEG	POS	NEG	POS	NEG	POS	NEG	POS	AMB	AMB	POS	POS	NEG	POS	POS	NEG	NEG	NEG	POS
CC5-MRSA-IV, Paediatric clone	NEG	POS	NEG	POS	NEG	POS	NEG	POS	AMB	AMB	POS	POS	NEG	POS	POS	NEG	NEG	NEG	POS
CC5-MRSA-IV [PVL+]	NEG	POS	NEG	AMB	NEG	POS	NEG	POS	AMB	AMB	POS	POS	NEG	POS	POS	NEG	NEG	NEG	POS
CC5-MRSA-V	NEG	POS	NEG	POS	NEG	POS	NEG	POS	AMB	AMB	POS	POS	NEG	POS	POS	NEG	NEG	NEG	POS
CC5-MRSA-V [PVL+]	NEG	POS	NEG	POS	NEG	POS	NEG	POS	AMB	AMB	POS	POS	NEG	POS	POS	NEG	NEG	NEG	POS
CC5-MRSA-VI, New Paediatric Clone	NEG	POS	NEG	POS	NEG	POS	NEG	POS	AMB	AMB	POS	POS	NEG	POS	POS	NEG	NEG	NEG	POS
ST5-MRSA-VII (SCC-JCSC6082)	NEG	POS	NEG	POS	NEG	POS	NEG	POS	AMB	NEG	POS	POS	NEG	POS	POS	NEG	NEG	NEG	POS
CC5-MRSA-IVvar, Maltese Clone	NEG	POS	NEG	POS	NEG	POS	NEG	POS	AMB	AMB	POS	POS	NEG	POS	POS	NEG	NEG	NEG	AMB
CC5-MRSA-SCC(MRSAZH47)	NEG	POS	NEG	POS	NEG	POS	NEG	POS	AMB	AMB	POS	POS	NEG	POS	POS	NEG	NEG	NEG	POS
CC5-MRSA-IV/VI	NEG	POS	NEG	AMB	NEG	POS	NEG	POS	AMB	AMB	POS	POS	NEG	POS	POS	NEG	NEG	NEG	POS
CC5-MRSA-IV/SCCfus	NEG	POS	NEG	AMB	NEG	POS	NEG	POS	AMB	AMB	POS	POS	NEG	POS	POS	NEG	NEG	NEG	POS
CC5/ST835-MRSA, WA-40/46	NEG	POS	NEG	POS	NEG	POS	NEG	POS	AMB	AMB	POS	POS	NEG	POS	POS	NEG	NEG	NEG	POS
ST6-MRSA-IV, WA-51	NEG	POS	NEG	NEG	NEG	POS	NEG	POS	AMB	POS	AMB	POS	NEG	POS	POS	NEG	AMB	NEG	NEG
ST6-MRSA-V	NEG	POS	NEG	NEG	NEG	POS	NEG	POS	AMB	POS	AMB	POS	AMB	POS	POS	NEG	POS	AMB	NEG
CC7-MRSA-IV	NEG	POS	NEG	NEG	NEG	POS	NEG	POS	AMB	POS	AMB	POS	NEG	POS	POS	NEG	NEG	NEG	POS
CC7-MRSA-V	NEG	POS	NEG	NEG	NEG	POS	NEG	POS	AMB	POS	AMB	POS	NEG	POS	POS	NEG	NEG	NEG	POS
ST250-MRSA-I, Early/Ancestral MRSA	NEG	POS	POS	POS	POS	NEG	NEG	POS	AMB	POS	AMB	POS	NEG	POS	POS	NEG	POS	NEG	NEG
ST247-MRSA-I, North German/Iberian MRSA	NEG	POS	POS	POS	POS	NEG	NEG	POS	AMB	POS	AMB	POS	NEG	POS	POS	NEG	POS	NEG	NEG
CC8-MRSA-IV, UK-14/WA-5	NEG	POS	POS	POS	POS	NEG	NEG	POS	AMB	POS	AMB	POS	NEG	POS	POS	NEG	POS	NEG	NEG
CC8-MRSA-IV, Lyon Clone/UK-2	NEG	POS	POS	POS	POS	NEG	NEG	POS	AMB	POS	AMB	POS	NEG	POS	POS	NEG	POS	NEG	NEG
CC8-MRSA-IV, UK-6	NEG	POS	POS	POS	POS	NEG	AMB	POS	AMB	POS	AMB	POS	NEG	POS	POS	NEG	POS	NEG	NEG
CC8-MRSA-IV, USA500	NEG	POS	POS	POS	POS	NEG	NEG	POS	AMB	POS	AMB	POS	NEG	POS	POS	NEG	POS	NEG	NEG
ST8-MRSA-IV [PVL/ACME+], USA300	NEG	POS	POS	POS	POS	NEG	NEG	POS	AMB	POS	AMB	POS	NEG	POS	POS	NEG	POS	NEG	NEG
ST8-MRSA-IV [PVL+], USA300	NEG	POS	POS	POS	POS	NEG	NEG	POS	AMB	POS	NEG	POS	NEG	POS	POS	NEG	POS	NEG	NEG
CC8-MRSA-IV [PVL/sed/j/r/k/q+]	NEG	POS	POS	POS	POS	NEG	NEG	POS	AMB	POS	AMB	POS	AMB	POS	POS	NEG	NEG	NEG	NEG
CC8-MRSA-IV [tst1/sec/sel+]	NEG	POS	POS	POS	POS	NEG	NEG	POS	AMB	POS	AMB	POS	AMB	POS	POS	NEG	POS	NEG	NEG
CC8-MRSA-V	NEG	POS	POS	AMB	POS	NEG	NEG	POS	AMB	POS	AMB	POS	NEG	POS	POS	NEG	AMB	NEG	NEG
CC8-MRSA-VIII	NEG	POS	POS	AMB	POS	NEG	NEG	POS	AMB	POS	NEG	POS	NEG	POS	POS	NEG	AMB	NEG	NEG
ST8-MRSA-IIA/B/C/D/E, Irish AR13/14	NEG	POS	POS	AMB	POS	NEG	NEG	POS	AMB	POS	AMB	POS	NEG	POS	POS	NEG	AMB	NEG	NEG
ST8-MRSA, UK-12/13, Irish AR43	NEG	POS	POS	POS	POS	NEG	AMB	POS	AMB	POS	AMB	POS	NEG	POS	POS	NEG	POS	NEG	NEG
ST254-MRSA, Hannover EMRSA	NEG	POS	POS	POS	POS	NEG	NEG	POS	AMB	POS	AMB	POS	NEG	POS	POS	NEG	POS	NEG	NEG
ST254-MRSA-IV/V, Hannover/UK-10	NEG	POS	POS	POS	POS	NEG	NEG	POS	AMB	POS	AMB	POS	NEG	POS	POS	NEG	POS	NEG	NEG
CC9-MRSA-IV	NEG	POS	NEG	NEG	NEG	POS	NEG	POS	AMB	AMB	POS	POS	NEG	POS	POS	NEG	NEG	NEG	POS
CC12-MRSA, WA-59	NEG	POS	NEG	NEG	NEG	POS	NEG	POS	AMB	POS	NEG	POS	NEG	POS	POS	NEG	NEG	NEG	NEG
CC12-MRSA-IV	NEG	POS	NEG	NEG	NEG	POS	NEG	POS	AMB	POS	AMB	POS	NEG	POS	POS	NEG	NEG	NEG	NEG
CC20-MRSA-V	NEG	POS	NEG	NEG	NEG	POS	NEG	POS	AMB	POS	AMB	POS	NEG	POS	POS	NEG	NEG	AMB	POS
ST22-MRSA-IV, Barnim/UK-15	NEG	POS	NEG	NEG	NEG	POS	AMB	NEG	POS	AMB	POS	POS	NEG	POS	POS	NEG	NEG	NEG	POS
ST22-MRSA-IV [ACME+]	NEG	POS	NEG	AMB	NEG	POS	POS	NEG	POS	AMB	POS	POS	NEG	POS	POS	NEG	NEG	AMB	POS
ST22-MRSA-IV [PVL+]	NEG	POS	NEG	NEG	NEG	POS	AMB	NEG	POS	AMB	POS	POS	NEG	POS	POS	NEG	NEG	AMB	POS
ST22-MRSA-V	NEG	POS	NEG	AMB	NEG	POS	AMB	NEG	POS	AMB	POS	POS	NEG	POS	POS	NEG	NEG	NEG	POS
ST22-MRSA-V [PVL+]	NEG	POS	NEG	NEG	NEG	POS	AMB	NEG	POS	AMB	POS	POS	NEG	POS	POS	NEG	NEG	NEG	POS
ST36-MRSA-II, UK-16	POS	POS	POS	NEG	POS	POS	POS	NEG	POS	AMB	POS	POS	POS	NEG	POS	NEG	NEG	NEG	POS
ST30-MRSA-IV [PVL+], Southwest Pacific Clone	POS	POS	POS	NEG	POS	POS	POS	NEG	POS	AMB	POS	POS	POS	NEG	POS	NEG	NEG	NEG	POS
ST30-MRSA-IV [tst1+]	POS	POS	POS	NEG	POS	POS	AMB	NEG	POS	AMB	POS	POS	POS	NEG	POS	NEG	NEG	NEG	AMB
CC30-MRSA-V [PVL+]	POS	POS	POS	NEG	POS	POS	POS	NEG	POS	AMB	POS	POS	POS	NEG	POS	NEG	NEG	NEG	NEG
ST45-MRSA-II, USA600	NEG	POS	NEG	AMB	NEG	POS	NEG	NEG	POS	NEG	AMB	POS	POS	AMB	POS	NEG	NEG	NEG	POS
ST45-MRSA-IV, Berlin EMRSA	NEG	POS	NEG	NEG	NEG	AMB	NEG	NEG	POS	NEG	AMB	POS	POS	AMB	POS	NEG	NEG	NEG	COMM
CC45-MRSA-IV [ACME+]	NEG	POS	NEG	NEG	NEG	POS	NEG	NEG	POS	AMB	POS	POS	POS	POS	POS	NEG	NEG	NEG	POS
ST45-MRSA-V, WA-4	NEG	POS	NEG	AMB	NEG	POS	NEG	NEG	POS	NEG	POS	POS	POS	POS	POS	NEG	NEG	NEG	POS
CC45/agr IV-MRSA-IV, WA-23	NEG	POS	POS	NEG	POS	AMB	NEG	NEG	POS	NEG	AMB	POS	POS	AMB	NEG	POS	NEG	NEG	POS
CC45/agr IV-MRSA-V	NEG	POS	POS	AMB	POS	AMB	NEG	AMB	POS	AMB	POS	POS	POS	AMB	NEG	POS	NEG	AMB	POS
CC45/agr IV-MRSA-IV/V	NEG	POS	POS	AMB	POS	AMB	NEG	NEG	POS	NEG	POS	POS	POS	AMB	NEG	POS	NEG	AMB	POS
CC59-MRSA-IV [PVL+], USA1000	NEG	POS	NEG	AMB	NEG	POS	NEG	POS	NEG	POS	AMB	POS	NEG	POS	NEG	POS	NEG	NEG	NEG
ST59-MRSA-IV, WA-73	NEG	POS	NEG	NEG	NEG	POS	NEG	POS	AMB	POS	AMB	POS	NEG	POS	NEG	POS	NEG	AMB	NEG
ST87-MRSA-IV, WA-24	NEG	POS	NEG	AMB	NEG	POS	NEG	POS	NEG	POS	AMB	POS	NEG	POS	NEG	POS	NEG	NEG	NEG
ST59-MRSA-IV [PVL+], WA-55/56	NEG	POS	NEG	AMB	NEG	POS	NEG	POS	AMB	POS	AMB	POS	AMB	POS	NEG	POS	NEG	AMB	NEG
ST59/ST952-MRSA-V(T) [PVL+], Taiwan Clone	NEG	POS	NEG	AMB	NEG	POS	NEG	POS	NEG	POS	AMB	POS	AMB	POS	NEG	POS	NEG	AMB	NEG
ST59-MRSA-V	NEG	POS	NEG	AMB	NEG	POS	NEG	POS	NEG	POS	AMB	POS	NEG	POS	NEG	POS	NEG	NEG	NEG
CC59-MRSA-V [PVL+]	NEG	NEG	NEG	NEG	NEG	POS	NEG	POS	NEG	POS	NEG	POS	NEG	POS	NEG	POS	NEG	NEG	NEG
ST59-MRSA-IV/V, WA-15	NEG	POS	NEG	AMB	NEG	POS	NEG	POS	NEG	POS	AMB	POS	NEG	POS	NEG	POS	NEG	NEG	NEG
ST72-MRSA-IV, USA700	NEG	POS	NEG	POS	NEG	POS	NEG	POS	AMB	POS	AMB	POS	NEG	POS	POS	NEG	NEG	NEG	POS
ST72-MRSA-IV [PVL+], WA-44	NEG	POS	NEG	AMB	NEG	POS	AMB	POS	AMB	POS	AMB	POS	NEG	POS	POS	NEG	NEG	NEG	POS
CC75-MRSA-IV	AMB	POS	AMB	NEG	POS	NEG	NEG	NEG	AMB	AMB	NEG	NEG	NEG	NEG	NEG	NEG	NEG	AMB	POS
ST883-MRSA-IV, WA-47	NEG	AMB	NEG	NEG	NEG	POS	NEG	AMB	POS	POS	AMB	NEG	NEG	NEG	AMB	NEG	NEG	NEG	NEG
ST1303-MRSA-IV	NEG	POS	NEG	NEG	NEG	POS	NEG	NEG	AMB	POS	AMB	NEG	NEG	NEG	NEG	NEG	NEG	AMB	POS
CC80-MRSA-IV	NEG	POS	NEG	NEG	NEG	POS	NEG	POS	AMB	AMB	POS	POS	NEG	POS	POS	NEG	NEG	NEG	NEG
CC80-MRSA-IV [PVL+], European Clone	NEG	POS	NEG	AMB	NEG	POS	NEG	POS	AMB	AMB	POS	POS	NEG	POS	POS	NEG	NEG	NEG	NEG
CC88-MRSA-IV, WA-2	NEG	POS	NEG	POS	NEG	POS	NEG	POS	AMB	POS	AMB	POS	NEG	POS	POS	NEG	NEG	NEG	NEG
CC88-MRSA-IV [PVL+]	NEG	POS	NEG	POS	NEG	POS	NEG	POS	AMB	POS	AMB	POS	NEG	POS	POS	NEG	NEG	NEG	NEG
CC88-MRSA-V [PVL+]	NEG	POS	NEG	POS	NEG	POS	AMB	POS	AMB	POS	AMB	POS	NEG	POS	POS	NEG	NEG	NEG	NEG
CC88-MRSA-VI	NEG	POS	NEG	POS	NEG	POS	NEG	POS	AMB	POS	AMB	POS	NEG	POS	POS	NEG	NEG	AMB	NEG
ST93-MRSA-IV [PVL-]	NEG	POS	POS	NEG	POS	NEG	NEG	POS	AMB	NEG	NEG	POS	NEG	POS	POS	NEG	NEG	NEG	POS
ST93-MRSA-IV [PVL+], Queensland Clone	NEG	POS	POS	NEG	POS	NEG	AMB	POS	AMB	NEG	NEG	POS	NEG	POS	POS	NEG	NEG	NEG	POS
ST93-MRSA-V [PVL+]	NEG	POS	POS	NEG	POS	NEG	AMB	POS	AMB	NEG	NEG	POS	NEG	POS	POS	NEG	NEG	POS	POS
CC97-MRSA-IV, WA-54	NEG	POS	NEG	NEG	NEG	POS	NEG	POS	AMB	POS	NEG	POS	NEG	POS	POS	NEG	NEG	NEG	POS
CC97-MRSA-V	NEG	POS	NEG	NEG	NEG	POS	NEG	POS	AMB	POS	AMB	POS	NEG	POS	POS	NEG	NEG	NEG	NEG
CC97-MRSA-V [ACME+]	NEG	POS	NEG	NEG	NEG	POS	NEG	POS	AMB	POS	AMB	POS	NEG	POS	POS	NEG	NEG	AMB	AMB
CC97-MRSA-I/V	NEG	POS	NEG	NEG	NEG	POS	NEG	POS	AMB	POS	AMB	POS	NEG	POS	POS	NEG	NEG	AMB	POS
CC121-MRSA-V, WA-22	NEG	POS	POS	NEG	POS	NEG	NEG	POS	AMB	POS	AMB	POS	NEG	POS	NEG	POS	NEG	NEG	POS
CC152-MRSA-V [PVL+]	NEG	POS	AMB	NEG	POS	NEG	AMB	POS	AMB	POS	NEG	POS	NEG	NEG	NEG	POS	NEG	NEG	NEG
ST154-MRSA [PVL+]	NEG	POS	POS	NEG	POS	POS	NEG	POS	AMB	POS	AMB	POS	NEG	POS	POS	NEG	NEG	NEG	NEG
ST188-MRSA-IV, WA-38/78	NEG	POS	NEG	POS	NEG	POS	NEG	POS	AMB	POS	AMB	POS	AMB	POS	POS	NEG	NEG	NEG	NEG
ST188-MRSA-V	NEG	POS	NEG	POS	NEG	POS	AMB	POS	AMB	POS	AMB	POS	AMB	POS	POS	NEG	NEG	POS	NEG
ST239-MRSA-III	NEG	POS	POS	POS	POS	NEG	NEG	NEG	POS	POS	AMB	POS	NEG	POS	POS	NEG	POS	NEG	NEG
CC361-MRSA-IV, WA-29	NEG	POS	POS	POS	POS	NEG	NEG	POS	AMB	POS	AMB	POS	NEG	POS	POS	NEG	NEG	NEG	POS
CC361-MRSA-V	NEG	POS	POS	POS	POS	NEG	NEG	POS	AMB	POS	AMB	POS	NEG	POS	POS	NEG	NEG	NEG	POS
CC361-MRSA-VIII, WA-28	NEG	POS	AMB	NEG	POS	NEG	NEG	POS	NEG	POS	NEG	POS	NEG	POS	POS	NEG	NEG	NEG	POS
CC398-MRSA-IV	NEG	POS	POS	NEG	POS	NEG	AMB	NEG	POS	NEG	NEG	POS	POS	AMB	POS	NEG	NEG	NEG	NEG
ST398-MRSA-V	NEG	POS	POS	NEG	POS	NEG	AMB	NEG	POS	NEG	AMB	POS	POS	NEG	POS	NEG	NEG	NEG	NEG
ST398-MRSA-V [PVL+]	NEG	POS	POS	NEG	POS	NEG	NEG	NEG	POS	NEG	NEG	POS	POS	NEG	POS	NEG	NEG	NEG	NEG
CC509/ST207-MRSA-V	NEG	POS	NEG	NEG	NEG	POS	NEG	NEG	POS	POS	NEG	POS	NEG	POS	POS	NEG	NEG	NEG	NEG
ST573-MRSA-V, WA-10	NEG	POS	NEG	POS	NEG	POS	NEG	POS	AMB	POS	AMB	POS	NEG	POS	POS	NEG	NEG	NEG	POS
ST772-MRSA-V [PVL+], WA-60/Bengal Bay Clone	NEG	POS	NEG	POS	NEG	POS	AMB	POS	AMB	POS	AMB	POS	NEG	POS	POS	NEG	NEG	NEG	POS
ST779-MRSA	NEG	POS	NEG	AMB	NEG	POS	NEG	POS	AMB	POS	AMB	POS	NEG	POS	POS	NEG	NEG	NEG	NEG
ST834-MRSA-IV, WA-13	NEG	POS	NEG	NEG	NEG	POS	NEG	POS	AMB	POS	AMB	POS	NEG	POS	POS	NEG	NEG	NEG	NEG
CC913-MRSA-IV	NEG	POS	NEG	NEG	NEG	POS	NEG	POS	POS	POS	POS	POS	NEG	POS	POS	NEG	POS	NEG	NEG
ST1774-MRSA-IV [ACME+]	NEG	POS	NEG	POS	NEG	POS	NEG	NEG	POS	POS	AMB	POS	AMB	POS	NEG	POS	NEG	AMB	NEG
CC15-MSSA	NEG	POS	POS	POS	POS	POS	NEG	POS	AMB	POS	AMB	POS	POS	AMB	POS	NEG	NEG	NEG	NEG
ST426-MSSA	POS	POS	POS	NEG	POS	AMB	AMB	NEG	POS	NEG	AMB	POS	POS	AMB	NEG	POS	NEG	NEG	NEG

Legend:

Positive :	POS
Ambiguous :	AMB
Negative :	NEG
Rare (in >0 to 33% of isolates) :	RARE
Variable (in >33 to 66% of isolates) :	VAR
Common (in >66 to <100% of isolates) :	COMM
